# Genetic and environmental determinants of *O*^6^-methylguanine DNA-methyltransferase (*MGMT*) gene methylation: a 10-year longitudinal study of Danish twins

**DOI:** 10.1186/s13148-021-01009-5

**Published:** 2021-02-15

**Authors:** Lijie Wang, Afsaneh Mohammadnejad, Weilong Li, Jesper Lund, Shuxia Li, Signe Clemmensen, Maria Timofeeva, Mette Soerensen, Jonas Mengel-From, Kaare Christensen, Jacob Hjelmborg, Qihua Tan

**Affiliations:** 1 Epidemiology, Biostatistics and Biodemography, Department of Public Health, University of Southern Denmark, J.B. Winsløws Vej 9 B., 5000 Odense C, Denmark; 2 Department of Epidemiology and Health Statistics, School of Public Health, Cheeloo College of Medicine, Shandong University, Jinan, China; 3 Population Research Unit, Faculty of Social Sciences, University of Helsinki, Helsinki, Finland; 4 Digital Health and Machine Learning Research Group, Hasso Plattner Institute for Digital Engineering, Potsdam, Germany; 5 Unit of Human Genetics, Department of Clinical Research, University of Southern Denmark, Odense, Denmark

**Keywords:** *MGMT*, DNA methylation, CpG site, Twin models, Heritability, Glioma

## Abstract

**Background:**

Epigenetic inactivation of *O*^6^-methylguanine DNA-methyltransferase (*MGMT*) is associated with increased sensitivity to alkylating chemotherapeutic agents in glioblastoma patients. The genetic background underlying *MGMT* gene methylation may explain individual differences in treatment response and provide a clue to a personalized treatment strategy. Making use of the longitudinal twin design, we aimed, for the first time, to estimate the genetic contributions to *MGMT* methylation in a Danish twin cohort.

**Methods:**

DNA-methylation from whole blood (18 monozygotic (MZ) and 25 dizygotic (DZ) twin pairs) repeated 10 years apart from the Longitudinal Study of Aging Danish Twins (LSADT) were used to search for genetic and environmental contributions to DNA-methylation at 170 CpG sites of across the *MGMT* gene. Both univariate and bivariate twin models were applied. The intraclass correlations, performed on cross-sectional data (246 MZ twin pairs) from an independent study population, the Middle-Aged Danish Twins (MADT), were used to assess the genetic influence at each CpG site of *MGMT* for replication.

**Results:**

Univariate twin model revealed twelve CpG sites showing significantly high heritability at intake (wave 1, *h*^2^ > 0.43), and seven CpG sites with significant heritability estimates at end of follow-up (wave 2, *h*^2^ > 0.5). There were six significant CpG sites, located at the gene body region, that overlapped among the two waves (*h*^2^ > 0.5), of which five remained significant in the bivariate twin model, which was applied to both waves. Within MZ pair correlation in these six CpGs from MADT demarks top level of genetic influence. There were 11 CpGs constantly have substantial common environmental component over the 10 years.

**Conclusions:**

We have identified 6 CpG sites linked to the *MGMT* gene with strong and persistent genetic control based on their DNA methylation levels. The genetic basis of *MGMT* gene methylation could help to explain individual differences in glioblastoma treatment response and most importantly, provide references for mapping the methylation Quantitative Trait Loci (meQTL) underlying the genetic regulation.

**Supplementary Information:**

The online version contains supplementary material available at 10.1186/s13148-021-01009-5.

## Background

Glioblastoma (GBM) is the most common malignant brain tumor which is highly fatal as its five-year relative survival is only 6.8% [1, 2]. The current standard therapeutic management for newly diagnosed GBM is maximal safe surgical resection, followed by systematic radiotherapy combined with concomitant and adjuvant temozolomide (TMZ). The addition of TMZ to radiotherapy has successively improved the long-time survival for GBM patients [3]. However, many of the GBM patients are insensitive to alkylating chemotherapeutic agents (e.g., TMZ) and thus cannot get benefit from the standard treatment [4].

One major cause is the silencing of the *O*^6^-methylguanine DNA-methyltransferase (*MGMT*) gene [5–7]. The *MGMT* gene resides in chromosome 10q26 and encodes a DNA-repair enzyme [8, 9]. Aalkylating agents-induced cytotoxicity is triggered by adding its methyl group to specific sites, especially *O*^6^ positions of guanine. The *O*^6^-MeG adduct causes cell killing by inaccurate pairing of methylated guanine with thymine during DNA replication. The MGMT protein restores alkylation-induced DNA lesion by transferring the methyl group from the *O*^6^-MeG adduct to a cysteine residue in its active site irreversibly, thus blunts the therapeutic effect of alkylating agents [10, 11].

The silencing of the *MGMT* gene expression can be affected by both genetic and epigenetic factors [12, 13]. It is widely accepted that the *MGMT* promoter methylation is the leading regulation mechanisms which reduce gene expression. The study of the relationship between gene expression and the methylation patterns of the overall and specific CpG sites [14] in the promoter of *MGMT* has been a topic of wide interest [15]. Everhard et al. [16] found six CpG sites, which were located in the promoter region of *MGMT,* highly correlated with expression in GBMs. Bady et al. [17] identified two distinct regions in the CpG island of the promoter with high importance for *MGMT* silencing in GBM.

Though numerous studies have demonstrated that the *MGMT* promoter methylation status may determine the efficiency of TMZ treatment for the GBM patients [15–17], this biomarker has not yet been used in routine clinical practice to guide therapy for glioblastoma [18]. Methylation could also occur in the *MGMT* gene body. Gene body hypermethylation was positively correlated with MGMT expression in some GBM patients [19], which could partially explain the inconsistencies between the *MGMT* promoter methylation, gene expression level and different patient prognosis.

There are 176 CpG sites annotated for *MGMT* by HumanMethylation450 (450 k) beadchips. The total amount of discrete CpG methylation patterns, and intra-tumoral methylation homogeneity of *MGMT* is variable in GBM [16, 20–22], and also other tumors [23]. In addition, Markus et al. [24] reported that there is considerable variation of MGMT activity in normal tissues. These findings indicate that a degree of inter-individuals methylation heterogeneity and intra-individual variability exists.

DNA methylation is dynamic and changes throughout the life course, while its levels are affected by environmental factors, as well as genetic variation. Cis- or trans-acting genetic factors, known as methylation Quantitative Trait Loci (meQTL) can introduce or disrupt CpG sites and have a significant effect on the methylation status of the specific gene. To our knowledge, genetic contribution or heritability in *MGMT* methylation is not well established and therefore, need more attention. Heritability is estimated by the correlation between genetic sharing and phenotypic sharing. Twin studies are regarded as the some of the best ways for assessing human heritability. Comparison of phenotype correlation in monozygotic (MZ) twin pairs who share their genetic makeups and dizygotic (DZ) twin pairs who share on average half of their genetic materials allows for better interpretation and quantification of genetic factors. Longitudinal twin studies on long term conservation of individual molecular phenotypes contribute to the exploration of genetic and environmental bases for maintaining molecular homeostasis [25–27]. This study introduces, for the first time, the twin design for disease studies to assess the genetic contribution to the molecular phenotype of *MGMT* methylation to provide (1) reference for mapping meQTL of the *MGMT* gene; and (2) explanation to the observed individual differences in treatment response.

## Methods

### Study subjects and blood collection

The study samples were obtained from two independent surveys from the Danish Twin Registry, the Longitudinal Study of Aging Danish Twins (LSADT) launched in 1995 and the Middle-Aged Danish Twins (MADT) conducted in 1998. Eighty-six Danish twins including 18 monozygotic (MZ) and 25 dizygotic (DZ) like-sex pairs were collected by the LSADT. Blood samples were collected twice with a 10-year gap. The first wave blood samples were collected in 1997 with ages ranging from 73.21 to 81.75 years. The second wave of blood samples was taken in 2007 [28]. A total of 246 monozygotic twin pairs, ranging in age from 55.94 to 79.88, were obtained from MADT [29]. Blood samples were collected during the follow-up visit in 2008–2011. Zygosity for LSADT was classified using highly polymorphic microsatellite markers, while zygosity for MADT was based on questions regarding physical similarity [30]. LSADT and MADT study design and data collection have previously been described in details elsewhere [29, 31, 32].

The study was conducted under approval by the Danish Scientific Ethics Committees and in agreement with the Helsinki II declaration. All participants in the surveys have given informed consent.

### Genomic DNA extraction

DNA was extracted from buffy-coat from EDTA anti-coagulant samples and converted with sodium bisulfite by the EZ-96 DNA-methylation kit (Zymo Research, Orange County, USA) following the manufacturer’s protocol [33]. Details of this process have been described by Tan et al [34].

### Array-based DNA methylation profiling

The measurement of genome-wide DNA-methylation was performed on the Infinium HumanMethylation450 (450 k) beadchips (Illumina, San Diego, CA, USA) to obtain the DNA methylation level at 485,512 CpG sites spanning genes and CpG island regions of the human genome. Six pairs of twins were assayed together on the same chip. Subset-quantile Within Array Normalization (SWAN) was performed to reduce technical bias between Type 1 and Type 2 probes by R package *minfi* [35]. Methylation level on each CpG site was calculated by the β value, defining as (the methylated allele intensity) / (methylated + unmethylated allele intensity + 100). β value ranged from 0 to 1, indicating non methylation and 100% methylation respectively [36]. M value (logit of β value with base 2) was used in the following methylation analysis, which avoided the heteroscedastic disadvantage of β value [37].

### Quality control (QC)

For each CpG site being interrogated, there are two site-specific probes, one for methylated and the other for unmethylated loci to which chemically converted DNA is being hybridized. The detection *P* value, which is the proportion of background signal levels in samples for both methylated and unmethylated channels was used to control the probe quality. CpG probes with detection *P* value > 0.01 were regarded as missing. CpG probes harboring Single Nucleotide Polymorphisms (SNPs), and probes with > 5% missing values were excluded from the analysis. We used *minfi* to perform the quality control. After QC, a total of 176 CpGs on the array were linked to the *MGMT* gene. All CpG sites were annotated with the R package *IlluminaHumanMethylation450kanno.ilmn12.hg19* [38].

### Estimating and adjusting cell composition

For each individual, we estimated cell composition based on six blood cell types (CD8T, CD4T, B cell, monocyte, granulocyte and natural killer cell) following the Houseman procedure. The residual values were used for further analysis as the cell type composition was adjusted as covariates in the regression models [39].

### Statistical analysis

In the discovery stage, using longitudinal data from LSADT, the correlation for each CpG site of *MGMT* in MZ and DZ twin pairs at two waves (1997 and 2007) were estimated by the intraclass correlations coefficients. Statistical significance for the difference of the correlation by zygosity were tested based on Fisher’s *z*-test [40]. Heritability analyses of each CpG site of *MGMT* were conducted with two approaches: univariate twin analysis (Additional file 1: Fig. S1 and Additional file 2: Fig. S2) and bivariate twin analysis (Additional file 3: Fig. S3). Meanwhile, cross-sectional data from MADT were used for replication purpose.

### Twin modelling

Based on the polygenic biometric structural equation ADCE model, the methylation variation at each CpG site can be divided into four components, additive genetic (A), dominant genetic (D), common or shared environmental (C) and unique environmental (E). Although both C and D variance components were included in the diagram (Additional file 1: Fig. S1 and Additional file 3: Fig. S3), they were confounded and cannot be estimated together [26, 27].

For the univariate twin analysis, we fitted full ACE and its nested models (AE, CE, and E), along with ADE and its nested models (AE, DE, and E) to methylation value of each of the 176 CpG sites. Goodness of fit of all models was evaluated by the Akaike information criteria (AIC) [41]. We first selected the best full model (BFM) between ACE and ADE models with the minimum AIC. Then we used the same way to get the best nested model (BNM) under the selected best full model. We applied the likelihood ratio test (LRT) which approximately follows the chi-squared distribution, to decide whether BFM or BNM was used. If the *P* value was > 0.05, we used BNM based on the principle of parsimony and testing whether the components, A, C, D and E, are significantly greater than zero. Otherwise, we choose the model between BFM and BNM with the minimum AIC. After we got the best fitted model, the genetic component (A) was extracted and the corresponding heritability (*h*^2^) can be obtained by calculating the proportion of genetic variance among the total variance, i.e. *h*^2^ =$\frac{A}{A+C+E}$ as narrow sense heritability for the ACE model and *h*^2^ =$\frac{A+D}{A+D+E}$ as broad sense heritability for the ADE model.

Secondly, we fit the bivariate twin model to investigate the continuity of genetic influences at different time points (1997 and 2007 waves). The model analyses the genetic and environmental architecture of the covariance between two traits (methylation values at waves 1 and 2). MZ/DZ ratio of the cross-twin cross-traits covariances shows whether it is genetic or environmental factors that influence the traits [27]. The phenotypic (methylation value) within pair correlation is determined by genetic and environmental variance components similarly as in the univariate ADCE model.

In the univariate and bivariate twin models, MZ twin pairs correlate 1 for both additive (A) and dominant (D) genetic factors, whereas DZ twin pairs correlate 0.5 for A and 0.25 for D. Both MZ and DZ pairs correlate 1 for common environment (C), whereas unique environment (E) is uncorrelated in both types of twin pairs. In the bivariate twin model (Additional file 3: Fig. S3), *r*_g_, *r*_*d*_, *r*_c_, and *r*_e_ are the additive genetic, dominant genetic, shared environmental and unique environmental correlations on phenotype levels at the two time points, respectively. Age and sex were adjusted as covariates during the two-step analysis.

### Replication

The significant CpGs with high heritability discovered from twin modelling were supposed to have high correlation in MZ twin pairs. To reconfirm our findings, we calculated the intraclass correlations for each CpG site of *MGMT* to show the correlation level in MZ twin pairs from MADT.

All statistical analyses were performed using the R software (http://www.r-project.org). The univariate and bivariate twin models were performed using the R package *mets* [42, 43] (https://cran.r-project.org/web/packages/mets/) and *OpenMx* [44] (https://cran.r-project.org/web/packages/OpenMx/) respectively. Intraclass correlation was estimated using the R package *mets*.

## Results

### Discovery stage

There were 16 monozygotic and 25 dizygotic twin pairs with complete information both in phenotype and methylation data from LSADT (Table 1). The promoter regions of *MGMT* were defined as 1500 bp and 200 bp upstream the transcription start site (TSS200 and TSS1500). Six CpG sites (cg10502904, cg23004031, cg00198994, cg00657202, cg07638938 and cg26127080) were deleted due to missing values. Thus, 170 CpG sites (23 of promoter regions, 1 of 1st Exon and 5′ UTR, 144 of gene body, 2 of 3′ UTR) were included in the following analysis.

**Table 1:** Descriptive characteristics for longitudinal twin samples from LSADT

	MZ twins			DZ twins			All
	Male	Female	Total	Male	Female	Total	
*n*, pair	6	10	16	5	20	25	41
Age, 1997							
Mean	76.97	76.13	76.45	76.79	76.05	76.19	76.29
Median	76.67	75.61	76.31	75.85	75.68	75.71	75.79
Min	75.21	73.21	73.21	74.66	74.05	74.05	73.21
Max	79.51	81.75	81.75	79.32	79.59	79.59	81.75
Age, 2007							
Mean	86.82	86.04	86.33	86.55	85.86	86.00	86.13
Median	86.27	85.25	85.97	85.95	85.60	85.68	85.73
Min	85.23	83.30	83.30	84.13	84.00	84.00	83.30
Max	89.53	91.70	91.70	89.13	89.49	89.49	91.70

Additional file 4: Table S1 shows the correlation for all CpGs of *MGMT* in MZ and DZ pairs at two time points separately. Overall, only 20 CpGs had significantly higher correlation for MZ twins than DZ twins in both 1997 and 2007 (36 CpGs in 1997, 39CpGs in 2007), an indication of genetic influence in a small proportion of the total CpGs studied. For the gene body region, mean methylation level had higher correlation for MZ twins than those for DZ twins, but with no significance in both waves. Similar pattern was applied to mean methylation level at promoter region with *P* < 0.001 in 2007.

### Univariate twin analysis

Additional file 5: Table S2 shows the process of how the final model was selected. After comparing the AIC of the two full models (ACE and ADE) and the corresponding nested models for each CpG site, as well as the mean of gene body and promoter regions, we found the best full and nested model. Then we did the likelihood ratio test (LRT) to decide the final best-fitted model. All the *P* values from comparison between best nested and best full model were no less than 0.05 in both two waves (1997 and 2007), meaning all the final models were from a nested model (AE, CE, DE and E).

Additional file 6: Table S3 describes the heritability of the best-fitted model derived from Additional file 5: Table S2. All CpG sites, as well as the mean of gene body and promoter regions in both waves were calculated. The heritability changed from 0 to nearly 1 (0.998 for cg09993319) in the 1997 wave and almost the same for the 2007 wave (the maximum was 0.996 for cg09993319). Table 2 describes the top significant 13 CpG sites in the two waves. In the 1997 wave, 12 CpG sites had significantly high proportions of additive genetic covariance ranging from 0.428 (95% CI 0.004–0.852) for cg06179303 to 0.998 (95% CI 0.997–1.000) for cg09993319 as their confidence interval did not include 0. This varied a little in the 2007 wave as 7 CpG sites met that rule. There were 6 CpG sites (cg09993319, cg17686260, cg27275103, cg16255663, cg26201213 and cg06952798), which overlapped among the two waves. All 6 CpG sites had low to moderate unique environmental contribution to their total variation and low E components in their covariance at the two waves. As shown in Additional file 5: Table S2, all the 6 CpG sites are derived from AE model.

**Table 2:** Heritability estimation at top 13 CpG sites of *MGMT* showing high heritability at two time points

CpG site	Year 1997			Year 2007		
	a^2^	c^2^	e^2^	a^2^	c^2^	e^2^
cg09993319^c^	0.9985 (0.9973–0.9997)	0.0000 (0.0000–0.0000)	0.0015 (0.0003–0.0027)	0.9961 (0.9931–0.9991)	0.0000 (0.0000–0.0000)	0.0039 (0.0009–0.0069)
cg27275103^c^	0.9923 (0.9857–0.9990)	0.0000 (0.0000–0.0000)	0.0077 (0.0010–0.0143)	0.9853 (0.9740–0.9966)	0.0000 (0.0000–0.0000)	0.0147 (0.0034–0.0260)
cg16255663^c^	0.9816 (0.9674–0.9958)	0.0000 (0.0000–0.0000)	0.0184 (0.0042–0.0326)	0.9760 (0.9575–0.9946)	0.0000 (0.0000–0.0000)	0.0240 (0.0054–0.0425)
cg17686260^c^	0.9810 (0.9661–0.9960)	0.0000 (0.0000–0.0000)	0.0190 (0.0040–0.0339)	0.9874 (0.9776–0.9972)	0.0000 (0.0000–0.0000)	0.0126 (0.0028–0.0224)
cg26201213^c^	0.7158 (0.4997–0.9320)	0.0000 (0.0000–0.0000)	0.2842 (0.0680–0.5003)	0.5978 (0.3148–0.8807)	0.0000 (0.0000–0.0000)	0.4022 (0.1193–0.6852)
cg06952798^c^	0.5428 (0.2146–0.8711)	0.0000 (0.0000–0.0000)	0.4572 (0.1289–0.7854)	0.5033 (0.0980–0.9086)	0.0000 (0.0000–0.0000)	0.4967 (0.0914–0.9020)
cg27483317^a^	0.7057 (0.4854–0.9261)	0.0000 (0.0000–0.0000)	0.2943 (0.0739–0.5146)	0.0000 (0.0000–0.0000)	0.5641 (0.2106–0.9176)	0.4359 (0.0824–0.7894)
cg09757049^a^	0.5358 (0.1247–0.9469)	0.0000 (0.0000–0.0000)	0.4642 (0.0531–0.8753)	0.0000 (0.0000–0.0000)	0.0000 (0.0000–0.0000)	1.0000 (1.0000–1.0000)
cg10215460^a^	0.5175 (0.2026–0.8324)	0.0000 (0.0000–0.0000)	0.4825 (0.1676–0.7974)	0.0000 (0.0000–0.0000)	0.0000 (0.0000–0.0000)	1.0000 (1.0000–1.0000)
cg14668152^a^	0.5124 (0.2010–0.8237)	0.0000 (0.0000–0.0000)	0.4876 (0.1763–0.7990)	0.0000 (0.0000–0.0000)	0.0000 (0.0000–0.0000)	1.0000 (1.0000–1.0000)
cg14273607^a^	0.4750 (0.0226–0.9275)	0.0000 (0.0000–0.0000)	0.5250 (0.0725–0.9774)	0.0000 (0.0000–0.0000)	0.0000 (0.0000–0.0000)	1.0000 (1.0000–1.0000)
cg06179303^a^	0.4279 (0.0041–0.8516)	0.0000 (0.0000–0.0000)	0.5721 (0.1484–0.9959)	0.0000 (0.0000–0.0000)	0.0000 (0.0000–0.0000)	1.0000 (1.0000–1.0000)
cg02792401^b^	0.0000 (0.0000–0.0000)	0.0000 (0.0000–0.0000)	1.0000 (1.0000–1.0000)	0.5255 (0.0644–0.9866)	0.0000 (0.0000–0.0000)	0.4745 (0.0134–0.9356)

^a^^,b^Represent significant CpG sites showing high heritability with 0 not included in the 2.5–97.5% confidence intervals in 1997 and 2007, respectively

^c^Represents overlapped significant CpG sites between 1997 and 2007

Table 3 shows 11 CpGs (cg03751055, cg25063211, cg07933035, cg12434587, cg26102564, cg18811130, cg12981137, cg02941816, cg13474692, cg02750154, cg18581292) with significant common environmental component overlapped between 1997 and 2007 ranging from 0.358 (95% CI 0.069–0.648) for cg18581292 to 0.979 (95% CI 0.963–0.996) for cg03751055 (31 CpGs in year 1997, 27 CpGs in year 2007, respectively), from the results of Additional file 5: Table S2 and Additional file 6: Table S3.

**Table 3:** Heritability estimation at top 47 CpG sites of *MGMT* with common environmental component at two time points

CpG site	Year 1997				Year 2007		
	a^2^	c^2^	e^2^		a^2^	c^2^	e^2^
cg03751055^c^	0.0000 (0.0000–0.0000)	0.9792 (0.9629–0.9955)	0.0208 (0.0045–0.0371)		0.0000 (0.0000–0.0000)	0.9841 (0.9716–0.9966)	0.0159 (0.0034–0.0284)
cg25063211^c^	0.0000 (0.0000–0.0000)	0.8925 (0.8297–0.9554)	0.1075 (0.0446–0.1703)		0.0000 (0.0000–0.0000)	0.6785 (0.4072–0.9498)	0.3215 (0.0502–0.5928)
cg07933035^c^	0.0000 (0.0000–0.0000)	0.8541 (0.7232–0.9851)	0.1459 (0.0149–0.2768)		0.0000 (0.0000–0.0000)	0.8015 (0.6090–0.9941)	0.1985 (0.0059–0.3910)
cg12434587^c^	0.0000 (0.0000–0.0000)	0.8136 (0.6495–0.9777)	0.1864 (0.0223–0.3505)		0.0000 (0.0000–0.0000)	0.8186 (0.6494–0.9878)	0.1814 (0.0122–0.3506)
cg26102564^c^	0.0000 (0.0000–0.0000)	0.7358 (0.5942–0.8774)	0.2642 (0.1226–0.4058)		0.0000 (0.0000–0.0000)	0.8214 (0.7199–0.9229)	0.1786 (0.0771–0.2801)
cg18811130^c^	0.0000 (0.0000–0.0000)	0.6892 (0.5240–0.8544)	0.3108 (0.1456–0.4760)		0.0000 (0.0000–0.0000)	0.7466 (0.6094–0.8838)	0.2534 (0.1162–0.3906)
cg12981137^c^	0.0000 (0.0000–0.0000)	0.6138 (0.2981–0.9295)	0.3862 (0.0705–0.7019)		0.0000 (0.0000–0.0000)	0.7168 (0.4396–0.9940)	0.2832 (0.0060–0.5604)
cg02941816^c^	0.0000 (0.0000–0.0000)	0.5689 (0.1537–0.9841)	0.4311 (0.0159–0.8463)		0.0000 (0.0000–0.0000)	0.7174 (0.4562–0.9786)	0.2826 (0.0214–0.5438)
cg13474692^c^	0.0000 (0.0000–0.0000)	0.5595 (0.1543–0.9646)	0.4405 (0.0354–0.8457)		0.0000 (0.0000–0.0000)	0.6074 (0.1231–1.0917)	0.3926 (− 0.0917–0.8769)
cg02750154^c^	0.0000 (0.0000–0.0000)	0.5114 (0.2660–0.7567)	0.4886 (0.2433–0.7340)		0.0000 (0.0000–0.0000)	0.7505 (0.6022–0.8988)	0.2495 (0.1012–0.3978)
cg18581292^c^	0.0000 (0.0000–0.0000)	0.3584 (0.0683–0.6484)	0.6416 (0.3516–0.9317)		0.0000 (0.0000–0.0000)	0.4864 (0.2308–0.7420)	0.5136 (0.2580–0.7692)
cg06566239^a^	0.0000 (0.0000–0.0000)	0.6584 (0.4027–0.9141)	0.3416 (0.0859–0.5973)		0.0000 (0.0000–0.0000)	0.0000 (0.0000–0.0000)	1.0000 (1.0000–1.0000)
cg00618725^a^	0.0000 (0.0000–0.0000)	0.6405 (0.2705–1.0106)	0.3595 (− 0.0106–0.7295)		0.0000 (0.0000–0.0000)	0.0000 (0.0000–0.0000)	1.0000 (1.0000–1.0000)
cg14312783^a^	0.0000 (0.0000–0.0000)	0.5993 (0.2752–0.9234)	0.4007 (0.0766–0.7248)		0.0000 (0.0000–0.0000)	0.3585 (− 1.1327–1.8496)	0.6415 (− 0.8496–2.1327)
cg24420981^a^	0.0000 (0.0000–0.0000)	0.5814 (0.0205–1.1423)	0.4186 (− 0.1423–0.9795)		0.0000 (0.0000–0.0000)	0.0000 (0.0000–0.0000)	1.0000 (1.0000–1.0000)
cg11223735^a^	0.0000 (0.0000–0.0000)	0.5367 (0.1905–0.8829)	0.4633 (0.1171–0.8095)		0.4334 (NaN–NaN)	0.0000 (0.0000–0.0000)	0.5666 (NaN–NaN)
cg16648911^a^	0.0000 (0.0000–0.0000)	0.5177 (0.2552–0.7802)	0.4823 (0.2198–0.7448)		0.0000 (0.0000–0.0000)	0.0000 (0.0000–0.0000)	1.0000 (1.0000–1.0000)
cg16698623^a^	0.0000 (0.0000–0.0000)	0.5145 (0.2241–0.8050)	0.4855 (0.1950–0.7759)		0.0000 (0.0000–0.0000)	0.0000 (0.0000–0.0000)	1.0000 (1.0000–1.0000)
cg17307615^a^	0.0000 (0.0000–0.0000)	0.5057 (0.1429–0.8685)	0.4943 (0.1315–0.8571)		0.0000 (0.0000–0.0000)	0.0000 (0.0000–0.0000)	1.0000 (1.0000–1.0000)
cg19806483^a^	0.0000 (0.0000–0.0000)	0.4869 (0.0110–0.9628)	0.5131 (0.0372–0.9890)		0.0000 (0.0000–0.0000)	0.0000 (0.0000–0.0000)	1.0000 (1.0000–1.0000)
cg18485261^a^	0.0000 (0.0000–0.0000)	0.4861 (0.0492–0.9230)	0.5139 (0.0770–0.9508)		0.0000 (0.0000–0.0000)	0.0000 (0.0000–0.0000)	1.0000 (1.0000–1.0000)
cg11948988^a^	0.0000 (0.0000–0.0000)	0.4843 (0.0623–0.9064)	0.5157 (0.0936–0.9377)		0.0000 (0.0000–0.0000)	0.0000 (0.0000–0.0000)	1.0000 (1.0000–1.0000)
cg25946389^a^	0.0000 (0.0000–0.0000)	0.4715 (0.0162–0.9269)	0.5285 (0.0731–0.9838)		0.0000 (0.0000–0.0000)	0.0000 (0.0000–0.0000)	1.0000 (1.0000–1.0000)
cg17380475^a^	0.0000 (0.0000–0.0000)	0.4228 (0.1566–0.6890)	0.5772 (0.3110–0.8434)		0.0000 (0.0000–0.0000)	0.0000 (0.0000–0.0000)	1.0000 (1.0000–1.0000)
cg19680672^a^	0.0000 (0.0000–0.0000)	0.4005 (0.1212–0.6798)	0.5995 (0.3202–0.8788)		0.0000 (0.0000–0.0000)	0.0000 (0.0000–0.0000)	1.0000 (1.0000–1.0000)
cg26528551^a^	0.0000 (0.0000–0.0000)	0.3832 (0.1061–0.6604)	0.6168 (0.3396–0.8939)		0.0000 (0.0000–0.0000)	0.0000 (0.0000–0.0000)	1.0000 (1.0000–1.0000)
cg15312358^a^	0.0000 (0.0000–0.0000)	0.3783 (0.0587–0.6980)	0.6217 (0.3020–0.9413)		0.0000 (0.0000–0.0000)	0.0000 (0.0000–0.0000)	1.0000 (1.0000–1.0000)
cg19706602^a^	0.0000 (0.0000–0.0000)	0.3529 (0.0763–0.6296)	0.6471 (0.3704–0.9237)		0.0000 (0.0000–0.0000)	0.2628 (− 0.0376–0.5632)	0.7372 (0.4368–1.0376)
cg26976729^a^	0.0000 (0.0000–0.0000)	0.3230 (0.0260–0.6199)	0.6770 (0.3801–0.9740)		0.0000 (0.0000–0.0000)	0.0000 (0.0000–0.0000)	1.0000 (1.0000–1.0000)
cg13171643^a^	0.0000 (0.0000–0.0000)	0.3145 (0.0155–0.6135)	0.6855 (0.3865–0.9845)		0.0000 (0.0000–0.0000)	0.0000 (0.0000–0.0000)	1.0000 (1.0000–1.0000)
cg25557018^a^	0.0000 (0.0000–0.0000)	0.3061 (0.0044–0.6077)	0.6939 (0.3923–0.9956)		0.0000 (0.0000–0.0000)	0.0000 (0.0000–0.0000)	1.0000 (1.0000–1.0000)
cg27000233^b^	0.0000 (0.0000–0.0000)	0.3263 (NaN–NaN)	0.6737 (NaN–NaN)		0.0000 (0.0000–0.0000)	0.3029 (0.1708–0.4349)	0.6971 (0.5651–0.8292)
cg14194875^b^	0.0000 (0.0000–0.0000)	0.2670 (− 0.0342–0.5682)	0.7330 (0.4318–1.0342)		0.0000 (0.0000–0.0000)	0.6596 (0.3137–1.0056)	0.3404 (− 0.0056–0.6863)
cg27483317^b^	0.7057 (0.4854–0.9261)	0.0000 (0.0000–0.0000)	0.2943 (0.0739–0.5146)		0.0000 (0.0000–0.0000)	0.5641 (0.2106–0.9176)	0.4359 (0.0824–0.7894)
cg07367735^b^	0.3773 (− 0.0659–0.8206)	0.0000 (0.0000–0.0000)	0.6227 (0.1794–1.0659)		0.0000 (0.0000–0.0000)	0.5802 (0.0485–1.1119)	0.4198 (− 0.1119–0.9515)
cg05596517^b^	0.3609 (− 0.0911–0.8130)	0.0000 (0.0000–0.0000)	0.6391 (0.1870–1.0911)		0.0000 (0.0000–0.0000)	0.5740 (0.1702–0.9779)	0.4260 (0.0221–0.8298)
cg00639517^b^	0.0000 (0.0000–0.0000)	0.0000 (0.0000–0.0000)	1.0000 (1.0000–1.0000)		0.0000 (0.0000–0.0000)	0.4576 (0.1108–0.8045)	0.5424 (0.1955–0.8892)
cg04540870^b^	0.0000 (0.0000–0.0000)	0.0000 (0.0000–0.0000)	1.0000 (1.0000–1.0000)		0.0000 (0.0000–0.0000)	0.2222 (0.1253–0.3190)	0.7778 (0.6810–0.8747)
cg05068430^b^	0.0000 (0.0000–0.0000)	0.0000 (0.0000–0.0000)	1.0000 (1.0000–1.0000)		0.0000 (0.0000–0.0000)	0.6789 (0.3242–1.0335)	0.3211 (− 0.0335–0.6758)
cg07448909^b^	0.0000 (0.0000–0.0000)	0.0000 (0.0000–0.0000)	1.0000 (1.0000–1.0000)		0.0000 (0.0000–0.0000)	0.3512 (0.0368–0.6656)	0.6488 (0.3344–0.9632)
cg09154334^b^	0.0000 (0.0000–0.0000)	0.0000 (0.0000–0.0000)	1.0000 (1.0000–1.0000)		0.0000 (0.0000–0.0000)	0.3255 (0.0383–0.6127)	0.6745 (0.3873–0.9617)
cg11019008^b^	0.0000 (0.0000–0.0000)	0.0000 (0.0000–0.0000)	1.0000 (1.0000–1.0000)		0.0000 (0.0000–0.0000)	0.4915 (0.0430–0.9400)	0.5085 (0.0600–0.9570)
cg11216456^b^	0.0000 (0.0000–0.0000)	0.0000 (0.0000–0.0000)	1.0000 (1.0000–1.0000)		0.0000 (0.0000–0.0000)	0.3477 (0.0523–0.6431)	0.6523 (0.3569–0.9477)
cg11235543^b^	0.0000 (0.0000–0.0000)	0.0000 (0.0000–0.0000)	1.0000 (1.0000–1.0000)		0.0000 (0.0000–0.0000)	0.3398 (0.2386–0.4410)	0.6602 (0.5590–0.7614)
cg23235067^b^	0.0000 (0.0000–0.0000)	0.0000 (0.0000–0.0000)	1.0000 (1.0000–1.0000)		0.0000 (0.0000–0.0000)	0.4256 (0.1038–0.7473)	0.5744 (0.2527–0.8962)
cg24747557^b^	0.0000 (0.0000–0.0000)	0.0000 (0.0000–0.0000)	1.0000 (1.0000–1.0000)		0.0000 (0.0000–0.0000)	0.3091 (0.0123–0.6058)	0.6909 (0.3942–0.9877)
cg27429313^b^	0.0000 (0.0000–0.0000)	0.0000 (0.0000–0.0000)	1.0000 (1.0000–1.0000)		0.0000 (0.0000–0.0000)	0.3577 (0.0478–0.6677)	0.6423 (0.3323–0.9522)

^a,b^Represent significant CpG sites with significant common environmental component with 0 not included in the 2.5–97.5% confidence intervals in 1997 and 2007, respectively

^c^Represents overlapped significant CpG sites between 1997 and 2007 with common environmental component

**Fig. 1:**
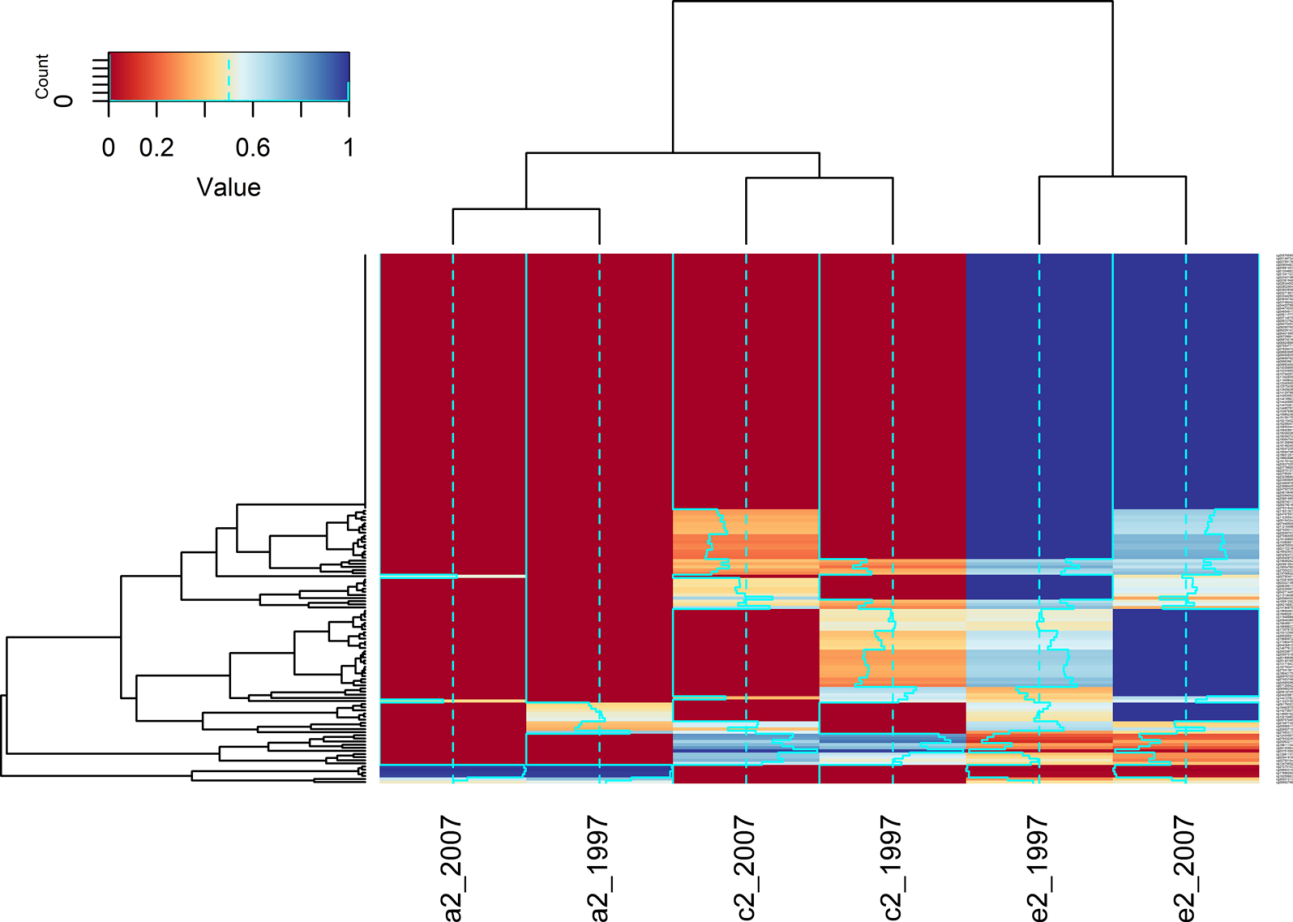
Heatmap of heritability in all CpG sites of *MGMT*

Figure 1 shows the heatmap of heritability in all CpG sites. It shows the high genetic control is stable over time but moderate genetic control (yellow) disappeared after 10 years. Figure 1 also suggests that there were CpGs constantly having substantial C component over the 10 years. For mean methylation levels at promoter and gene body region, the heritability estimates were all 0 in both waves, but with significant common environment contributions (approximately 0.70) in both waves for gene body region and significant common environment component (0.641, 95% CI 0.417–0.865) only in year 2007 for promoter region.

Table 4 shows the annotation information of the identified 6 CpGs with heritability. All the CpG sites were located in the gene body region. One CpG site, cg26201213 was in the CpG Island of S-Shore. Four CpG sites (cg06952798, cg16255663, cg09993319, and cg27275103) were CpG-SNPs, and minor allele frequencies (MAFs) of these SNPs in the European population were all greater than 0.05.

**Table 4:** Annotation file of the significant CpG sites with high genetic control

CpG site	Gene (Name)	Gene (Group)	Position^a^	CpG-SNP^b^	Alleles^d^	MAF^c^ of CpG-SNPs	CpG Island (Name)	Relation_to_Island
cg06952798	MGMT	Body	131,356,999	rs78877238	C/T	0.05(T)		OpenSea
cg27275103	MGMT	Body	131,477,739	rs77705384	C/T	0.14(T)		OpenSea
cg16255663	MGMT	Body	131,350,999	rs61859885	G/A	0.09(A)		OpenSea
cg09993319	MGMT	Body	131,529,435	rs7898151	G/A/T	0.47(G)		OpenSea
cg17686260	MGMT	Body	131,412,764					OpenSea
cg26201213	MGMT	Body	131,265,796				chr10:131,264,948–131,265,710	S_Shore

^a^Position is for reference genome build hg19/GRCh37

^b^CpG-SNP corresponds to the SNPs present at the CpG interrogation

^c^MAF represents minor allele frequency, frequency of the second most frequent allele in 1000 Genomes European population. MAF of CpG-SNPs was obtained from Ensembl for hg19 (http://grch37.ensembl.org/)

^d^Reference/Alternative alleles (Forward strand)

**Fig. 2:**
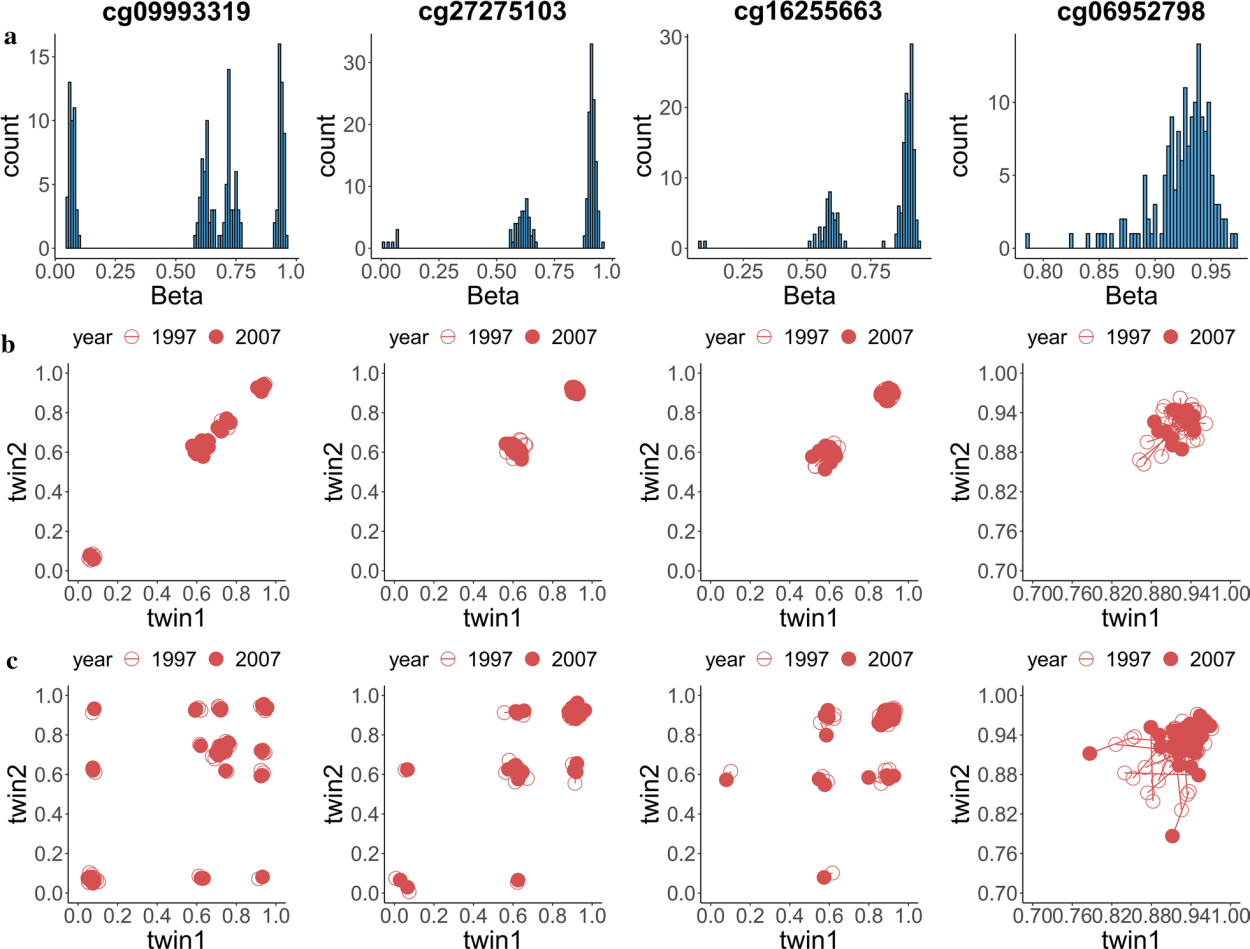
Histogram (**a**), scatter plot for MZ twins (**b**) and DZ twins (**c**) of the beta value for the 4 CpG-SNPs which are significant in both waves. The scatter plots are symmetric by the twin1 = twin2 line, as each twin pair of the same wave was plotted twice

**Fig. 3:**
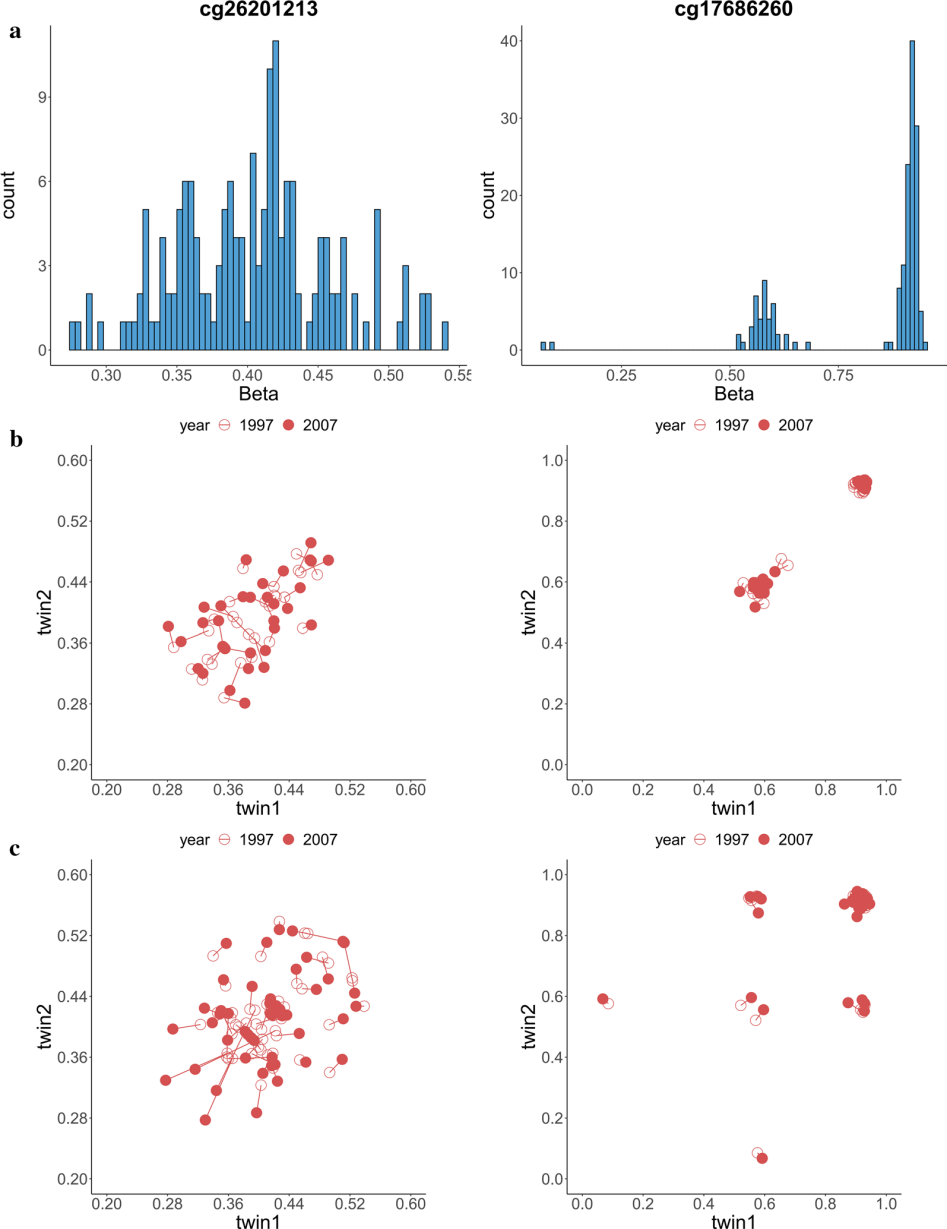
Histogram (**a**), scatter plot for MZ twins (**b**) and DZ twins (**c**) of the beta value for the 2 non CpG-SNPs which are significant in both waves. The scatter plots are symmetric by the twin1 = twin2 line, as each twin pair of the same wave was plotted twice

Figure 2 shows the histogram and scatter plots for the beta values of the 4 CpG-SNPs significant in both waves. Obviously, the histograms for methylation levels of the 3 CpGs (cg09993319, cg27275103, and cg16255663) displayed triple peak patterns. The less obvious peaks for cg06952798 might be due to the MAF of the SNP is 0.05, too small to have sufficient people to have three genotypes in this small size sample. Figure 3 shows the histogram and scatter plots for the 2 non CpG-SNPs significant in both waves. The histogram for cg26201213 showed a continuous pattern with a single peak. In contrast, there was a triple peak pattern for cg17686260. The scatter plots of MZ and DZ in both Figs. 2 and 3 indicated the methylation value changed a little during the 10 years. The scatter plot of the other 164 CpG sites are shown in Additional file 7: Fig. S4.

### Bivariate twin analysis

We next analyzed each CpG site by fitting the bivariate twin model for the methylation measurements at the two waves. As shown in Additional file 8: Table S4, except for cg06952798, 5 of the 6 CpG sites remained significant during the bivariate analysis with very high genetic correlation (1.000) in methylation value between the two waves. Except for cg26201213, the unique environment had low correlation of the two times ranging from − 0.260 to 0.228. Additional file 9: Fig. S5 shows the boxplot of covariance of additive genetic, shared environmental, dominant genetic, and unique environmental proportion.

### Replication stage

**Fig. 4:**
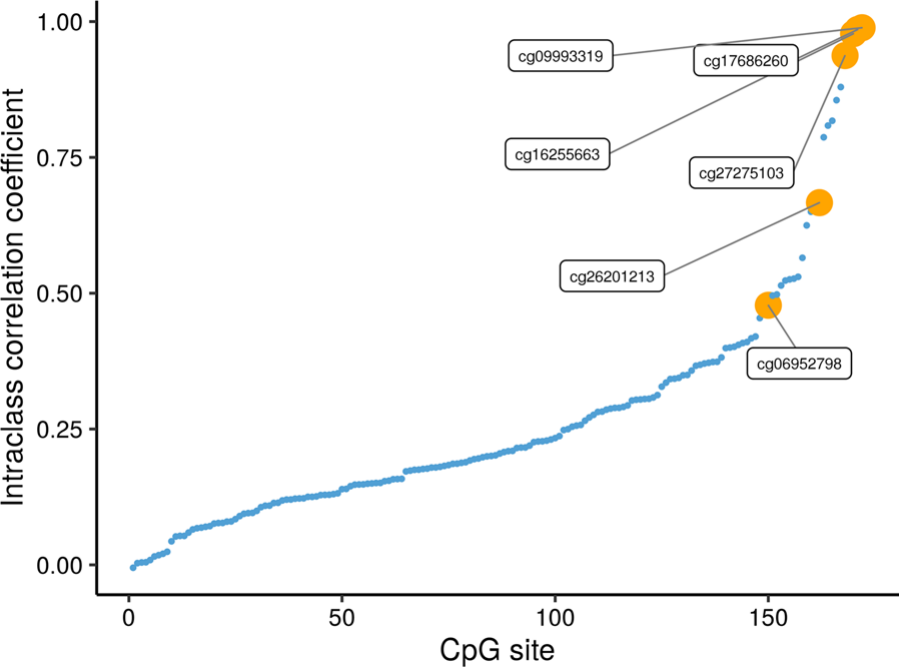
The correlation coefficient plot of MZ twin pairs. The 6 CpG sites, which are significant in both waves are shown in yellow

There were 246 pairs of MZ twins in MADT cohort with 133 male twin pairs (54.07%). The median age was 66.01. After QC, 172 CpGs were included, and the four dropped CpGs (cg00198994, cg00657202, cg07638938, cg26127080) overlapped with the dropped CpGs in discovery LSADT cohort. Figure 4 shows the correlation coefficient plot of the MZ twin pairs. The above 6 CpGs were all located at the upper left. Additional file 10: Fig. S6 shows the histogram and scatter plots for the 6 CpGs. The patterns for each CpG remained the same as in the discovery stage.

## Discussion

Using longitudinal data from Danish twins, we have assessed, for the first time, how heritable and environmental factors affect the variation in the DNA methylation level at individual CpG sites of the *MGMT* gene between two repeated measures spanning 10 years (from 1997 to 2007). Univariate and bivariate twin models were fitted on each CpG site of *MGMT* in order to show their longitudinal changes. Our results highlight the important effect of genetic factors contribute to six specific CpG sites (cg09993319, cg2727510, cg16255663, cg06952798, cg17686260, and cg26201213, all located in the gene body region), with highest genetic factors accounting for over 99% (99.85% in 1997 and 99.61% in 2007) of total variance for cg09993319, and lowest genetic factors accounting for 60%-70% of total variance for cg26201213. Other 11 CpGs (cg03751055, etc.) constantly have substantial common environmental component over the 10 years. The rest CpG sites, 90% of the whole CpG sites of *MGMT*, are not significantly heritable and explained solely by individual unique environment. All the findings we got were reconfirmed well in the younger population.

The estimated genetic components in *MGMT* methylation can pave the way for identifying the genetic variants underlying specific methylation site of *MGMT*. For example, genome-wide association studies (GWAS) can be performed to detect meQTLs with SNPs that regulate the methylation of CpG sites with high heritability. There have been many studies on the association between *MGMT* promoter CpG sites and glioma-related candidate SNPs [45, 46]. Rapkins et al. [47] reported the T allele of the rs16906252 promoter SNP has a significant role in the acquisition of *MGMT* methylation in GBM and is an indicator of response to temozolomide. Candiloro et al. [48] showed that *MGMT* promoter methylation in the peripheral blood of normal individuals is strongly associated with the T Allele of the rs16906252 SNP. Xu et al. [49] suggested that it is the *MGMT* haplotypes, instead of individual SNPs, which control MGMT transcription in healthy individuals and probably have a strong responsibility in sensitivity to alkylating chemotherapeutic agents.

However, the reported meQTLs are mostly local and cis-regulated, instead of genome scale. As CpGs with high heritability estimates are mainly controlled by genetic factors, the six identified CpGs in this study can serve as important and valuable targets for efficient meQTL mapping using a GWAS approach to look for genetic variants that regulate the methylation levels of these CpGs through either cis- or trans-regulation in the genome, with the identified meQTLs as biomarkers for intervention purposes to improve individual treatment response in cancer patients.

Of the six identified CpGs with high heritability, four CpGs (cg09993319, cg2727510, cg16255663, and cg06952798) are CpG-SNPs, which means that there is a genetic variation across samples at this “C–G” location. Most of them are major alleles with C/G. Because if the CpG-SNPs are very rare or minor allele, the methylation level will be low. Then we cannot estimate with power to detect this high heritability allele. The finding of CpG-SNPs as major heritable sites indicates that our twin modeling is capable of capturing methylation sites under genetic control. Meanwhile, the three peaks correspond to each genotype. Considering the high heritability estimates for these CpGs, the methylation of these CpGs can be dependent on genotype as meQTL. Allele-specific methylation (ASM) at CpG-SNPs, which is one specific type of cis-meQTL, has been shown as an important mechanism through which genetic variation regulation function of a gene involving, for example, gene splicing [50] and genomic imprinting [51]. The detected CpGs with high heritability indicate individual genetic variation could regulate *MGMT* activity through ASM.

In contrast, two CpGs (cg17686260 and cg26201213) are not the CpG-SNPs. The histogram for cg17686260 also shows a triple peak pattern. Considering the very high heritability estimates for this CpG (> 0.98), it can be highly likely that methylation of this CpG can be controlled by an adjacent genetic variant as a cis-meQTL. If this is the case, mapping of relevant meQTLs can be done with ease with genetic variation data within the *MGMT* gene body region. After searching on UCSC (http://genome.ucsc.edu/) and Ensembl (http://grch37.ensembl.org/) based on human genome hg19 reference, we found that there is one common SNP (rs61482214, reference/alternative alleles: G/–, MAF: 0.11) which located 1 bp distance away from cg17686260.

The other CpG site, cg26201213, even is not the CpG-SNP, still has high genetic control (0.6–0.7). The histograms for the other CpG site, cg26201213, shows a continuous pattern with a single peak. Such a pattern could imply that the corresponding CpG is under control of multiple factors (here mainly genetic) potentially with both cis- and trans-meQTLs. Our finding can motivate for other study to perform GWAS to detect meQTLs with SNPs nearby or on other chromosomes that regulate the methylation of this CpG site. In sum, our twin modeling not only assesses the genetic contribution to site-specific DNA methylation, but also provides valuable information that can guide the meQTL mapping practice.

It is interesting that the CpGs under consistently high genetic control are all located in *MGMT* gene body. Gene body methylation is usually thought of as having an opposite effect to promoter methylation for gene expression effect [52, 53]. The high genetic contribution to *MGMT* methylation in the gene body suggests that previous efforts on the genetics of *MGMT* methylation could have been biased toward the promoter region. In the literature, higher levels of gene body cytosine modification were correlated with higher *MGMT* expression levels, and also associated with glioblastoma treatment response [13, 19]. Combined with our results, more genetic association studies should be encouraged to look for genetic variants underlying gene body methylation and its clinical consequences. In the future, we aim for including glioblastoma patients to help to specifically clarify the direct roles of the detected CpGs for their clinical implications.

The study has also found 11 CpGs with significant common environmental component. Epidemiology studies may help to explain how these CpGs are constantly impacted by environmental factors (shared family environment or early-life environment). Early-life environment are important for regulating methylation of these CpGs. It would be interesting to look for specific early-life environmental factors that are involved. This information can be useful for prevention and clinical intervention purposes.

Our study was advantaged by its design. First, the longitudinal design allowed us to compare and verify our parameter estimates between the two waves which helped us to reduce chance findings. Results from univariate modeling at the two waves replicated each other to some degree, and were further verified by incorporating the classical twin method with longitudinal bivariate twin models. Second, to provide more stable and confident result, we looked at a much bigger cohort with MZ twins. We cannot calculate heritability of CpG sites. But if they had high genetic control, they should also have high correlation in MZ twins. These six CpGs were on top after we ranked the ICC on methylation. It was somewhat reconfirmed that the estimation was stable and completely reliable.

Our study also has limitations. First, our sample size for discovery (16 MZ twin-pairs and 25 DZ twin-pairs) is not large. As a consequence, only CpGs with high genetic contribution were detected as significant. However, we replicated successfully in an independent cohort. Second, our findings are based on the genomic DNA from blood tissue and it is unclear whether these findings can be generalized to genomic DNA from other tissues, such as the cancer tissues. In the literature, Markus et al. [24] had discussed that the *MGMT* expression level varies greatly in normal tissues and in some cases is associated with cancer predisposition. For a given individual, the expression level of *MGMT* was likely genetically determined. GWAS on DNA methylation levels of our identified heritable CpGs from the cancer tissues can help to verify our findings.

## Conclusions

In summary, the application of classical twin models to the molecular phenotype of *MGMT*-methylation provides a novel approach for studying the contribution of genetics and environment to the epigenetic regulation of *MGMT* gene activity [54]. Results from our study, upon verification in tumor patients, not only help to explain the individual differences in treatment response in glioblastoma patients, but also provide efficient targets to meQTL mapping with aim for more effective personalized cancer management tailored to specific needs, such as healthcare, prevention and treatment.

## Supplementary Information

**Additional file 1:** **Figure S1.** Path diagram of basic univariate twin model.

**Additional file 2:** **Figure S2.** Workflow of univariate twin analysis based on the ADCE model.

**Additional file 3:** **Figure S3.** Path diagram of bivariate twin model.

**Additional file 4:** **Table S1.** The correlation for each CpG site of *MGMT* in monozygotic and dizygotic twins at two time points.

**Additional file 5:** **Table S2.** Model selection process of 1997 year and 2007 year.

**Additional file 6:** **Table S3.** Heritability estimation at each CpG site of *MGMT* in two time points.

**Additional file 7:** **Figure S4.** Scatter plot for MZ twins and DZ twins of the beta value for all the other non-significant CpG sites of MGMT.

**Additional file 8:** **Table S4.** Bivariate twin analysis of the significant CpG sites.

**Additional file 9:** **Figure S5.** Boxplot of the 5 CpGs which are significant in bivariate twin model shows the covariance of additive genetic (a^2^), shared environmental (c^2^), dominant genetic (d^2^), and unique environmental proportion (e^2^).

**Additional file 10:** **Figure S6.** Histogram A) and scatter plot for MZ twins B) of the beta value for the 6 CpGs which are significant in both waves in MADT cohort.

## Data Availability

The discovery data was deposited to the NCBI GEO database http://www.ncbi.nlm.nih.gov/geo/ under accession number GSE73115.

## Abbreviations

MGMT: *O*^6^-Methylguanine DNA-methyltransferase
GBM: Glioblastoma
TMZ: Temozolomide
MZ: Monozygotic
DZ: Dizygotic
LSADT: Longitudinal study of aging Danish twins
MADT: Middle-aged Danish twins
*h*^2^: Heritability
meQTL: Methylation quantitative trait loci
SNPs: Single nucleotide polymorphisms
MAFs: Minor allele frequencies
GWAS: Genome-wide association studies
SWAN: Subset-quantile within array normalization
ASM: Allele-specific methylation
