# Supplementary figures and images for: Genetic and environmental determinants of O6-methylguanine DNA-methyltransferase (MGMT) gene methylation: a 10-year longitudinal study of Danish twins
Source: Clin Epigenetics. 2021 Feb 15;13:35. doi: 10.1186/s13148-021-01009-5 (PMC7885436; doi:10.1186/s13148-021-01009-5)

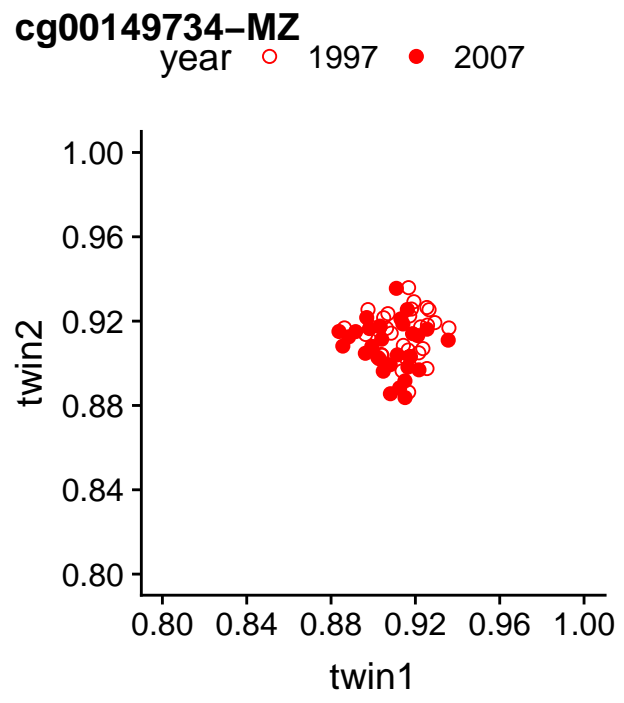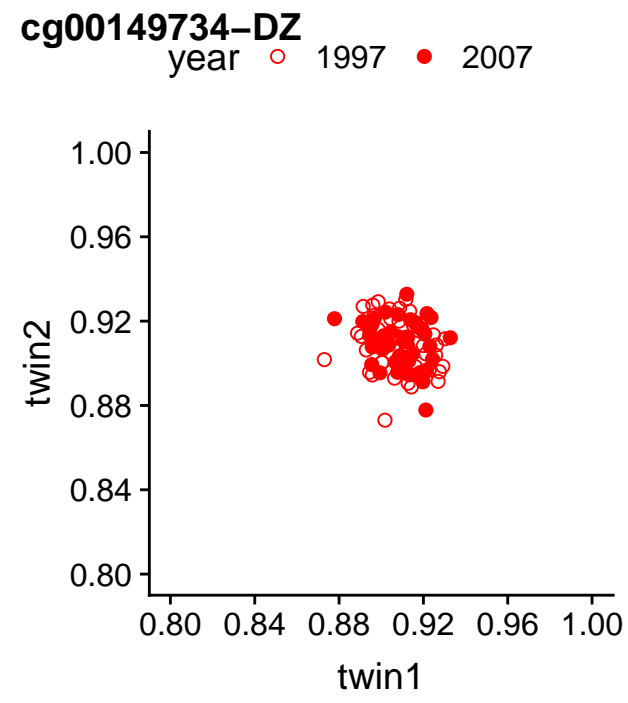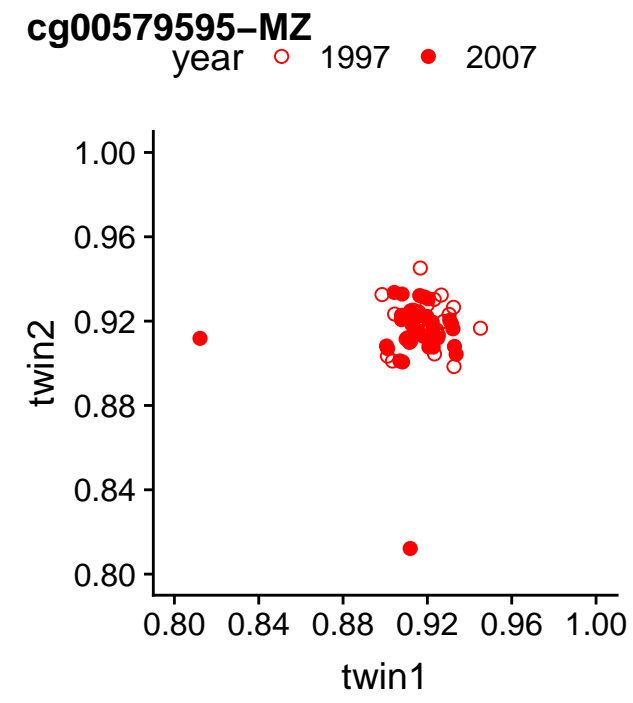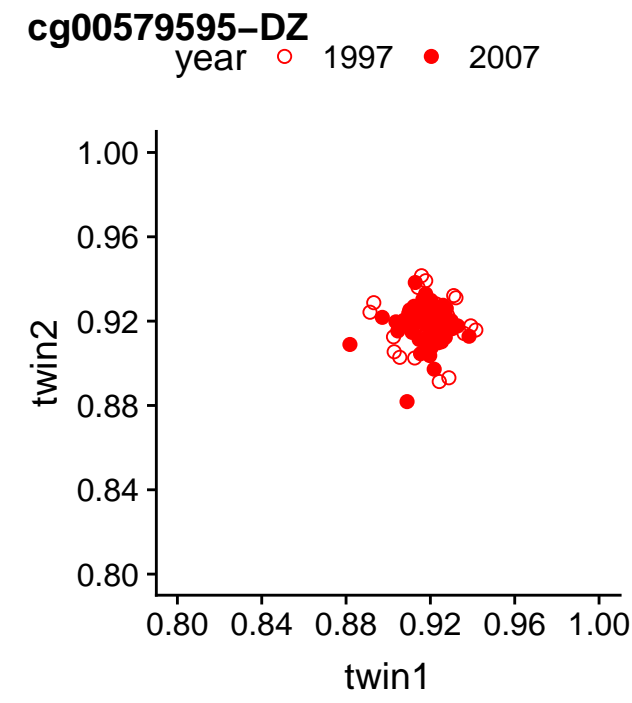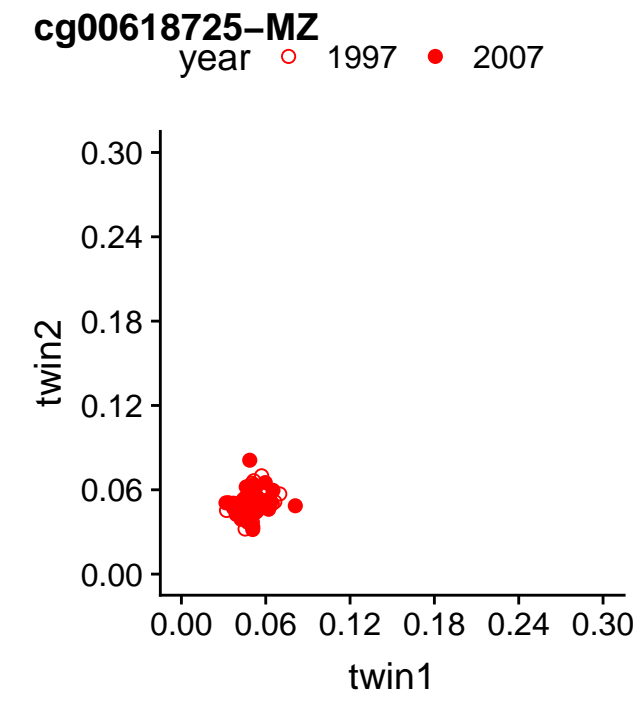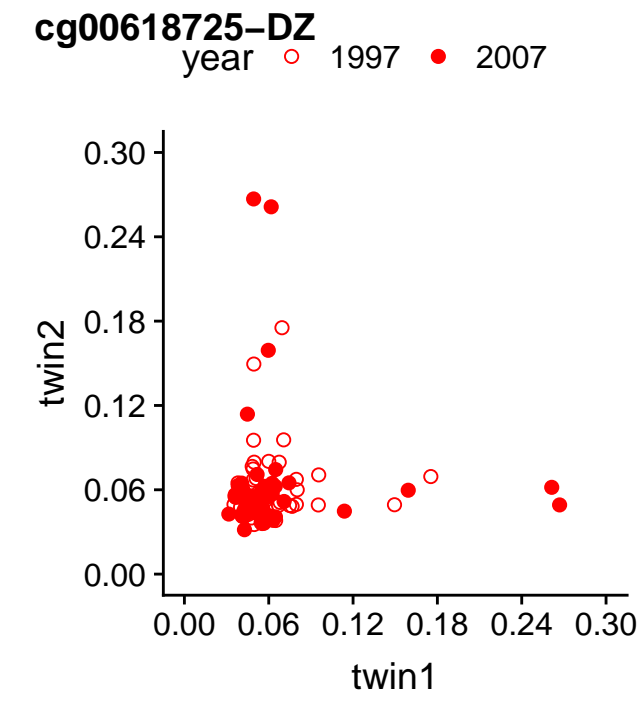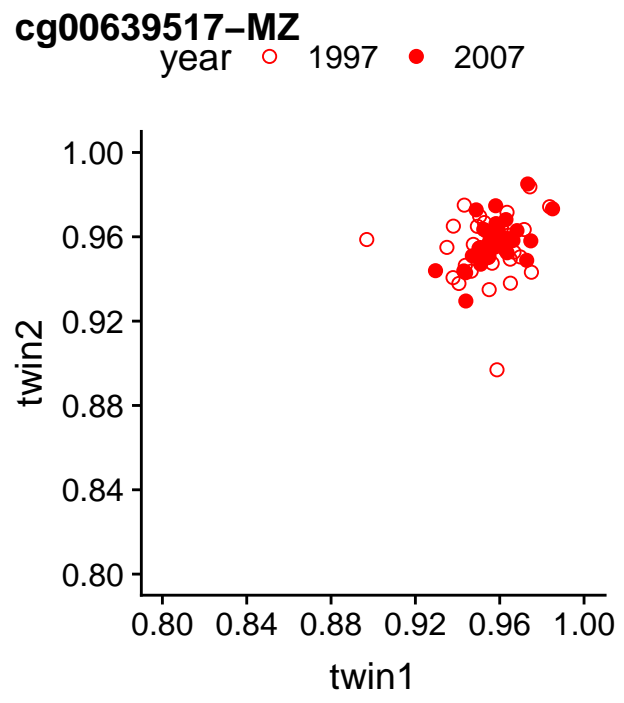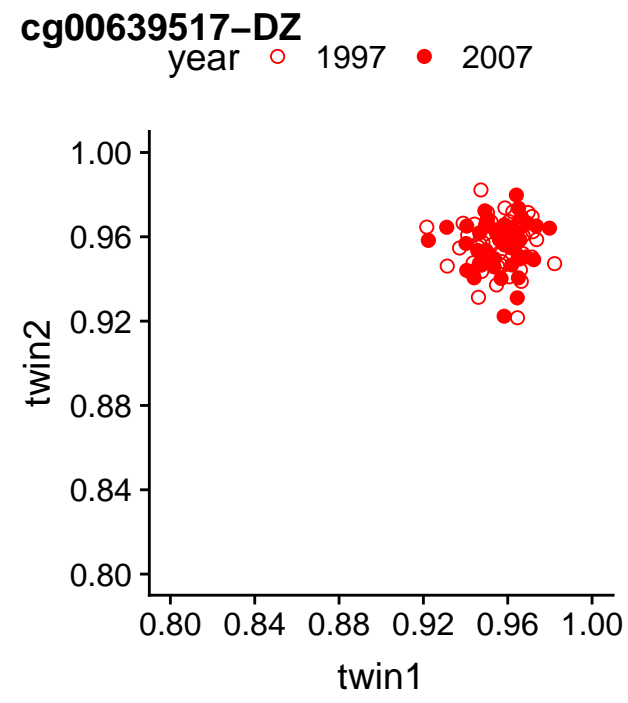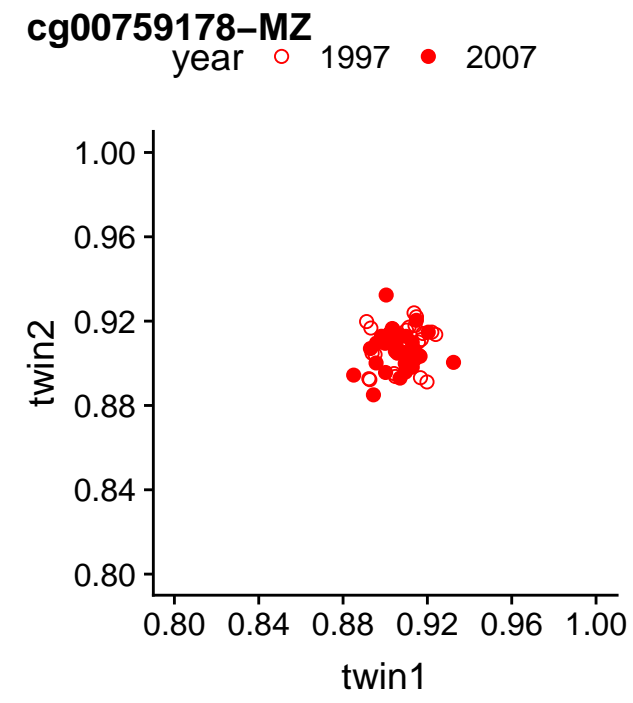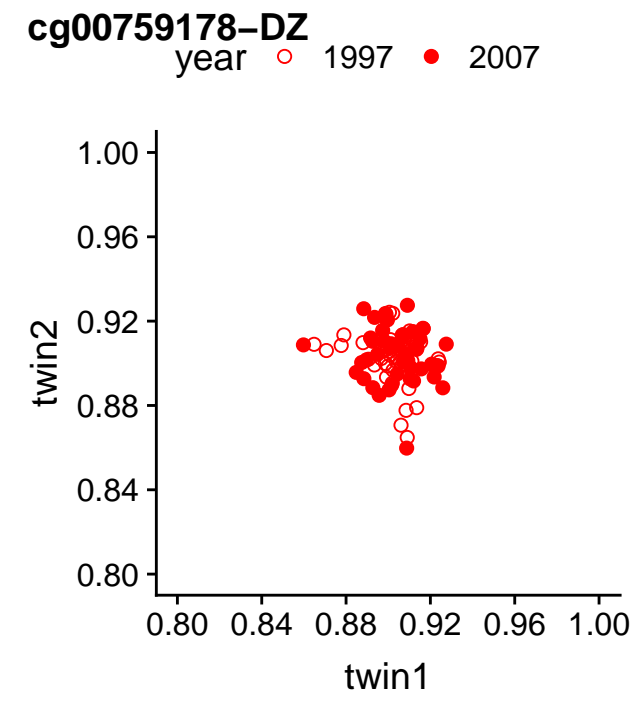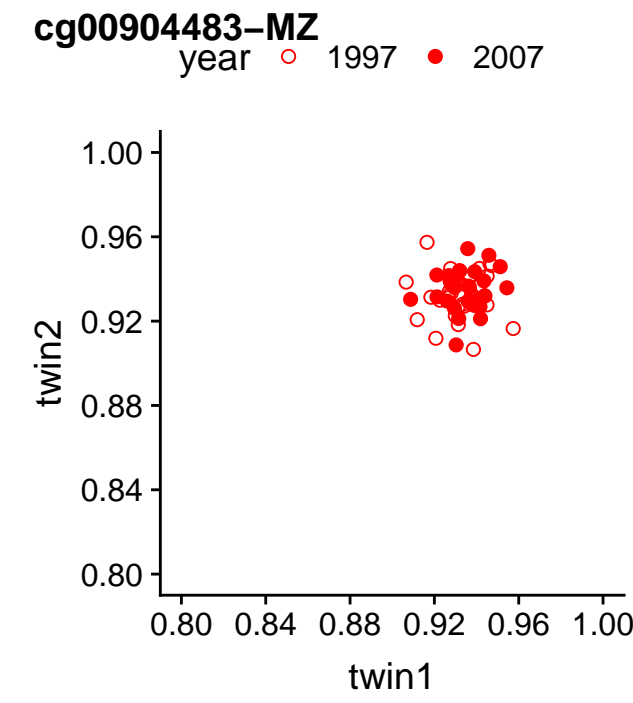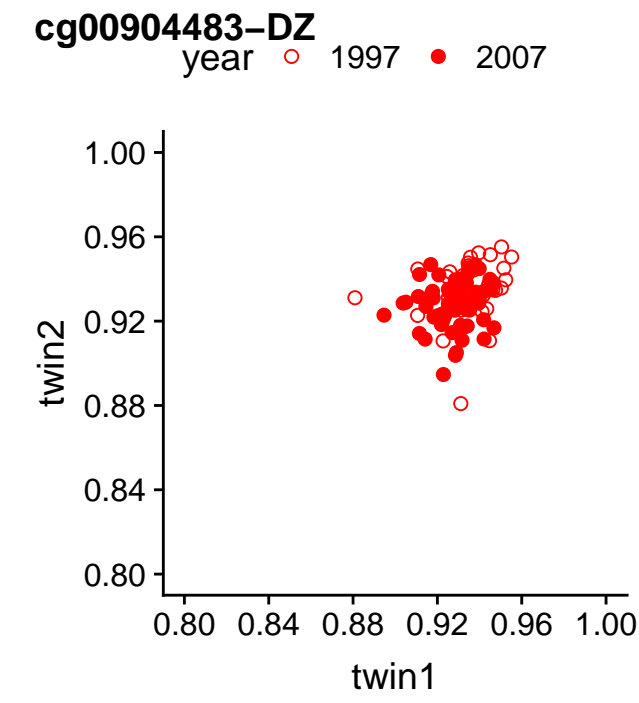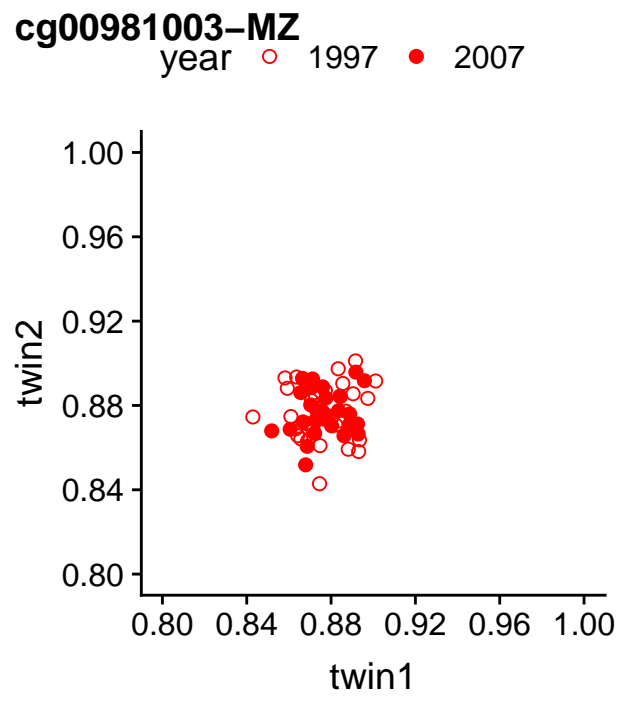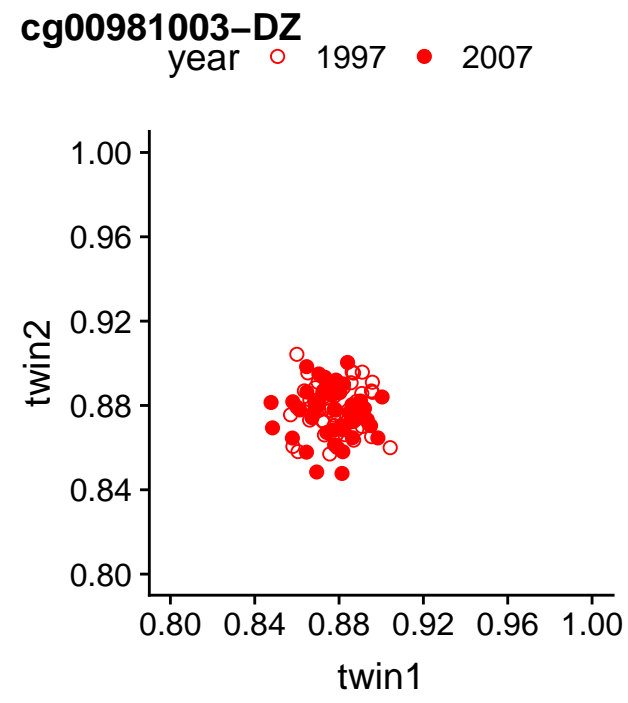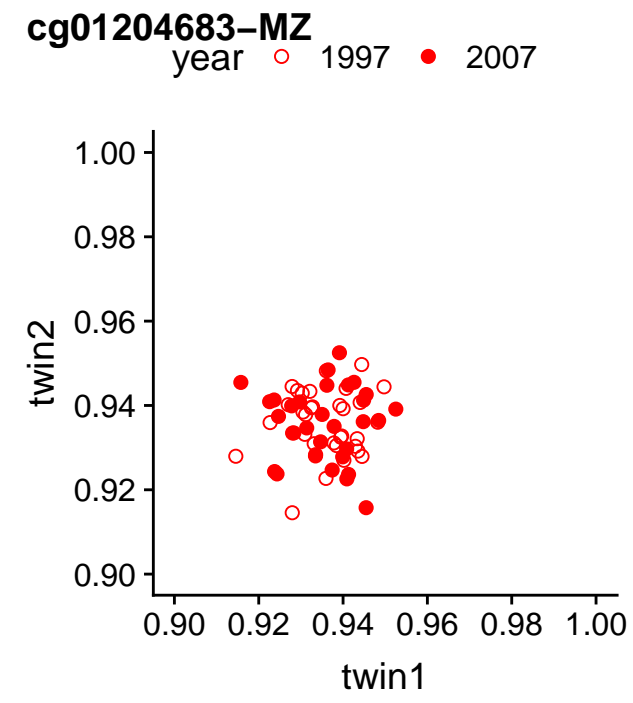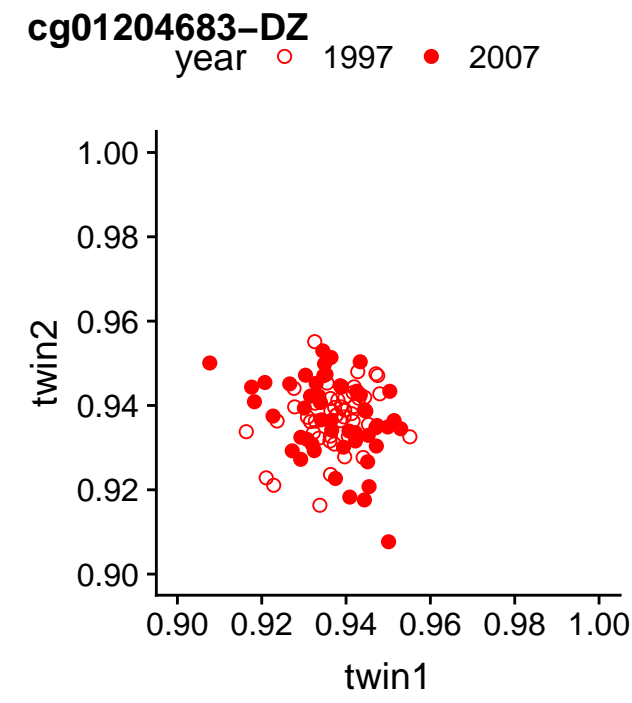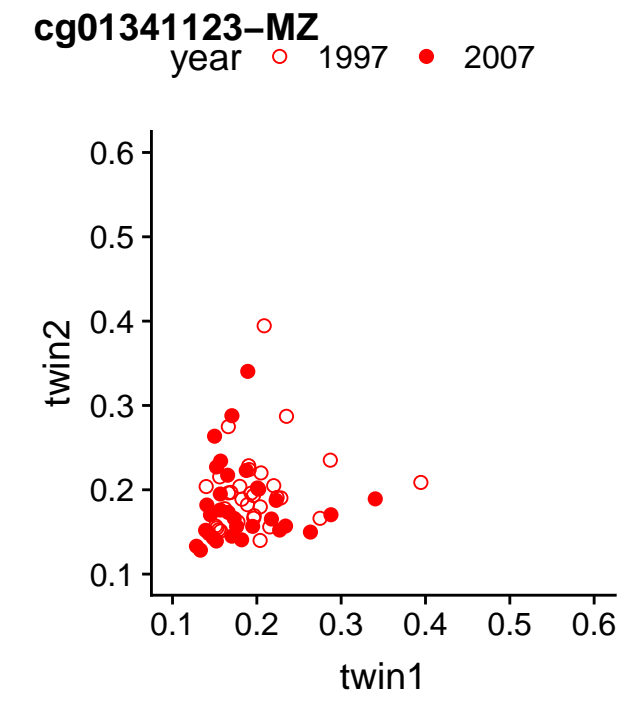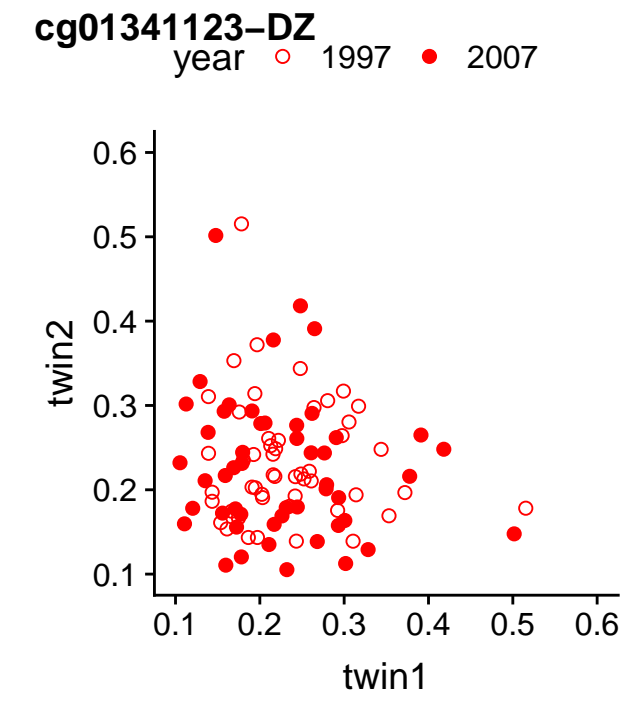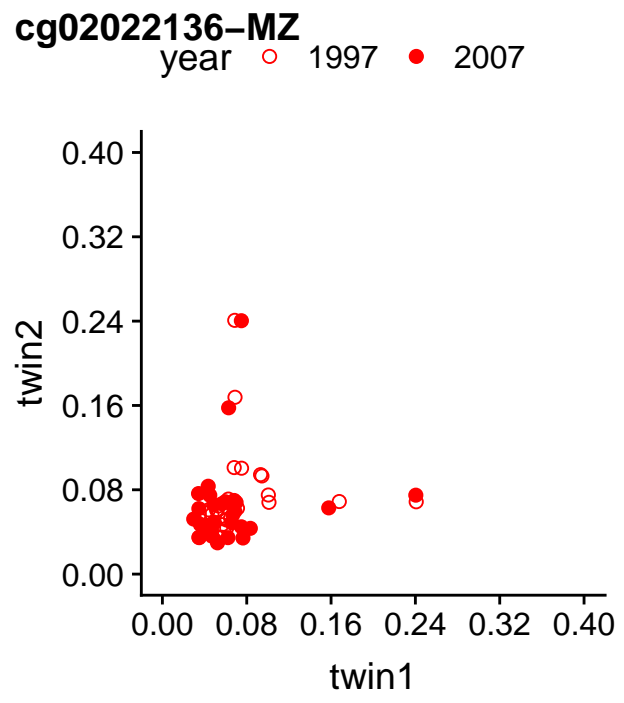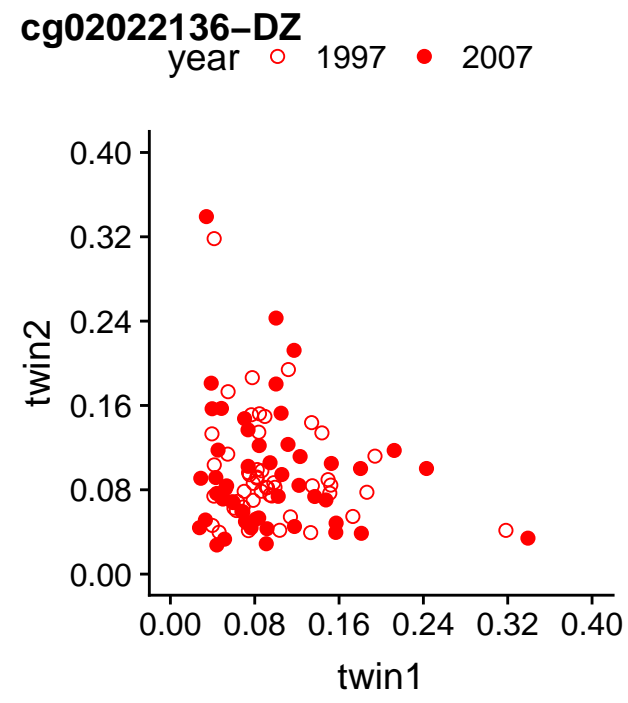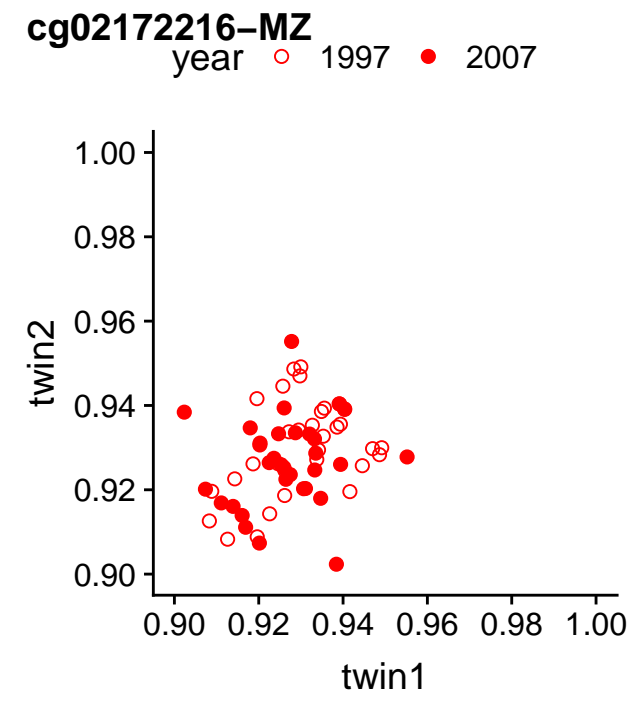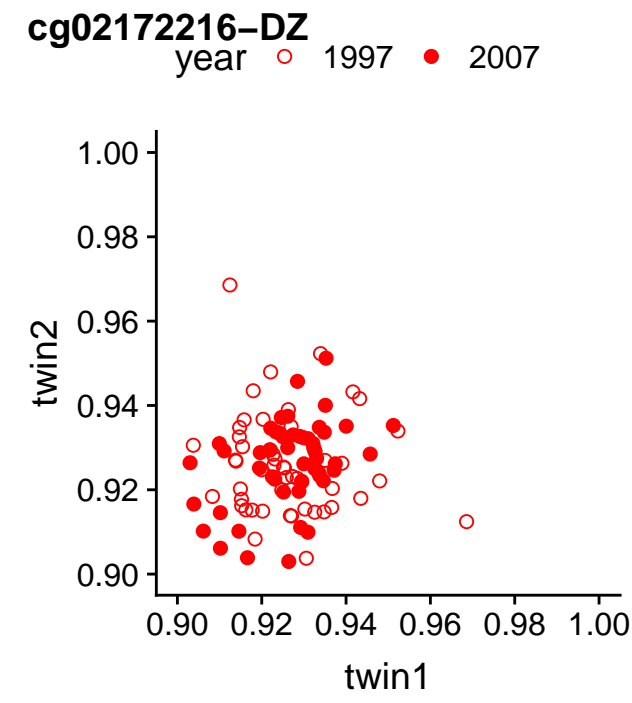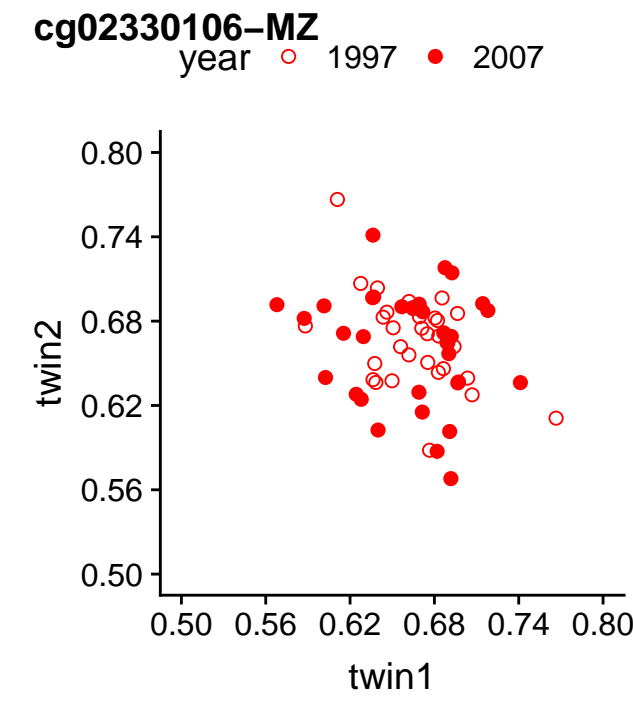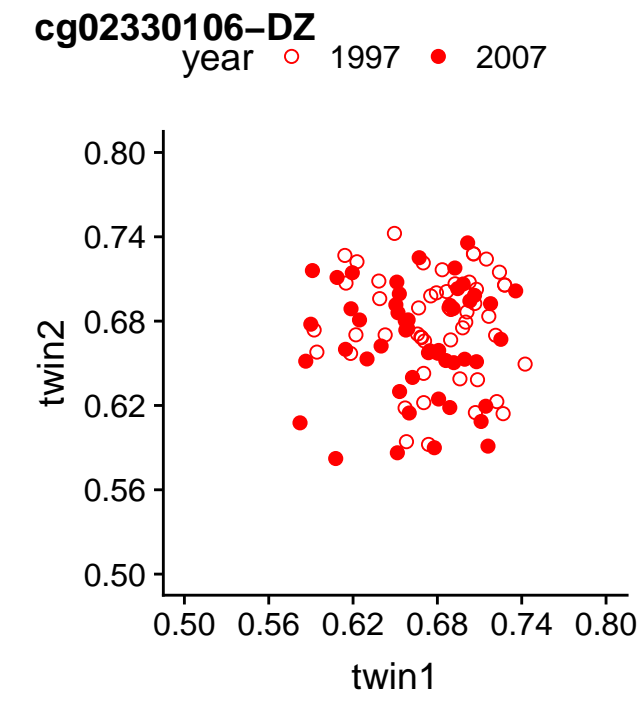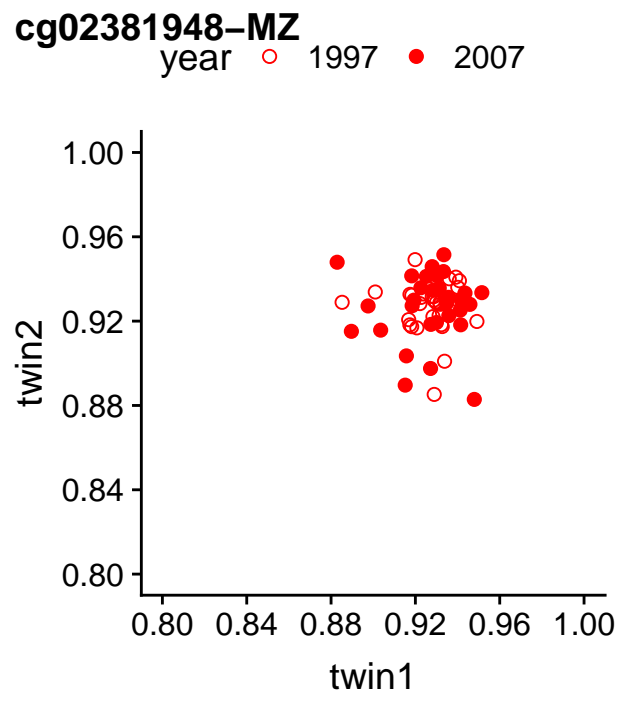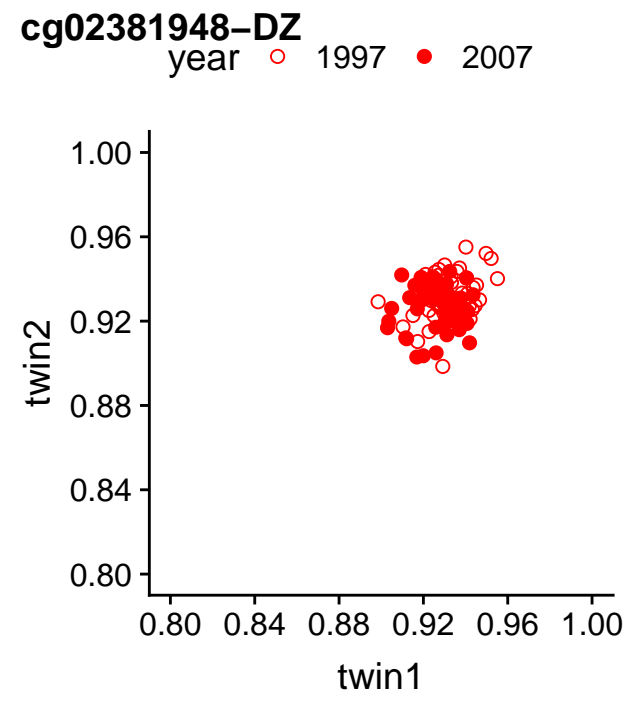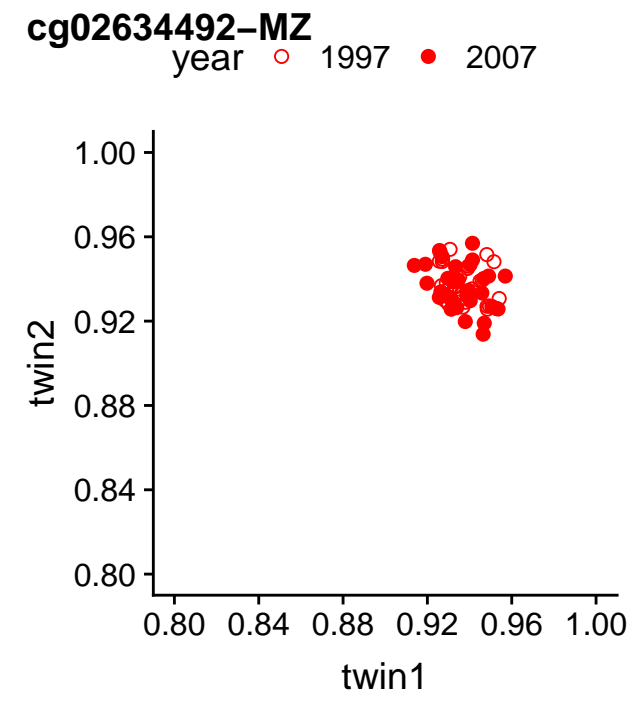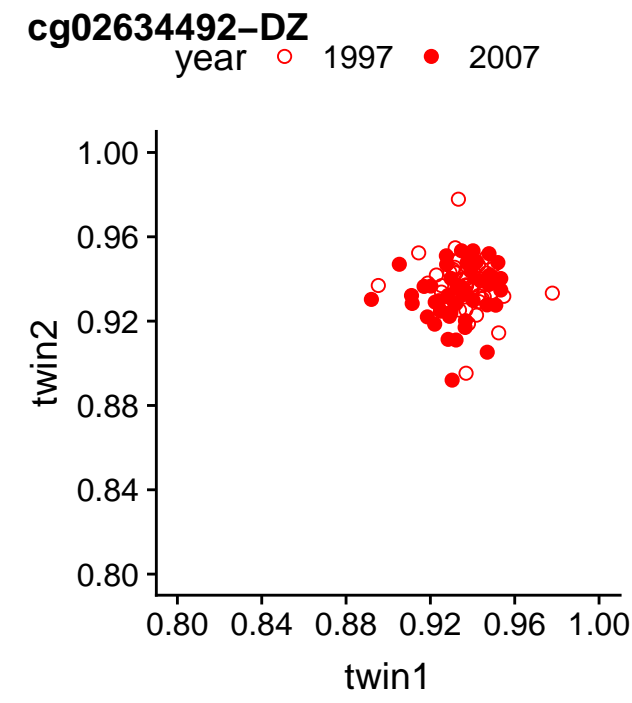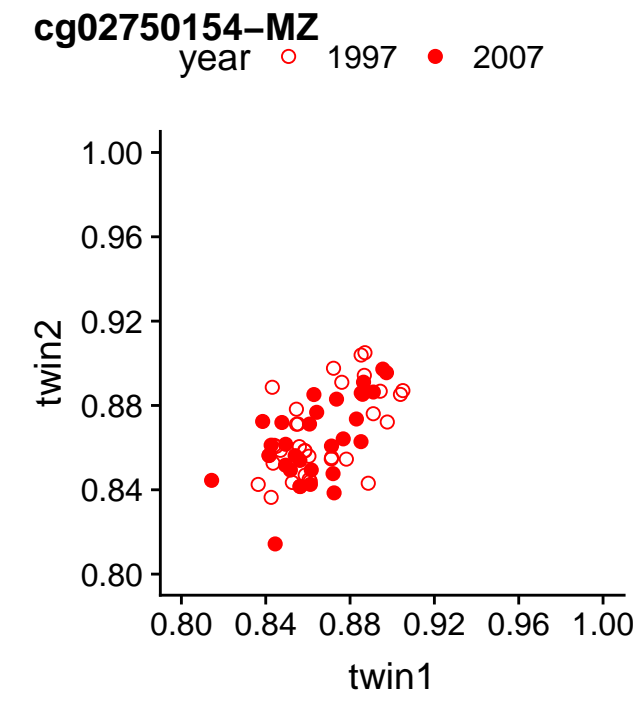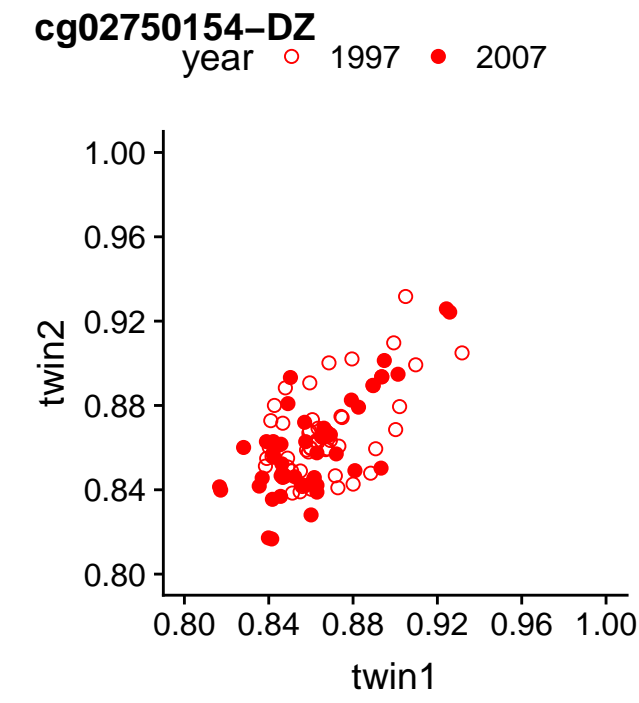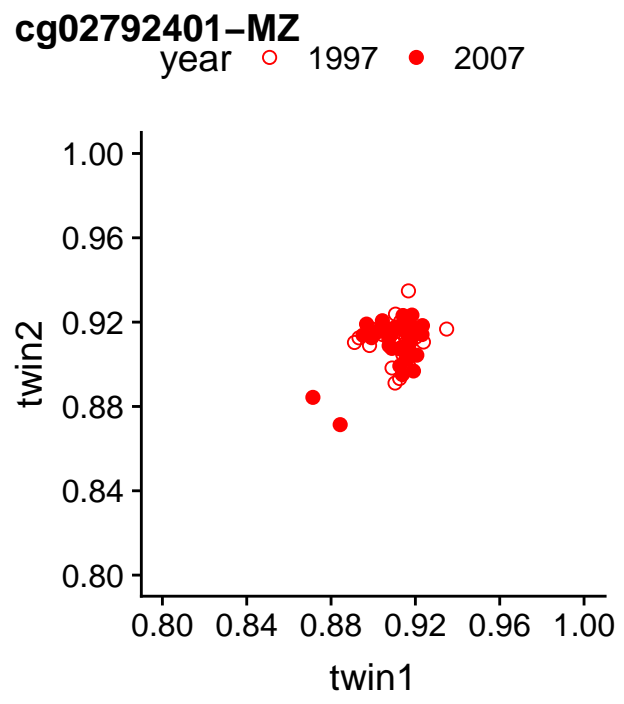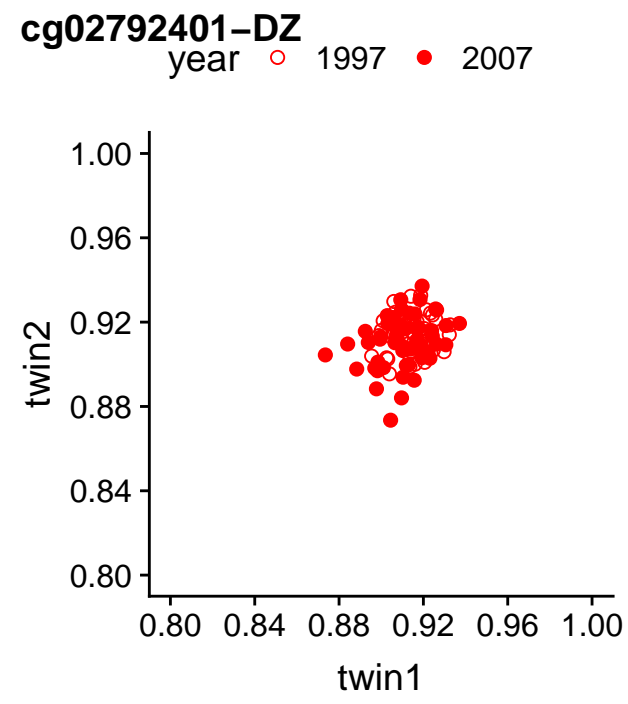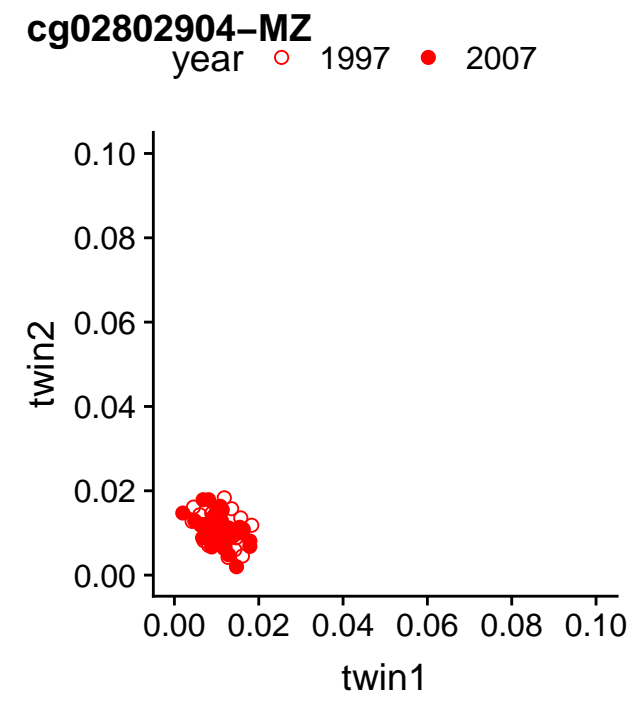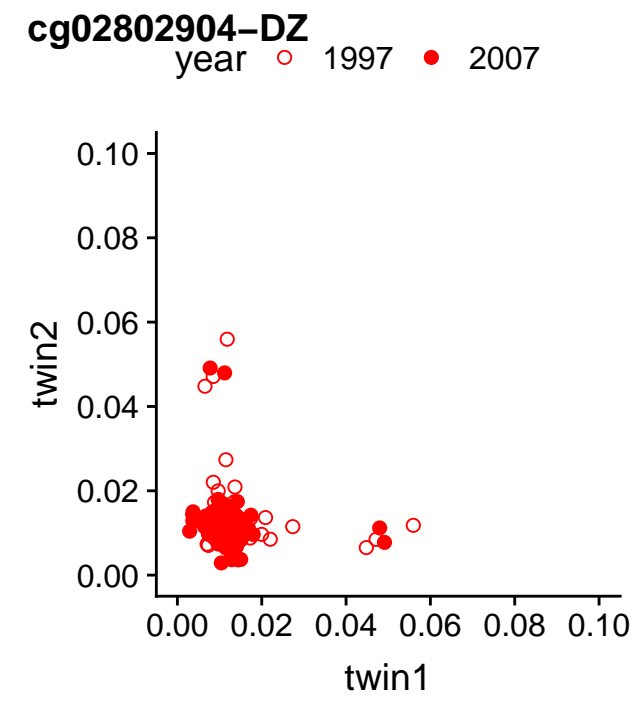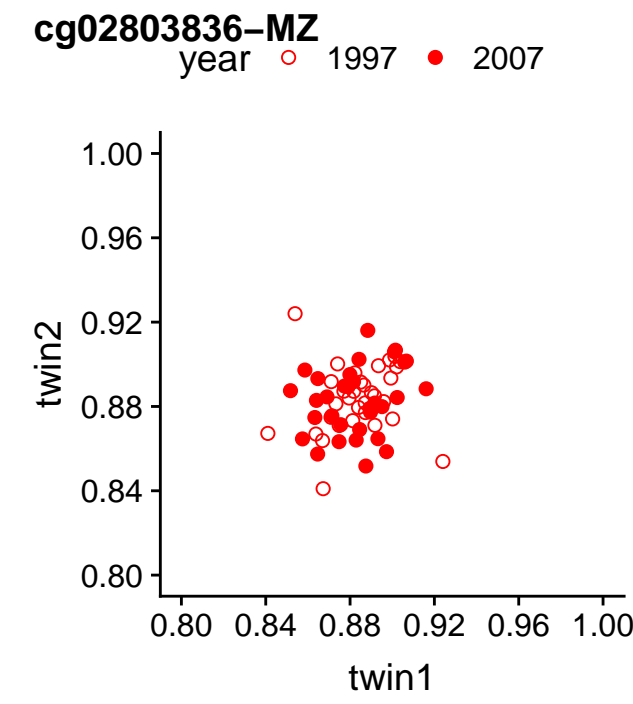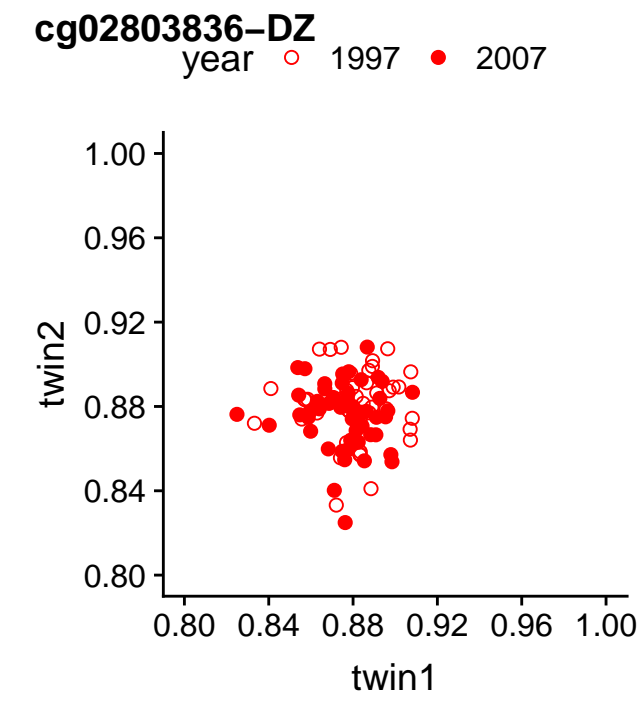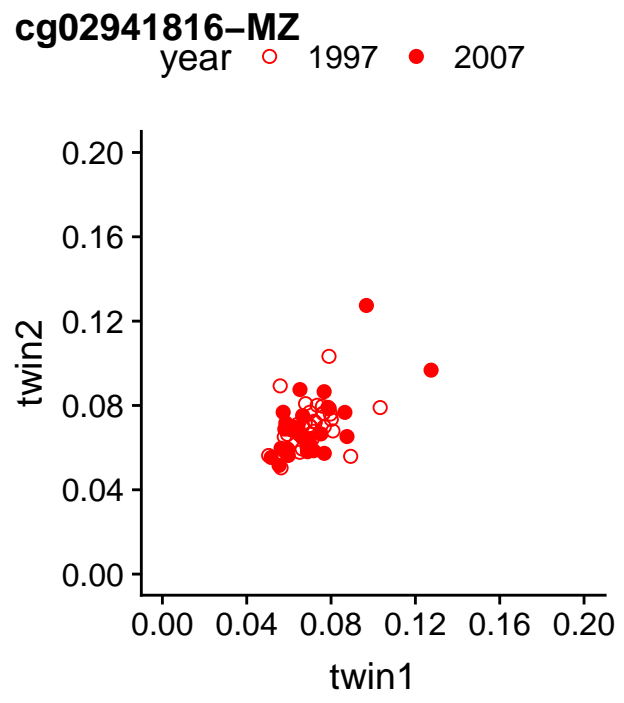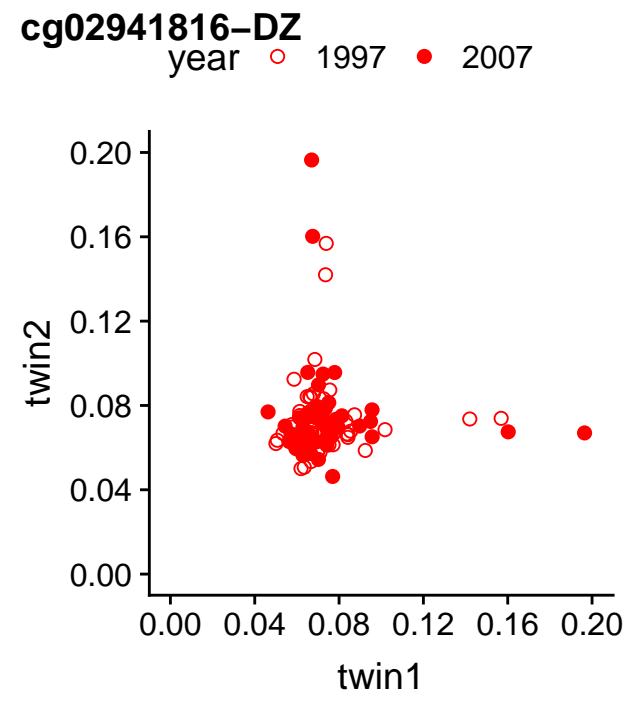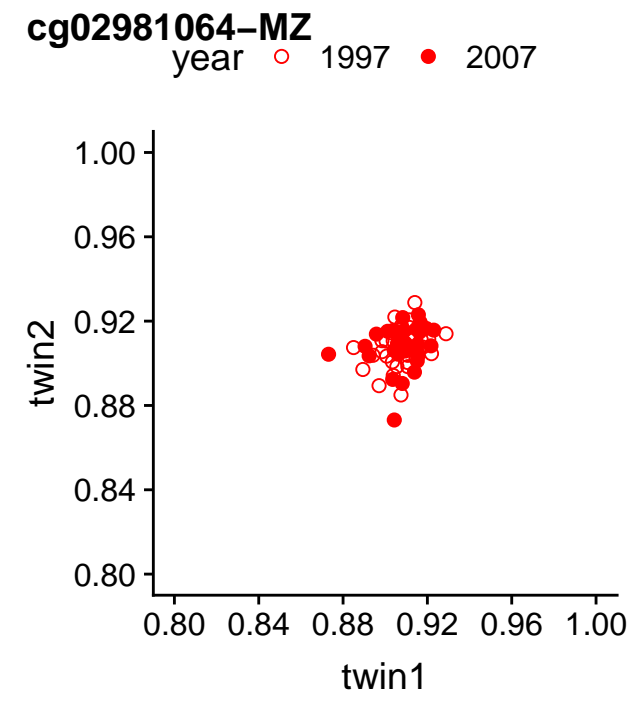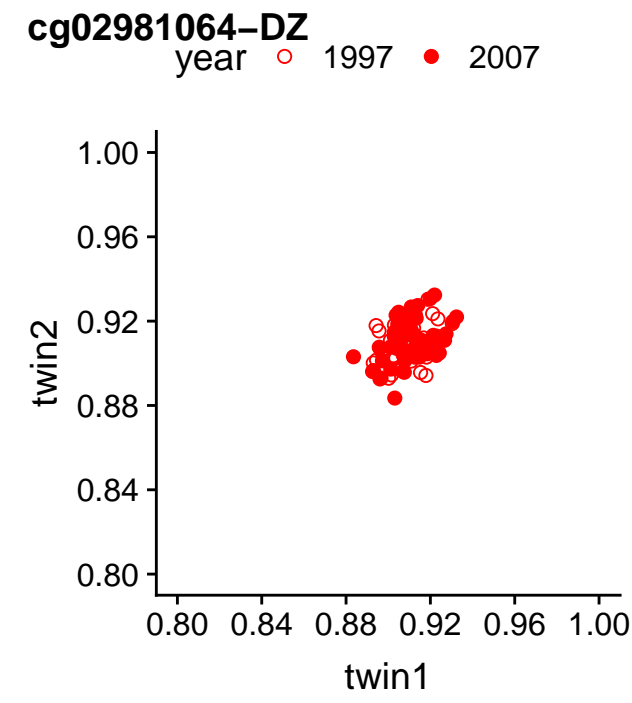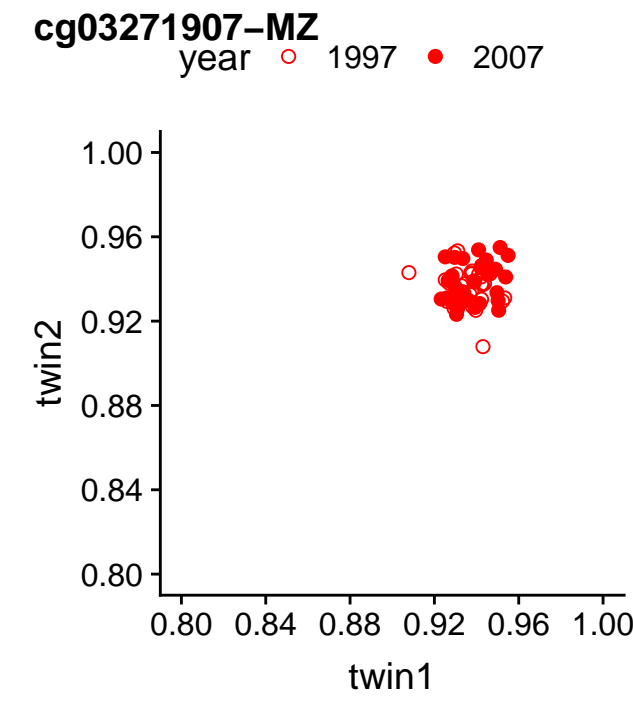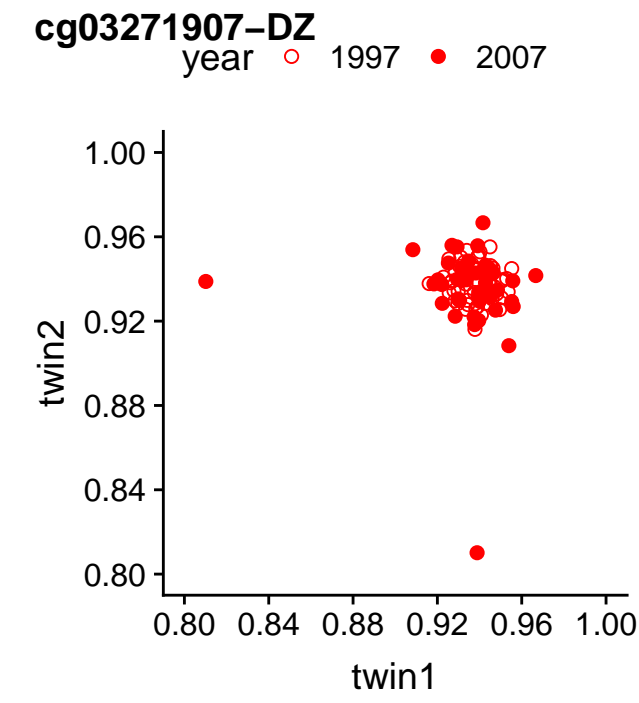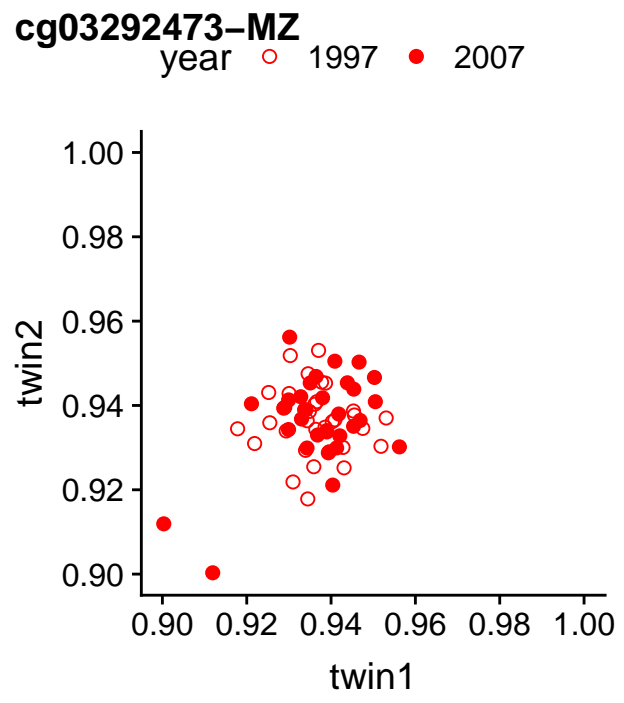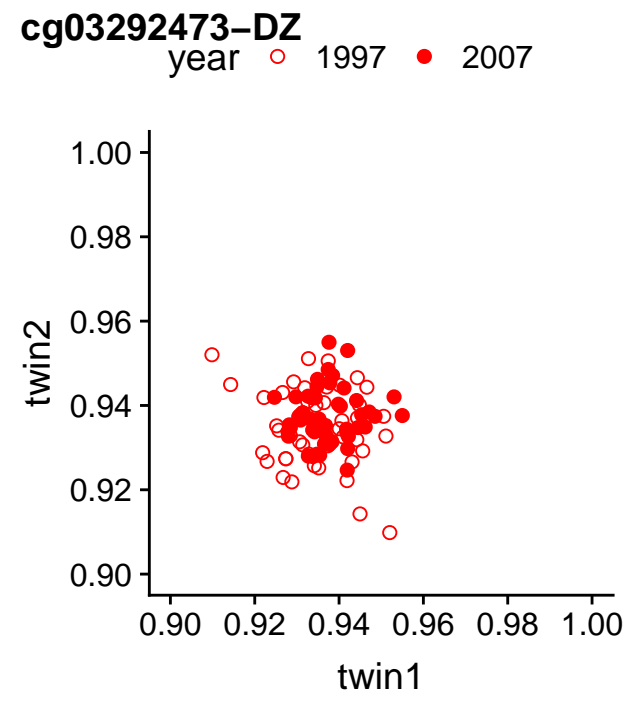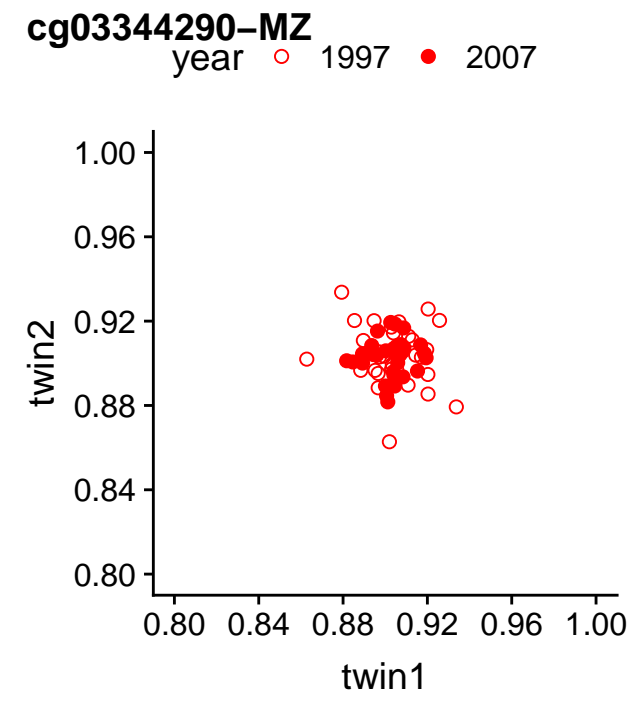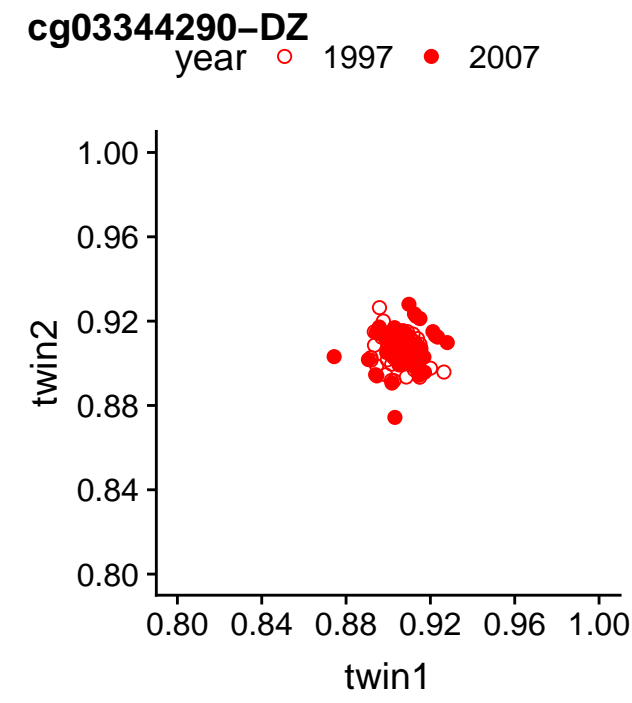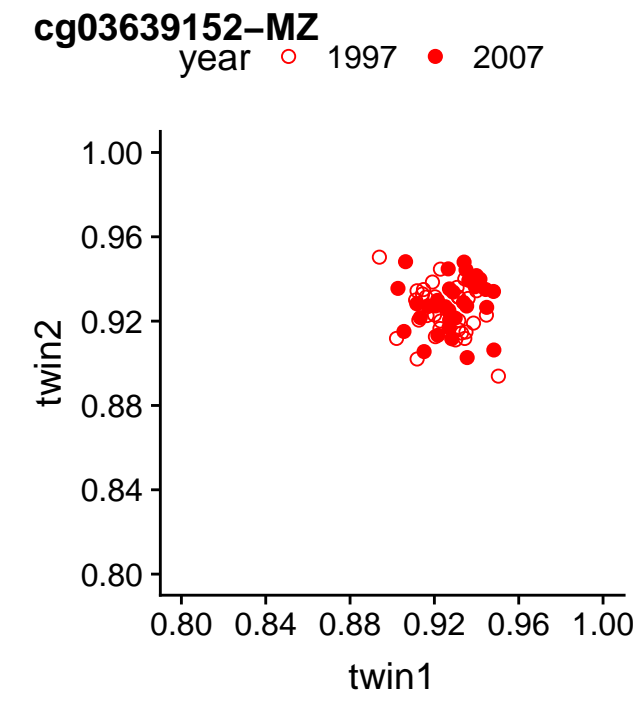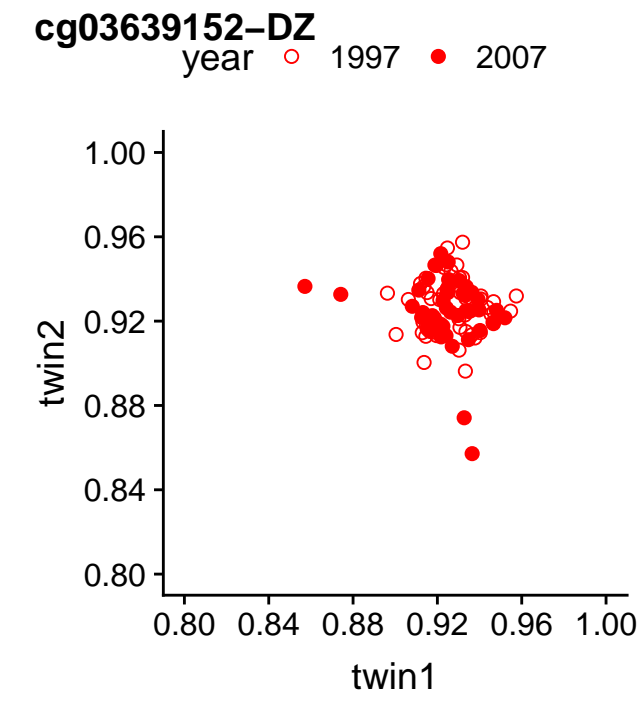

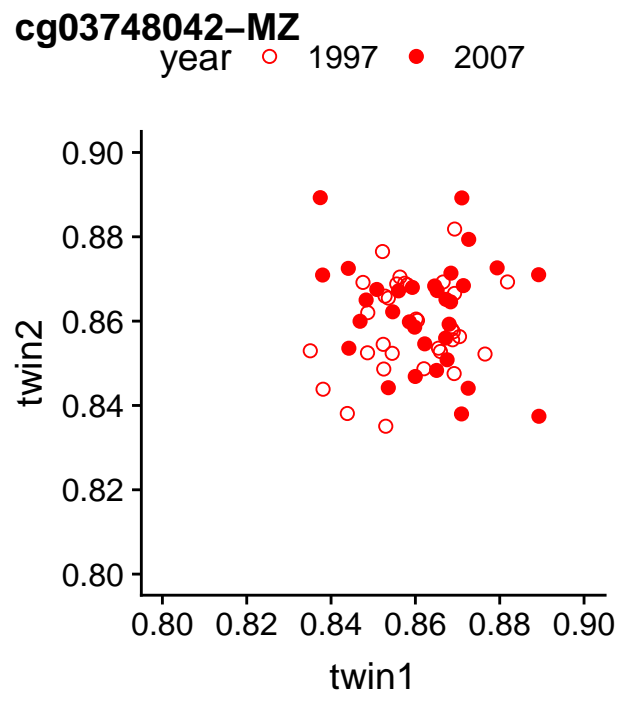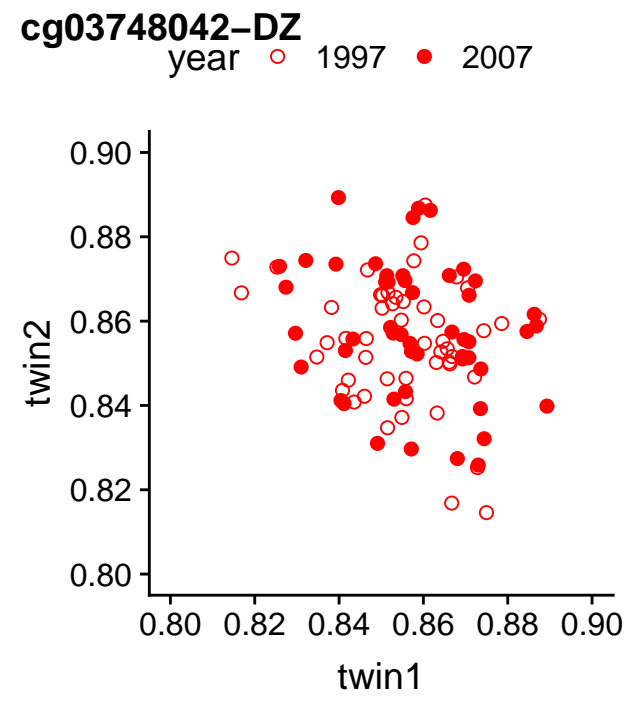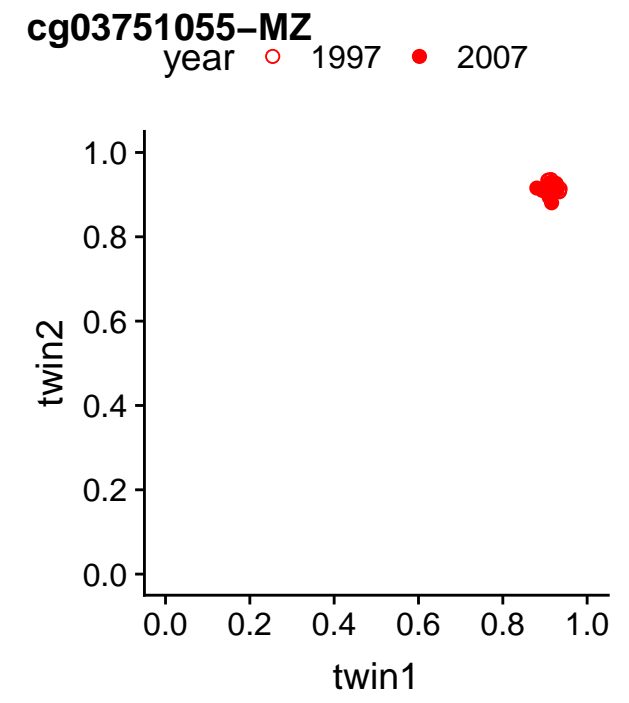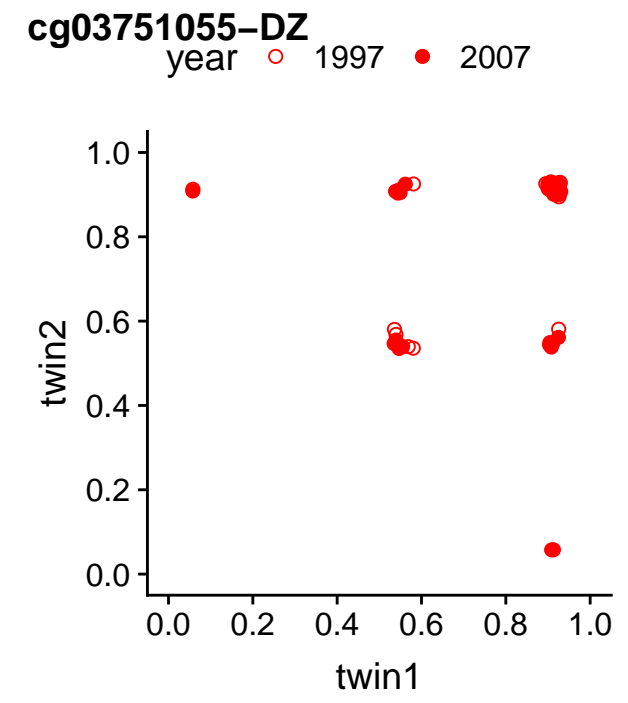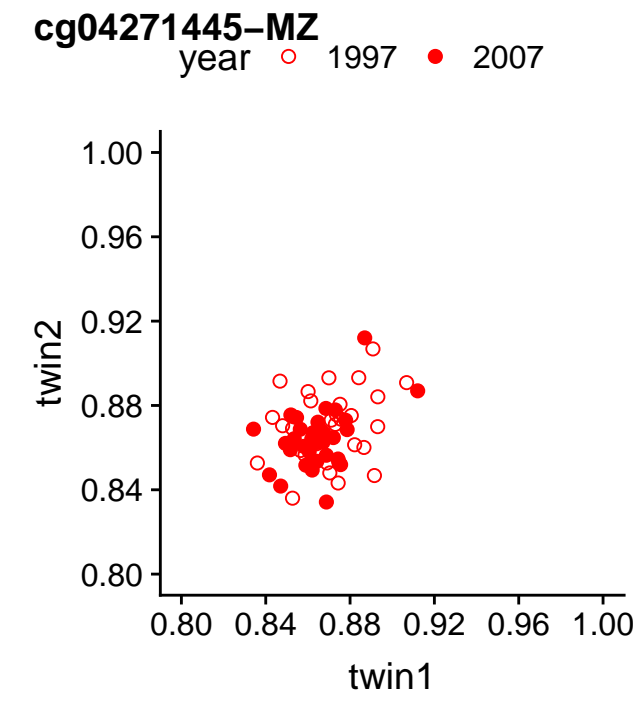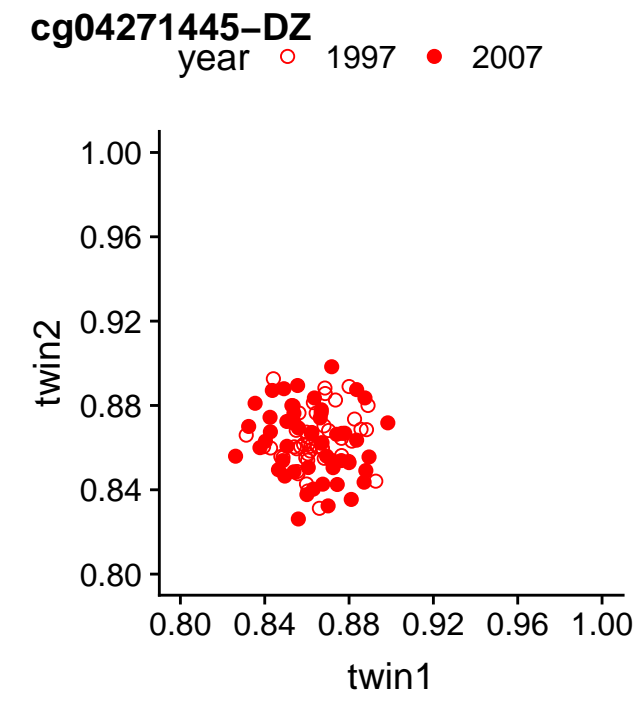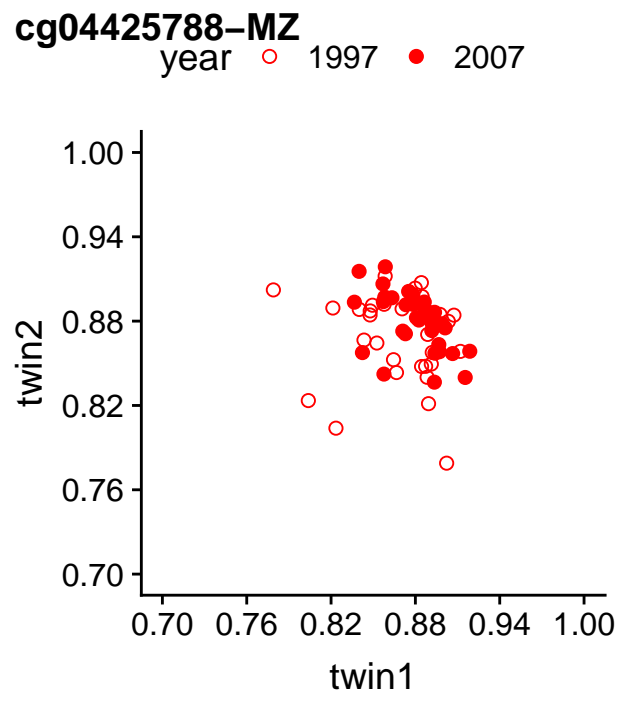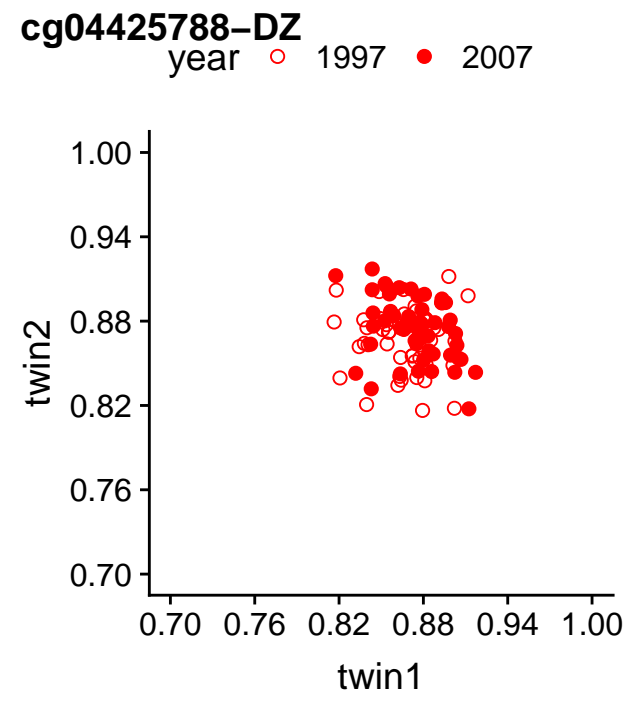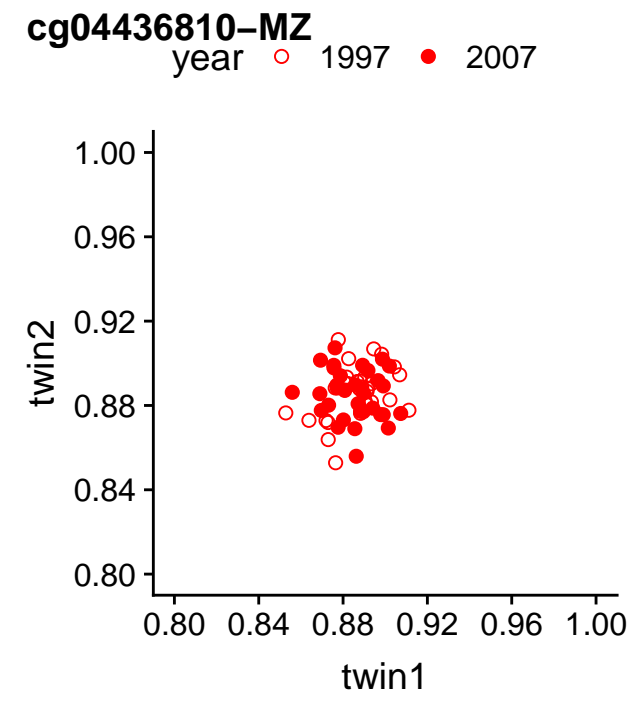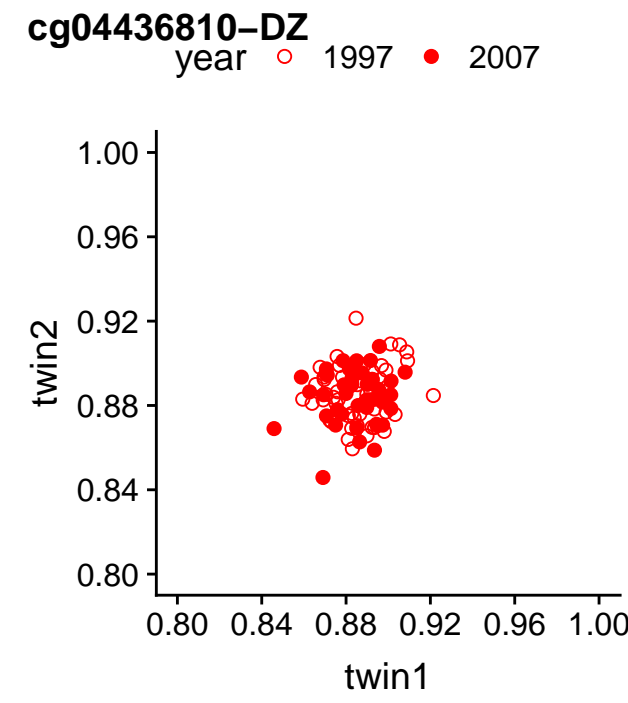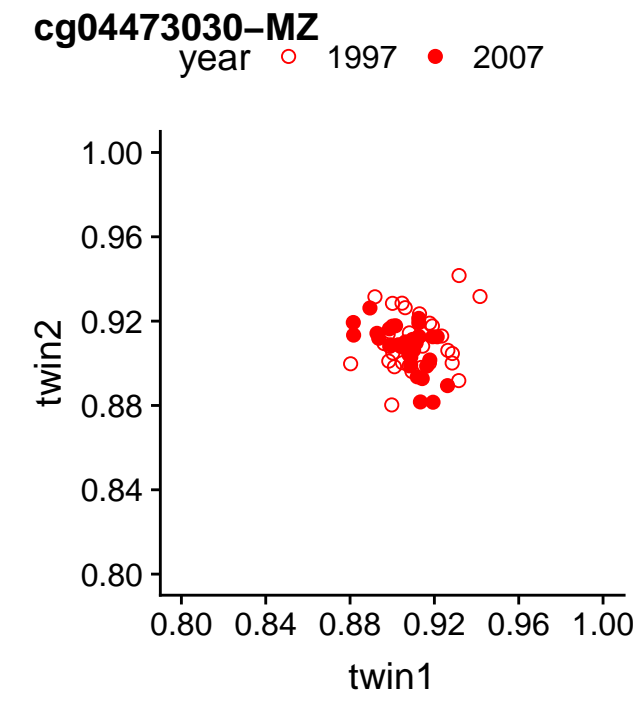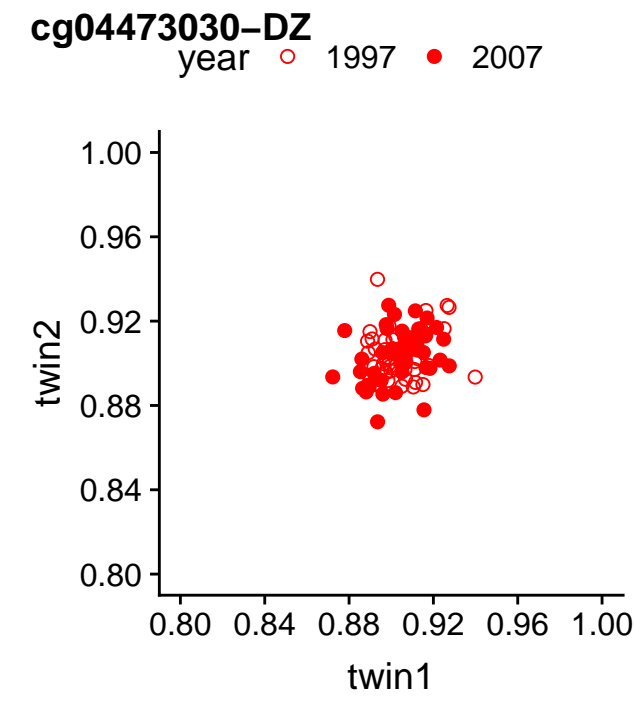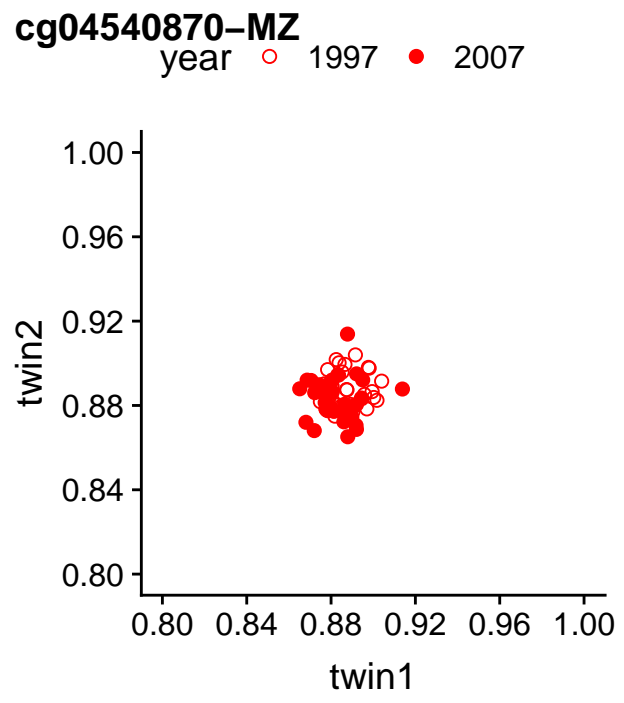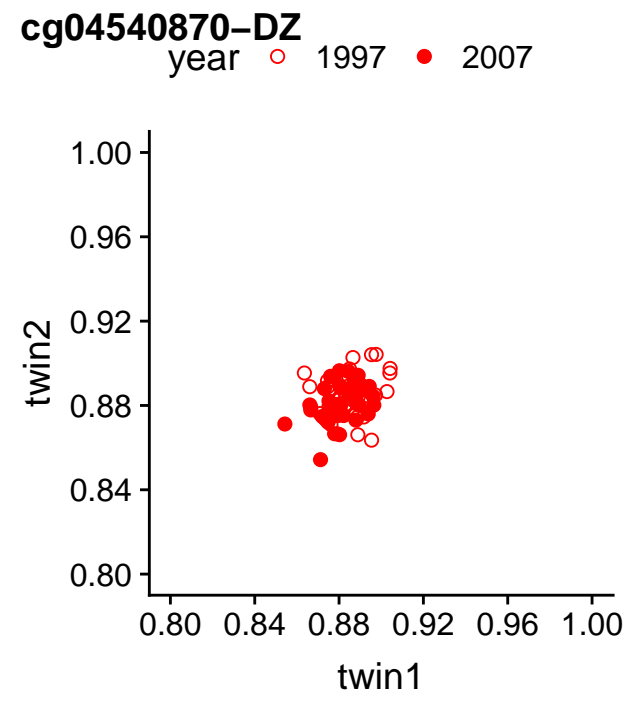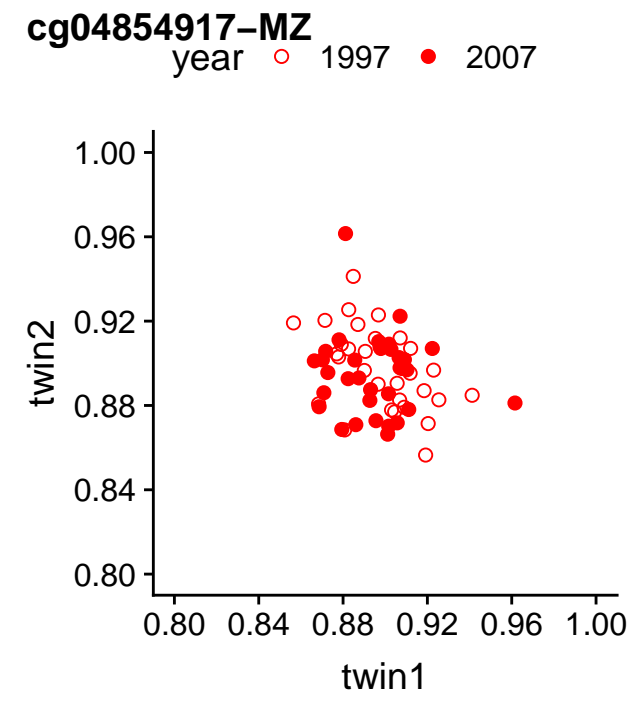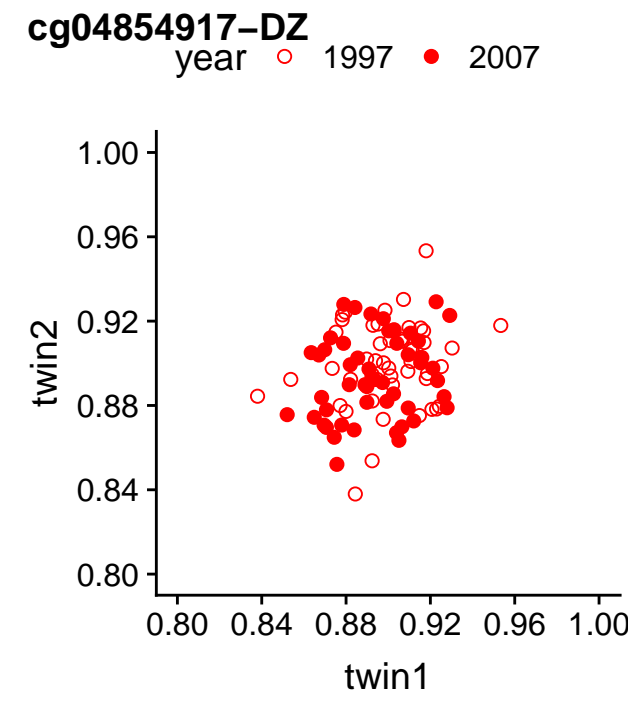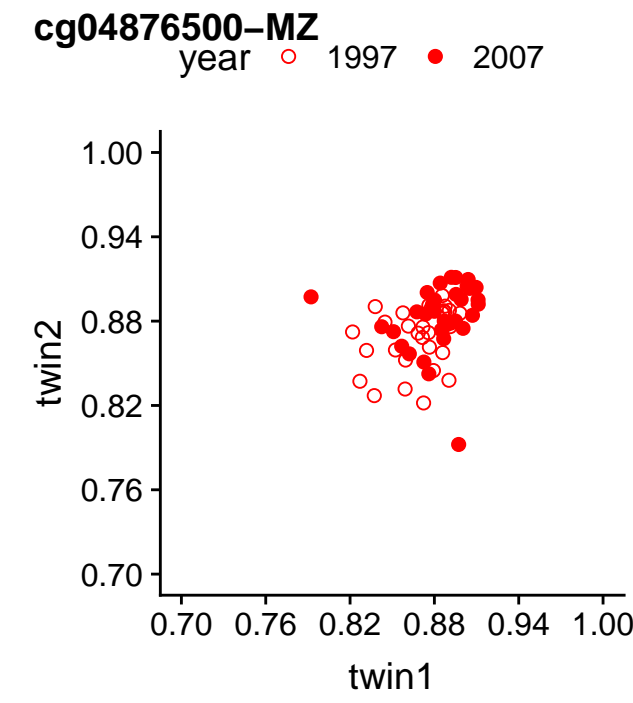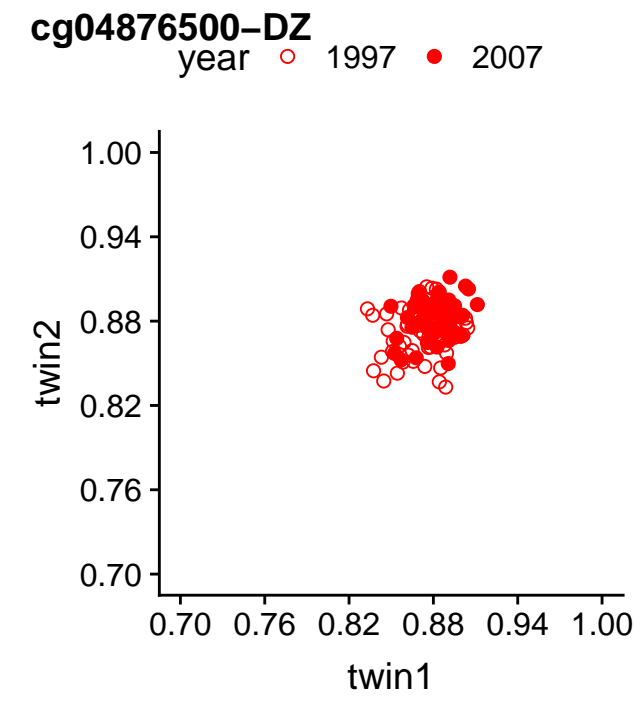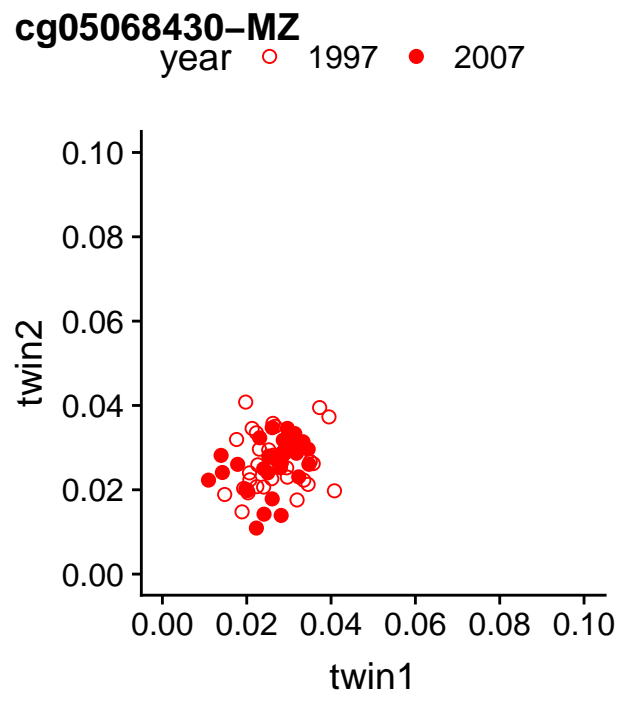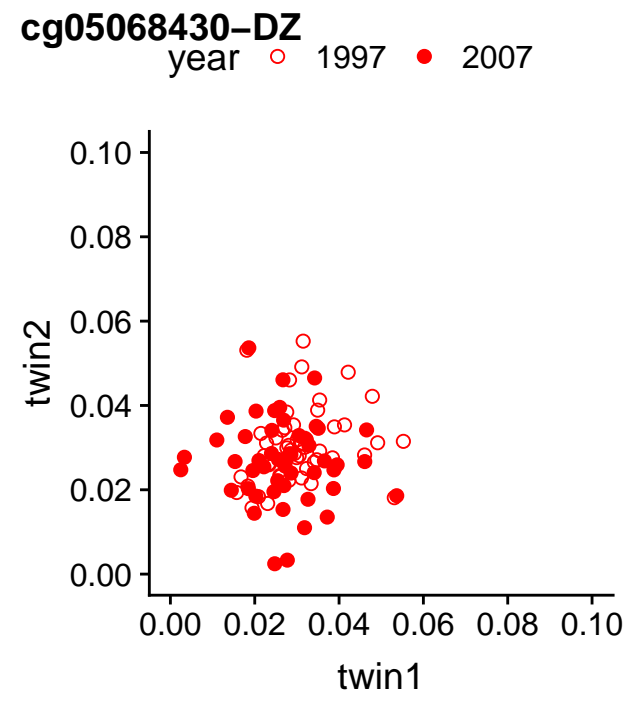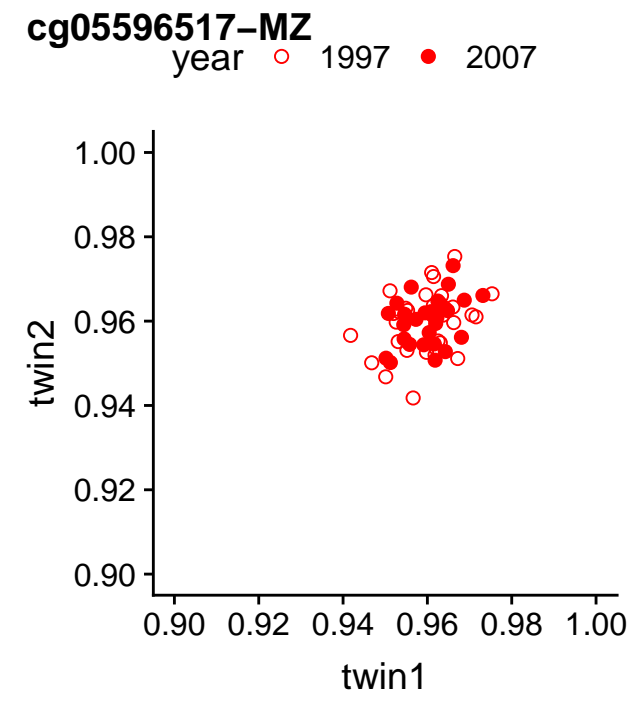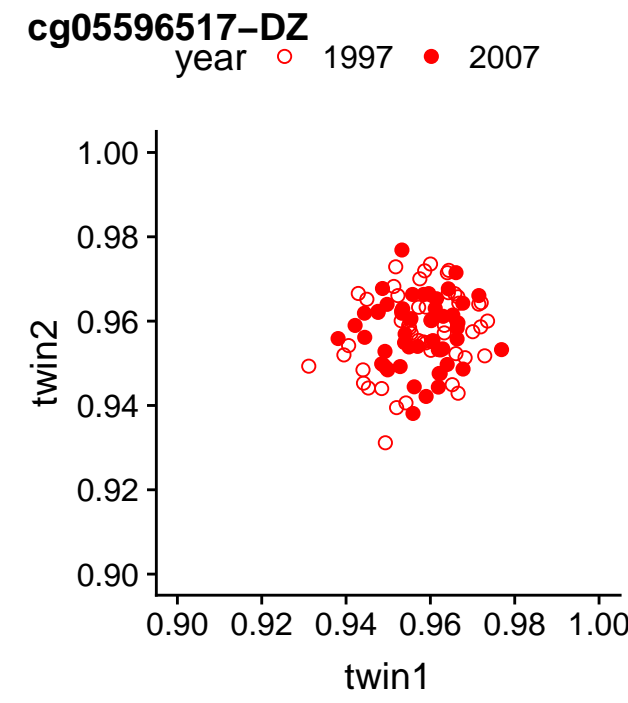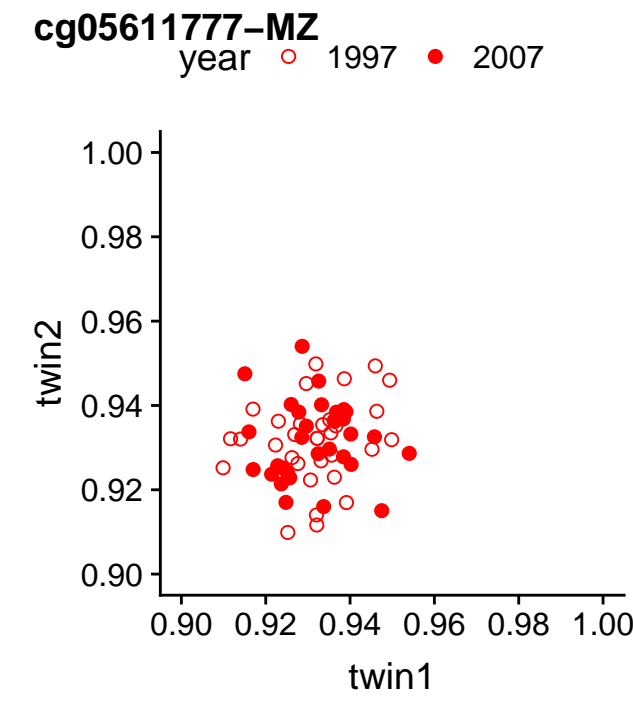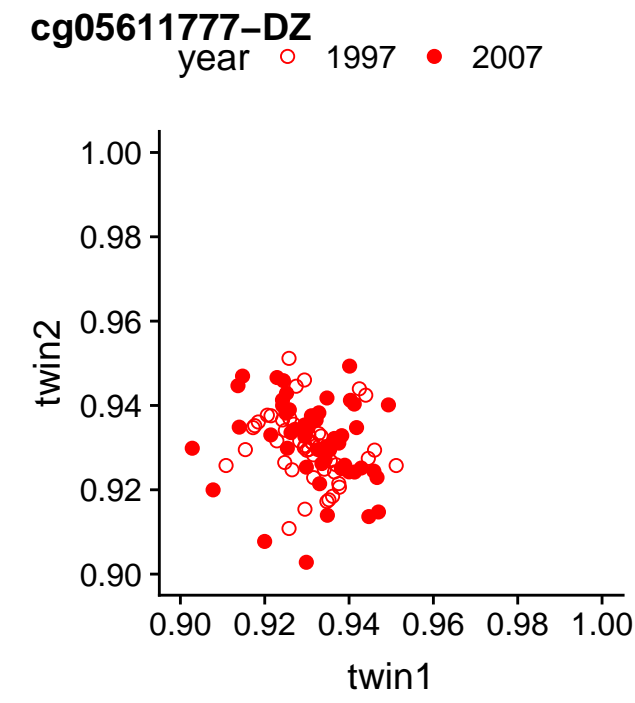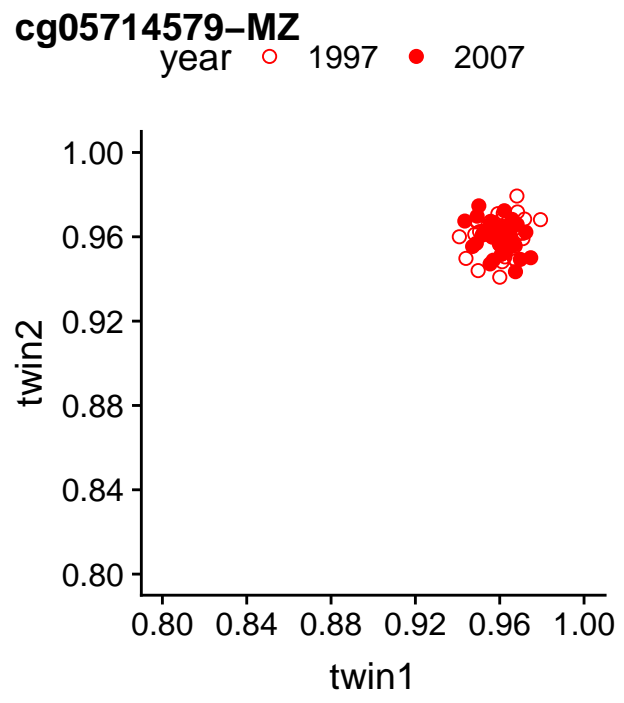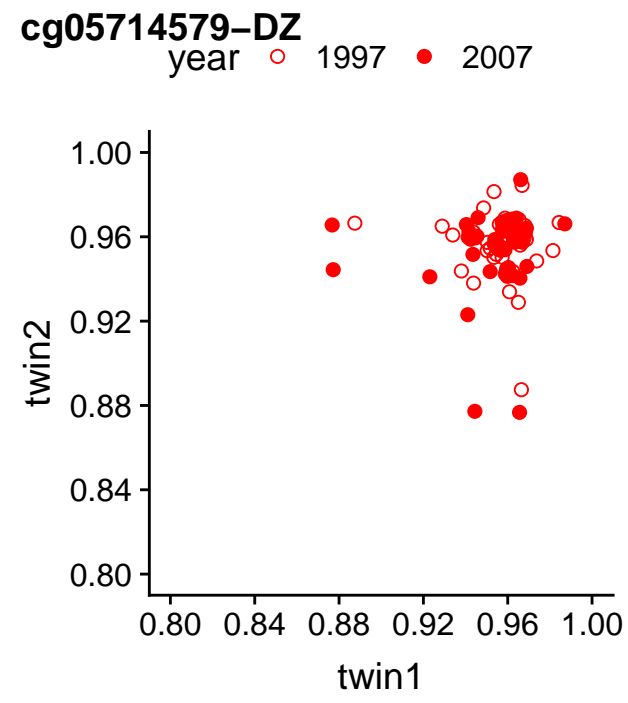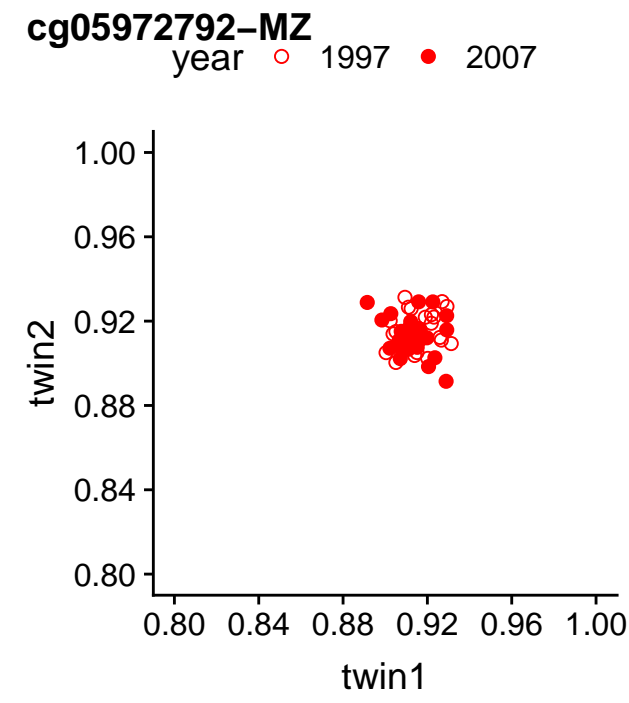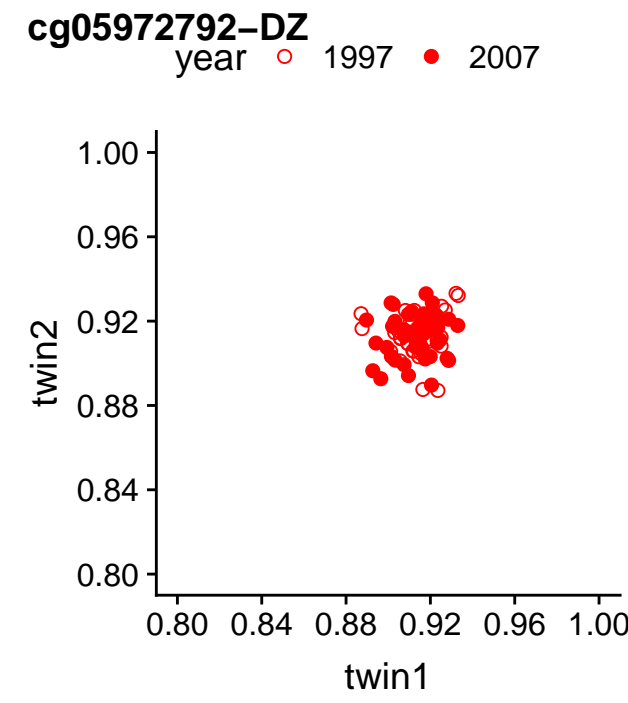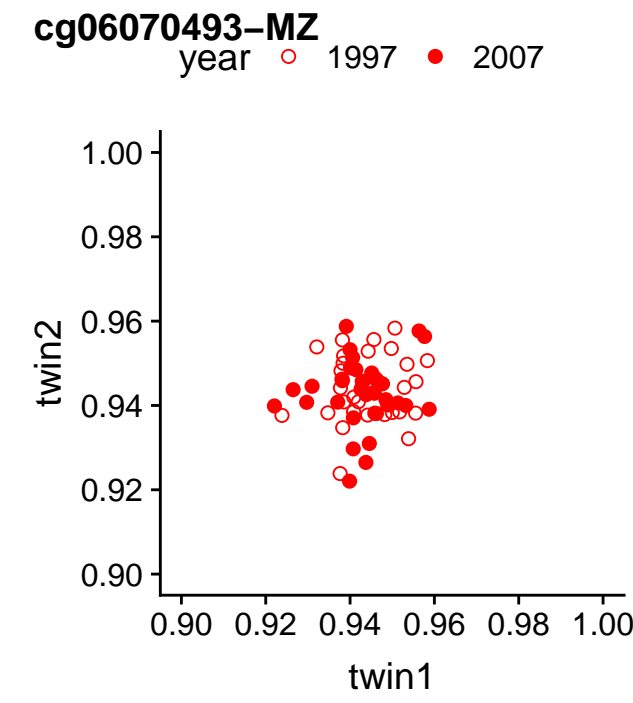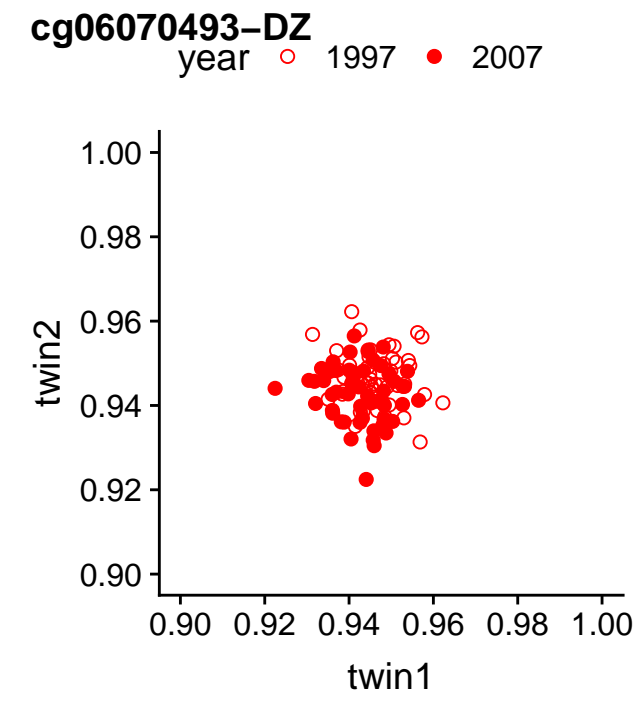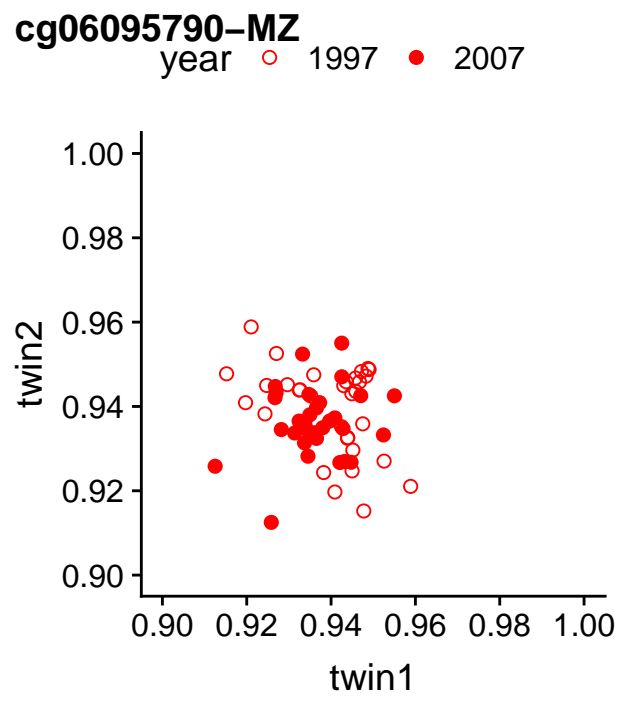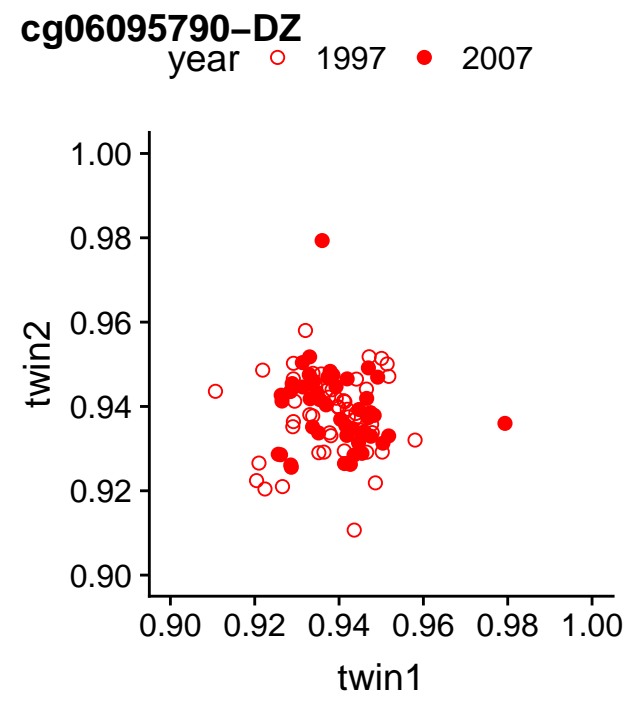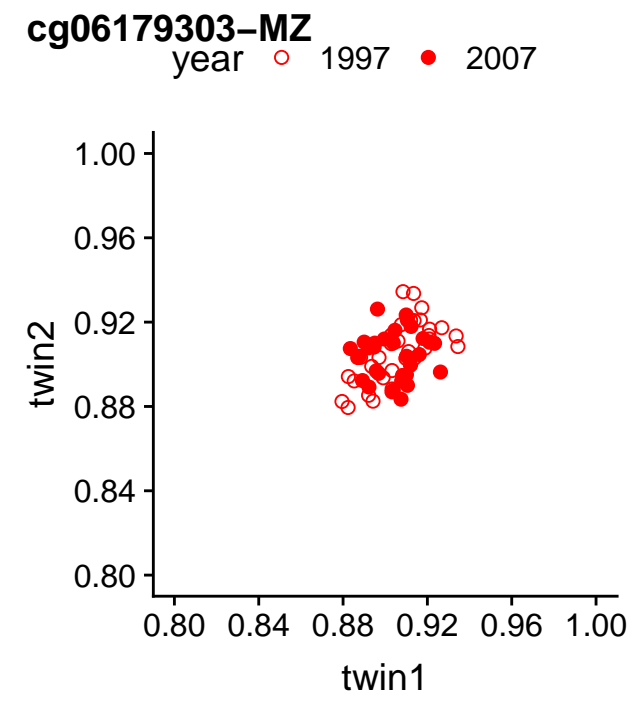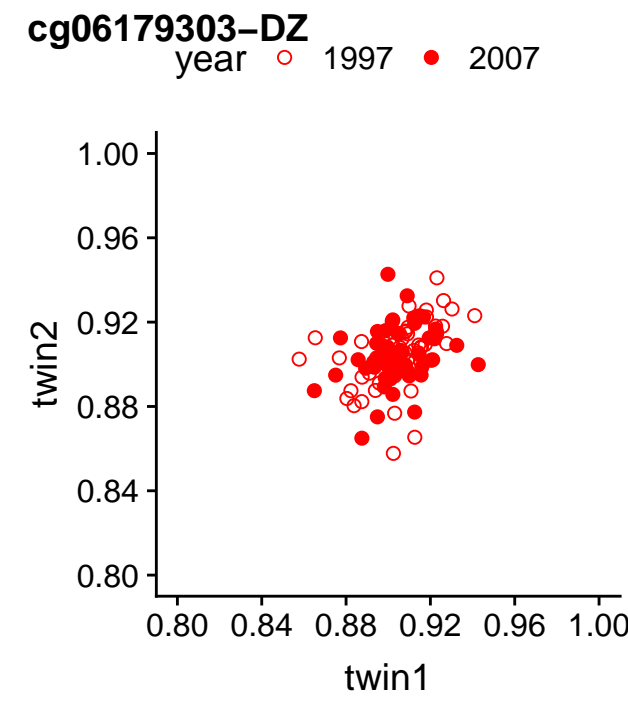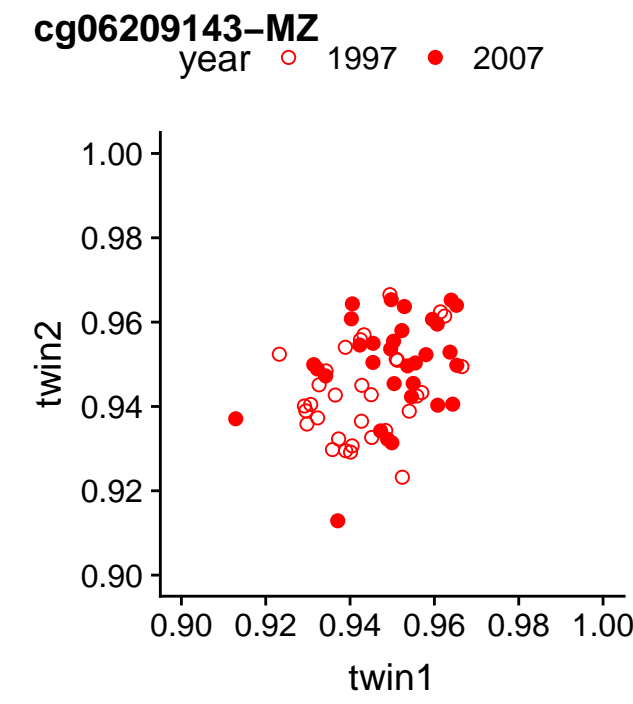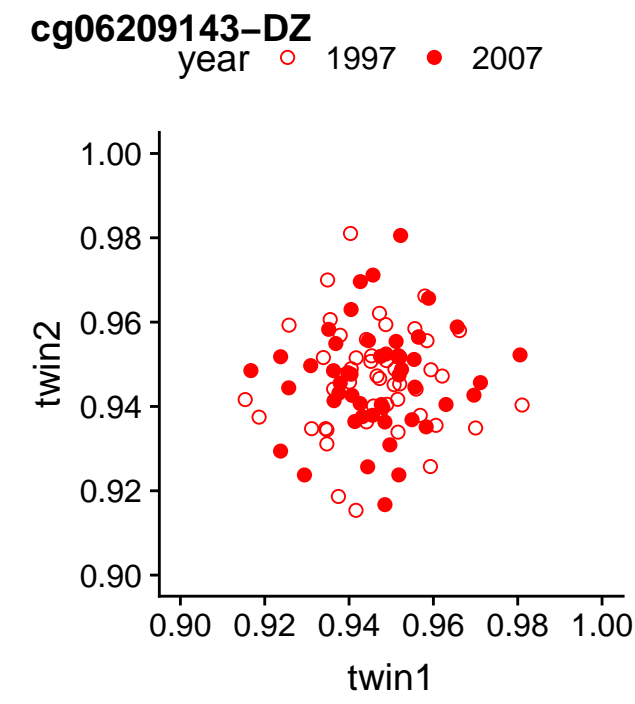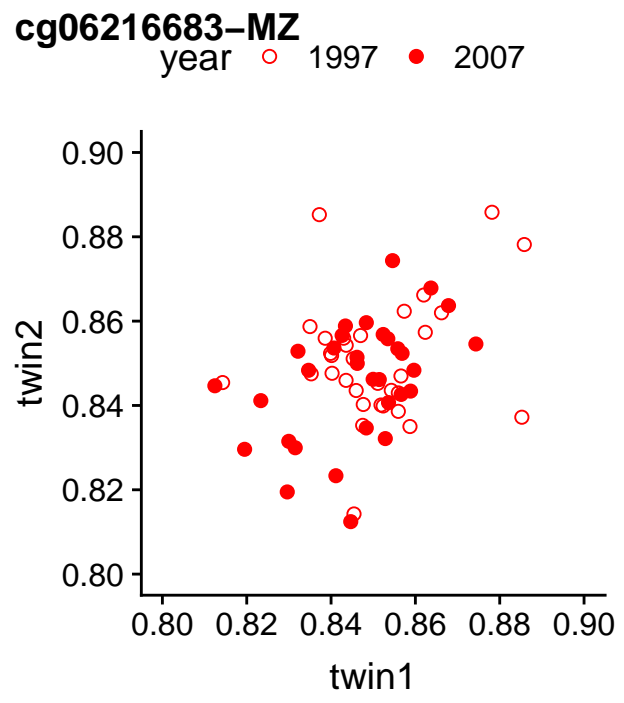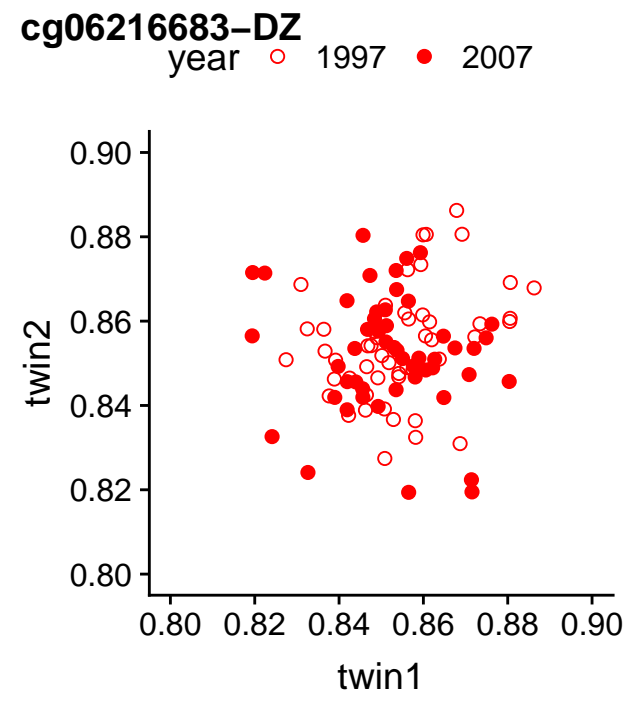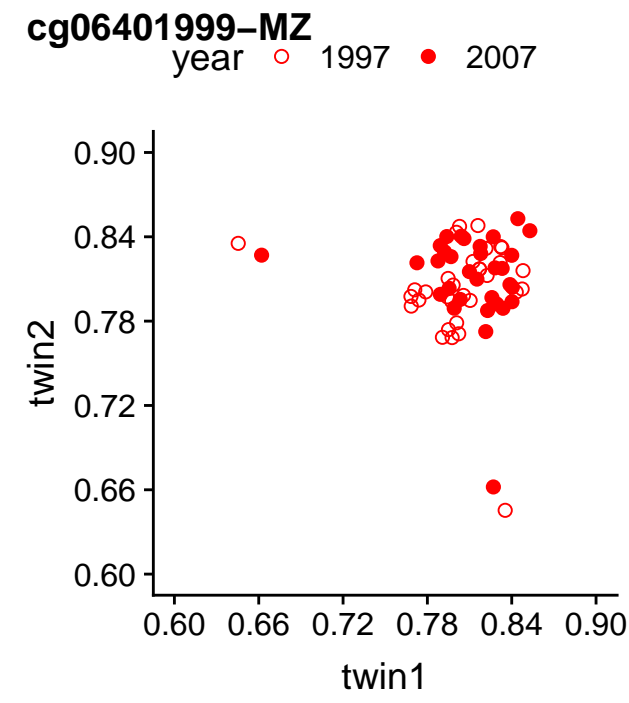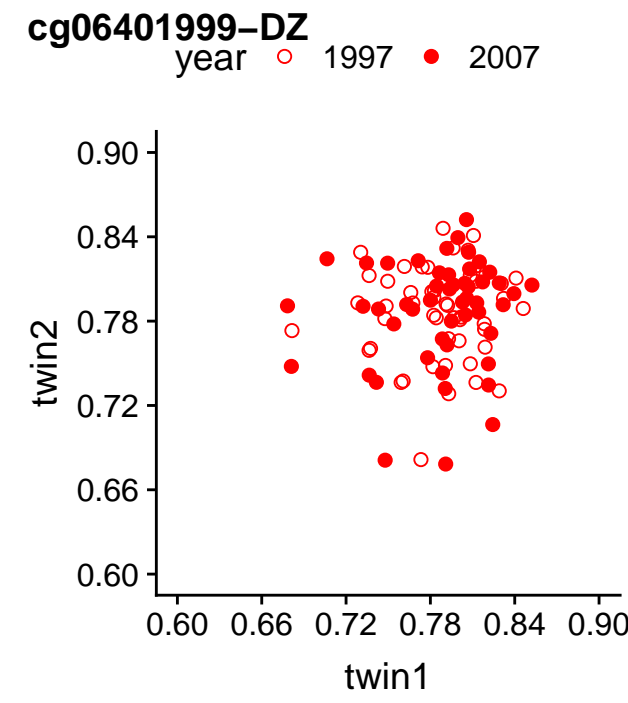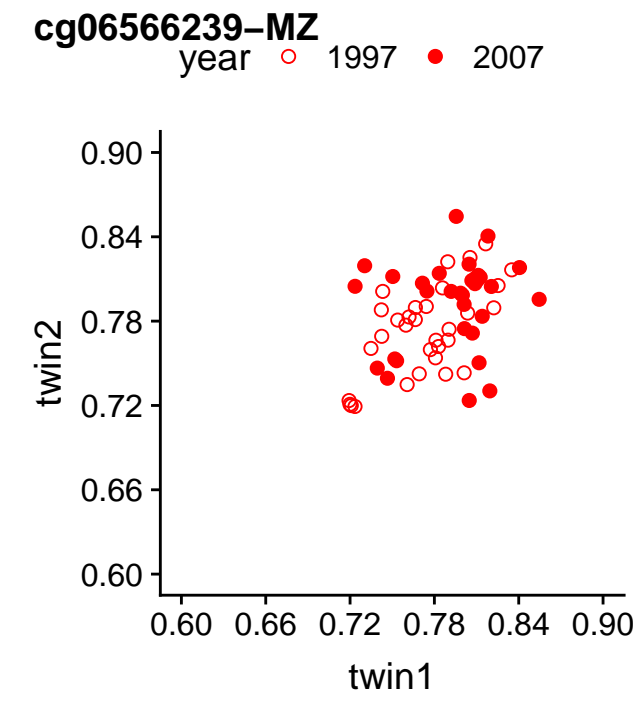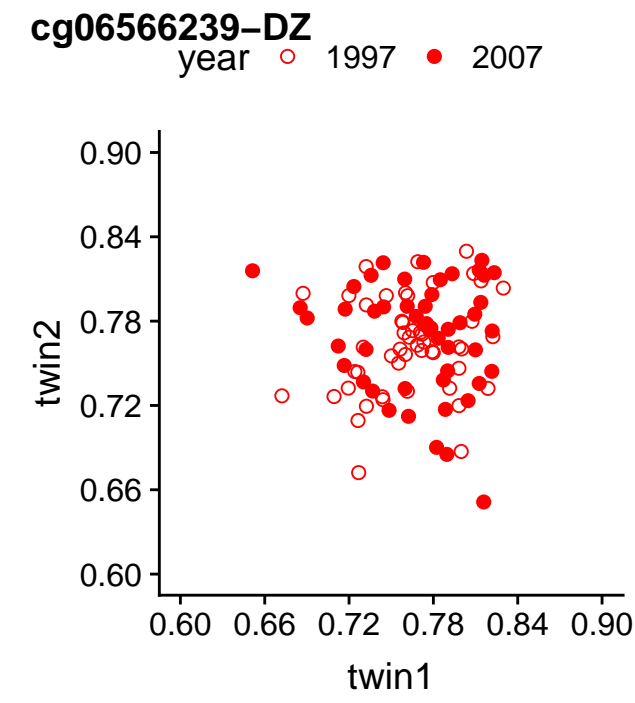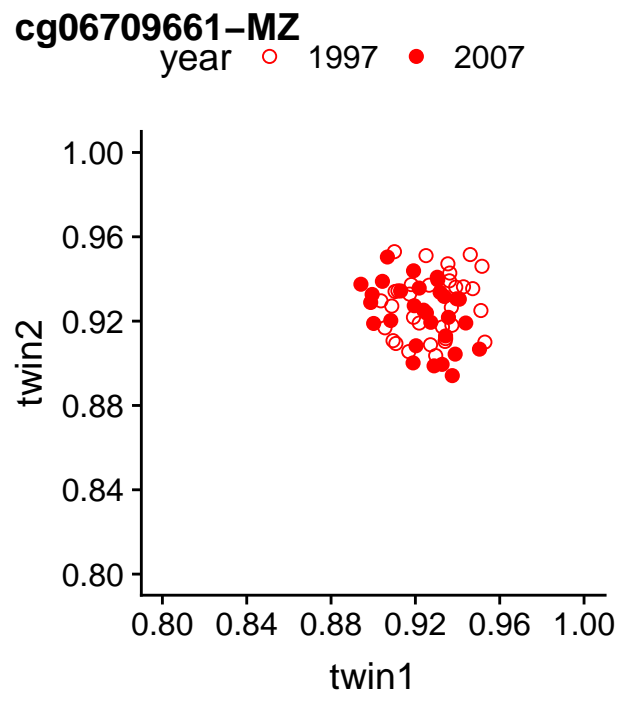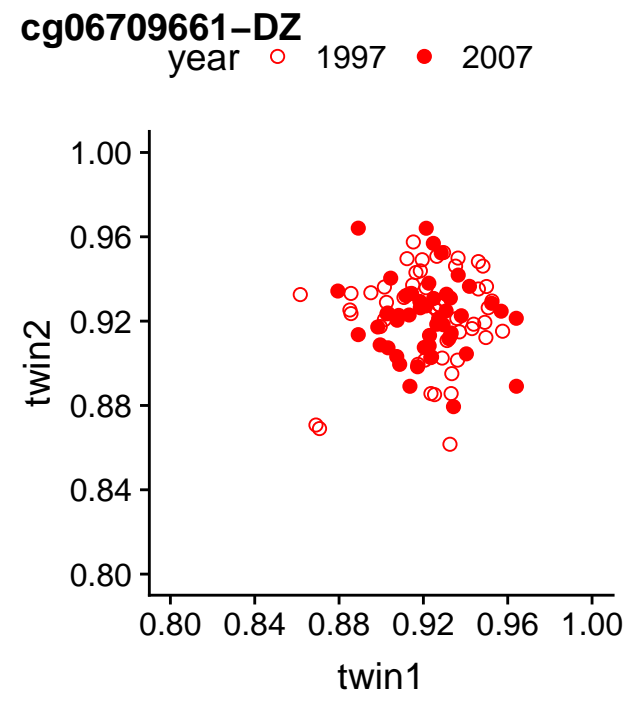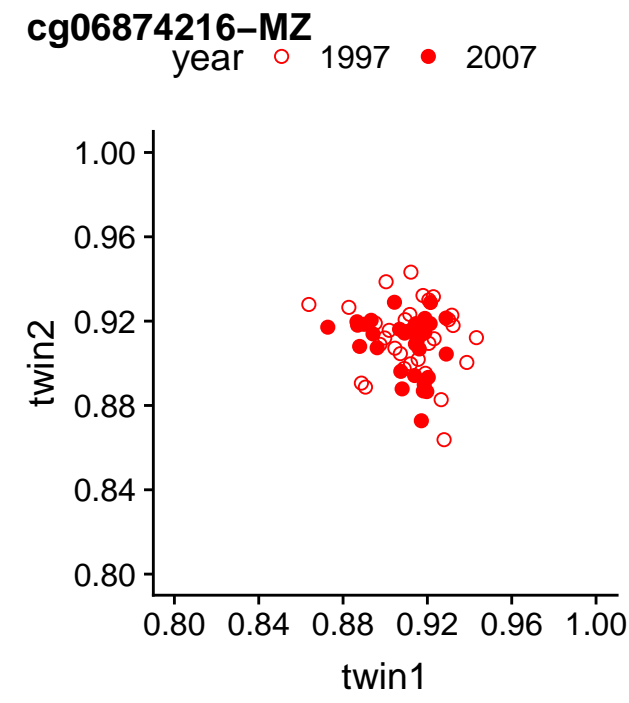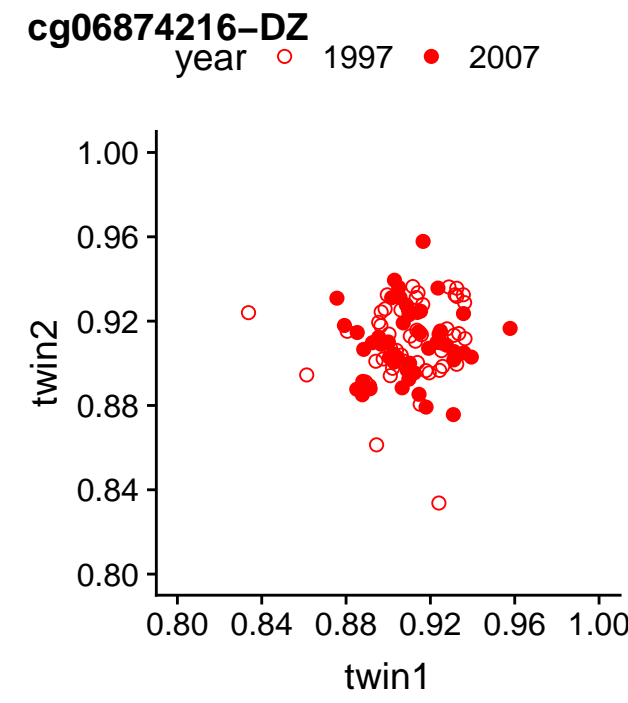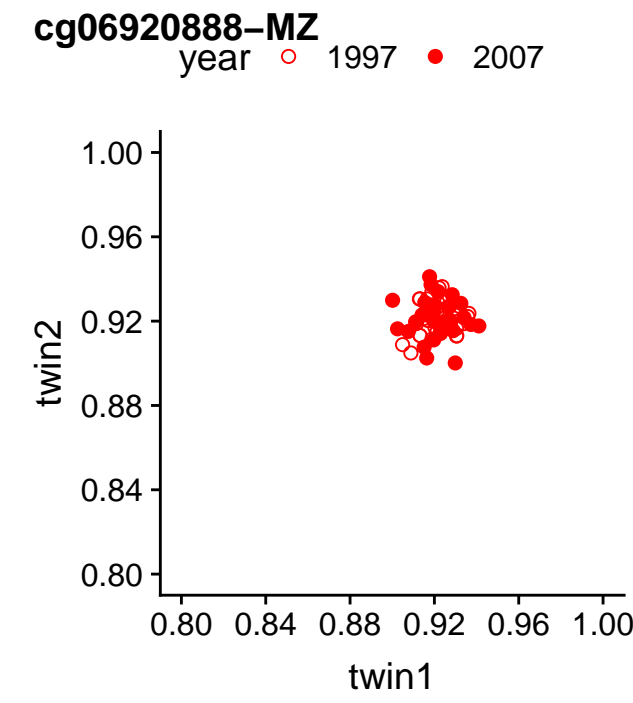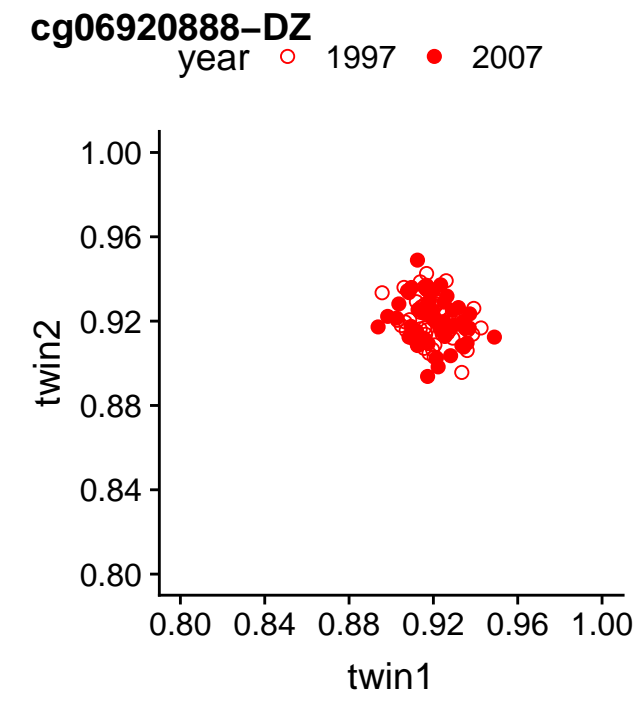

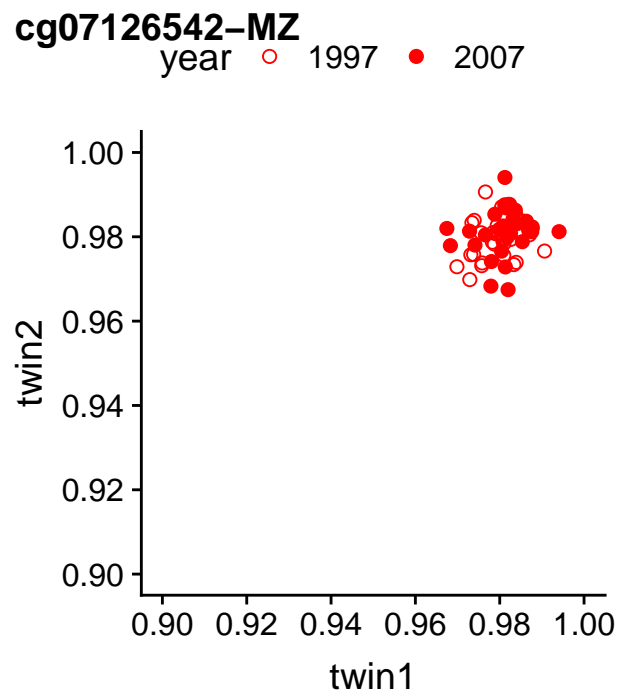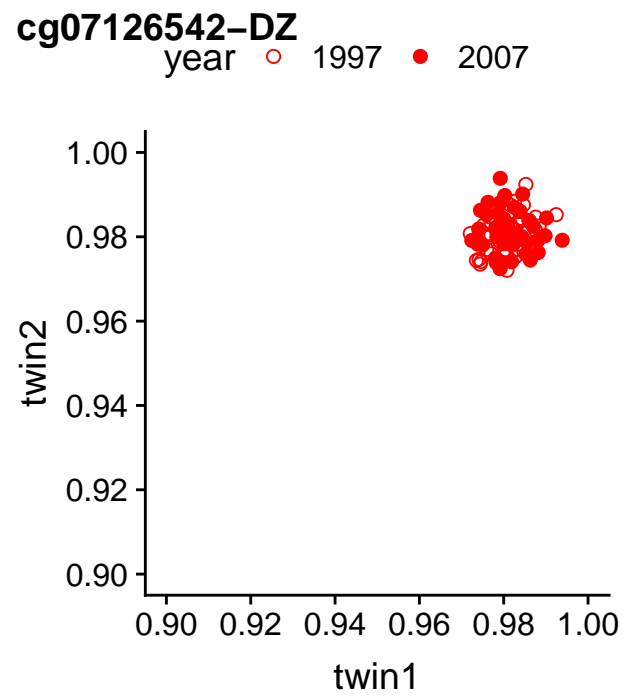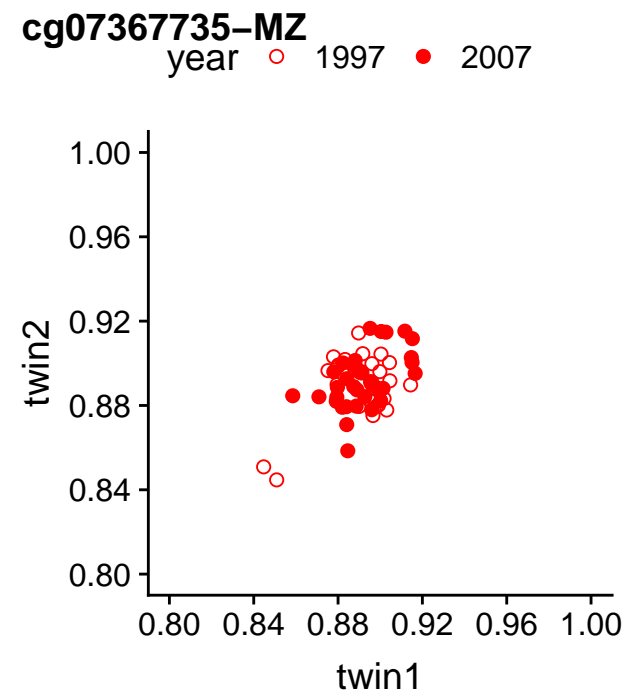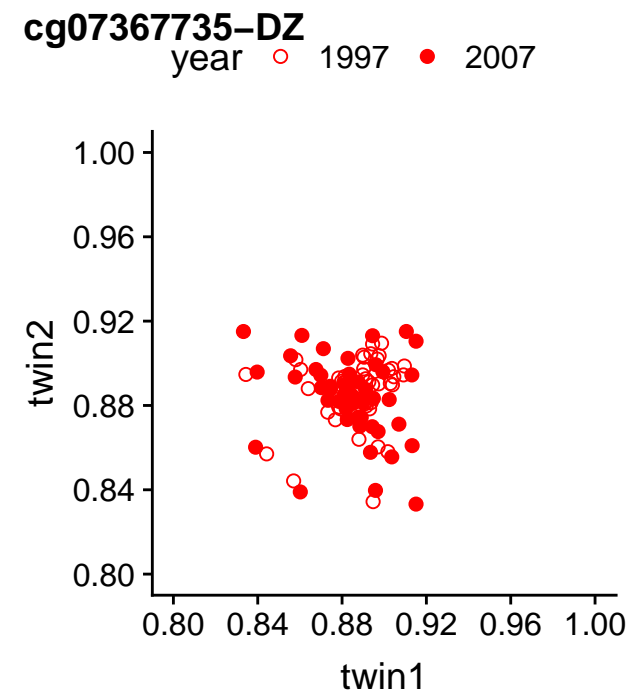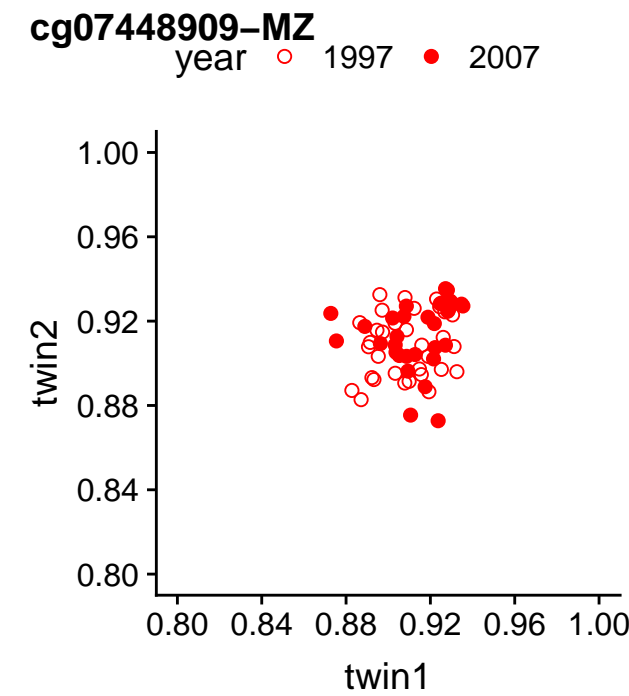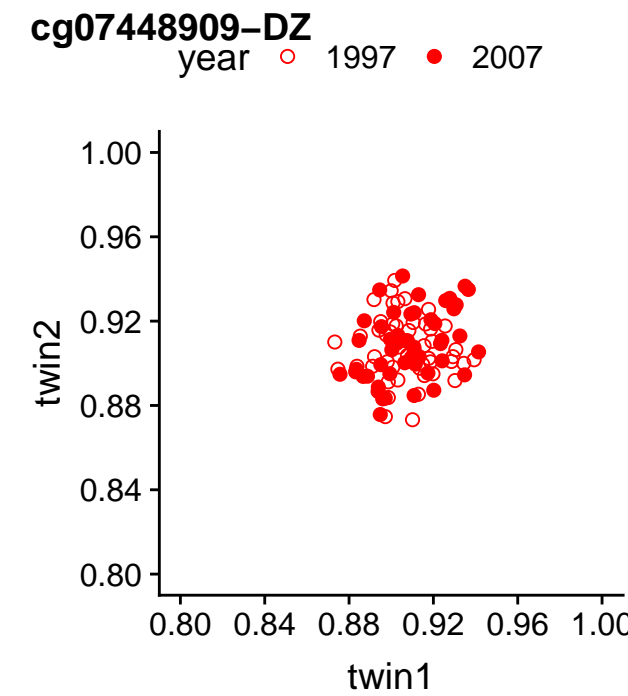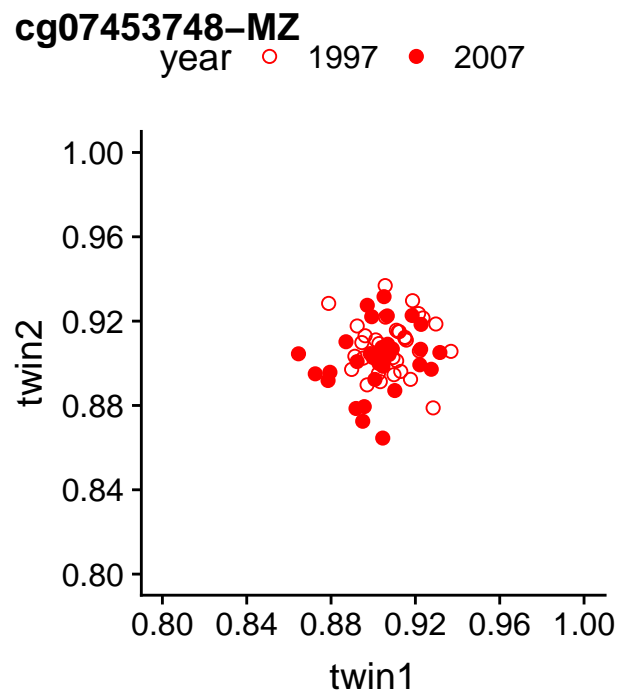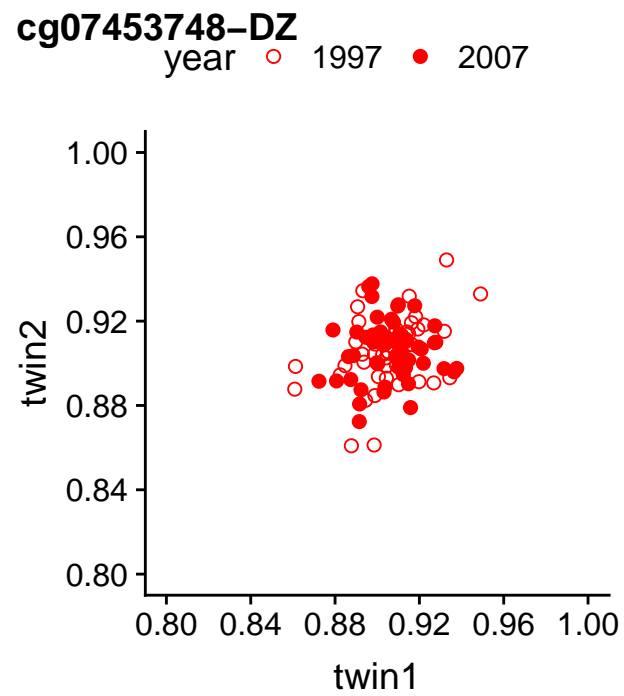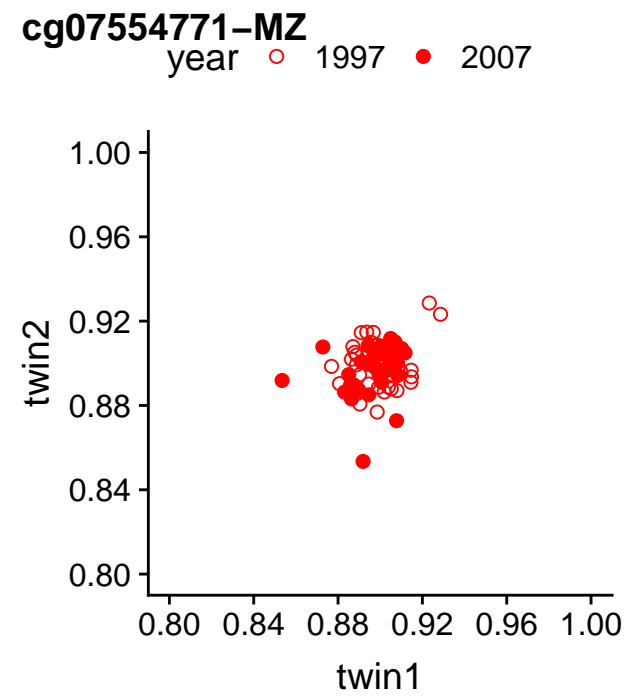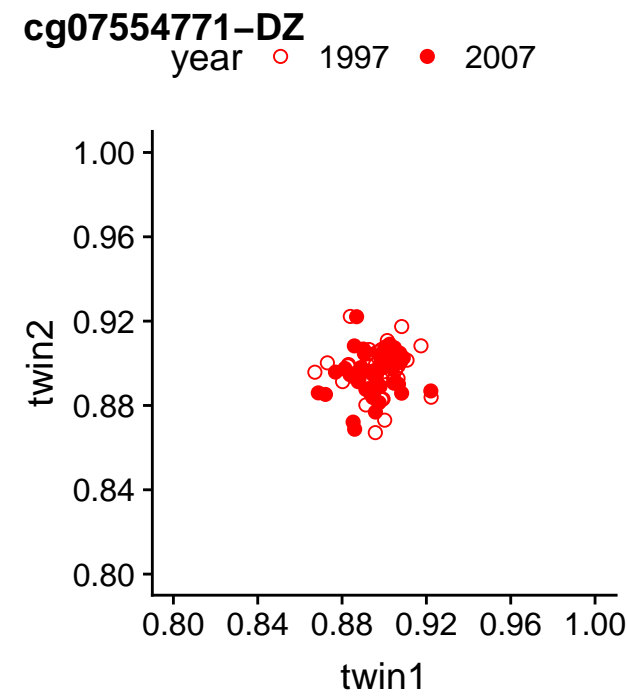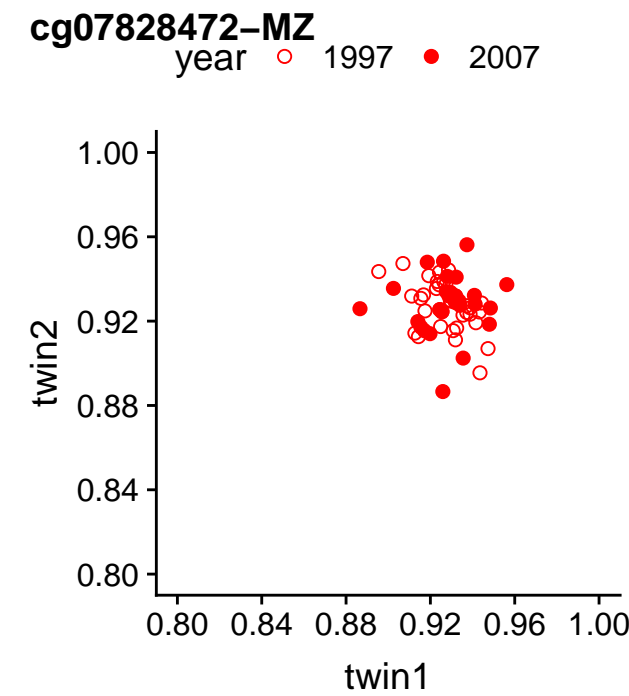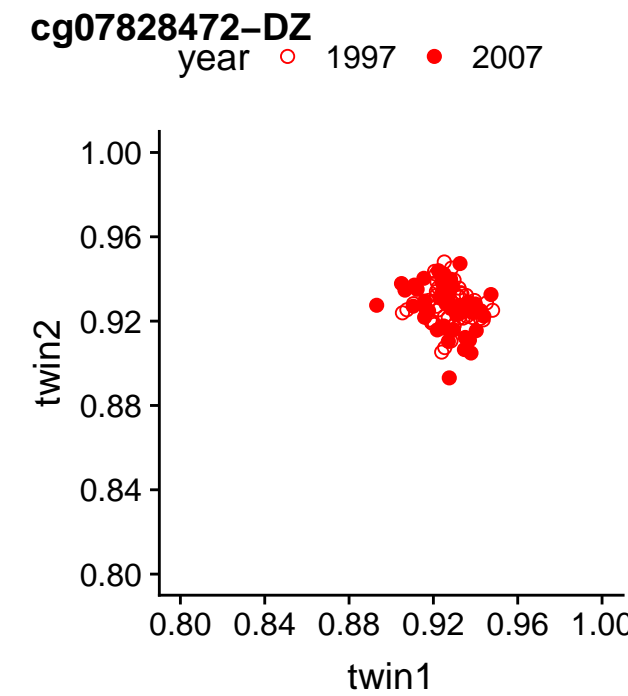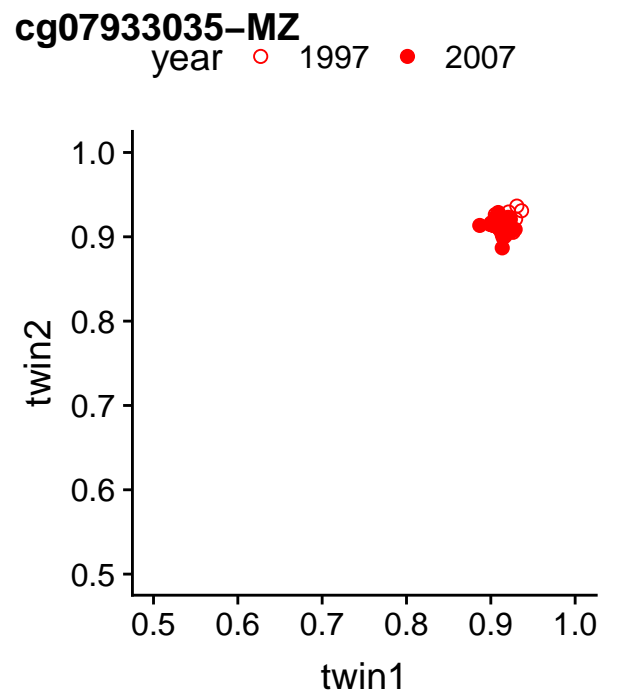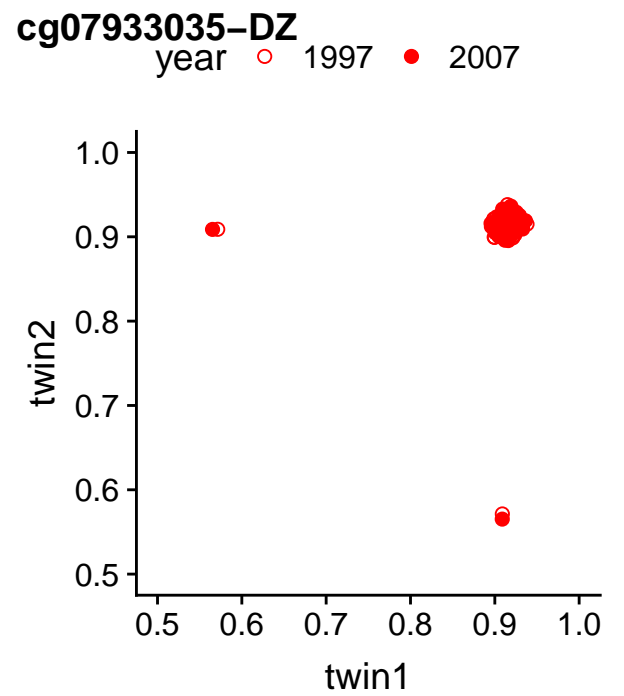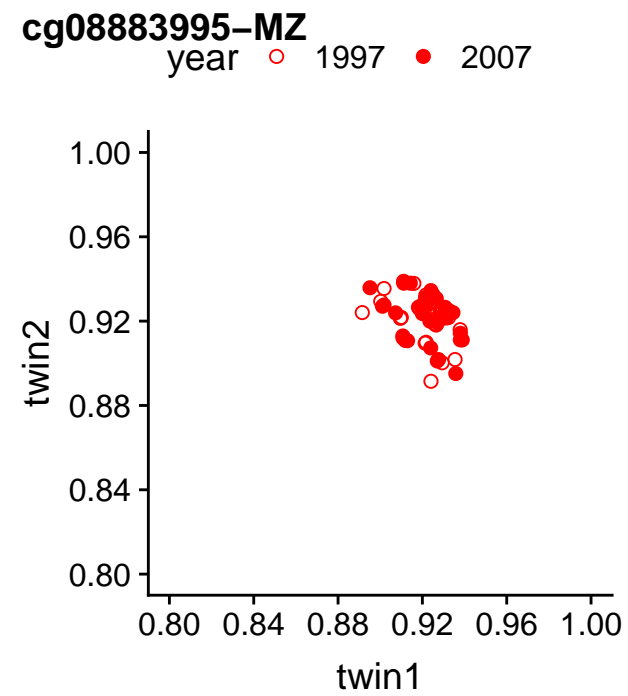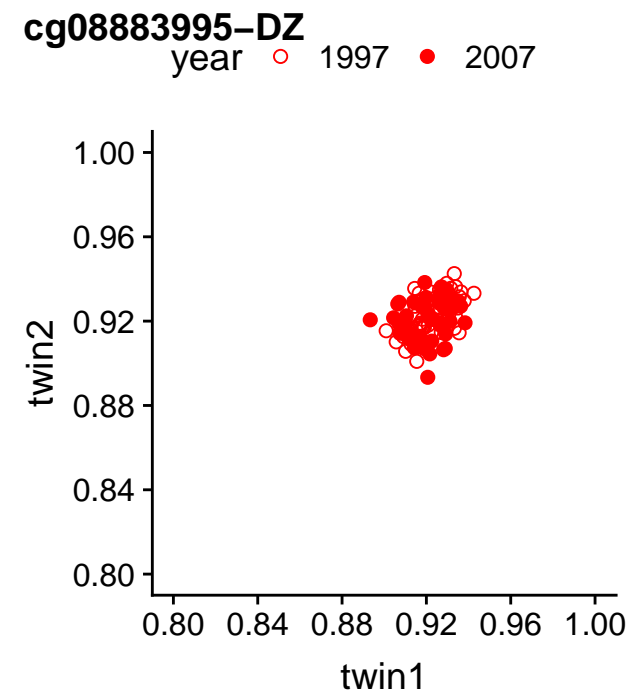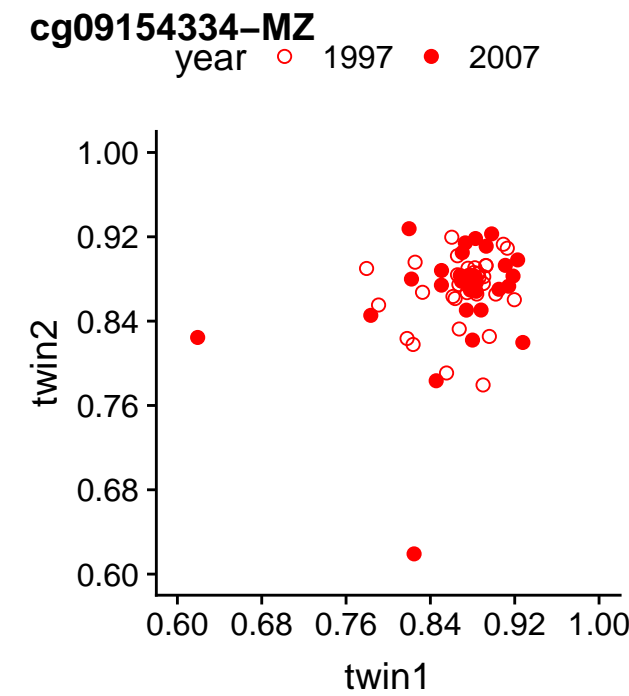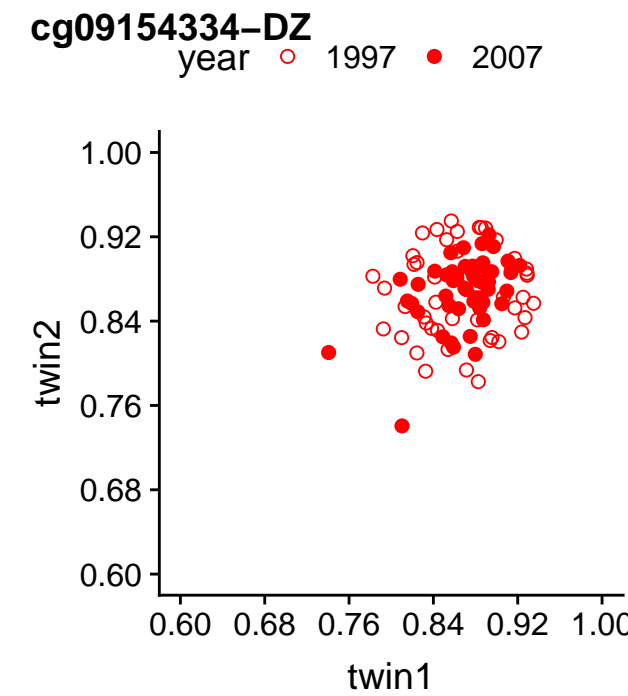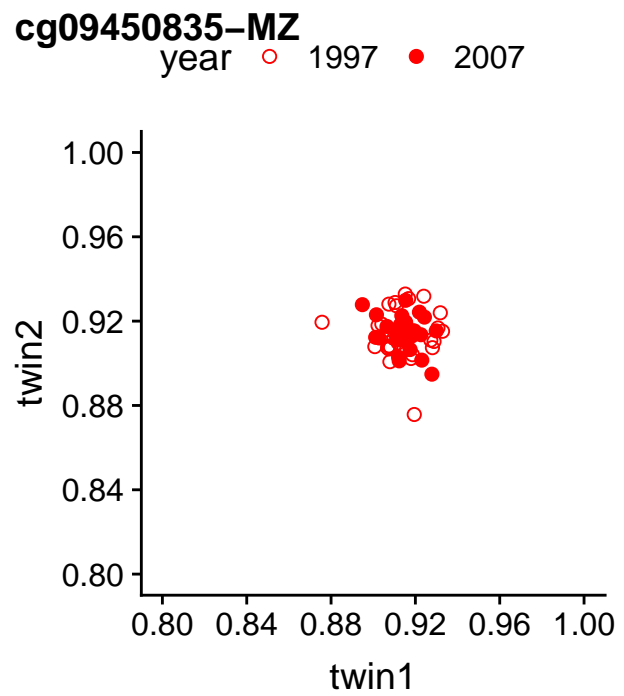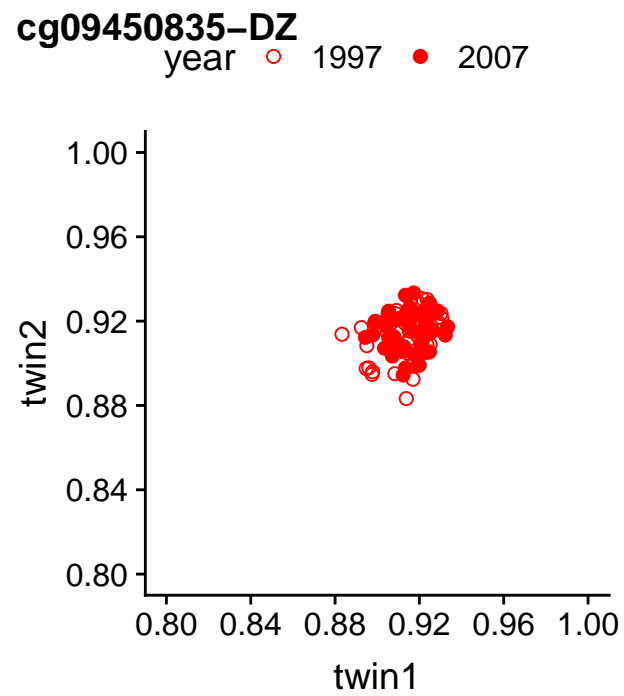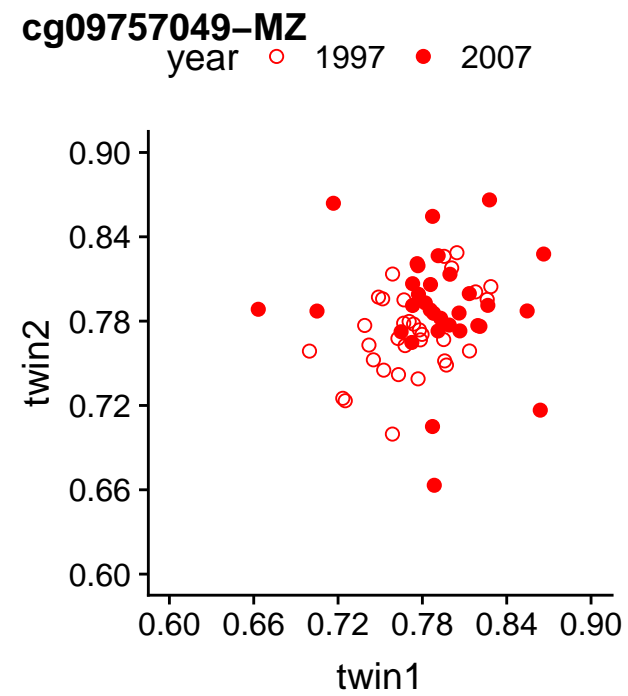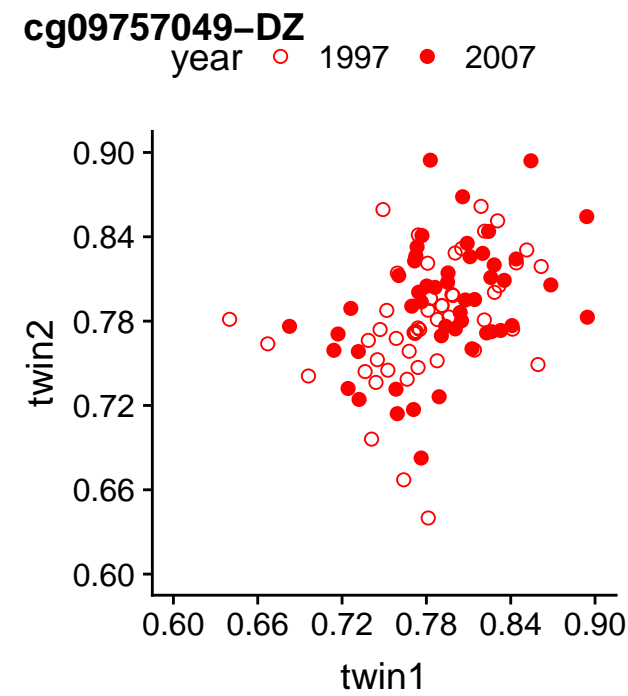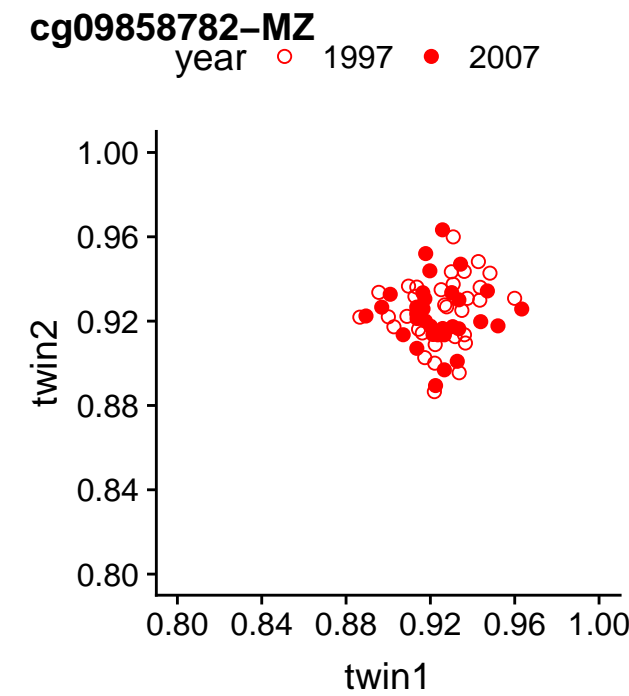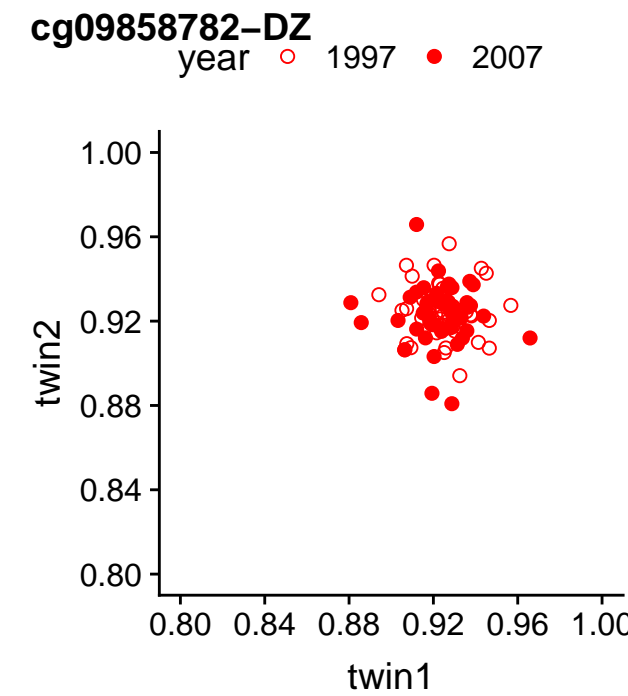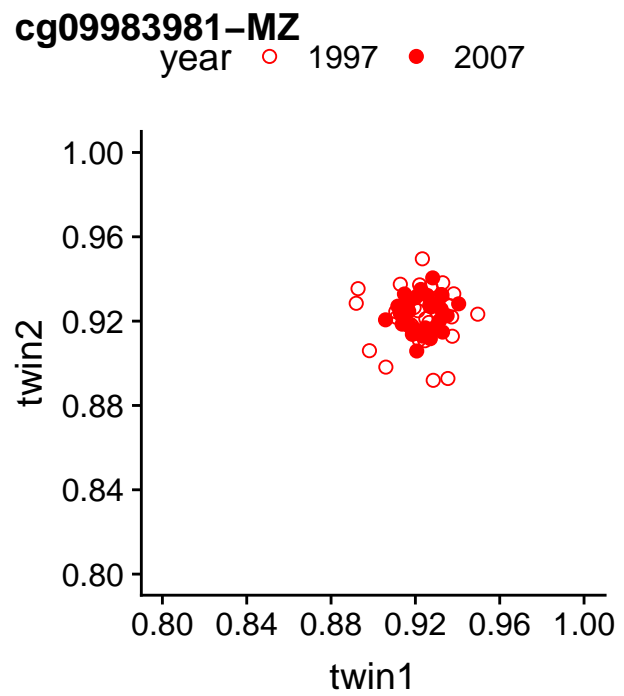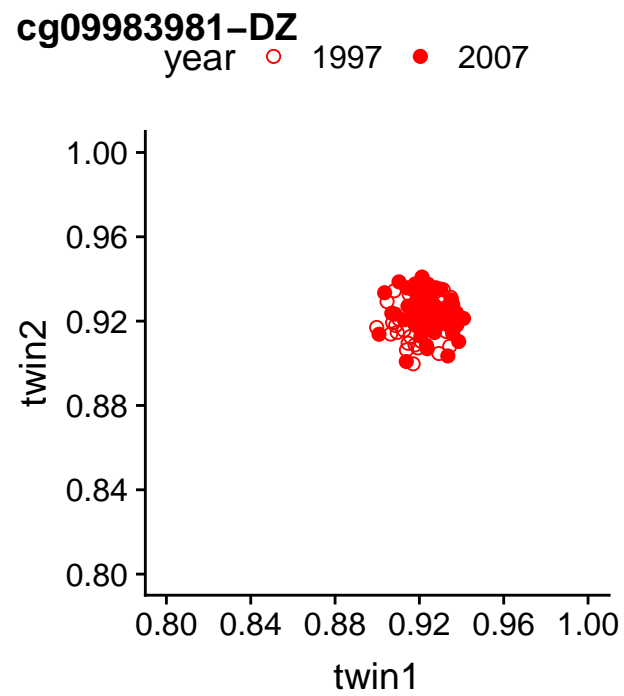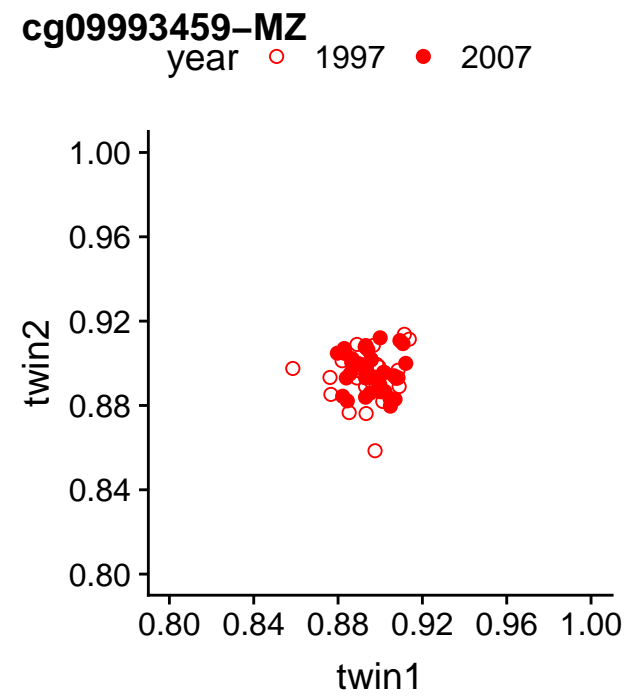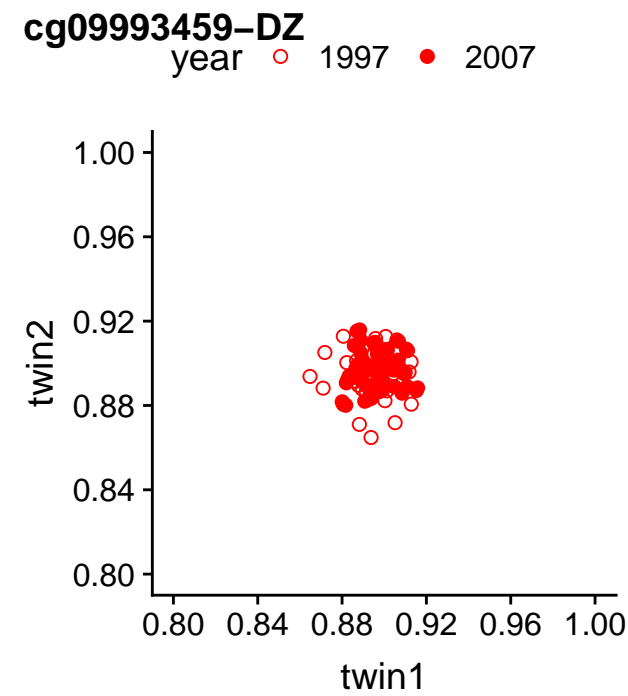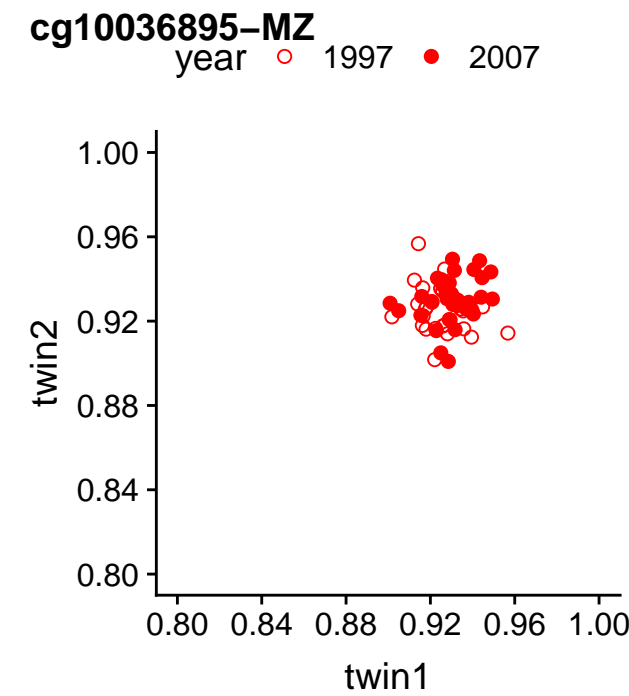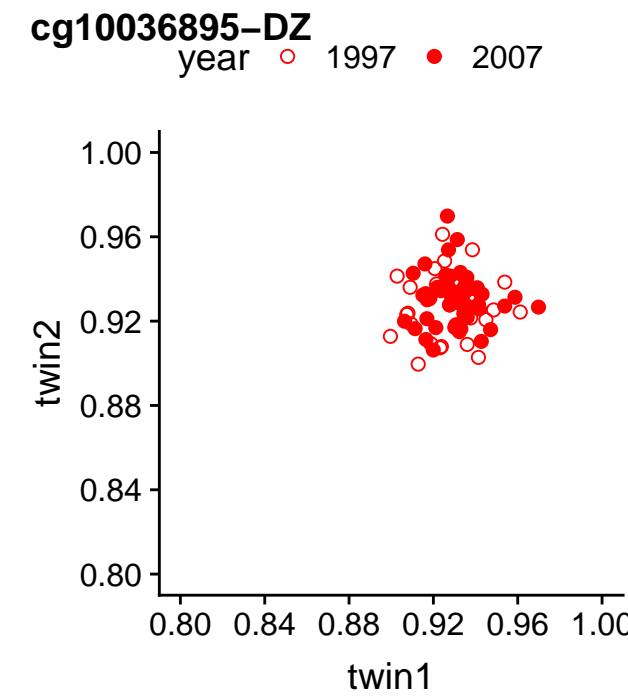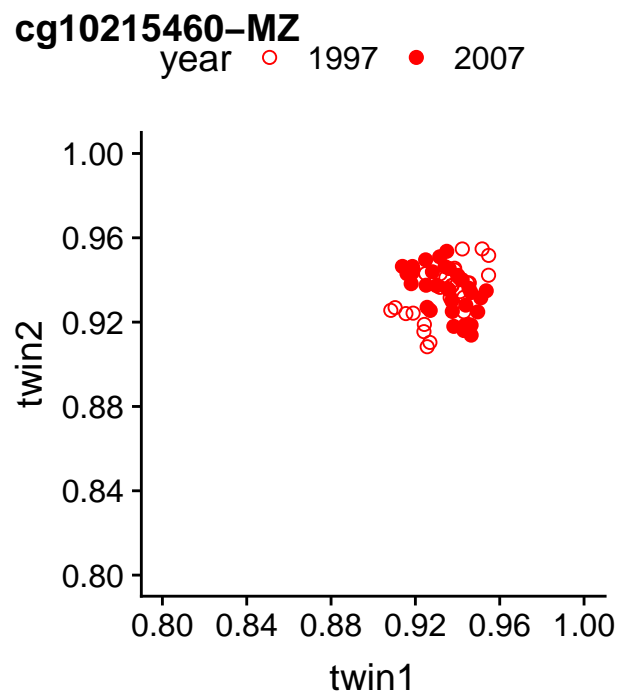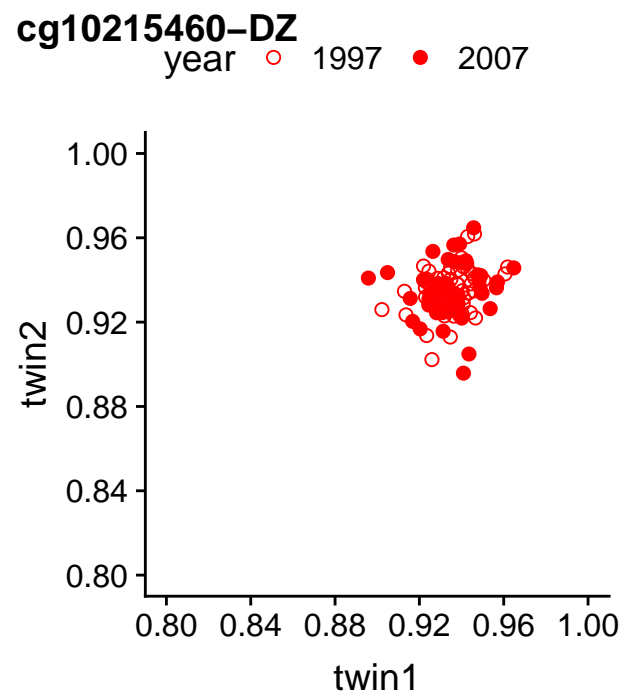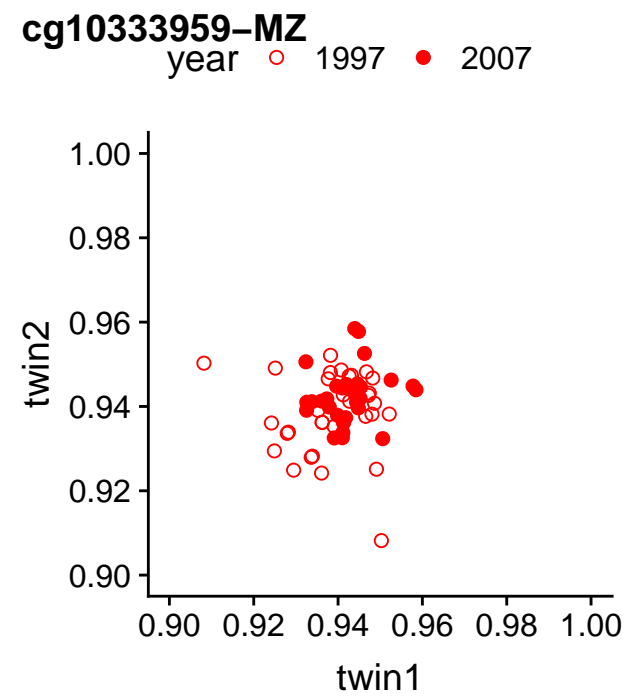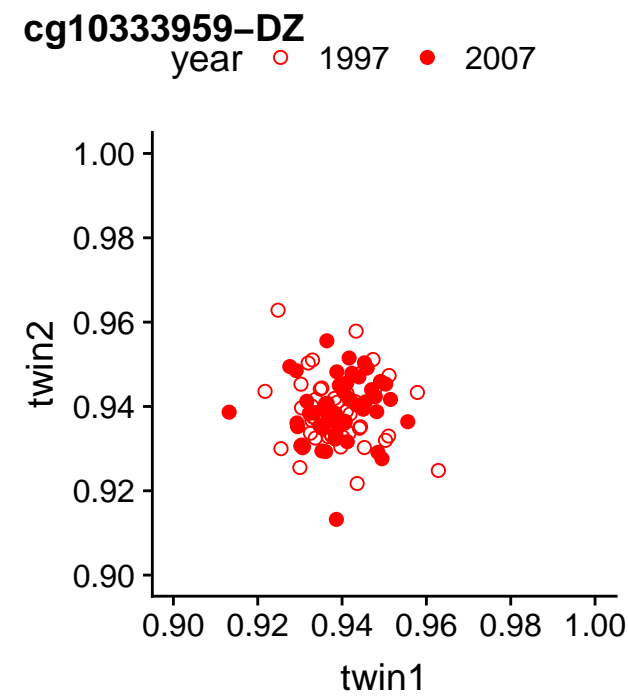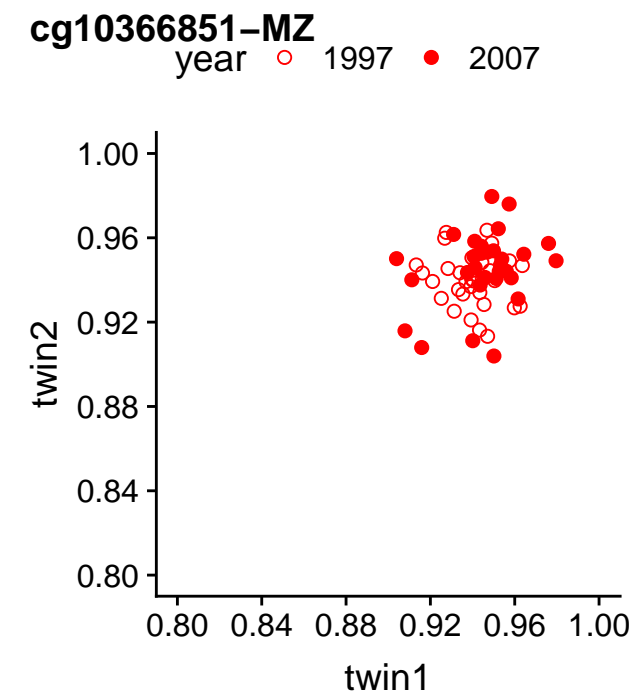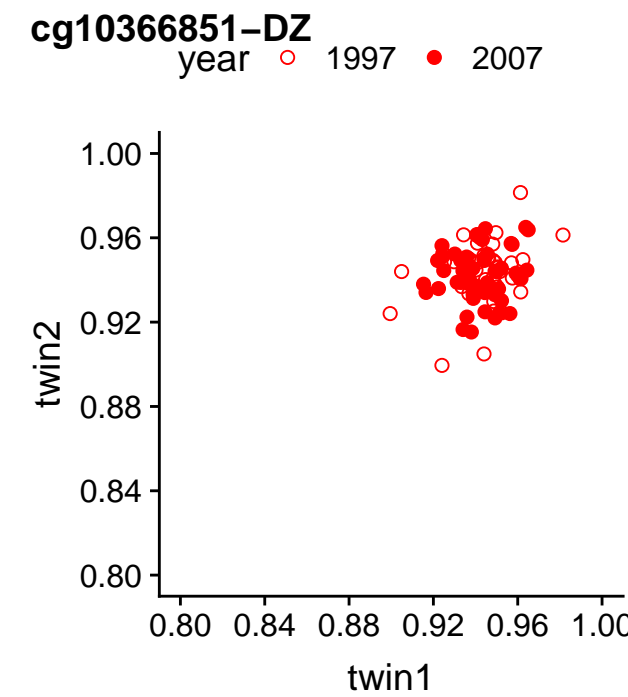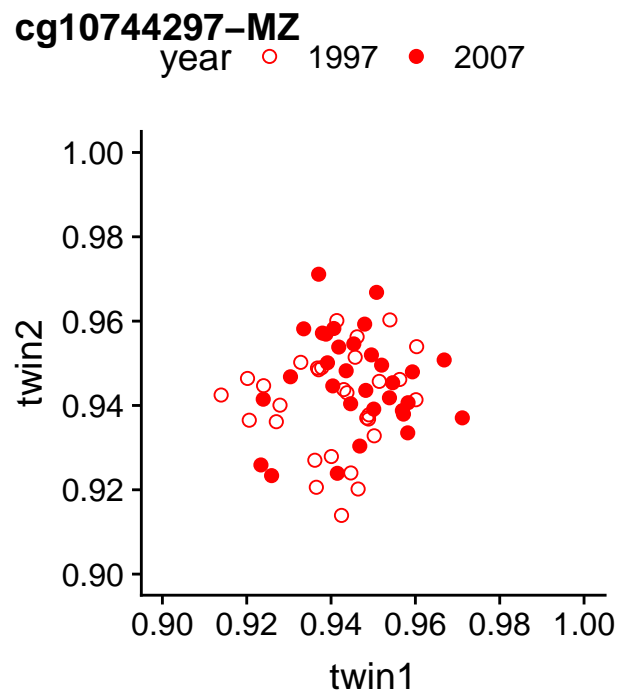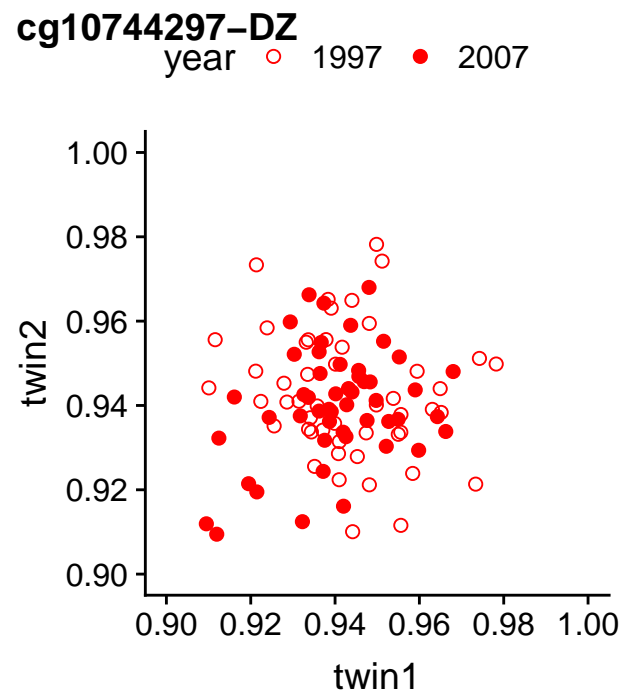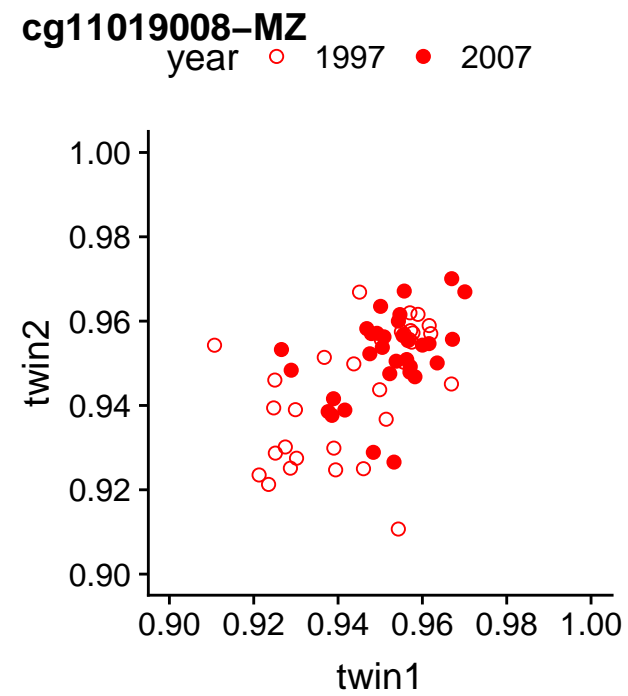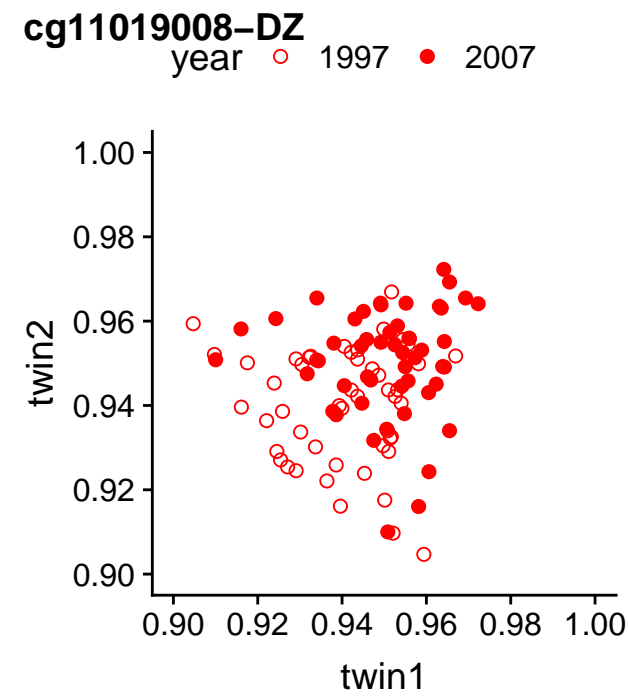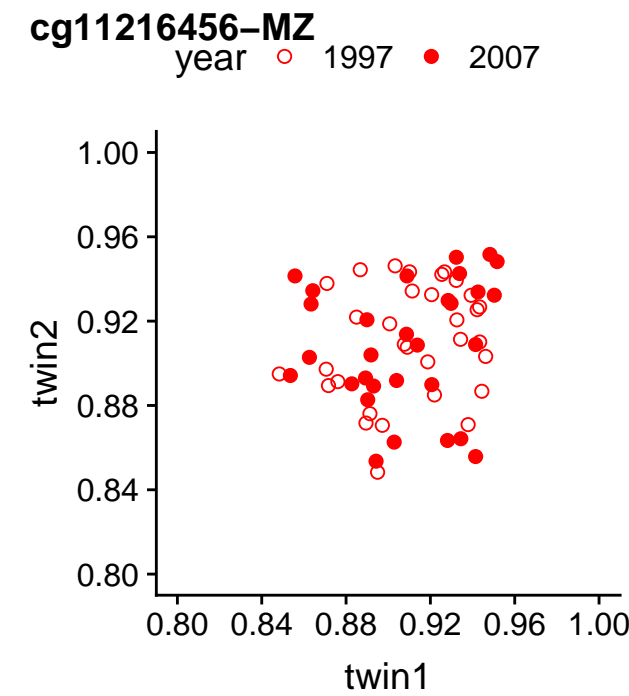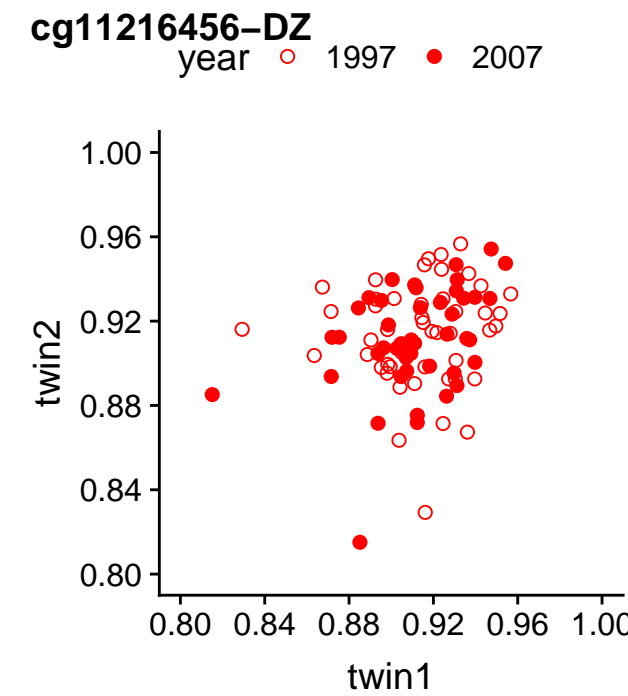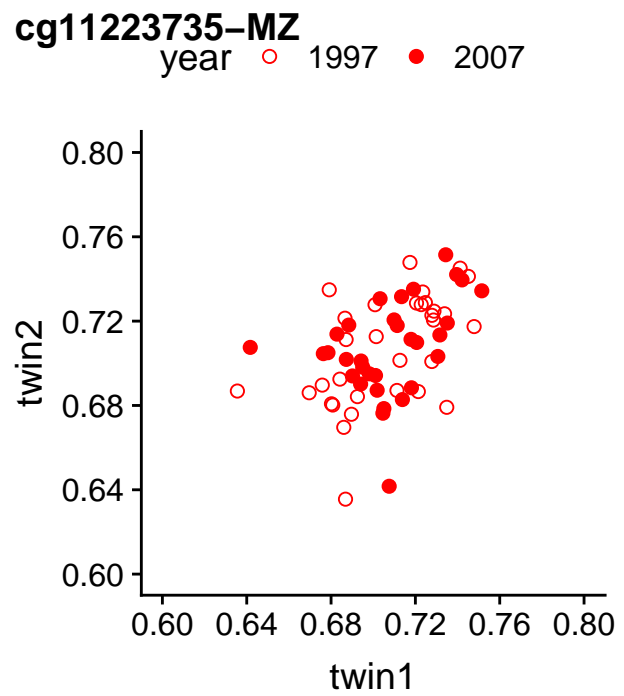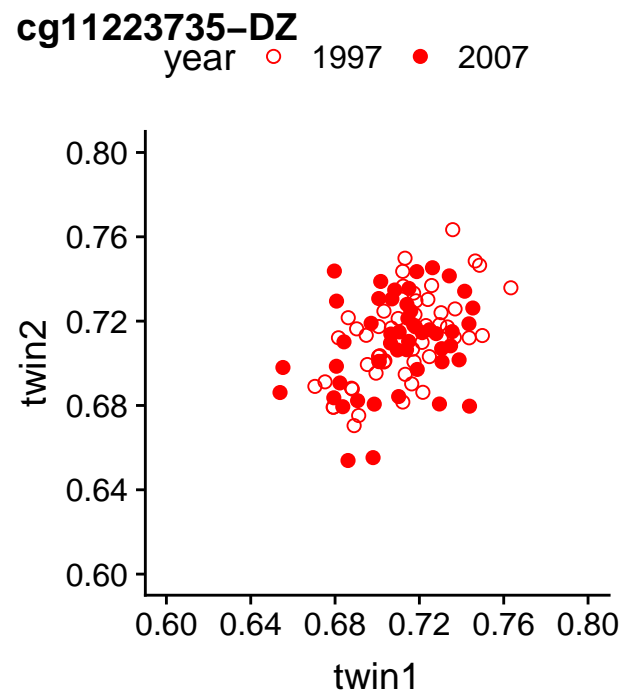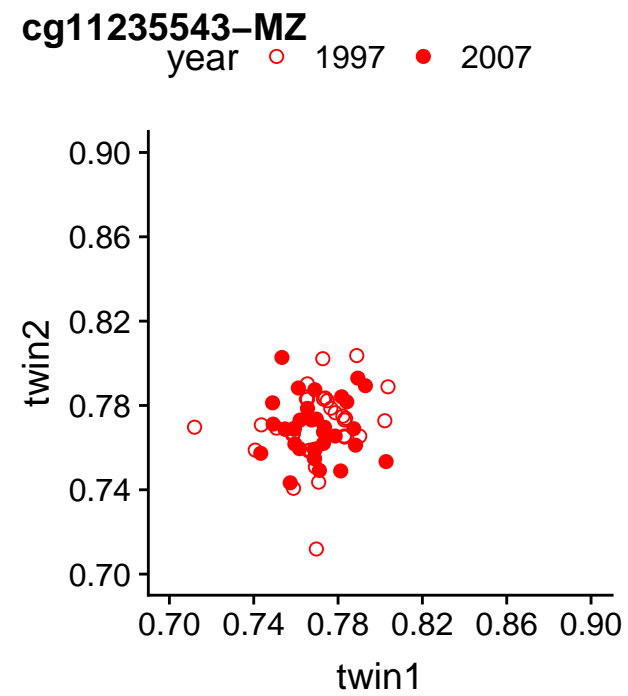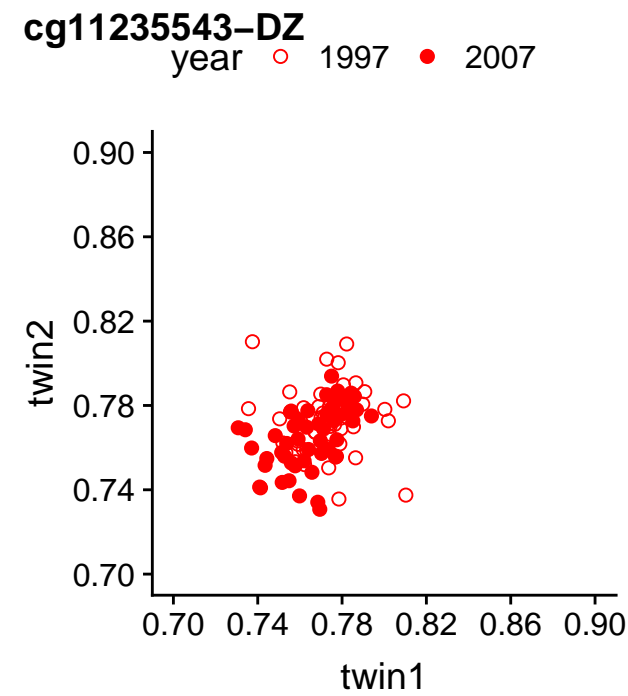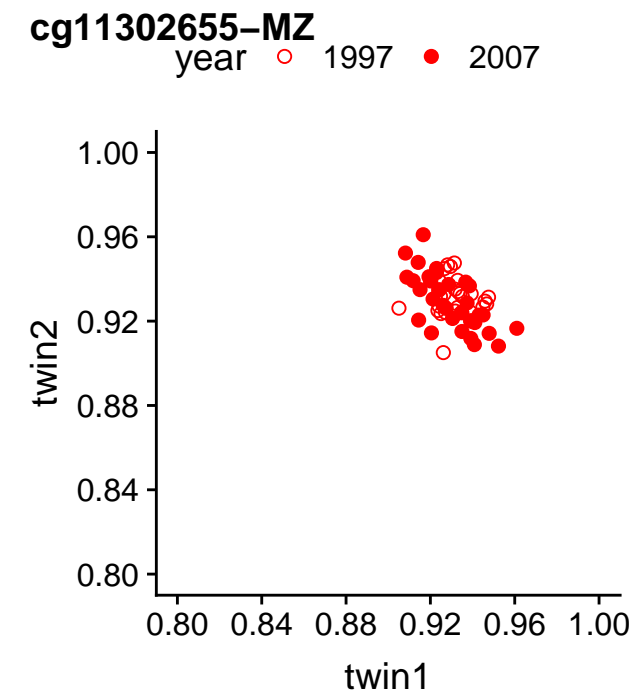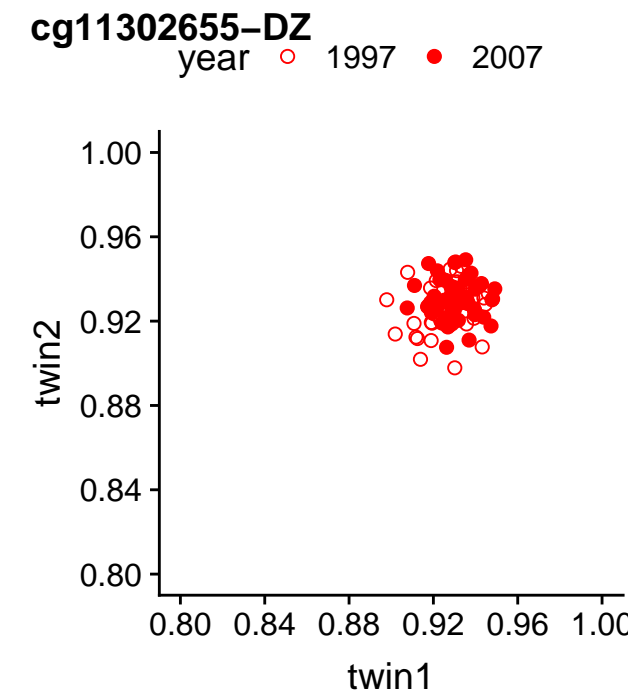

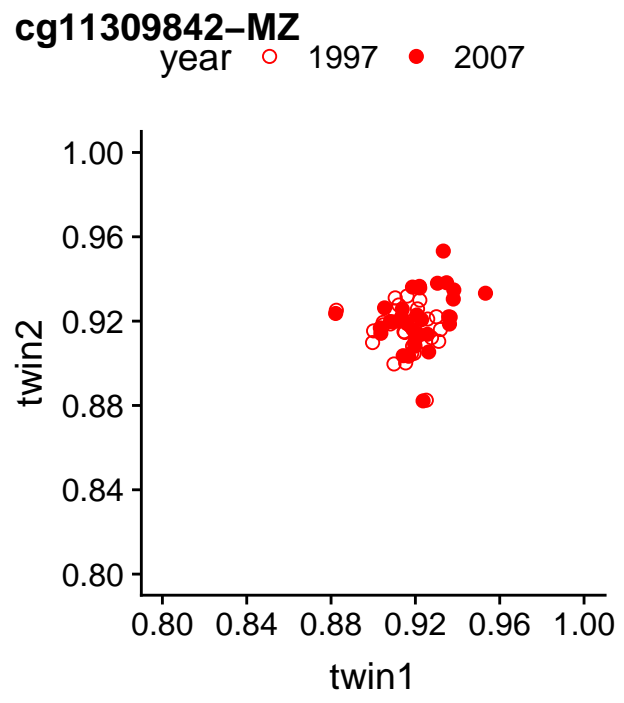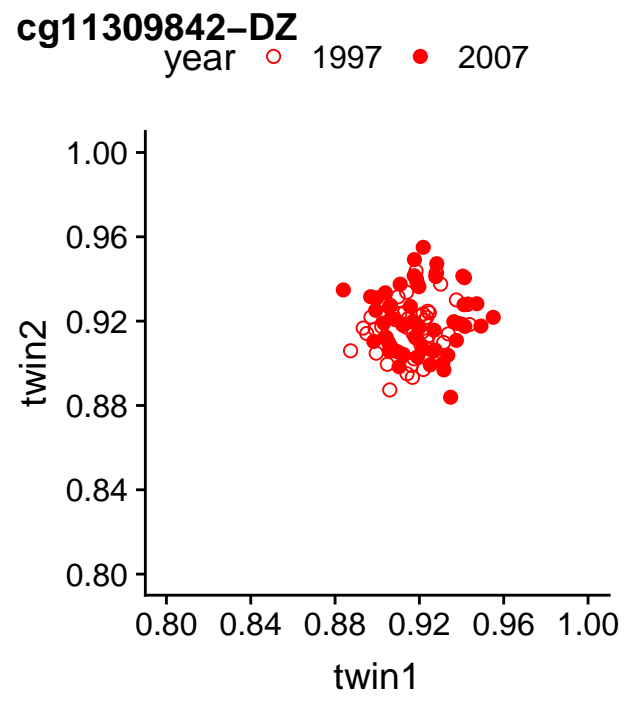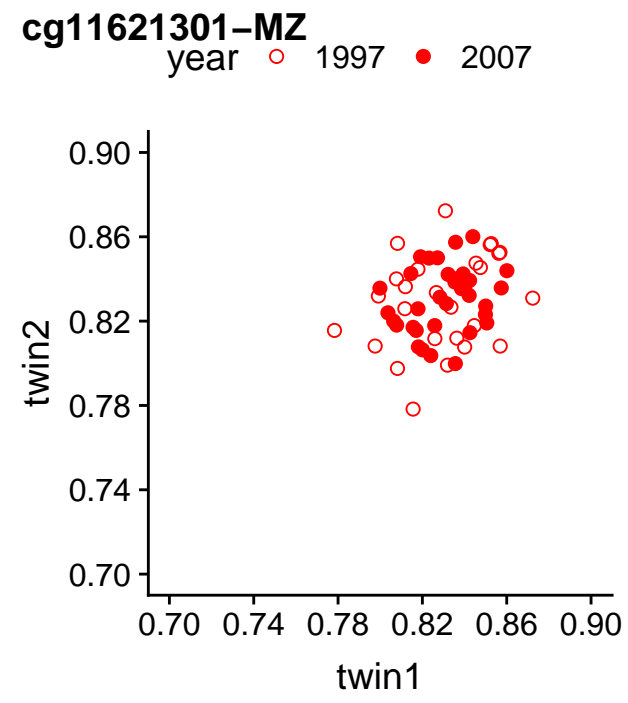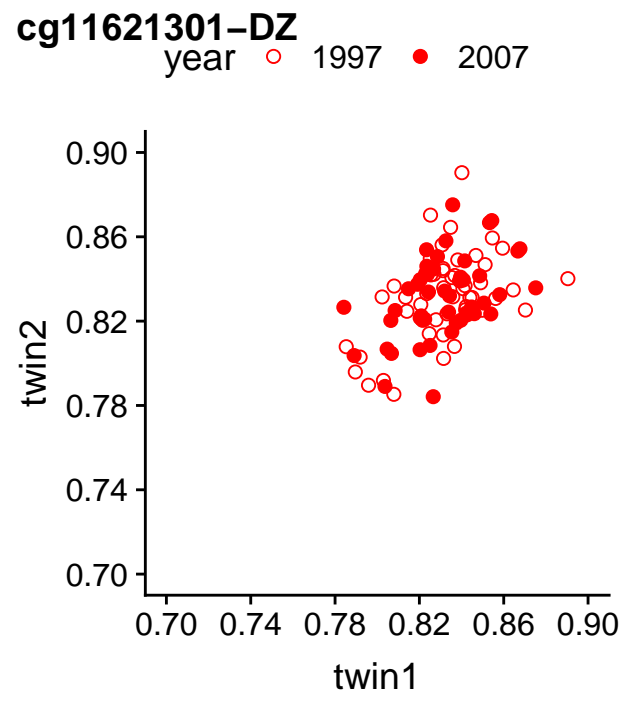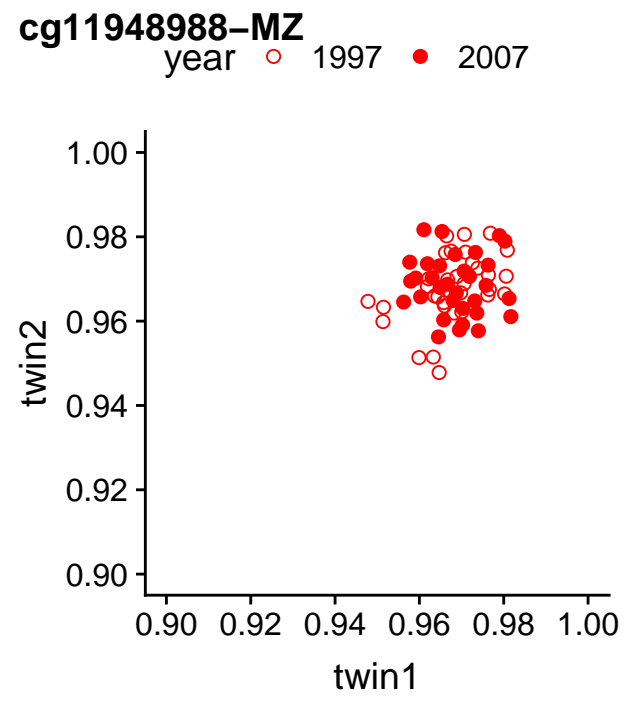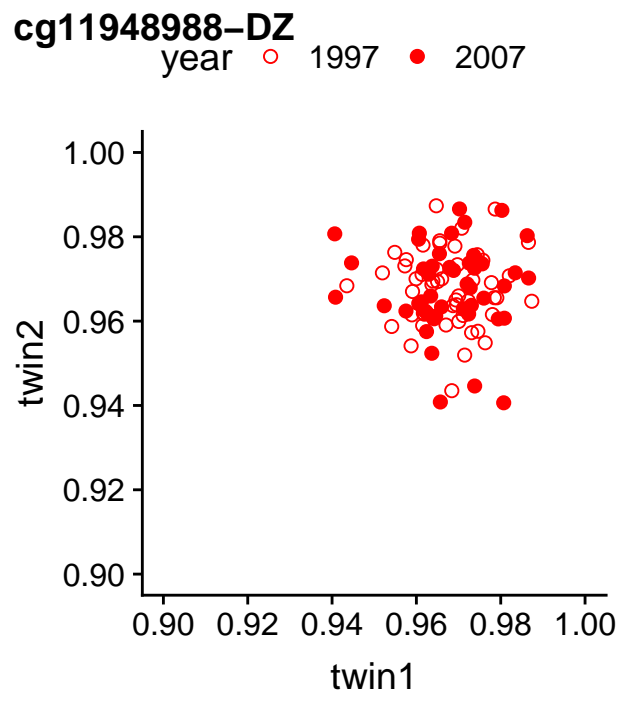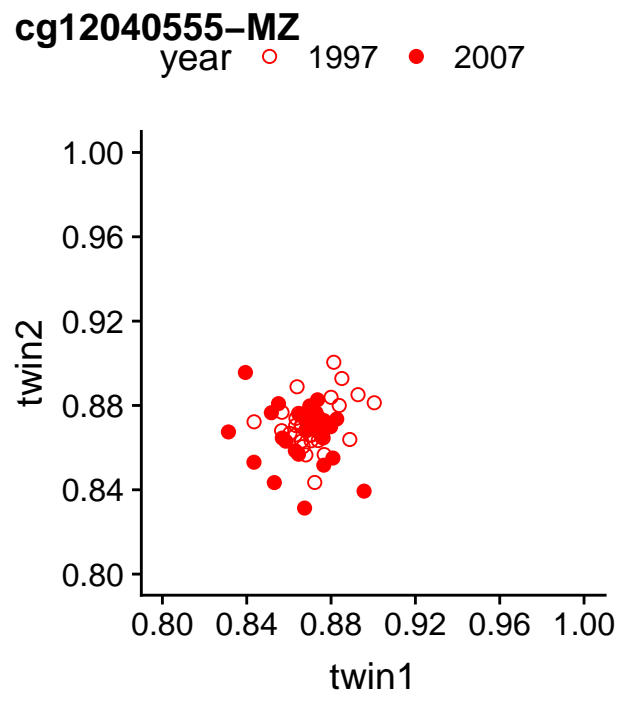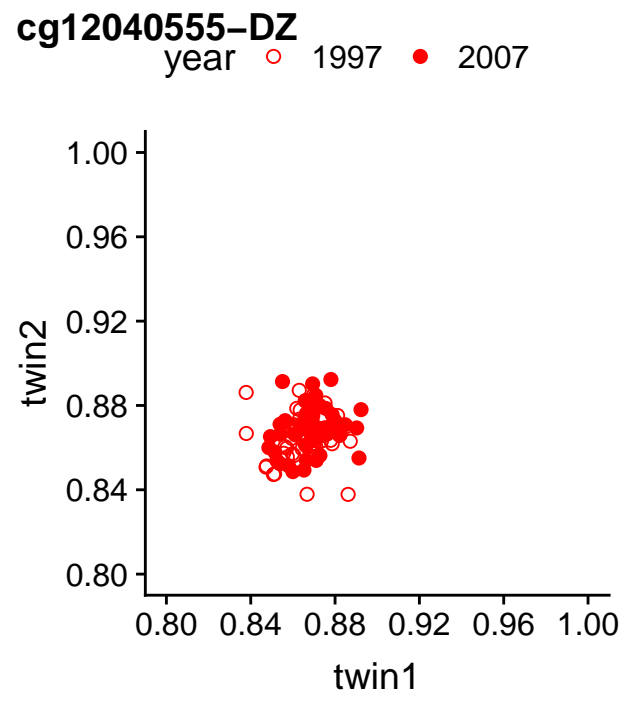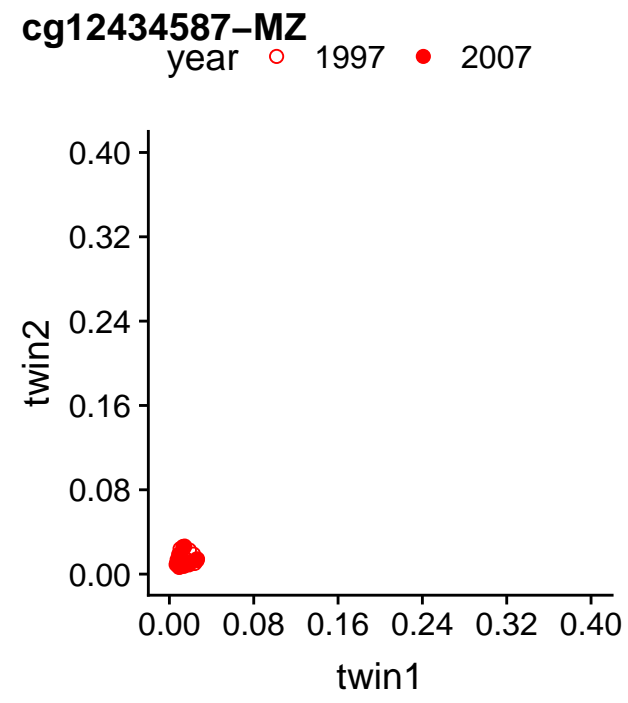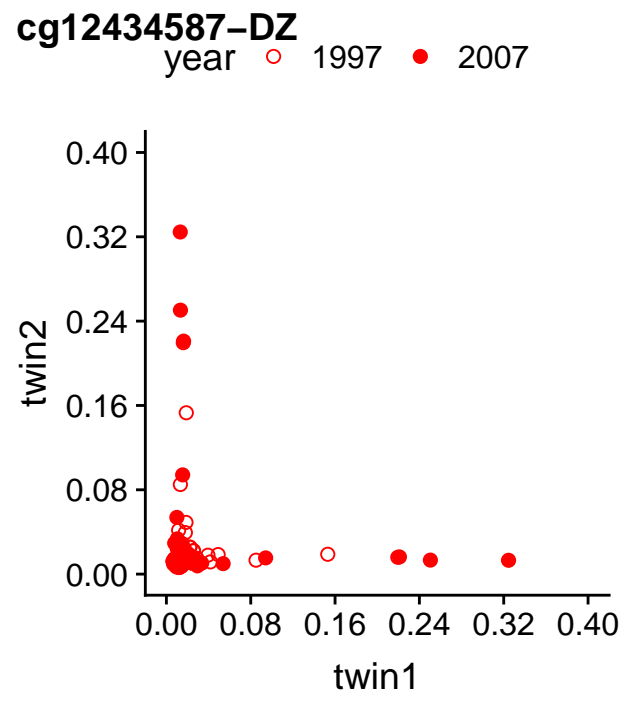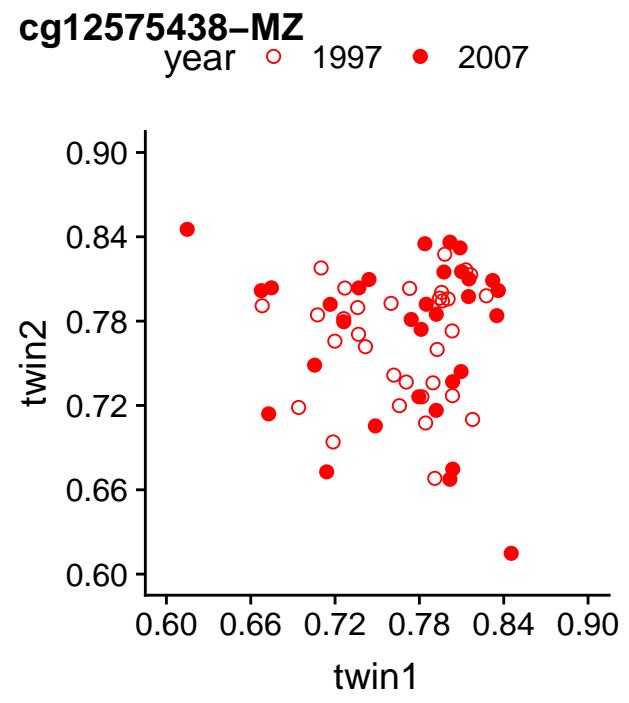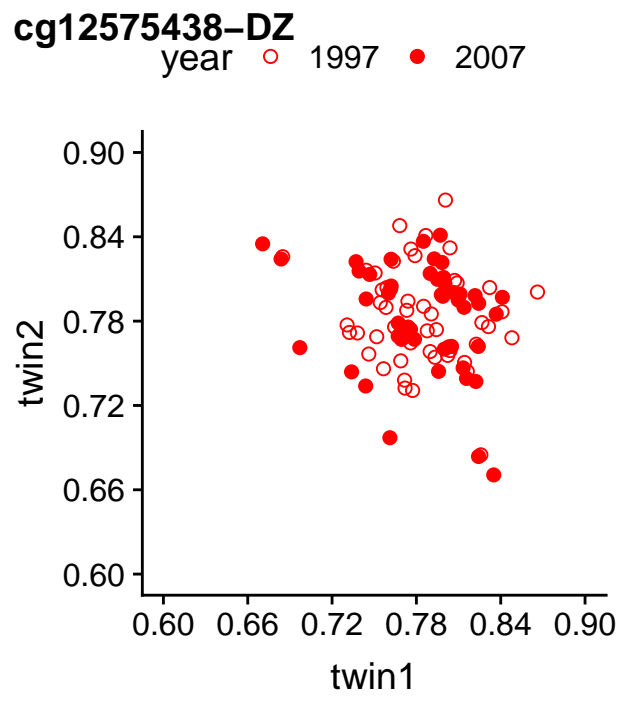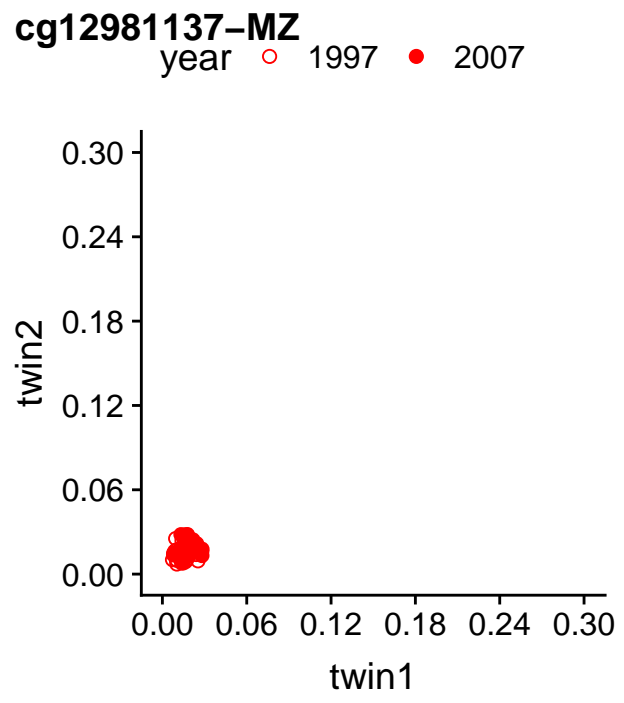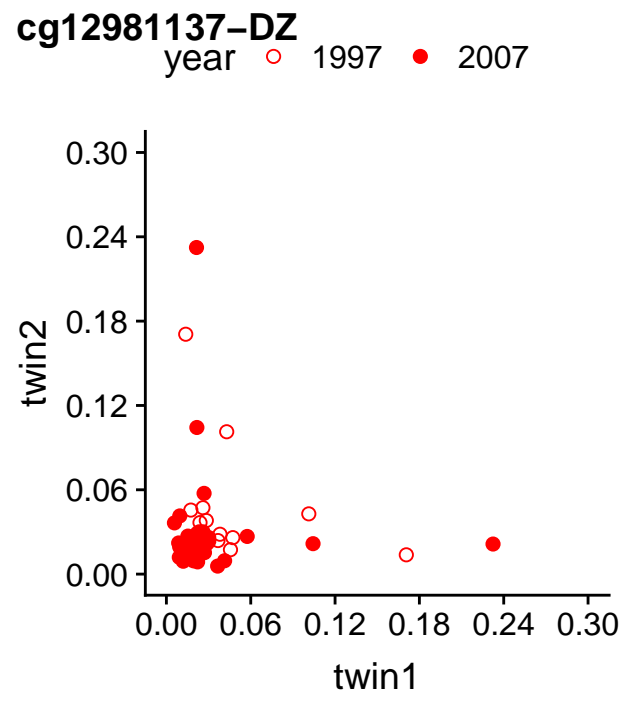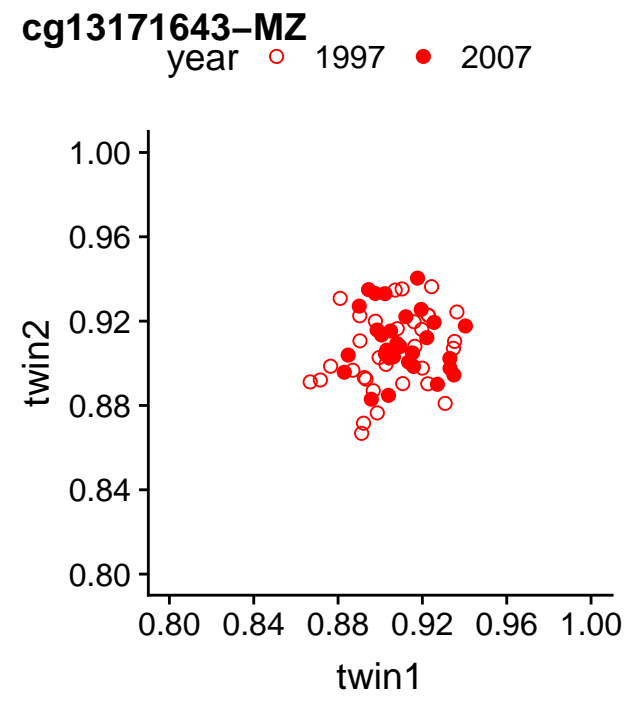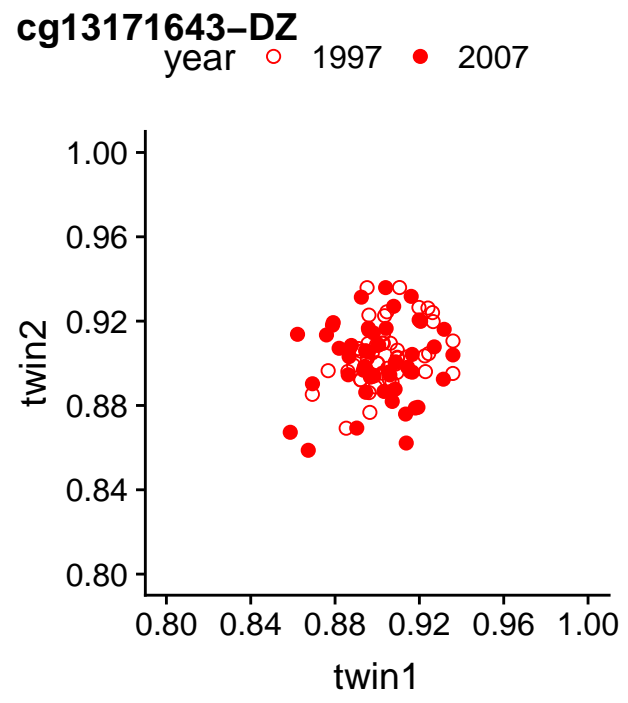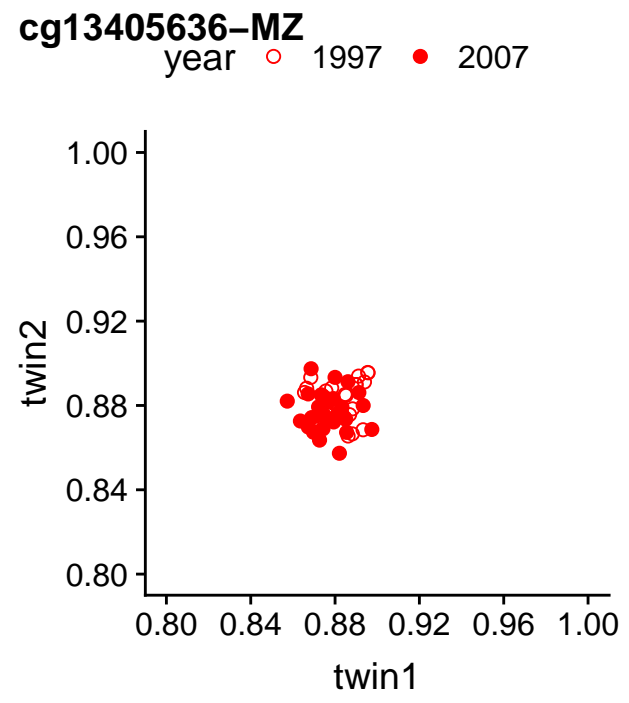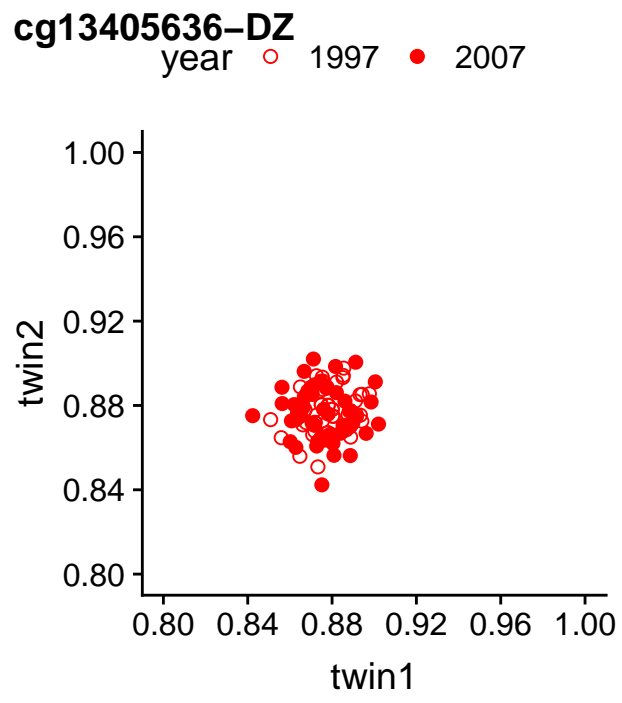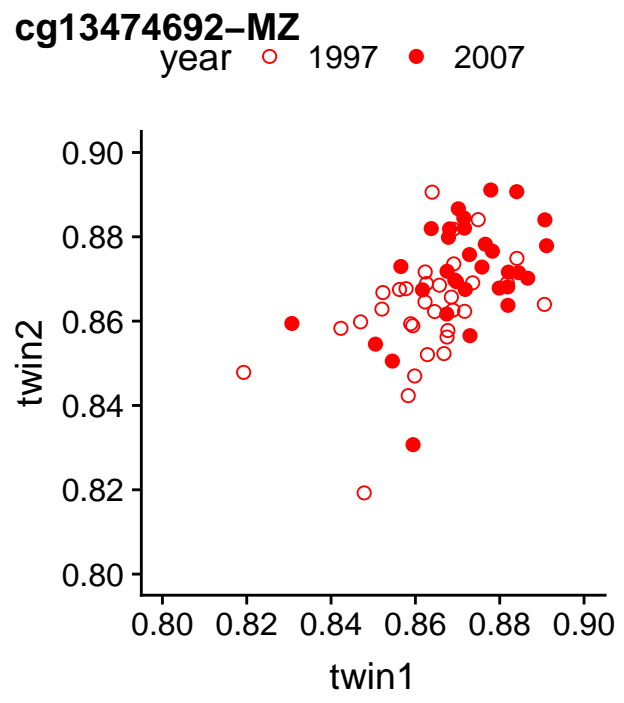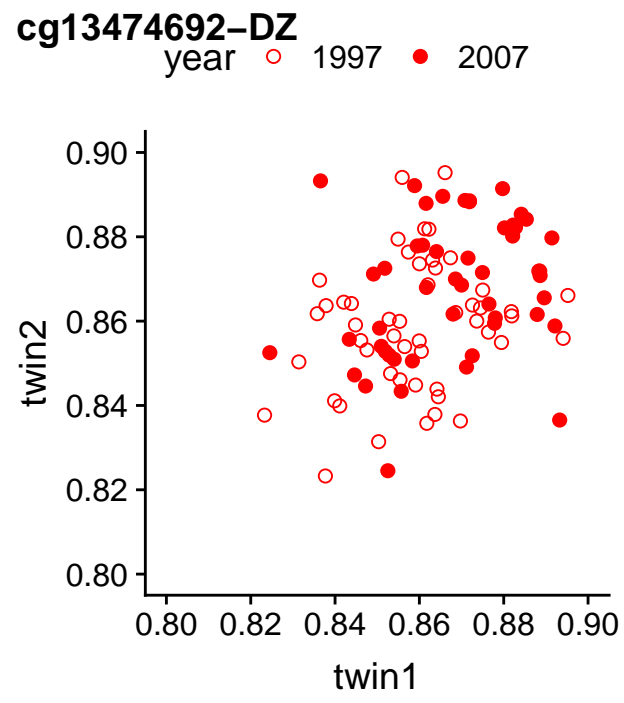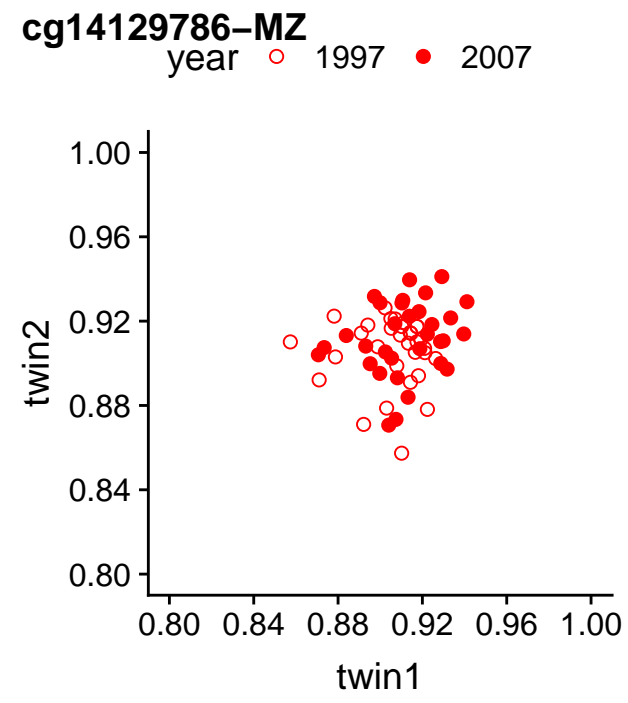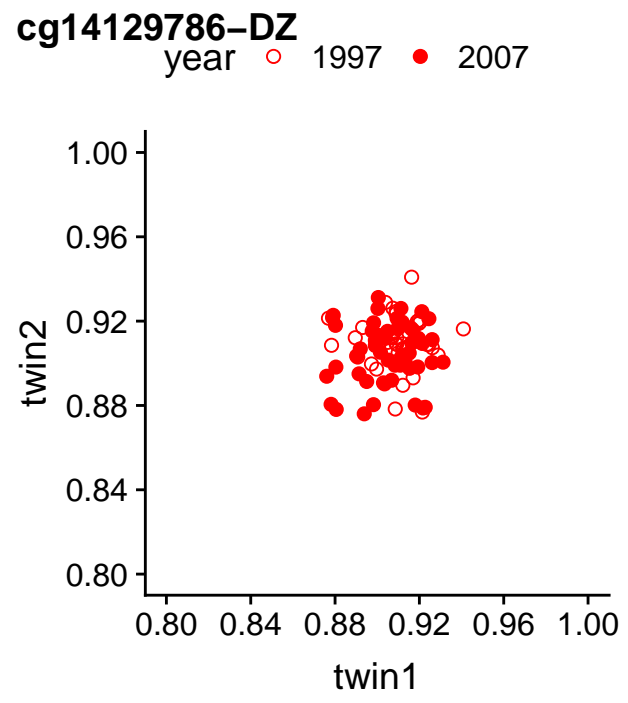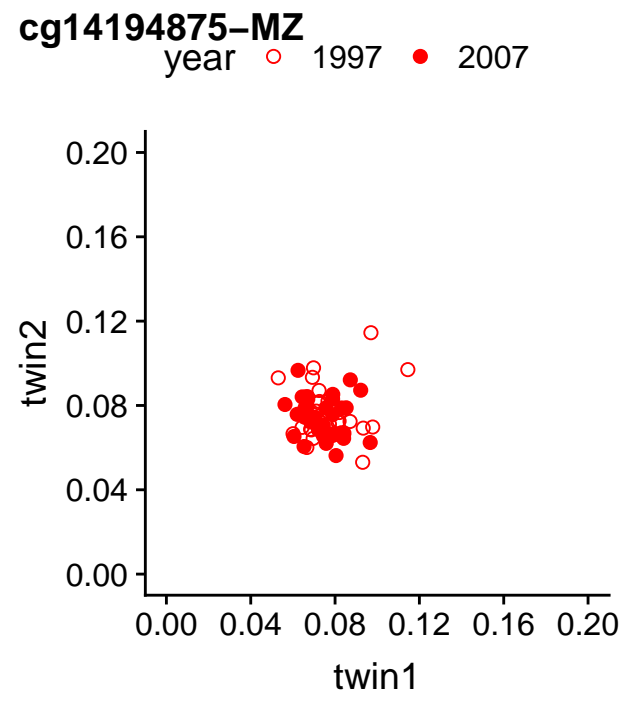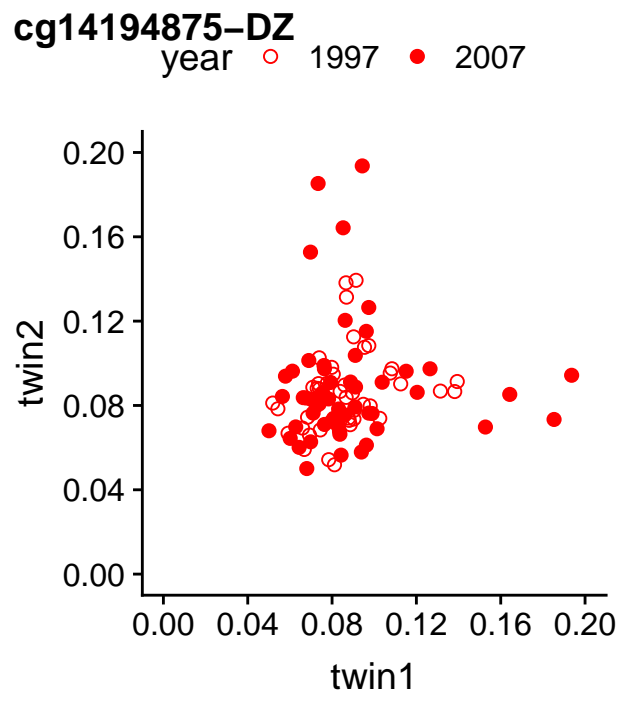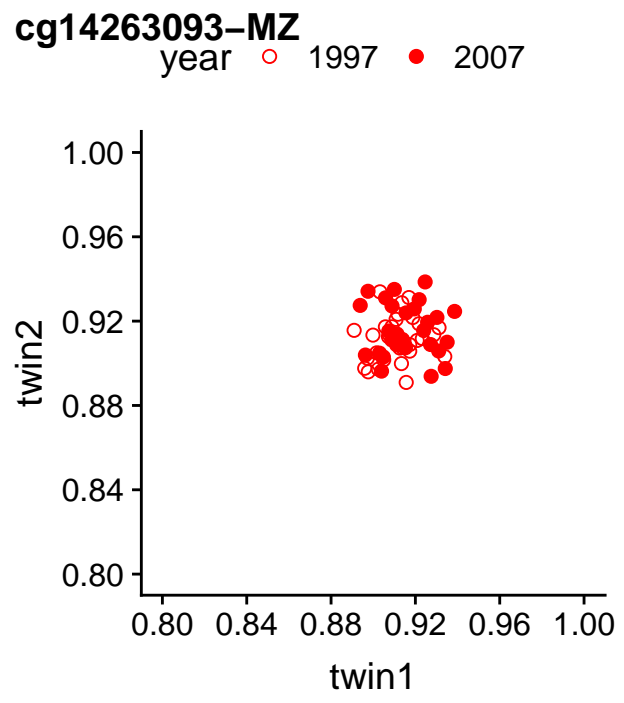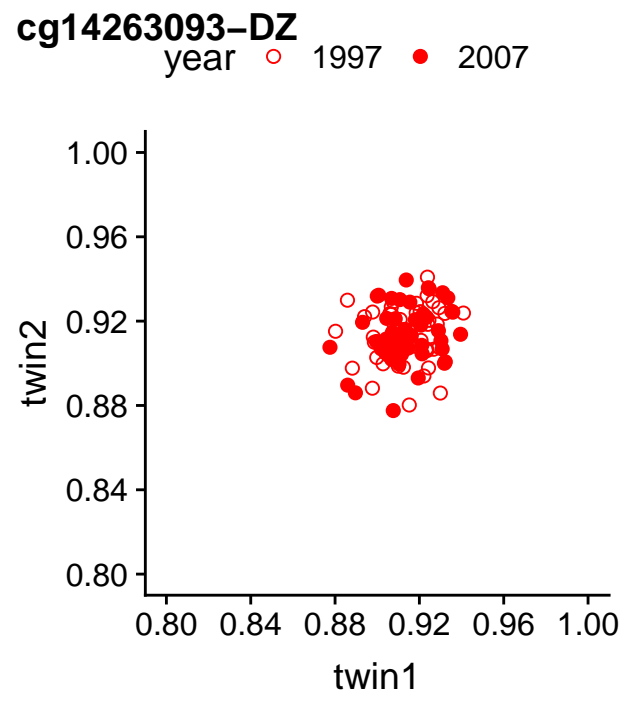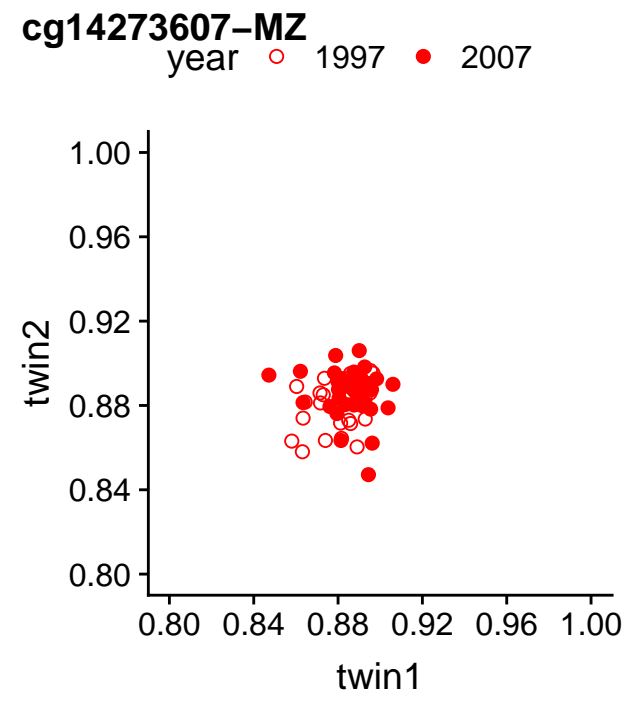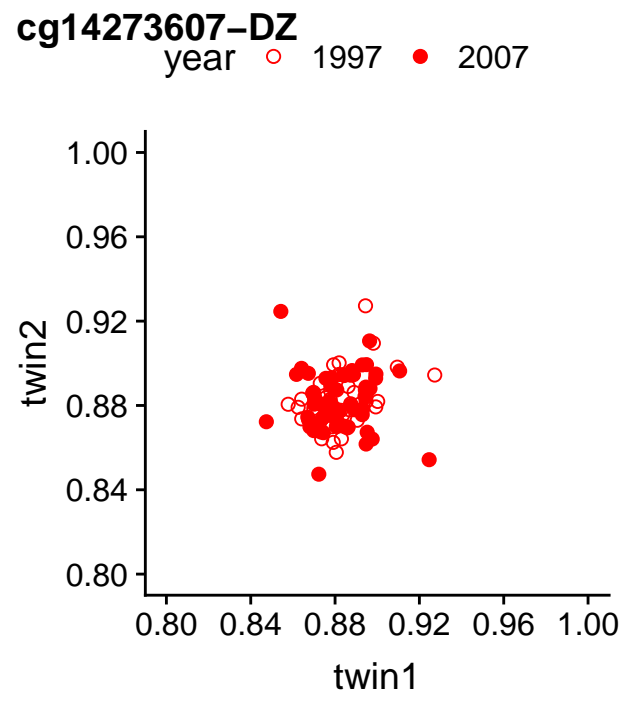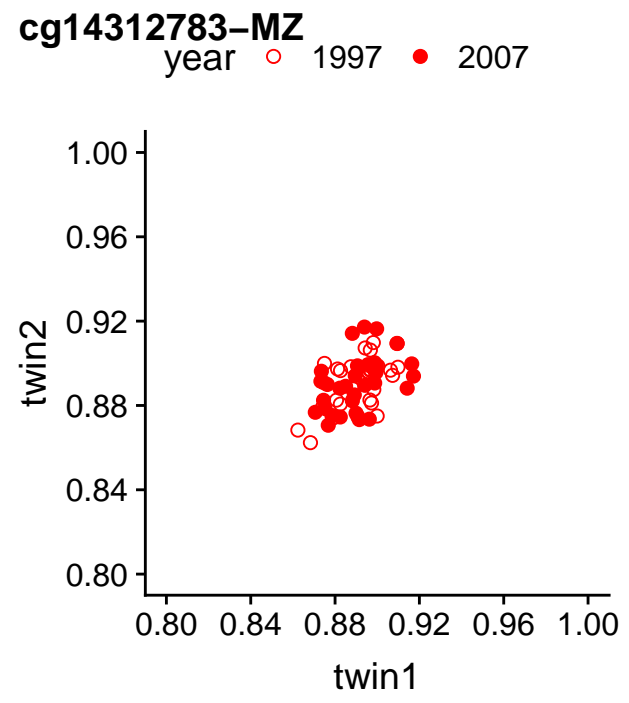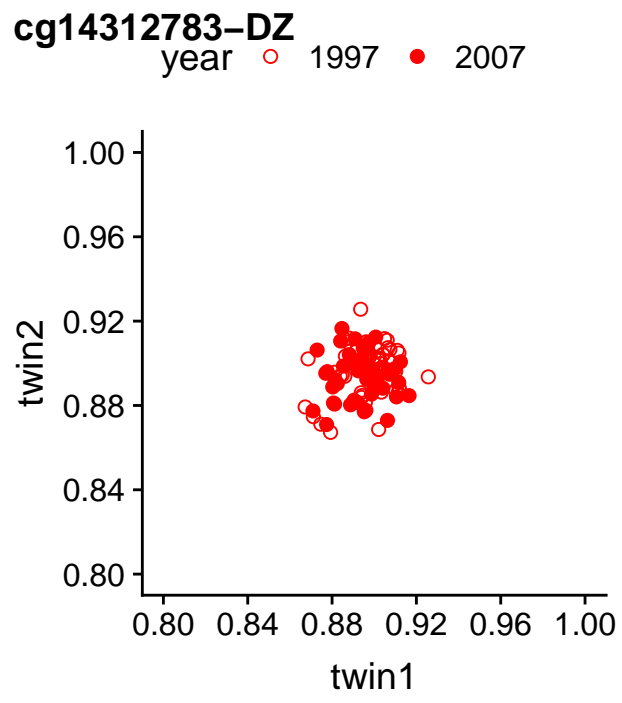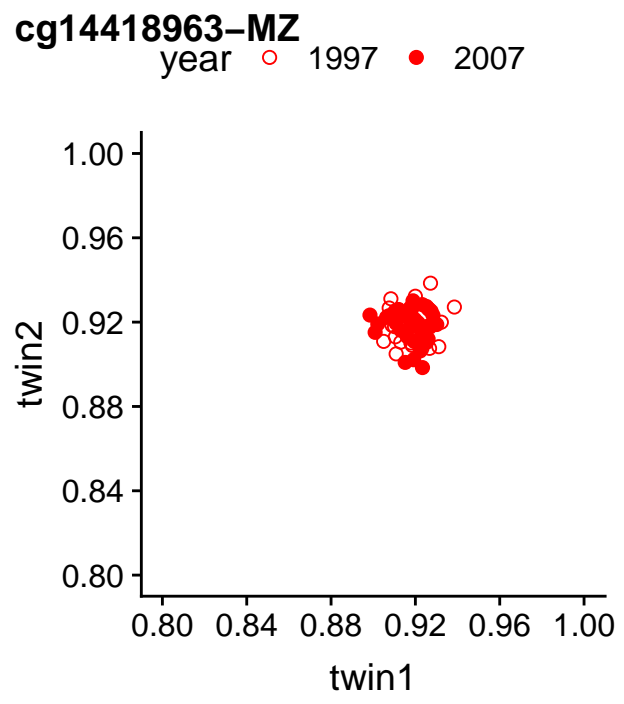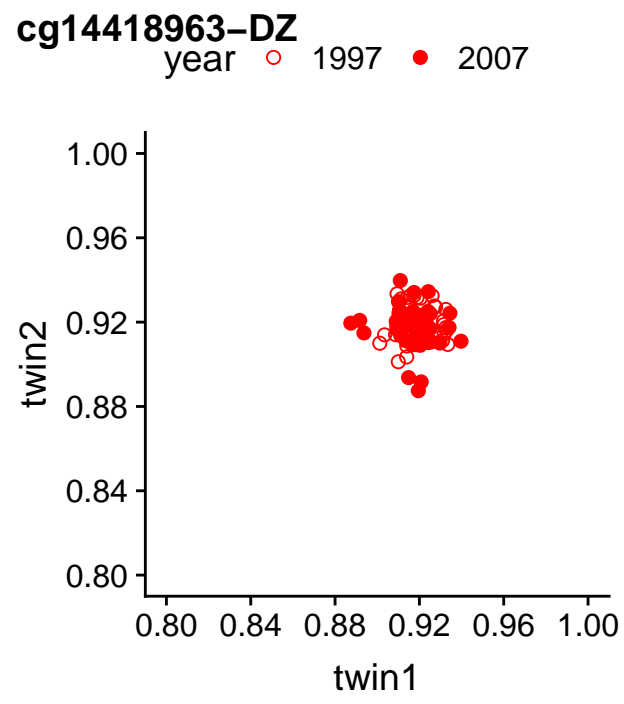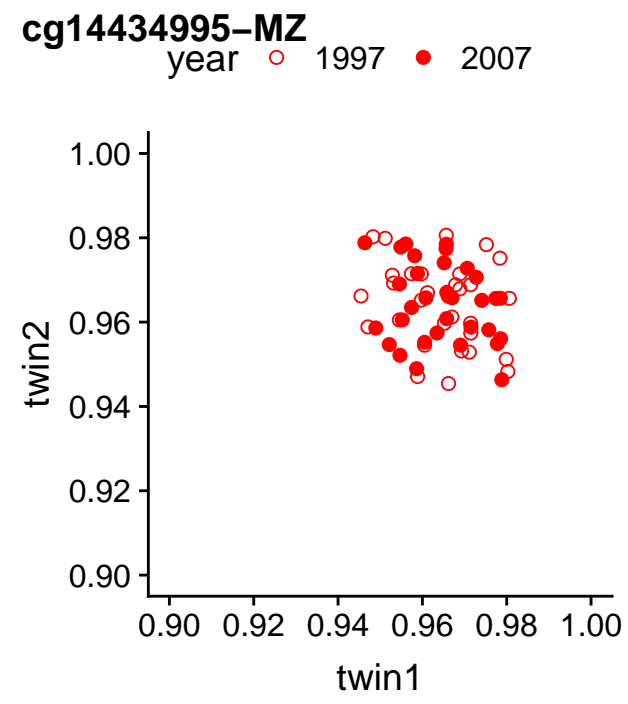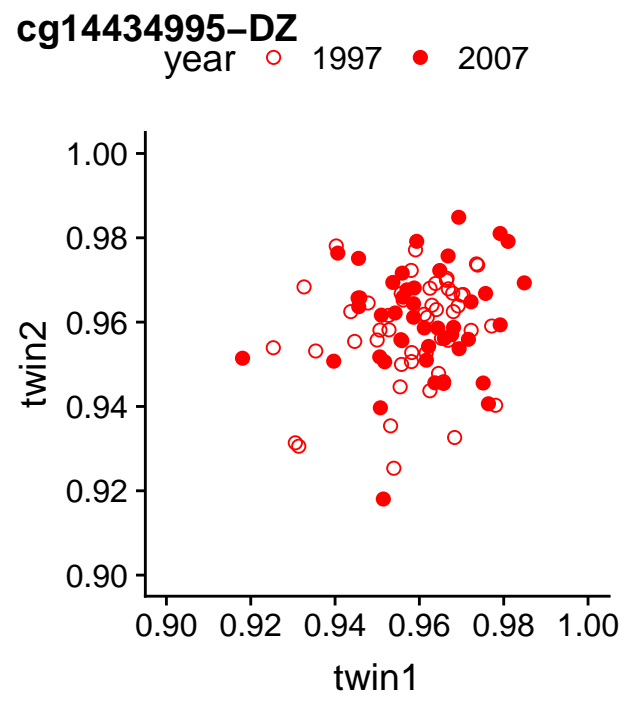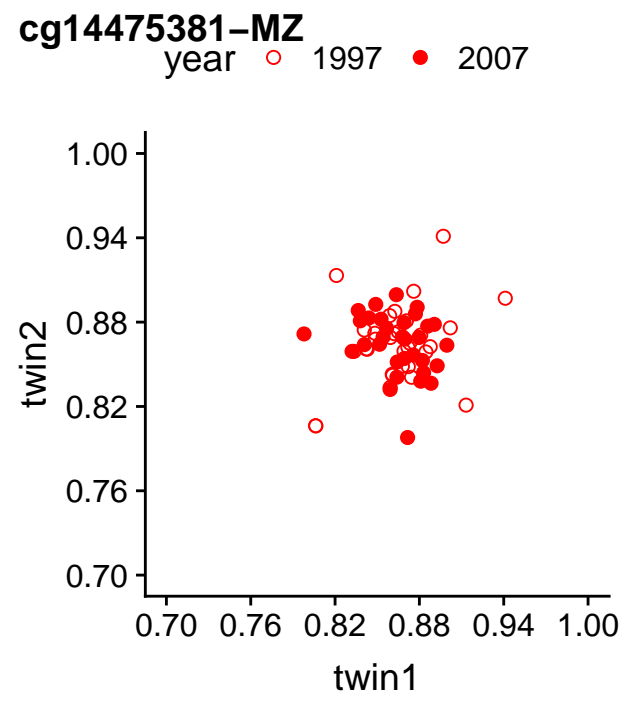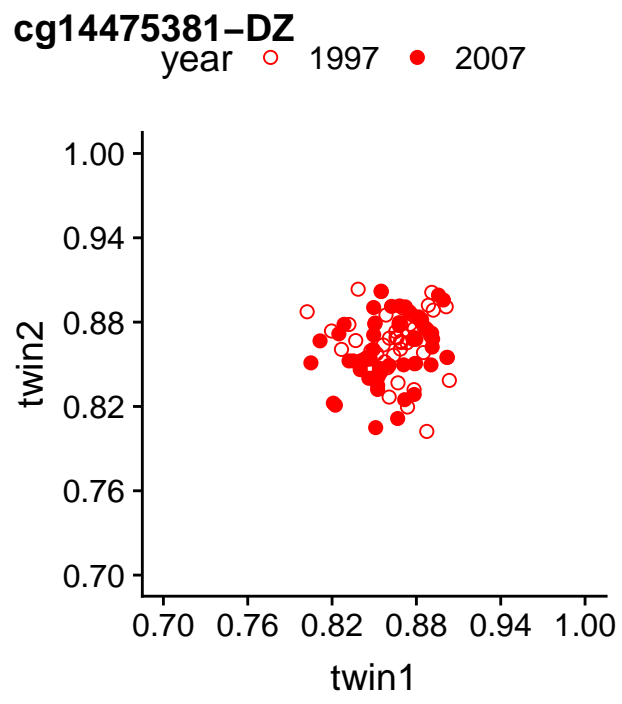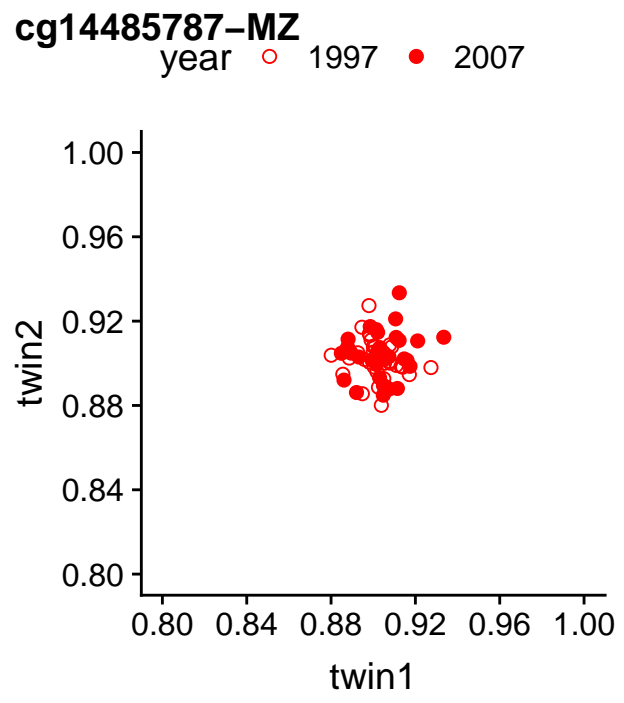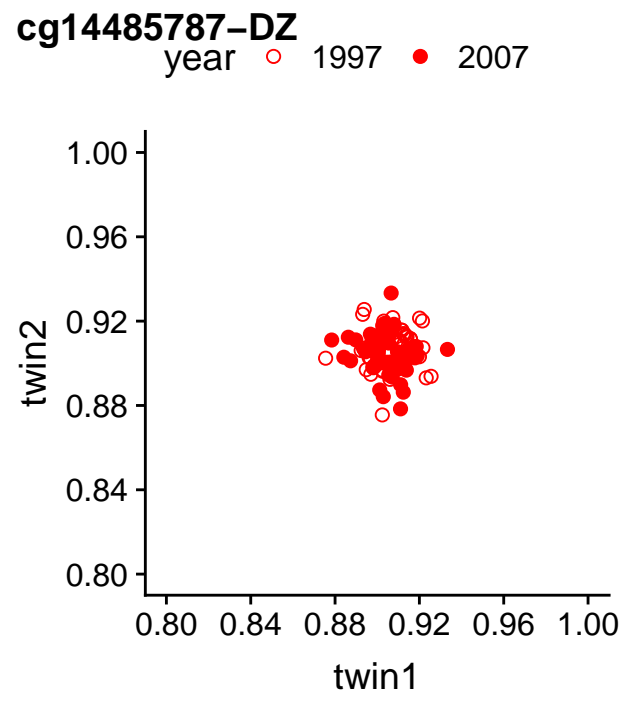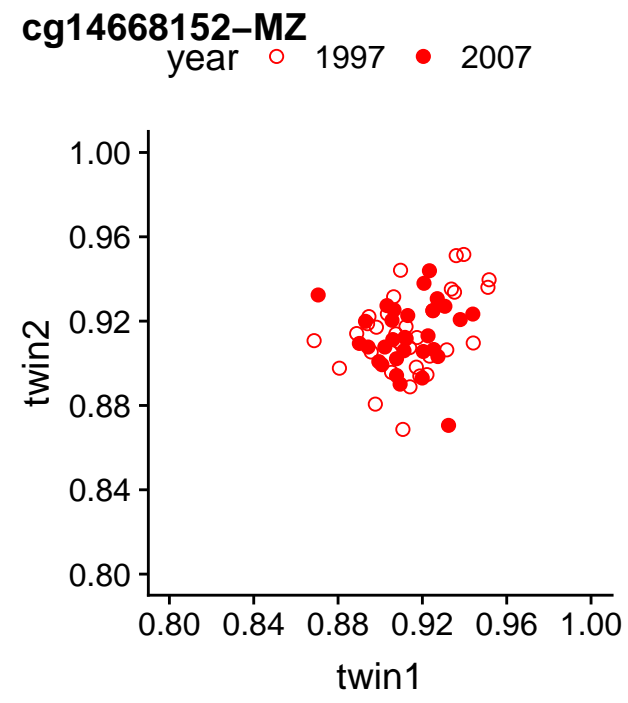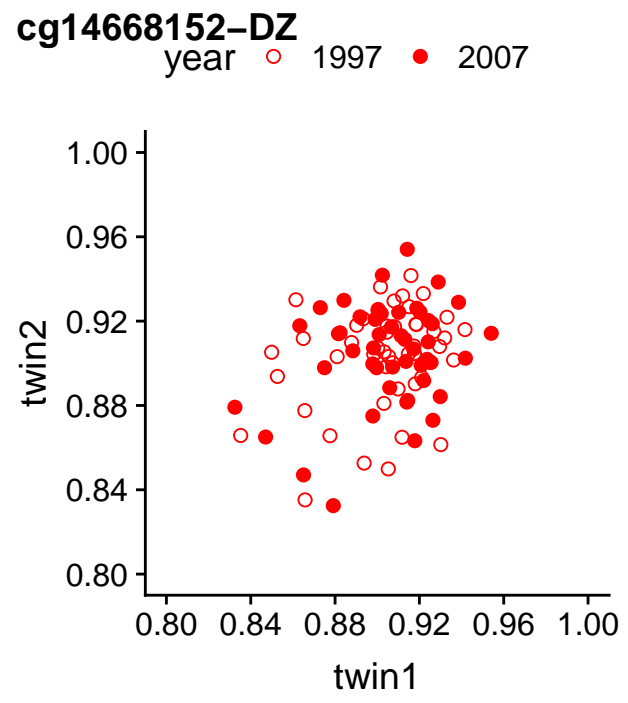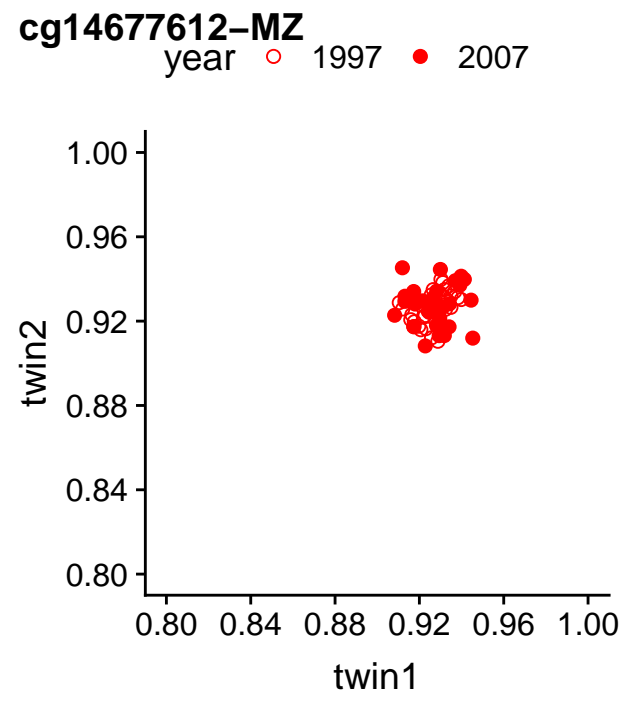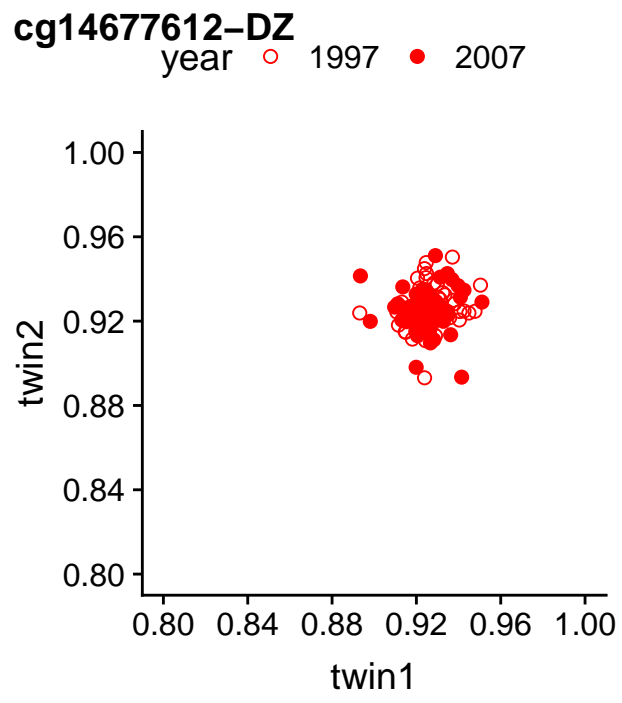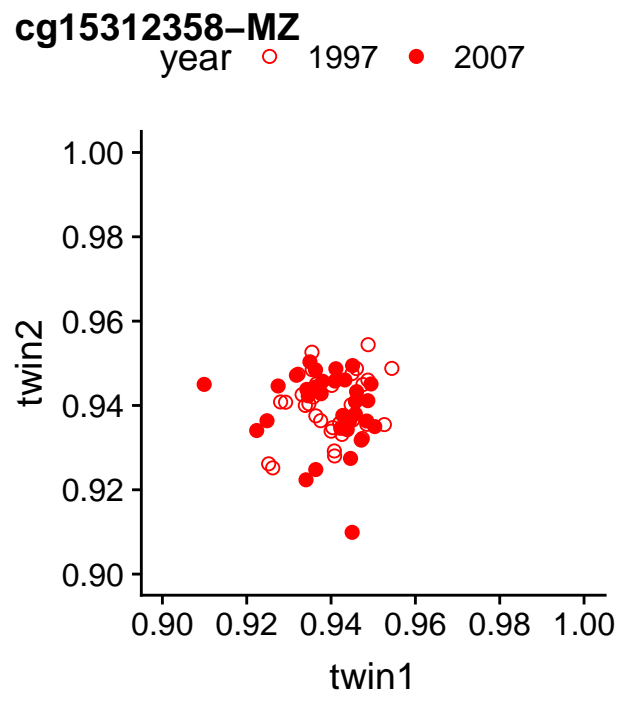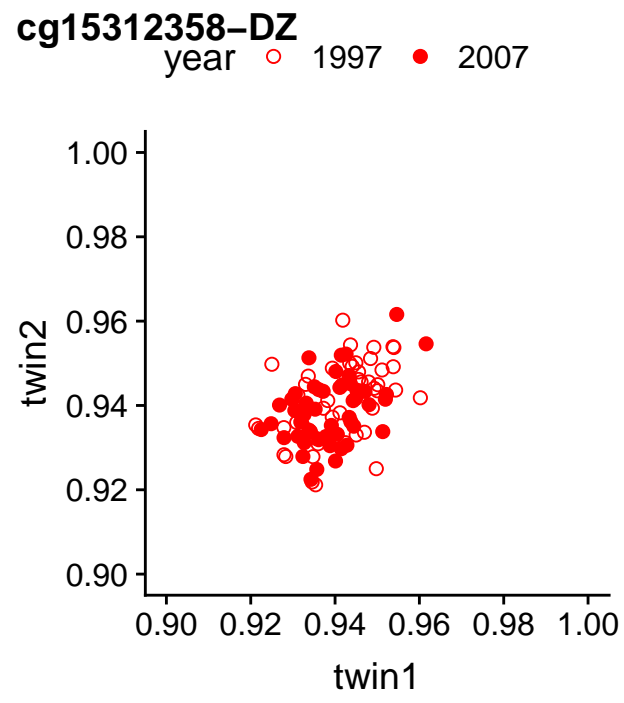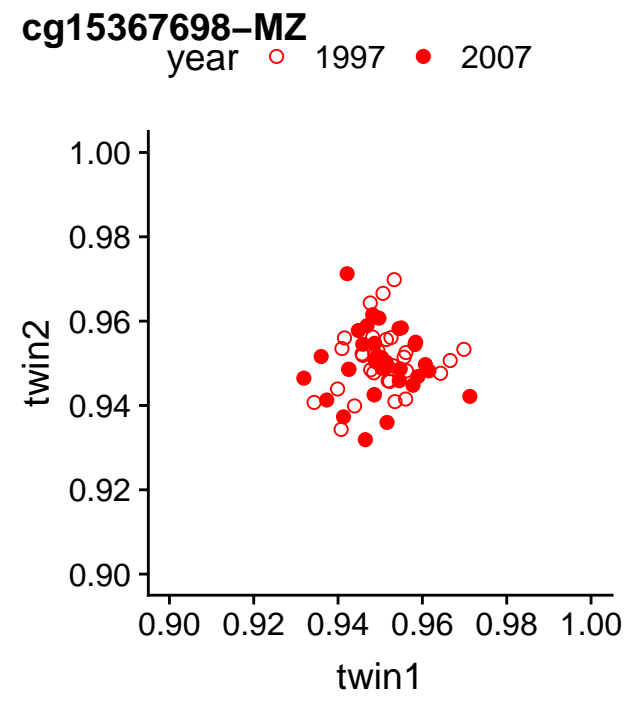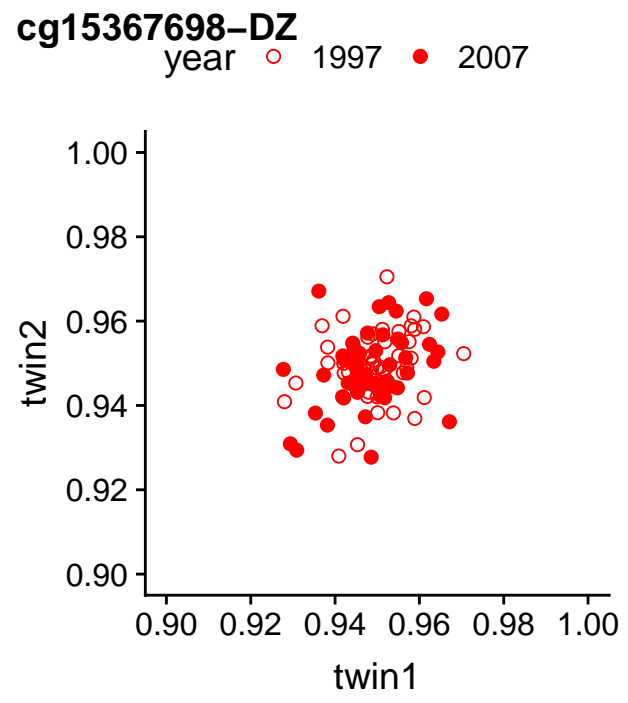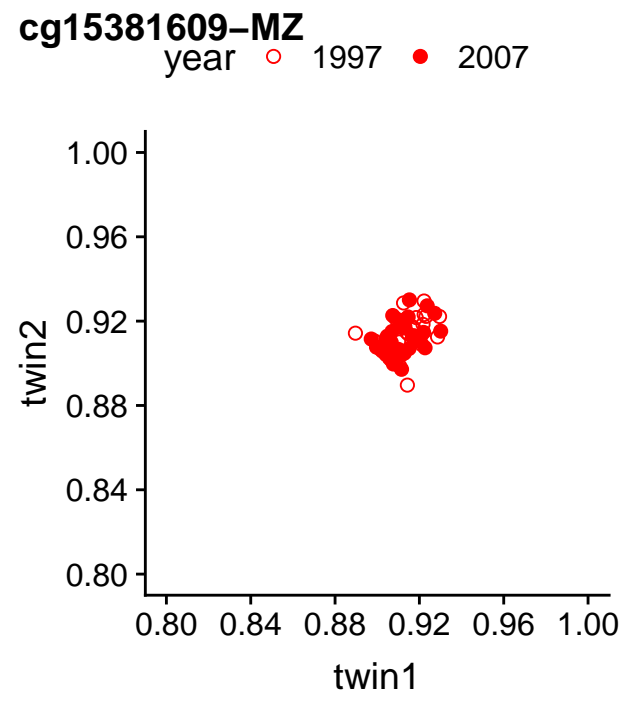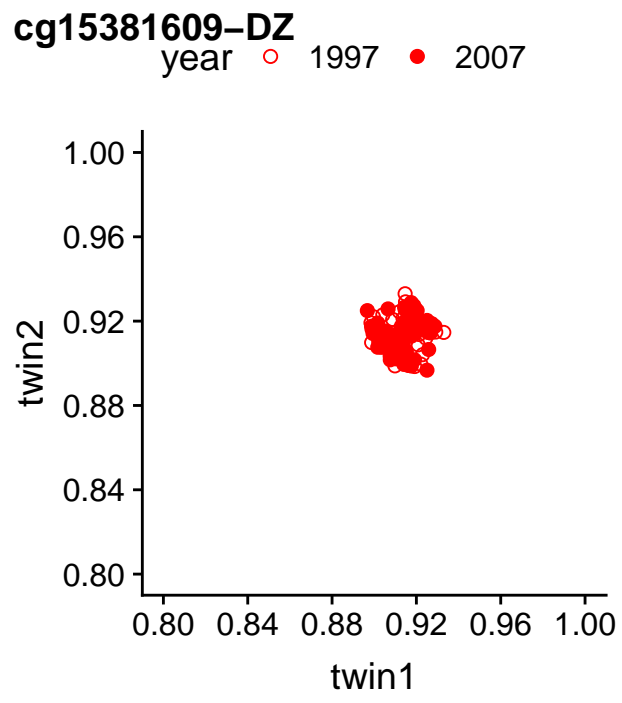

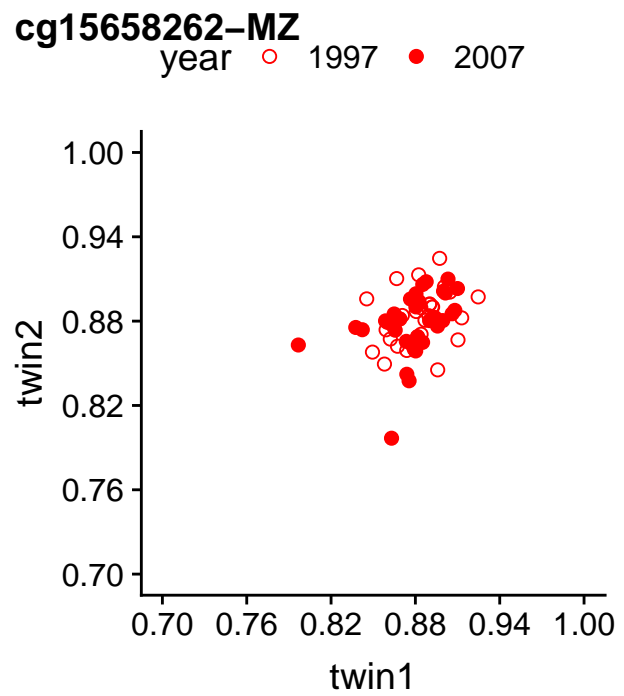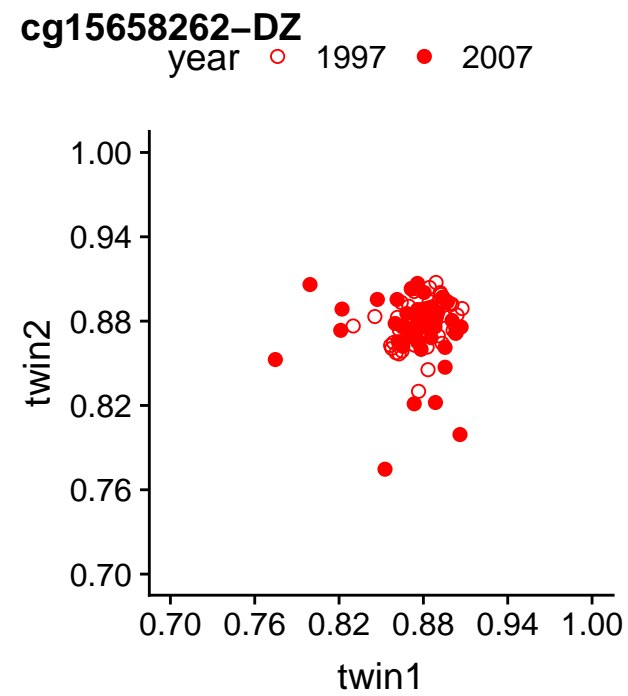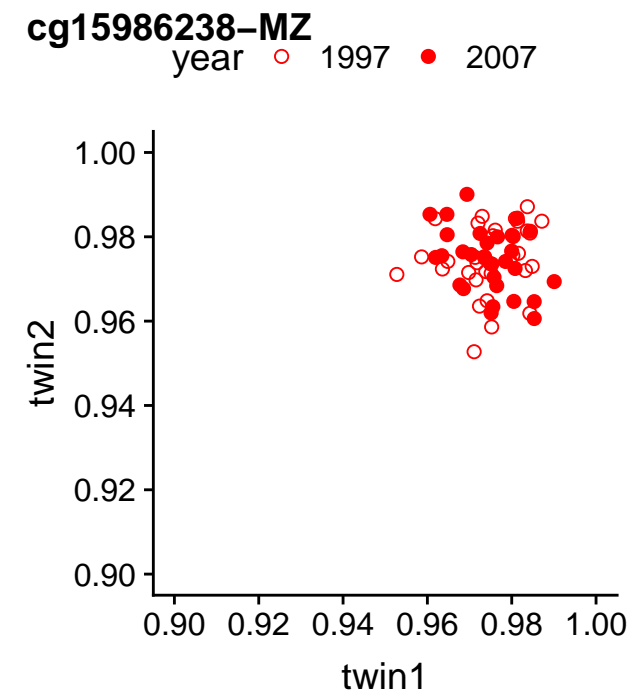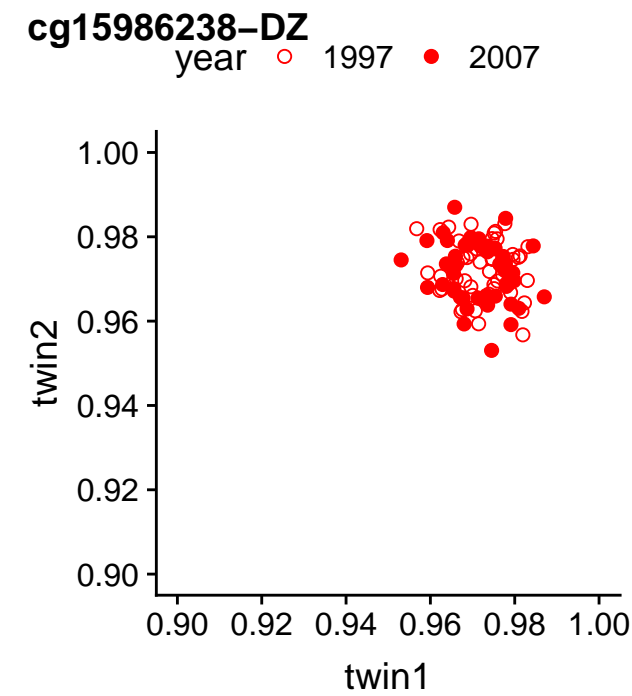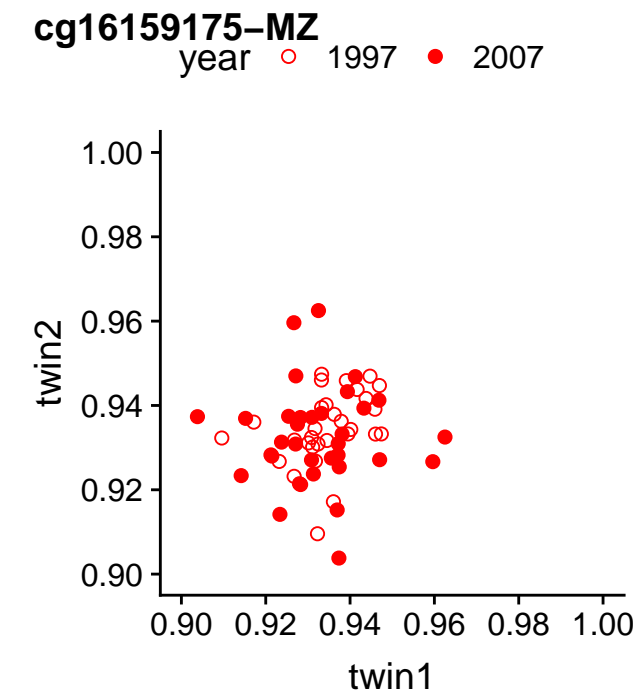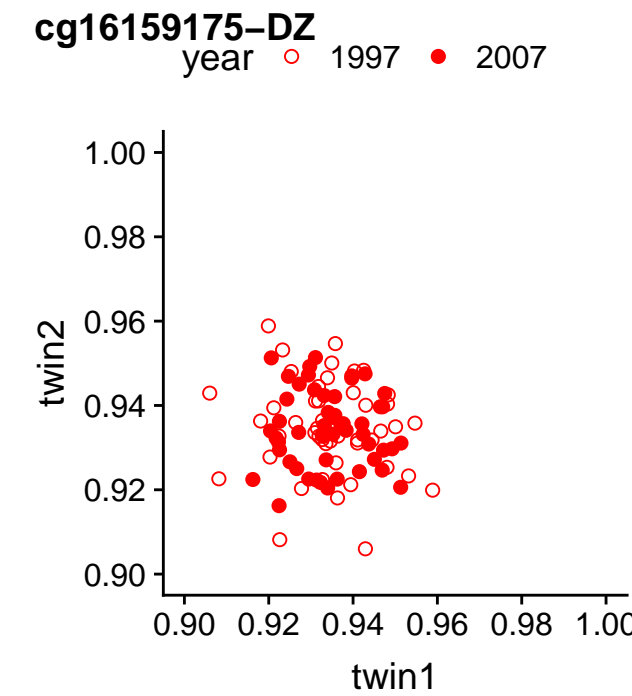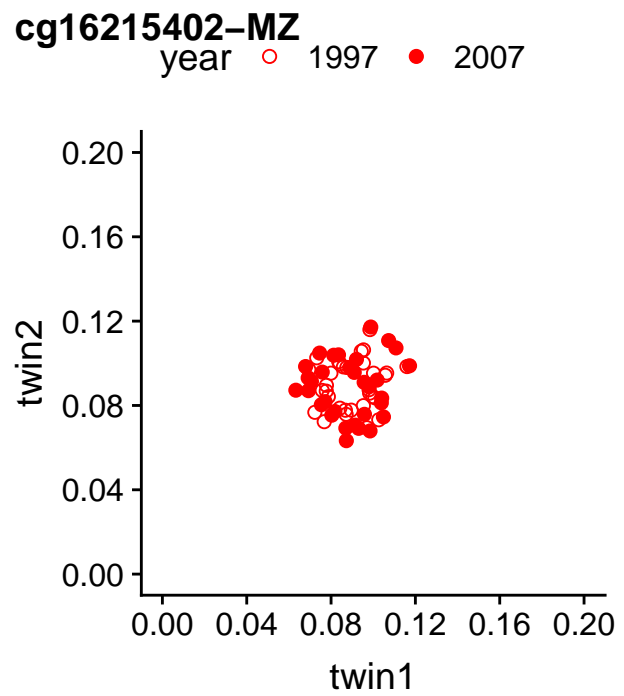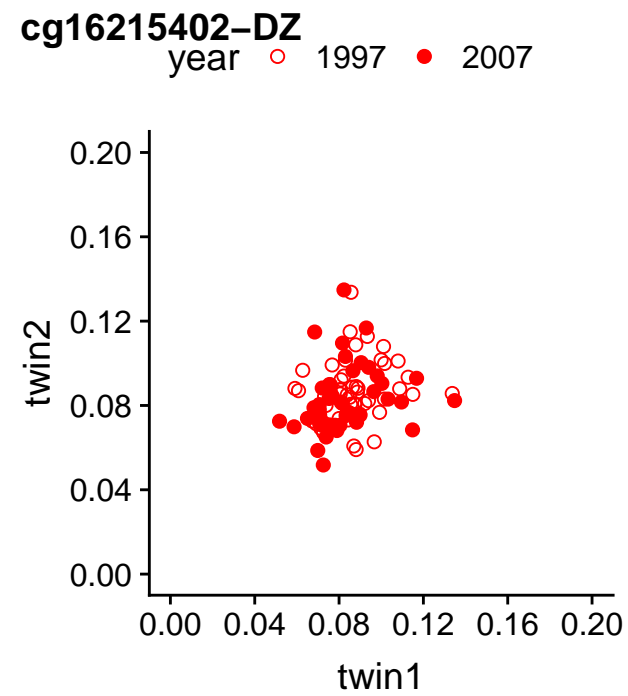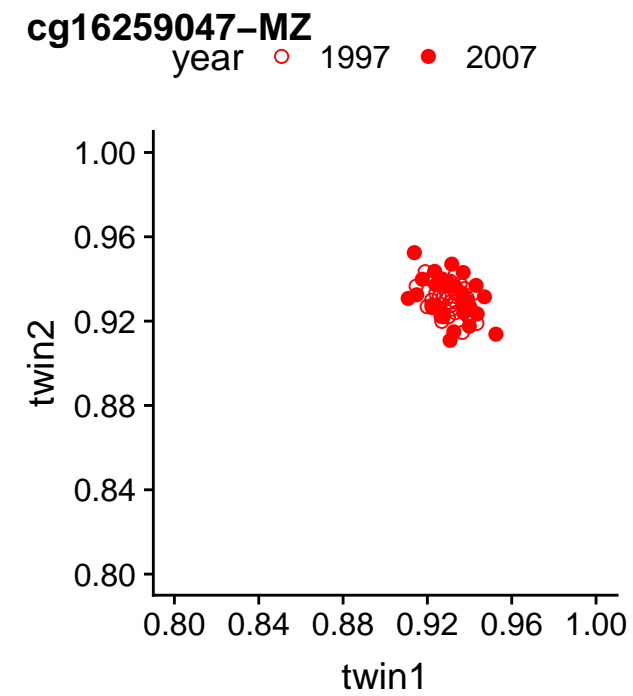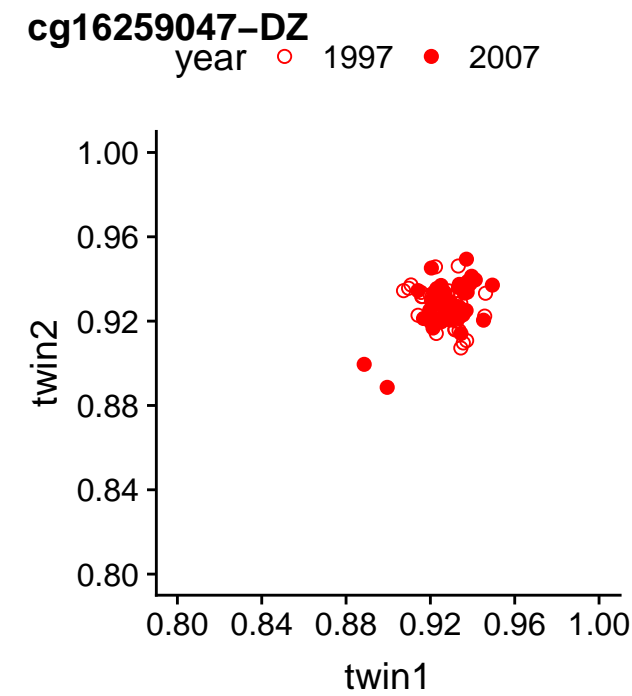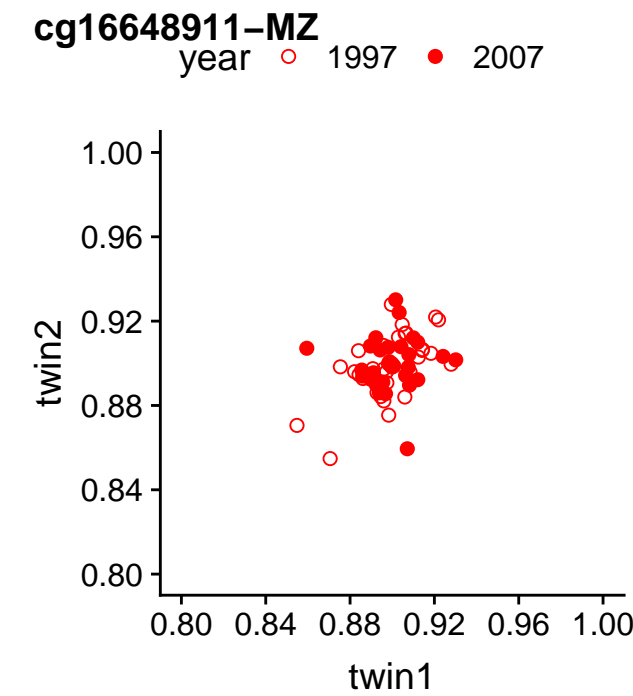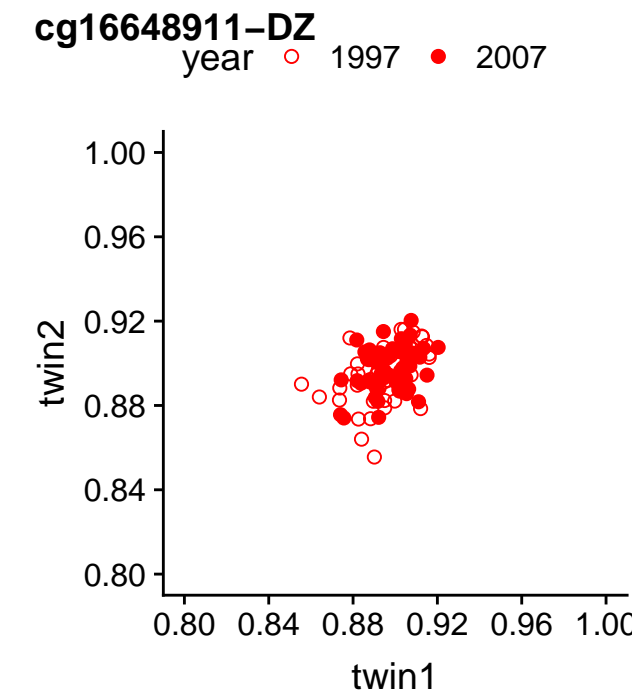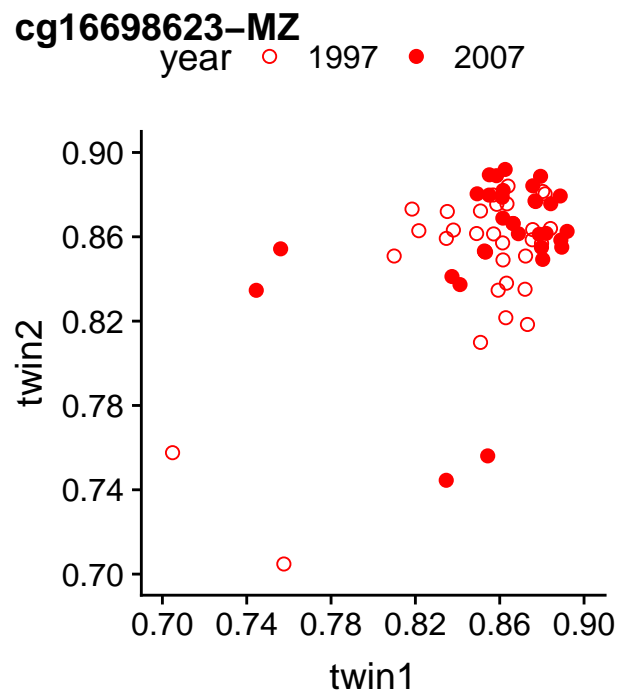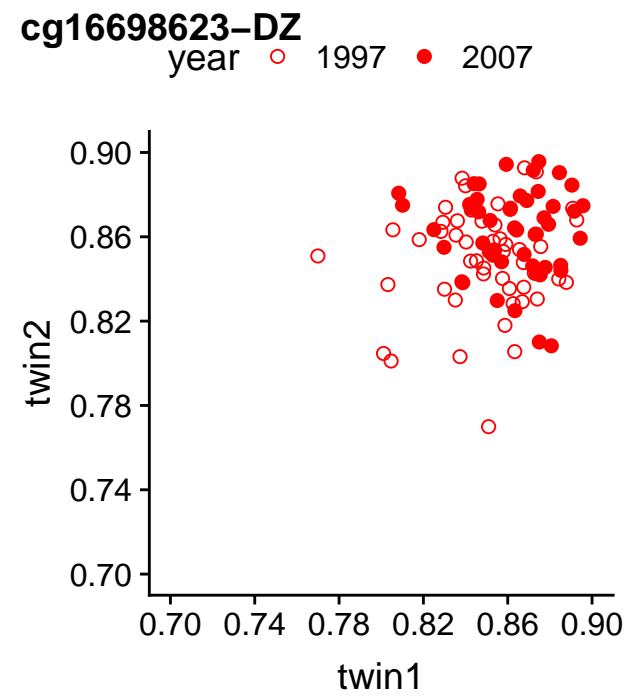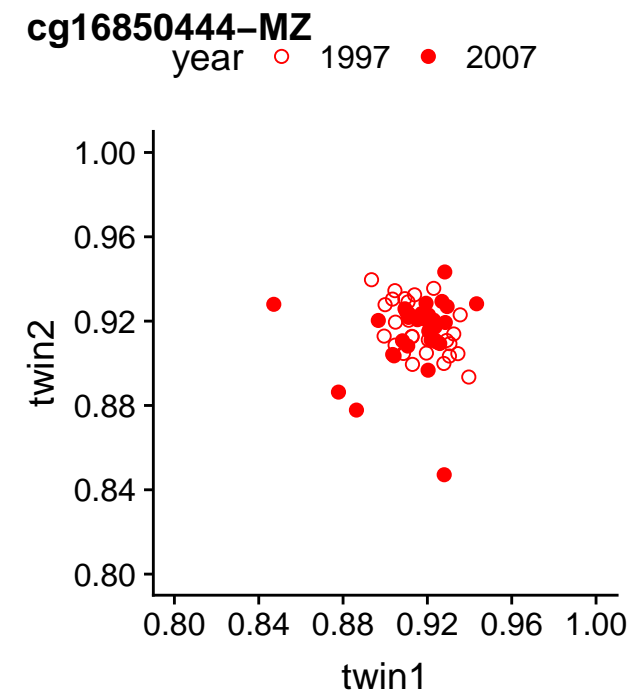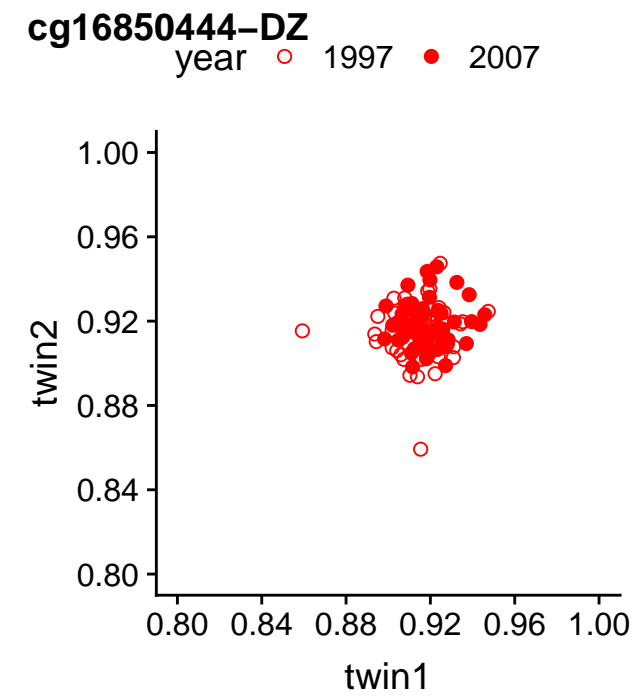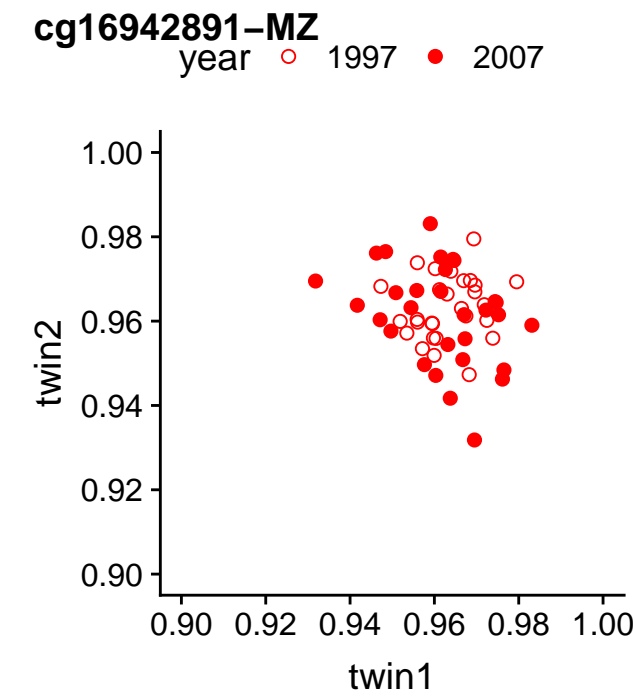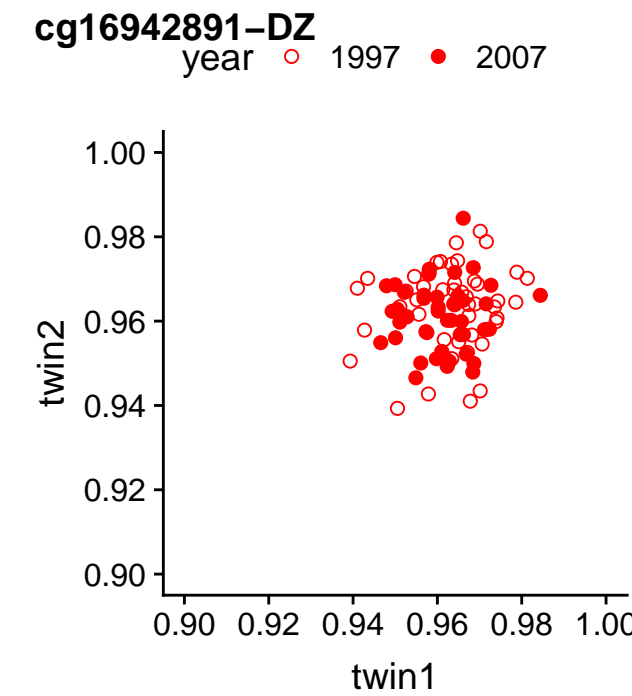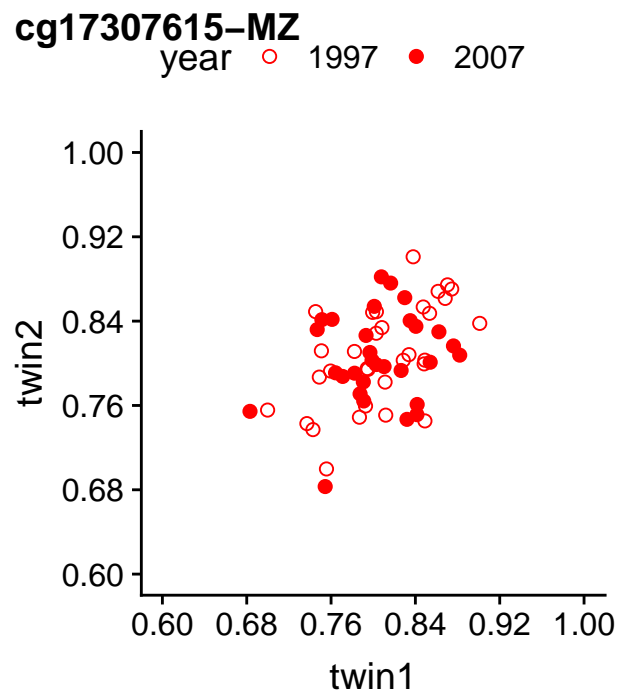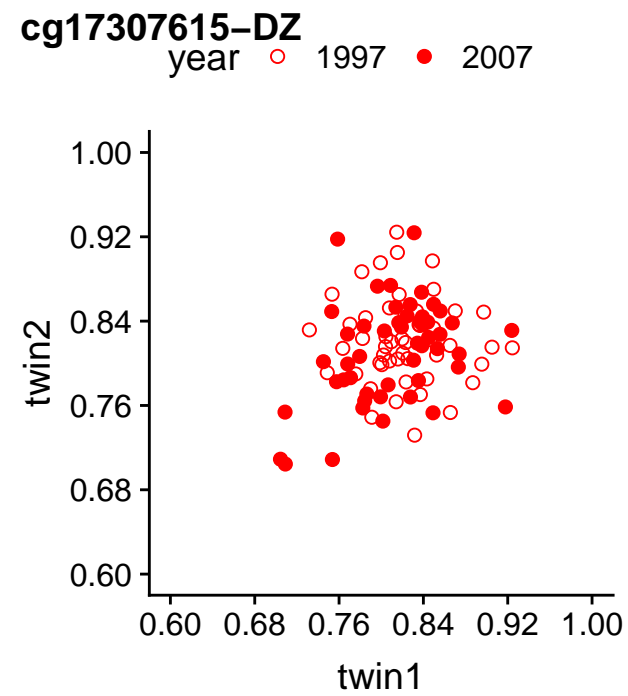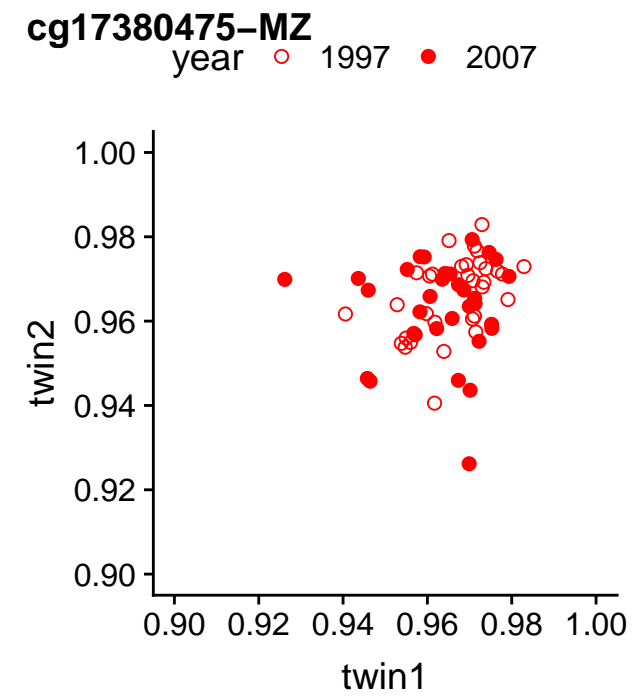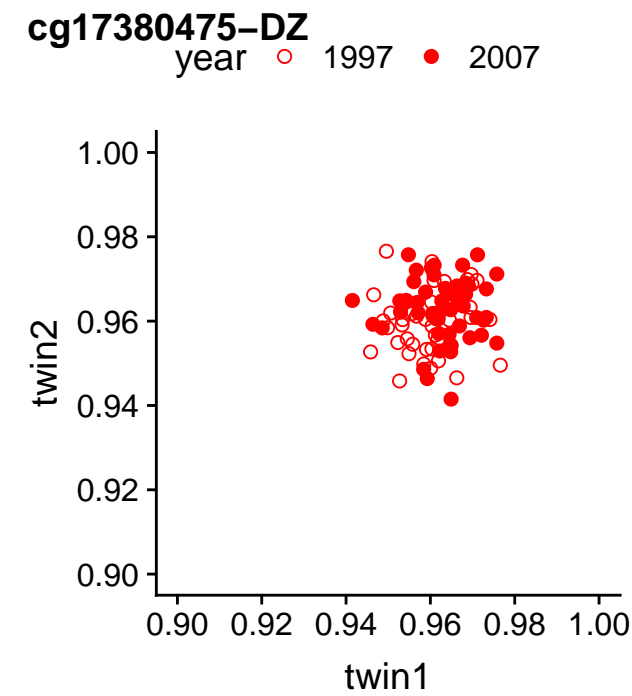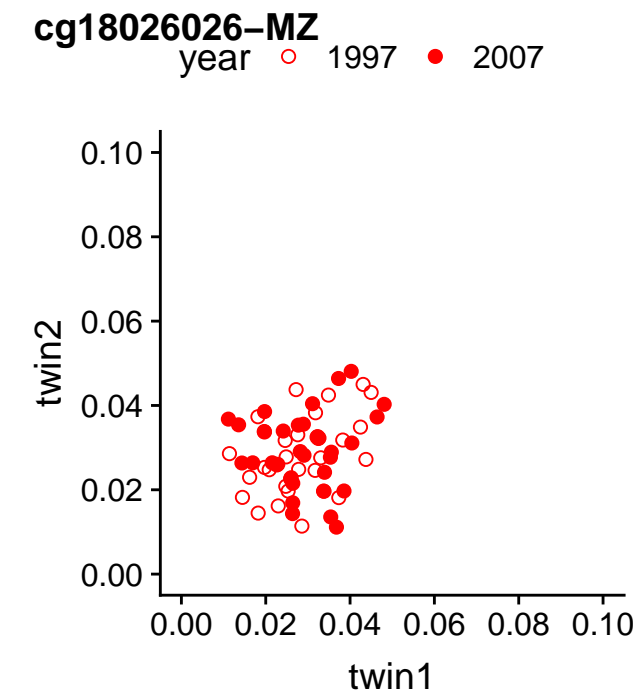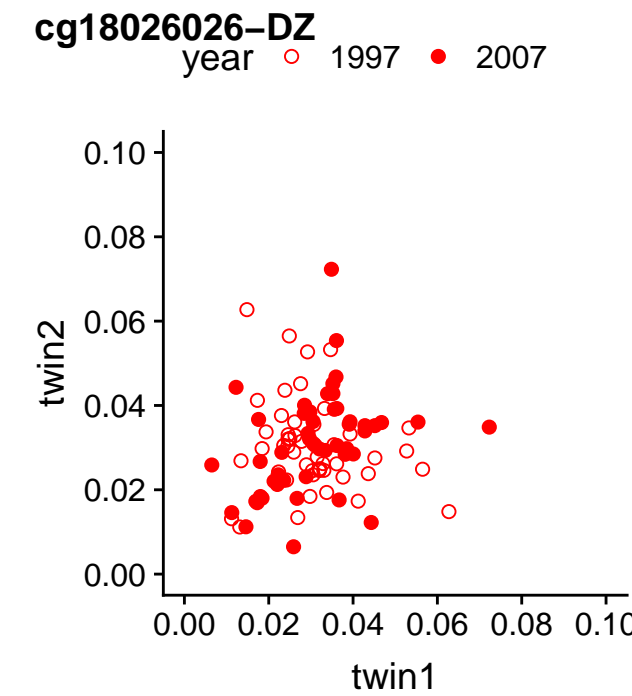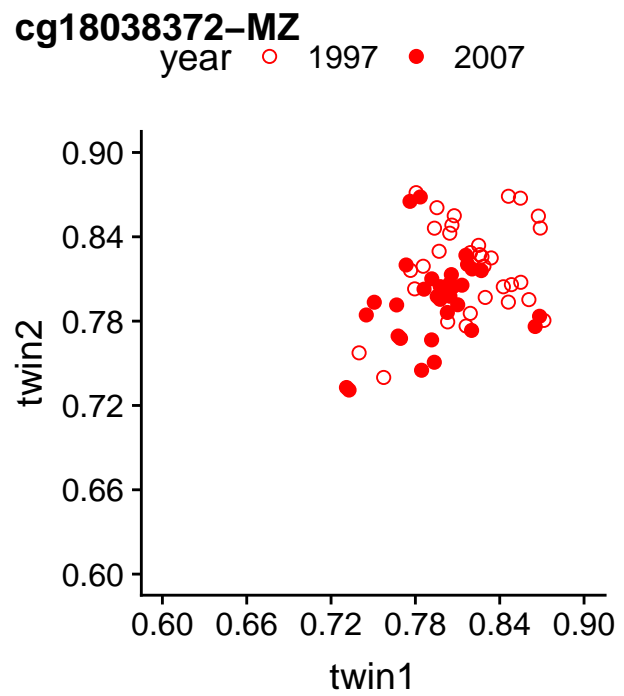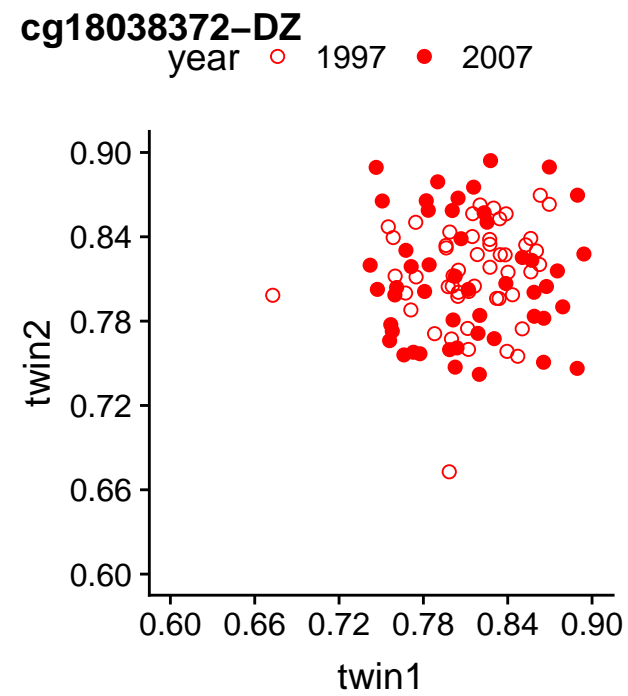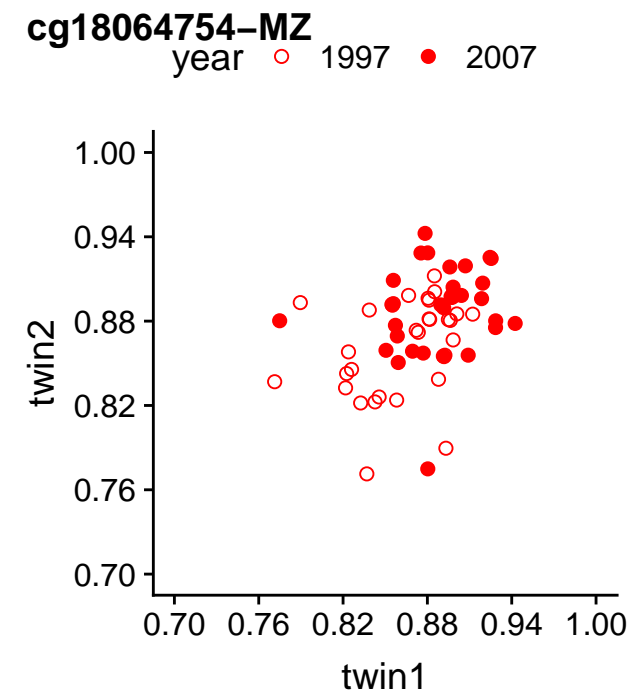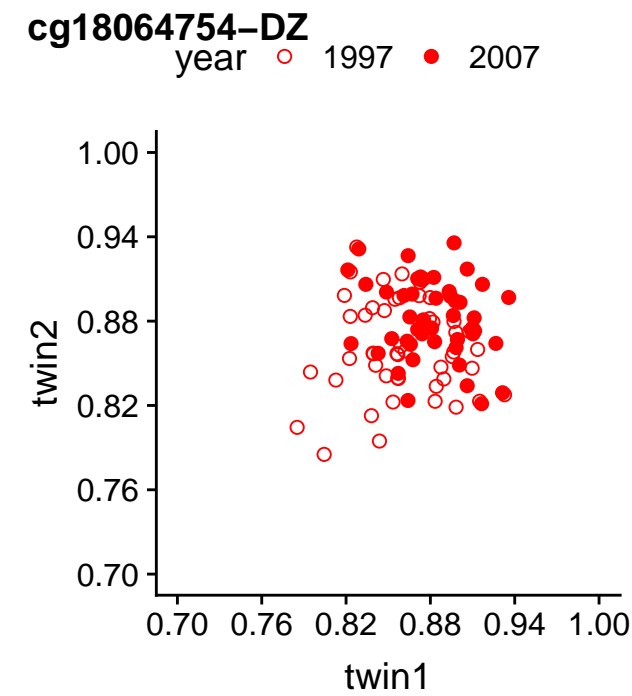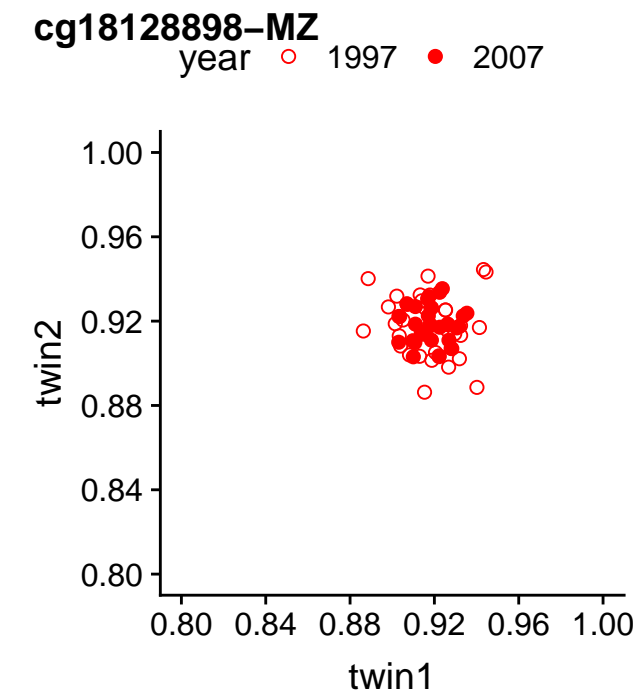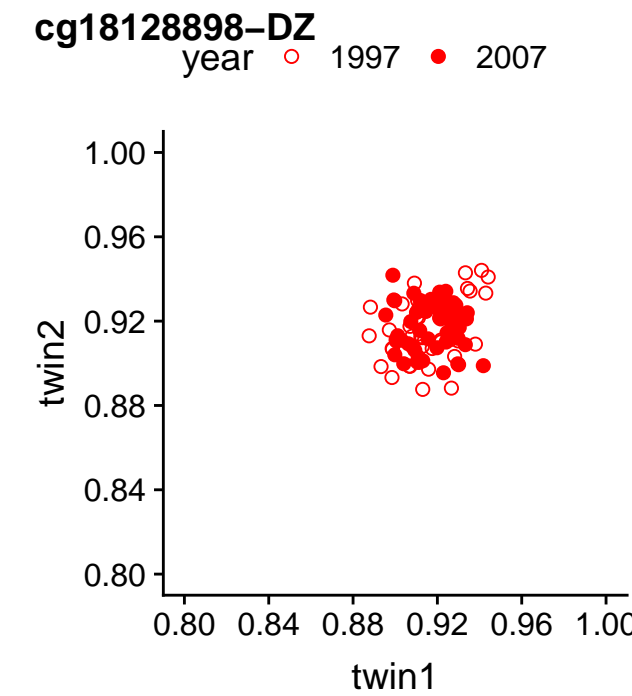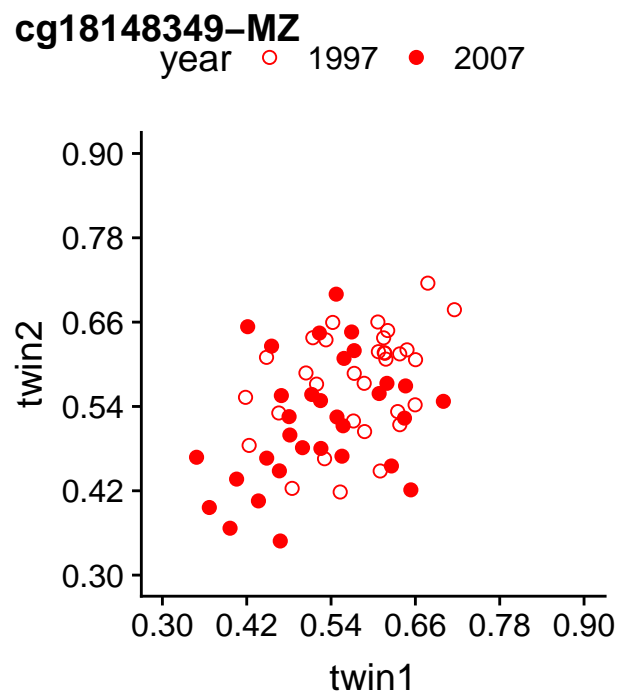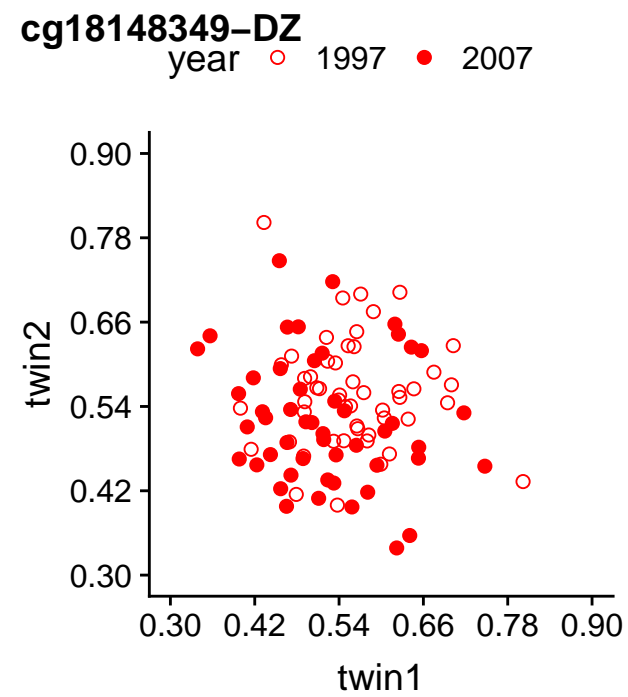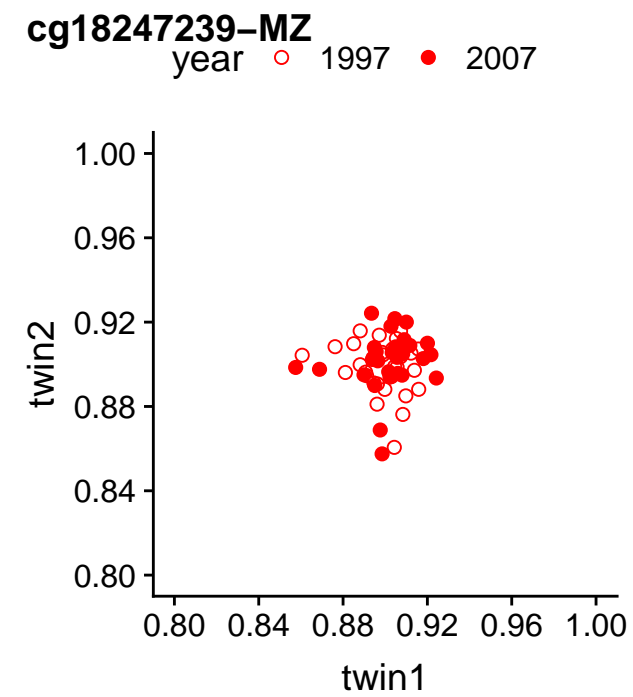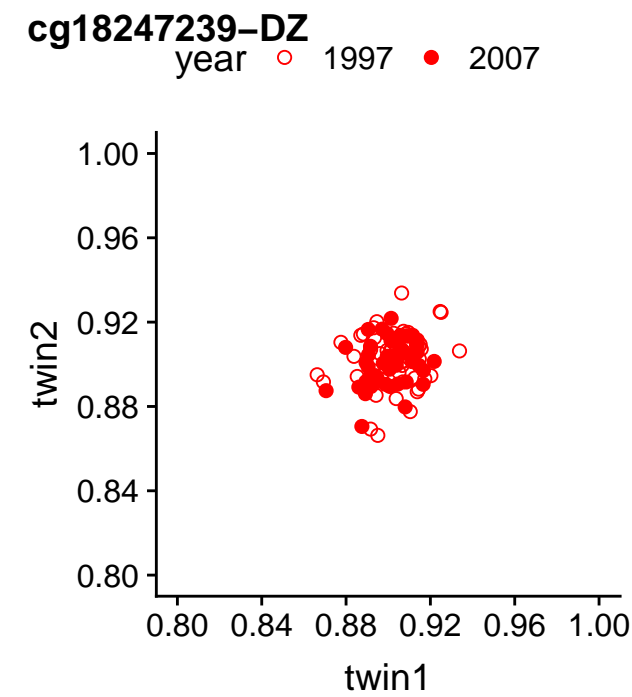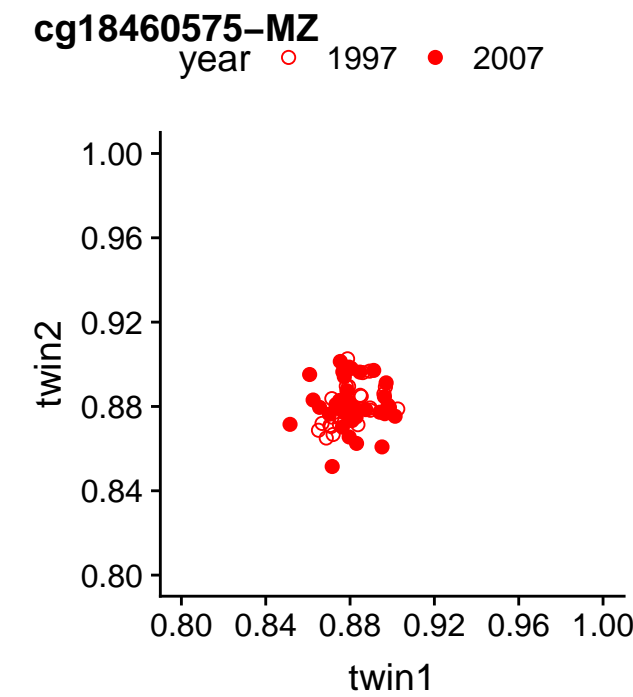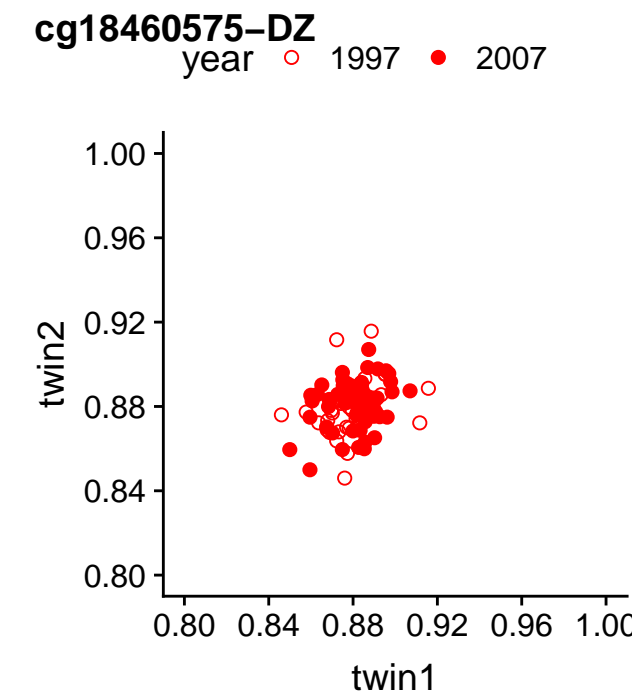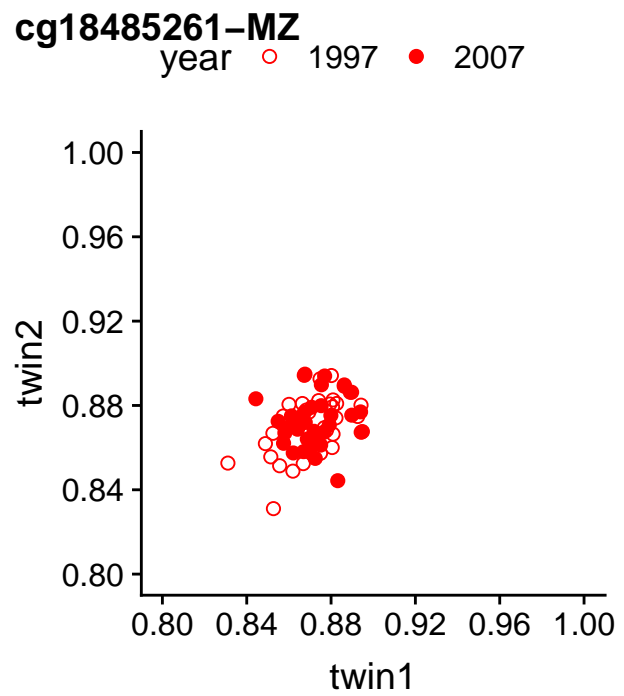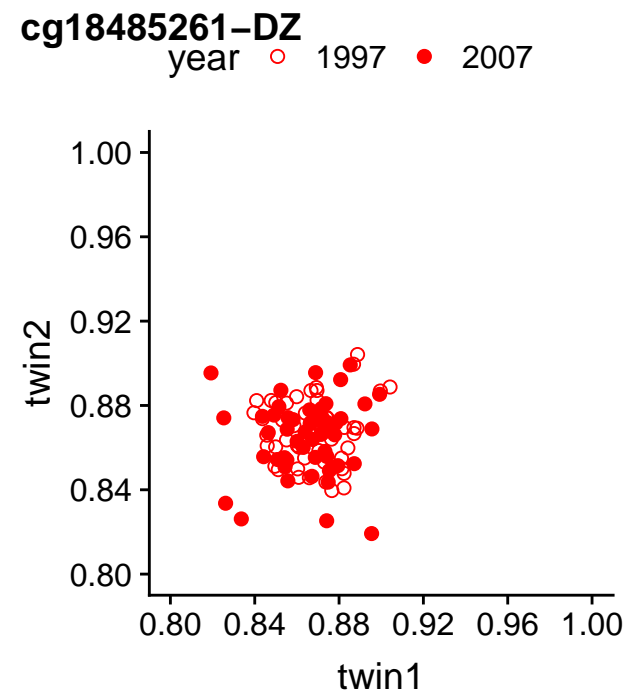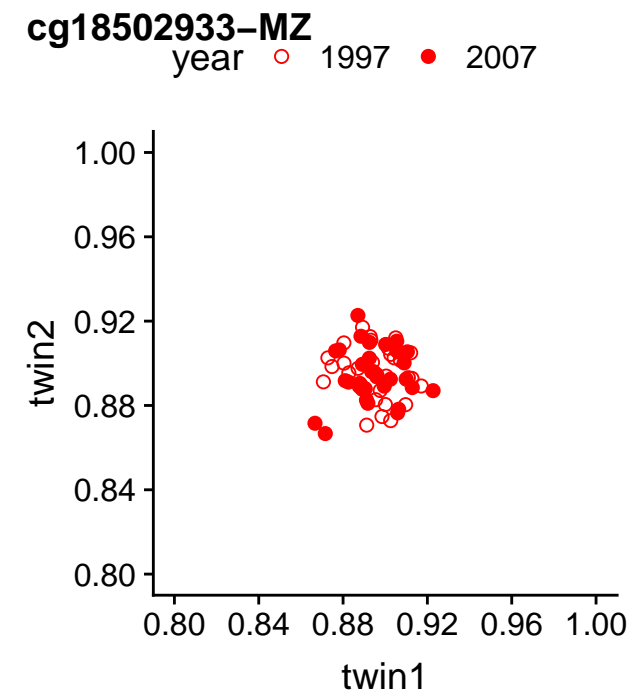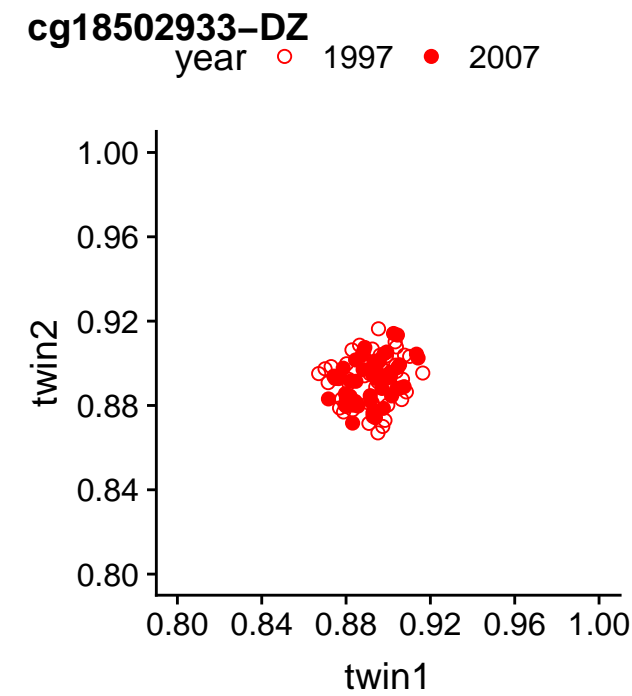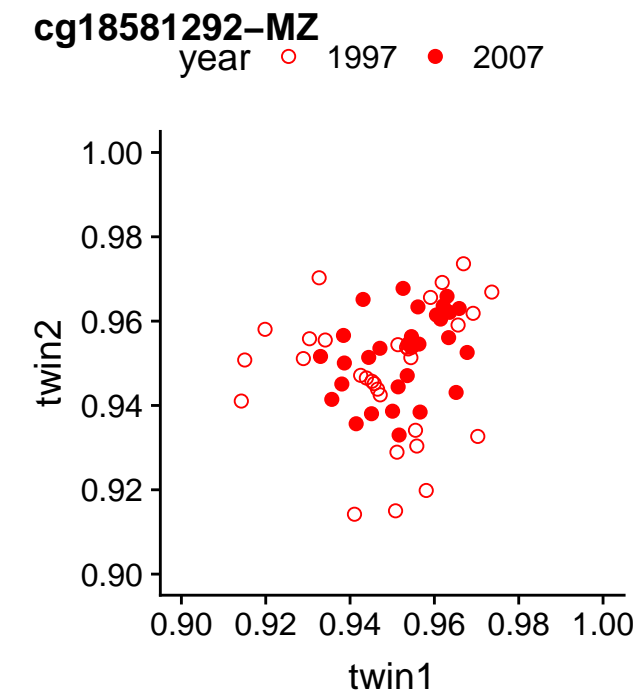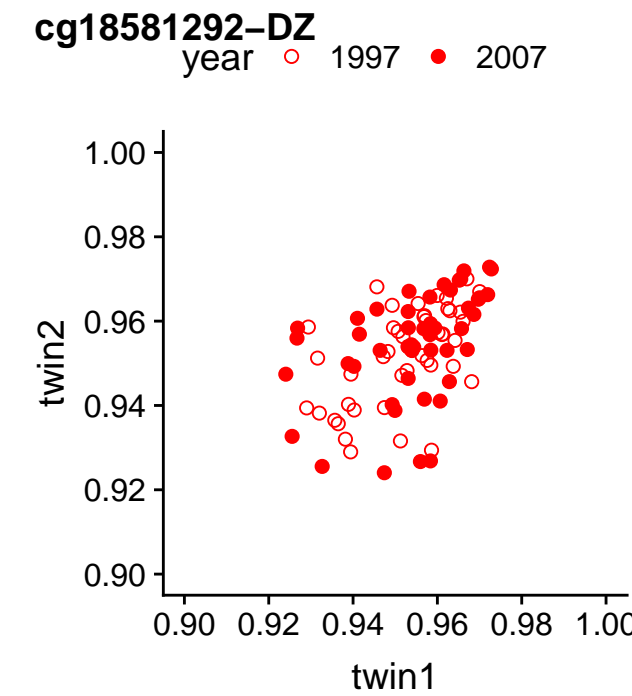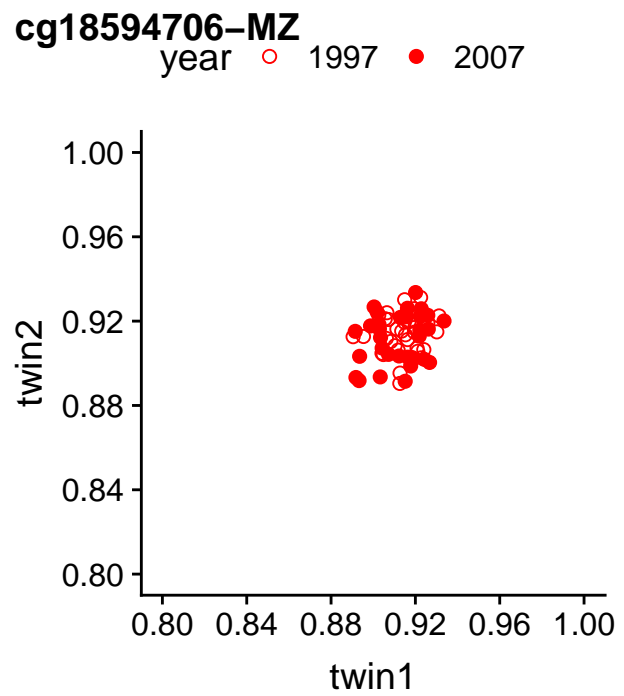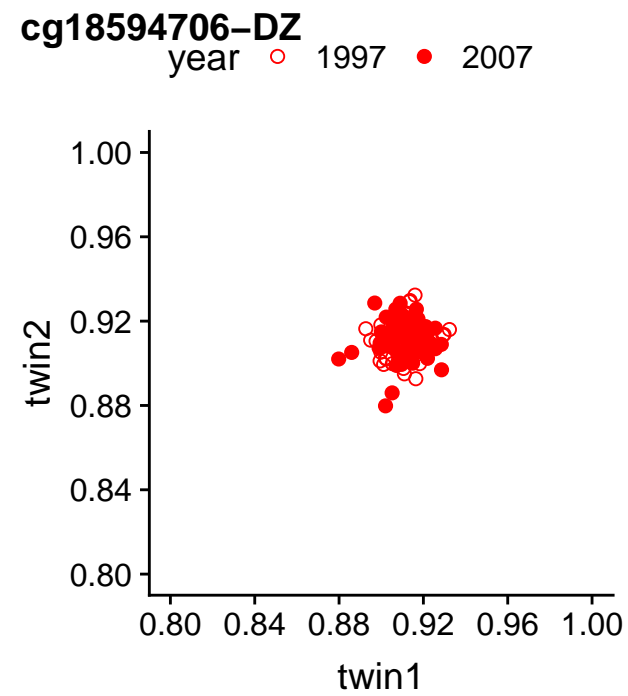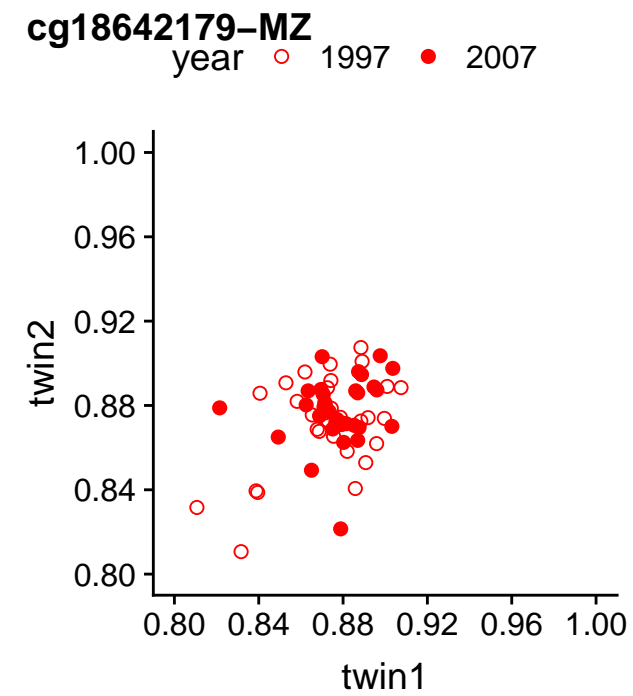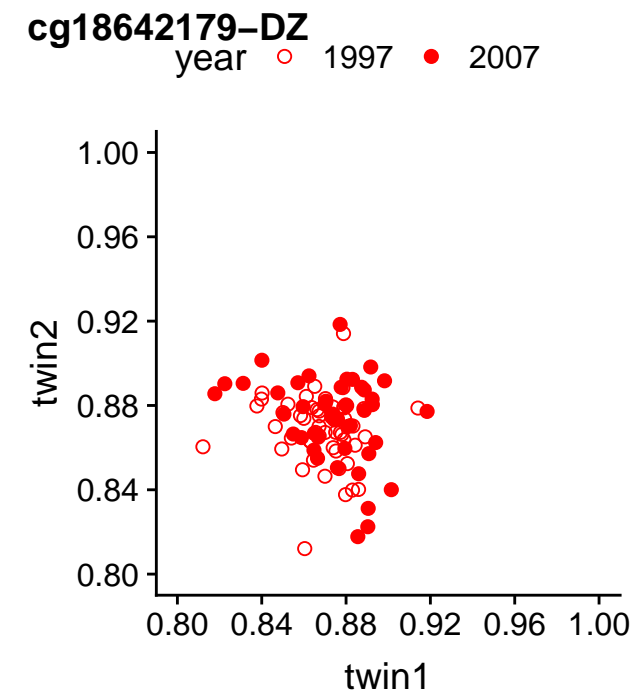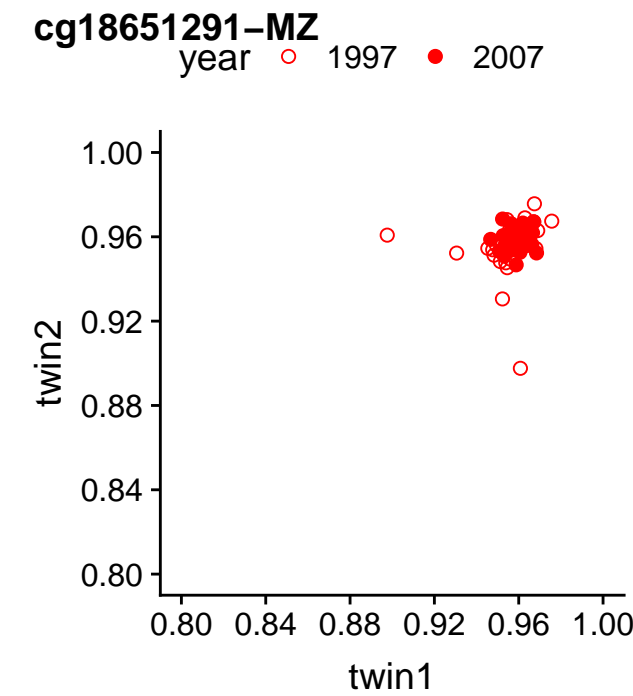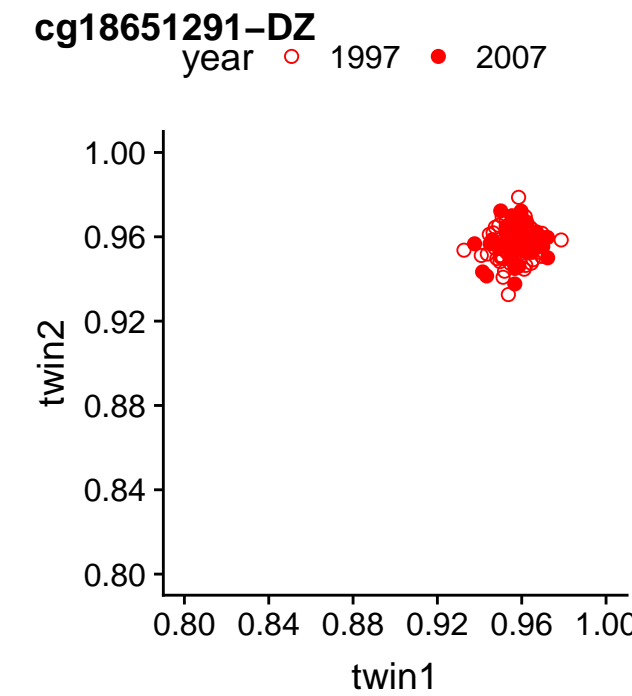

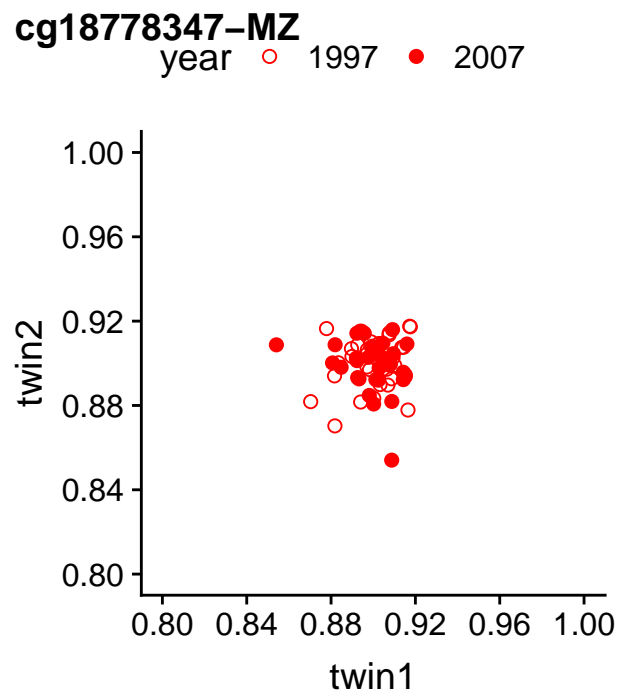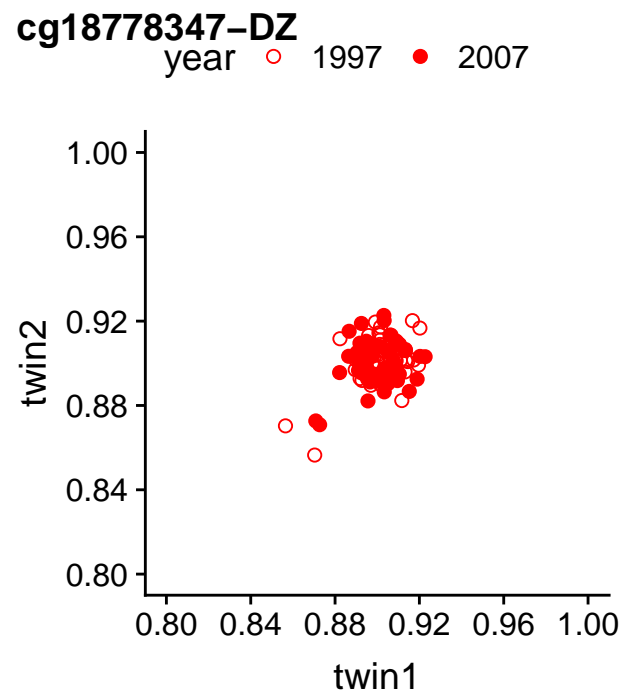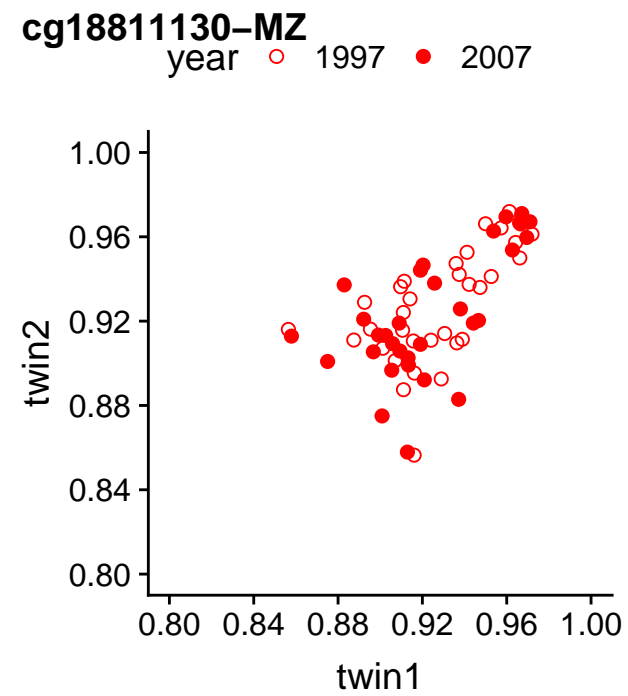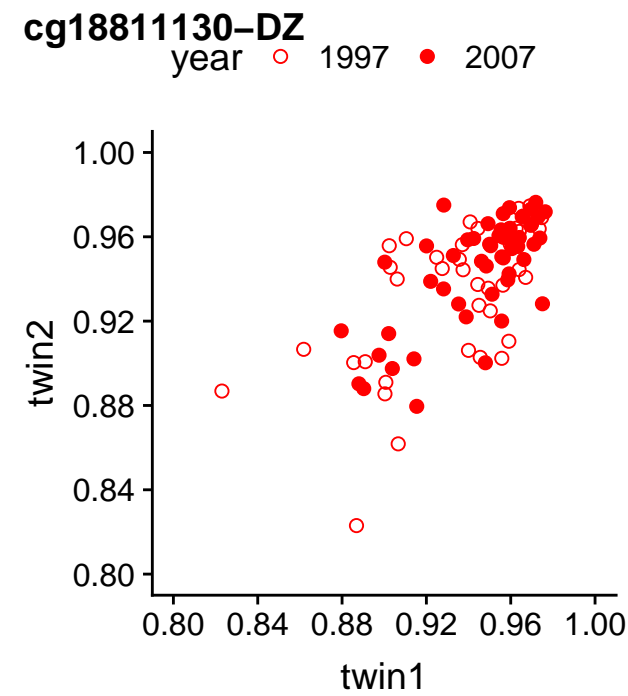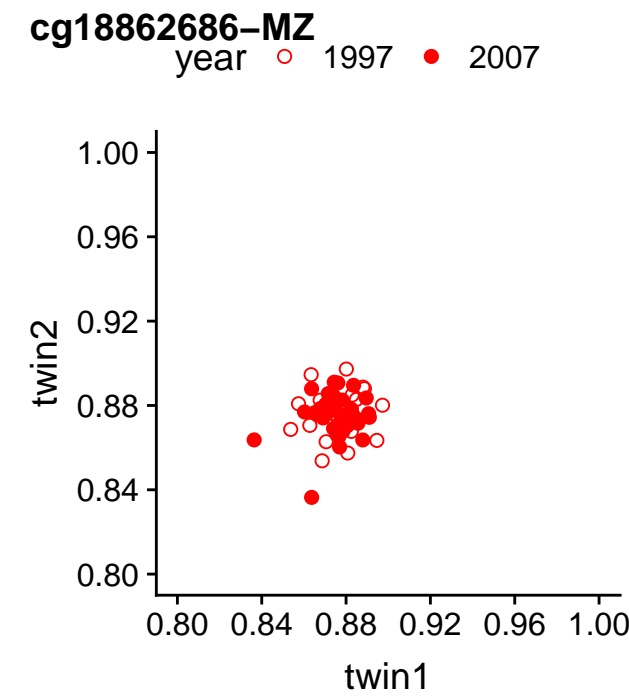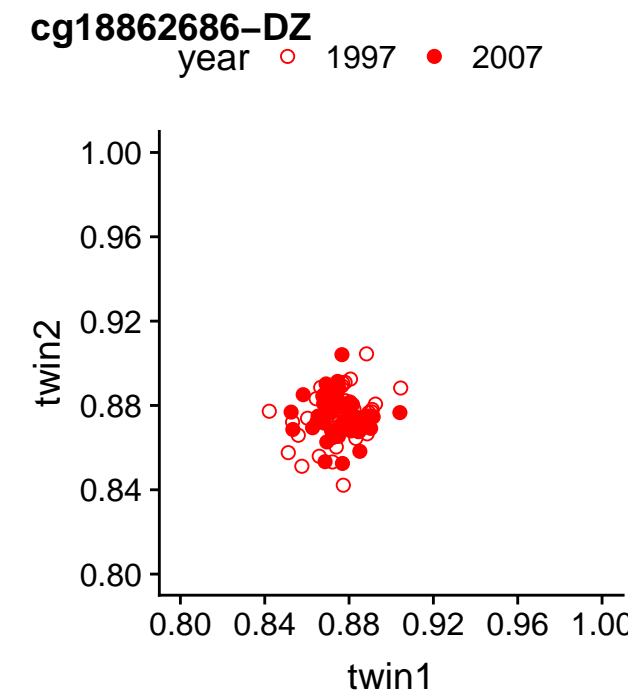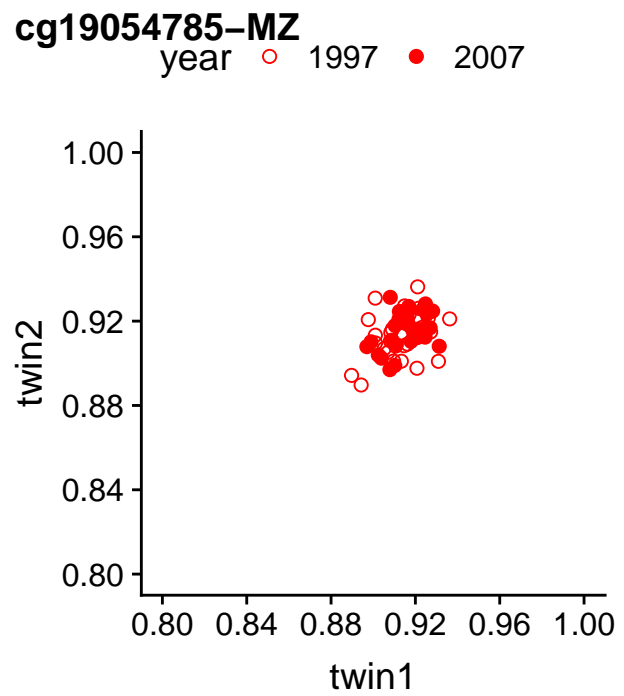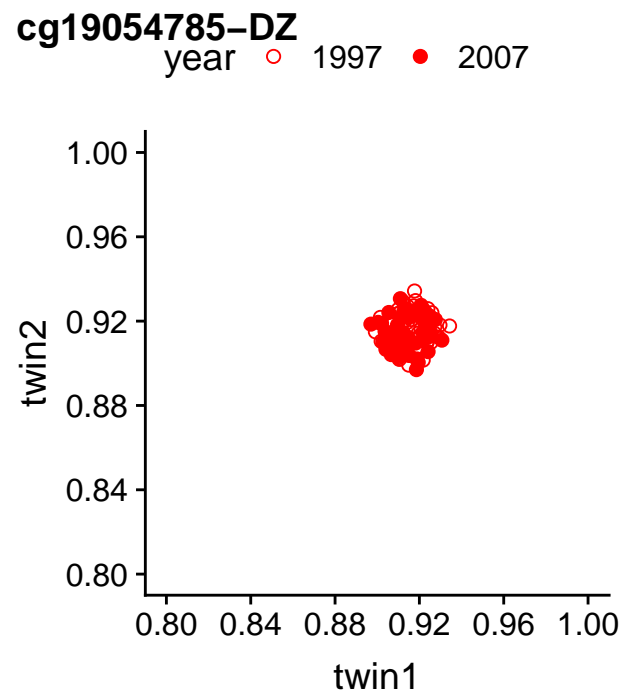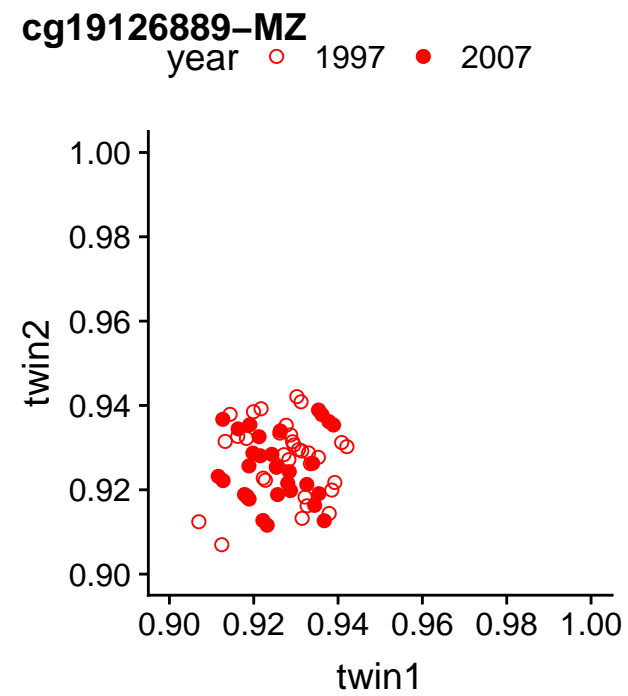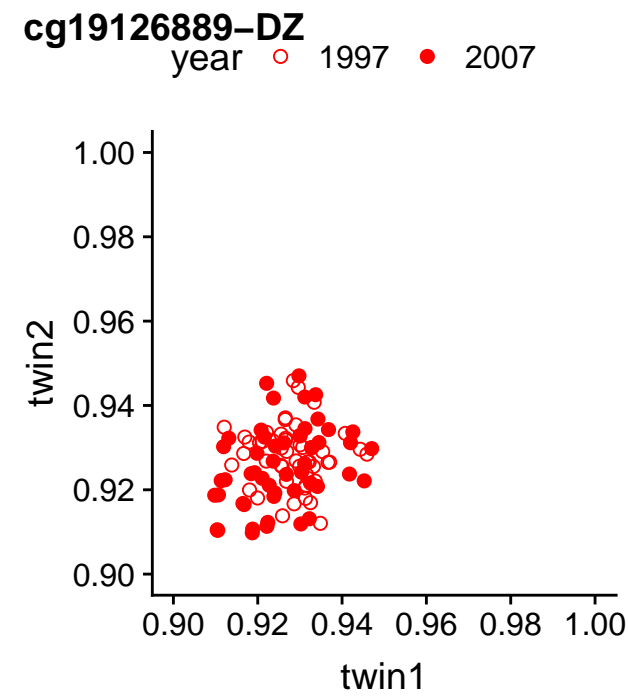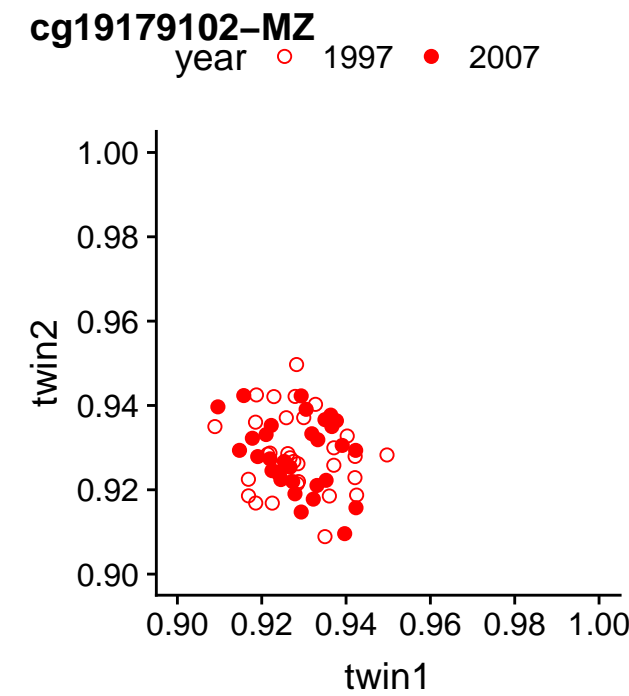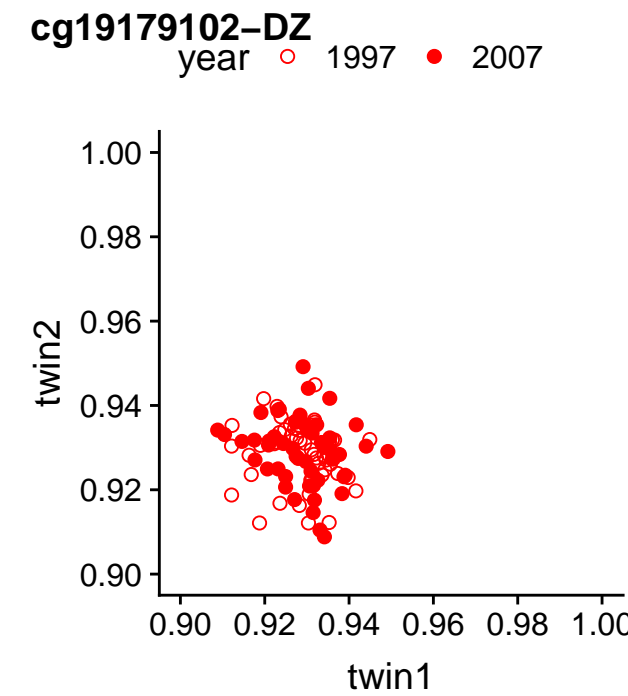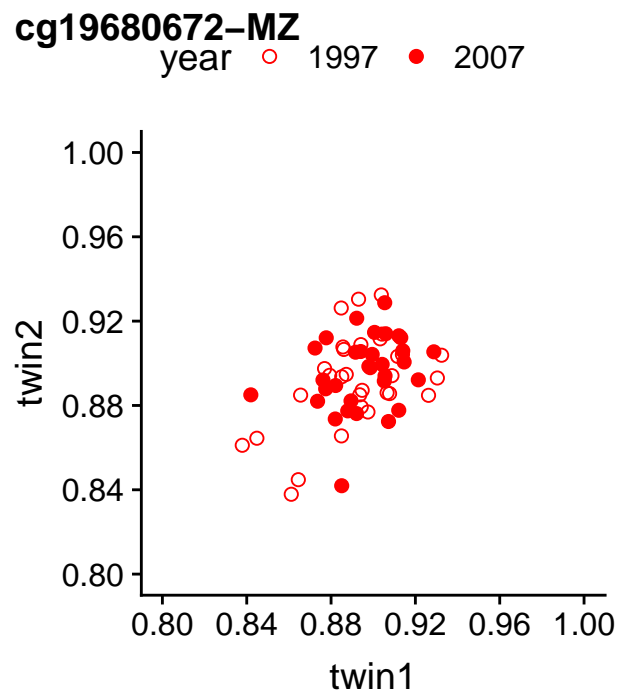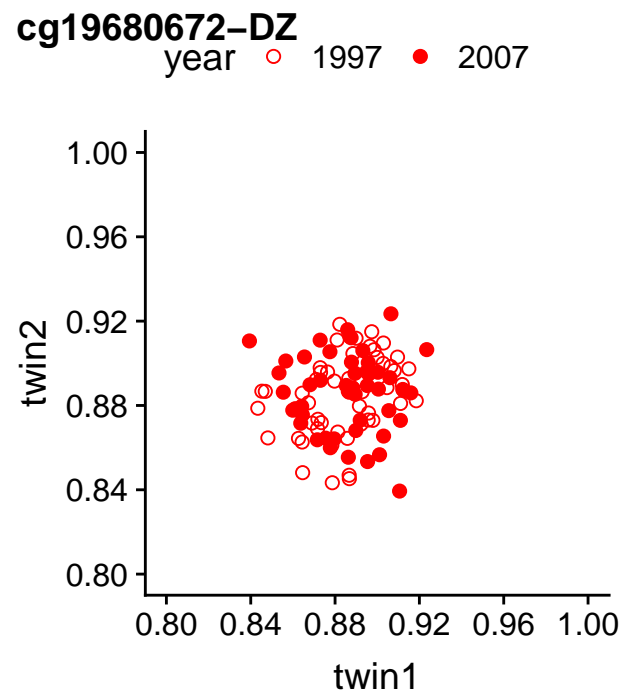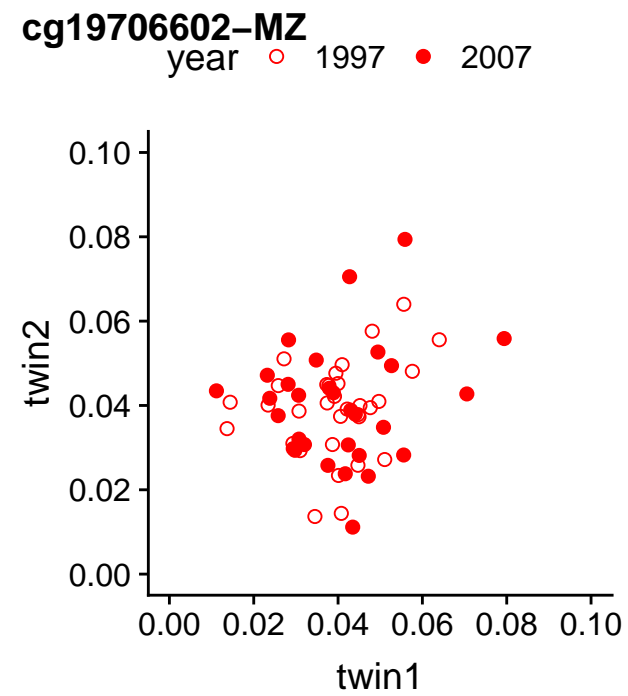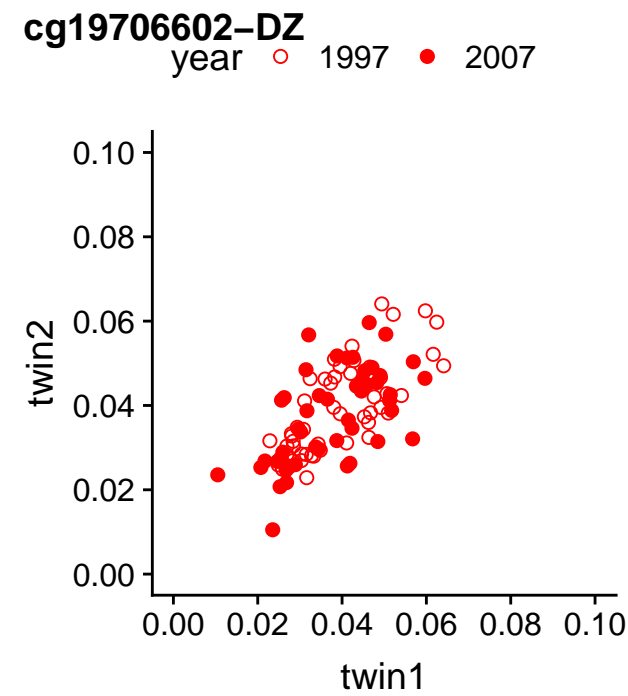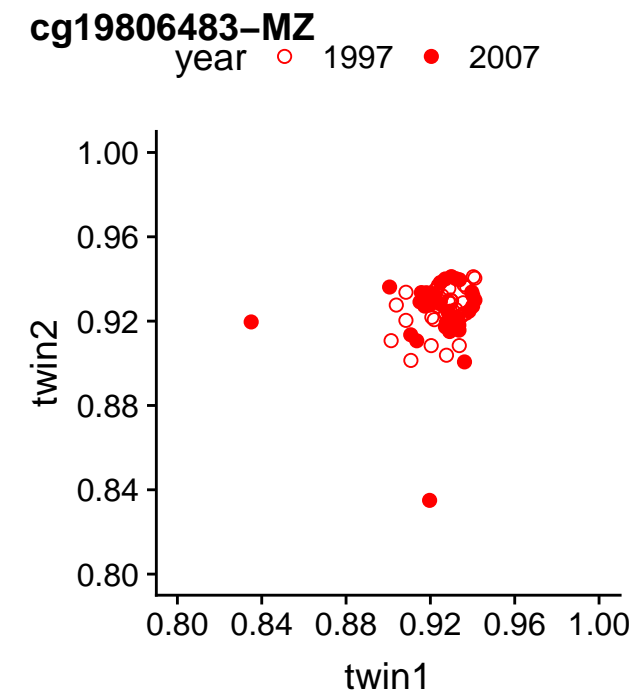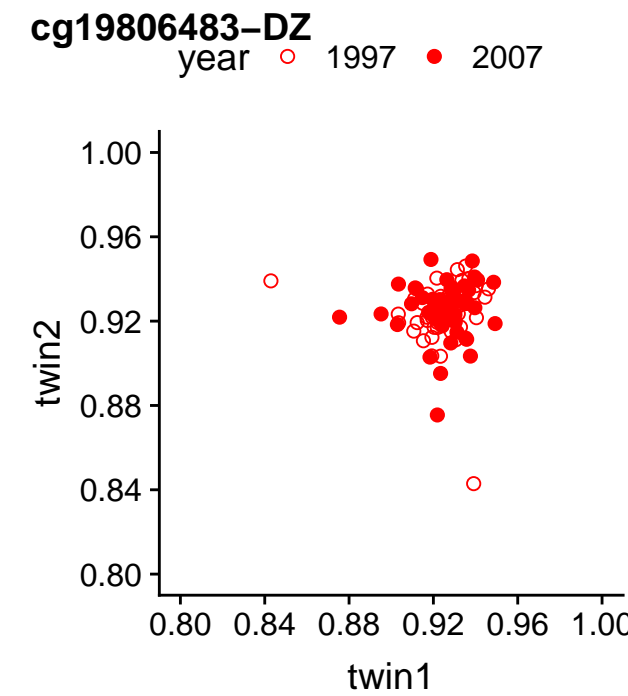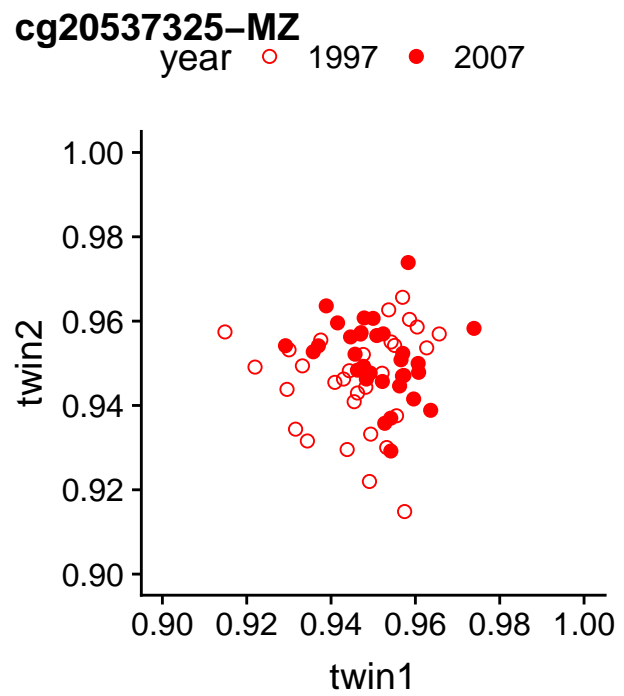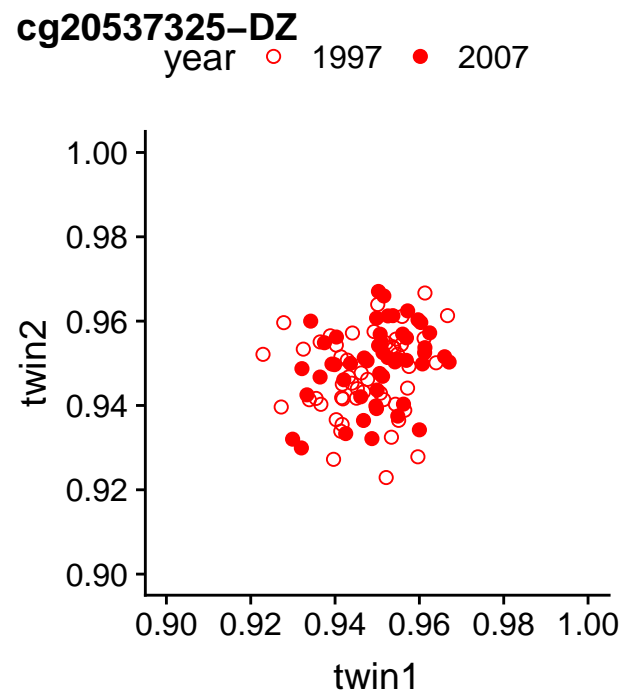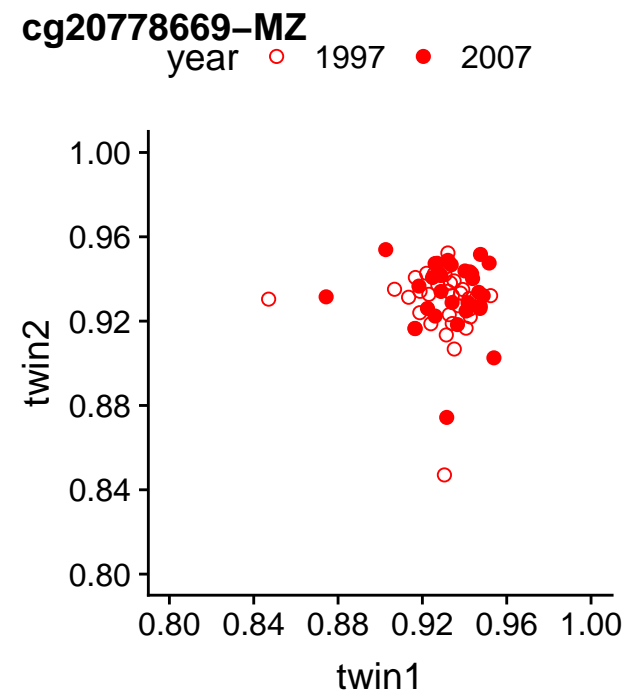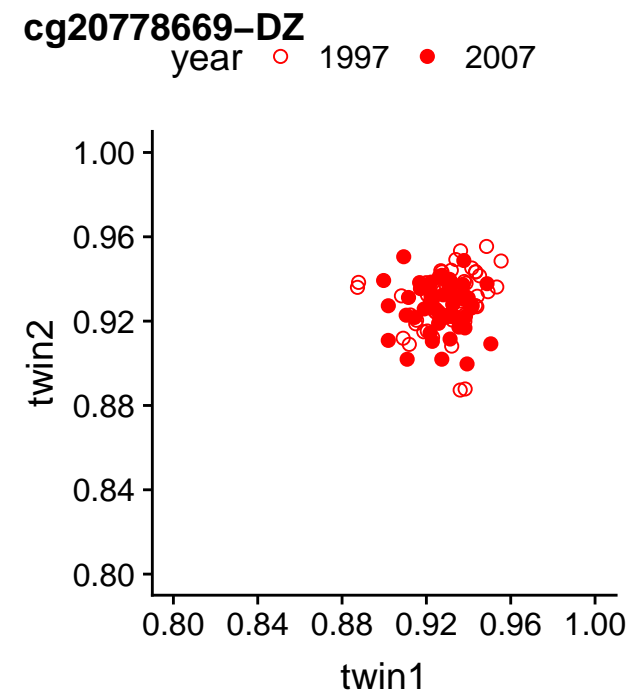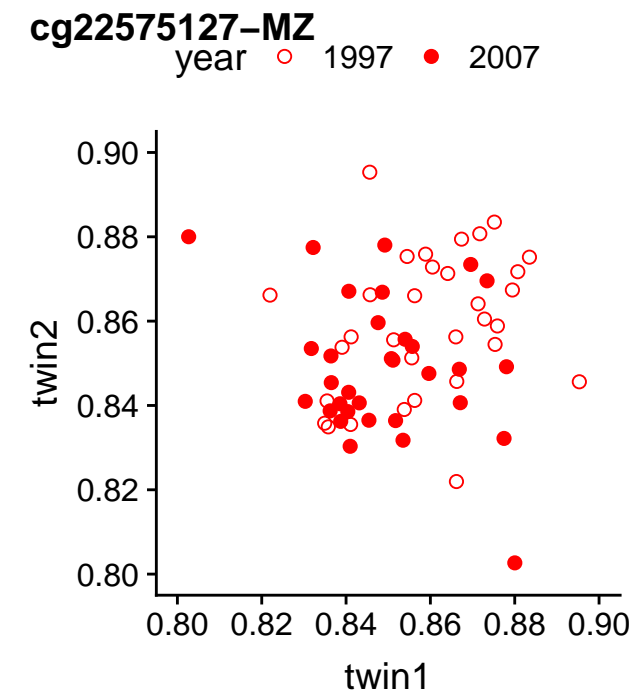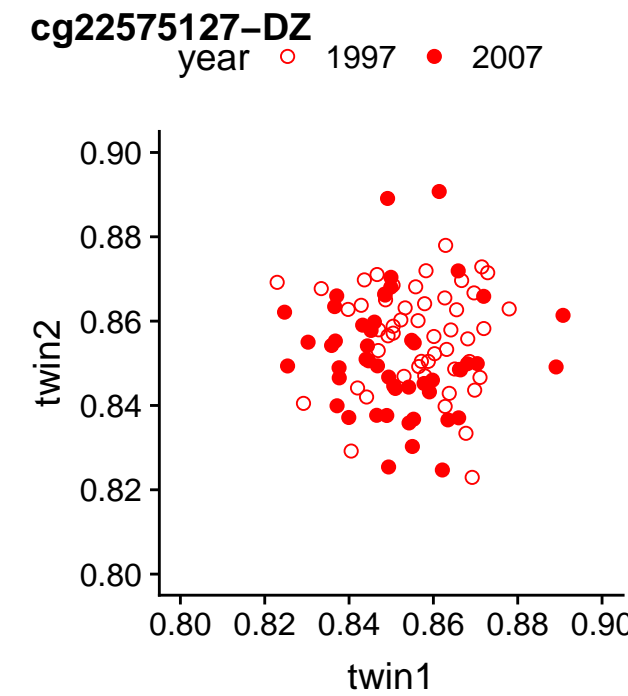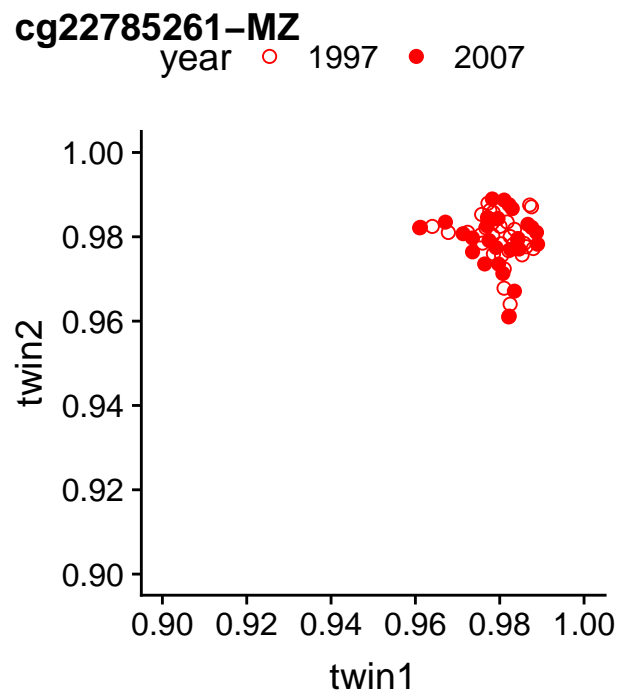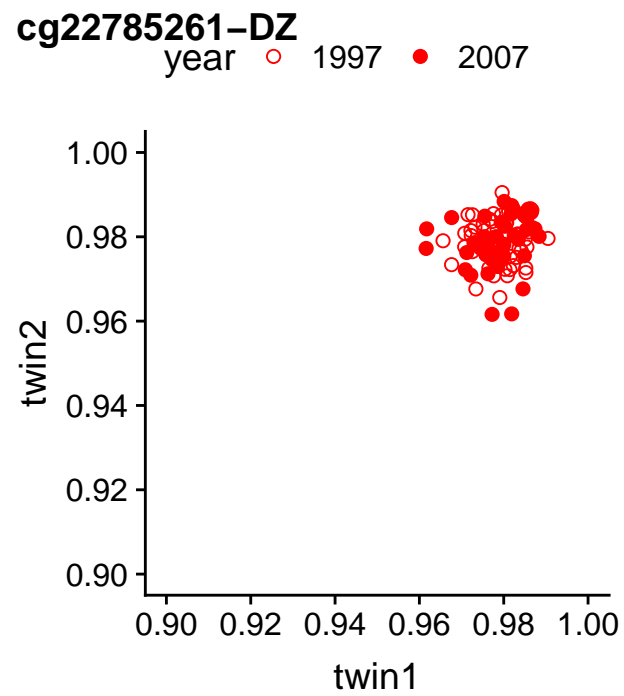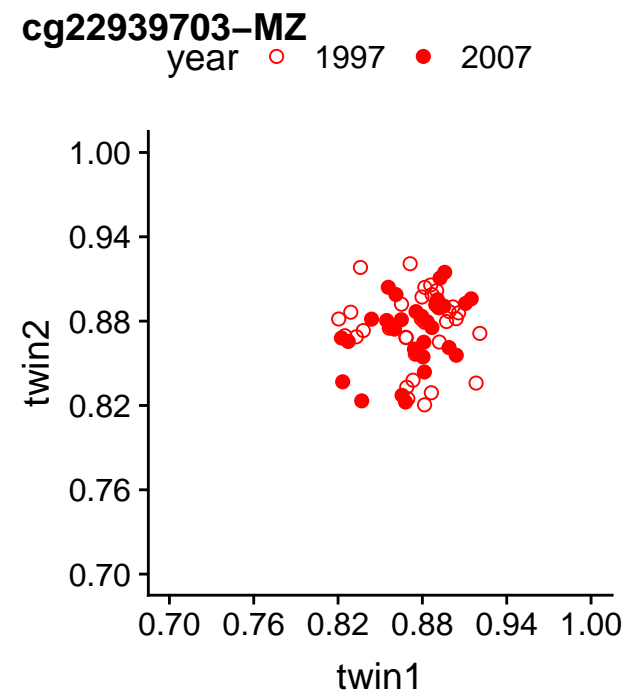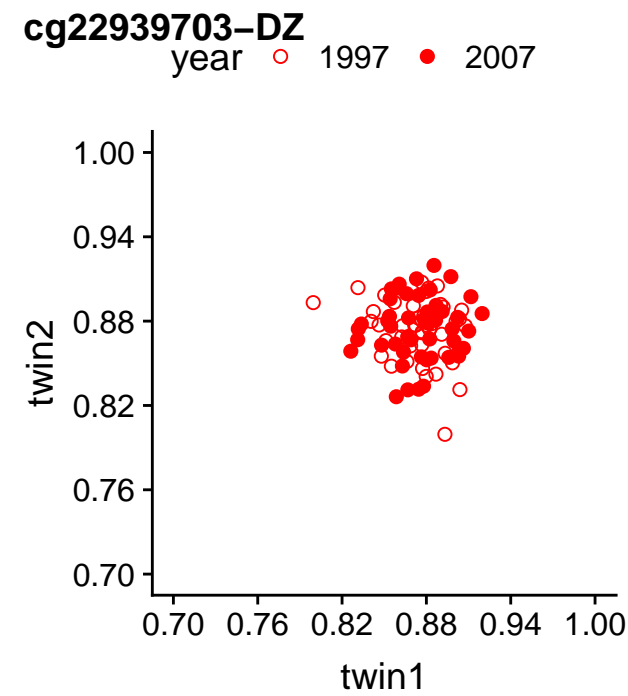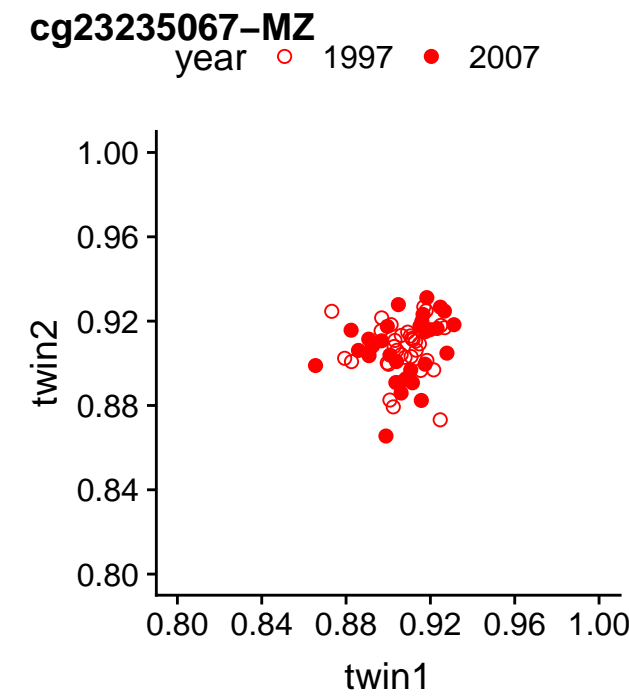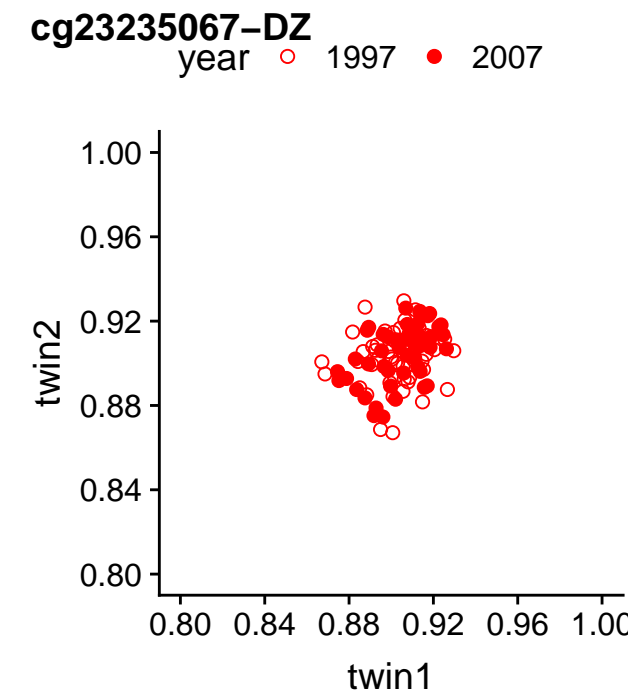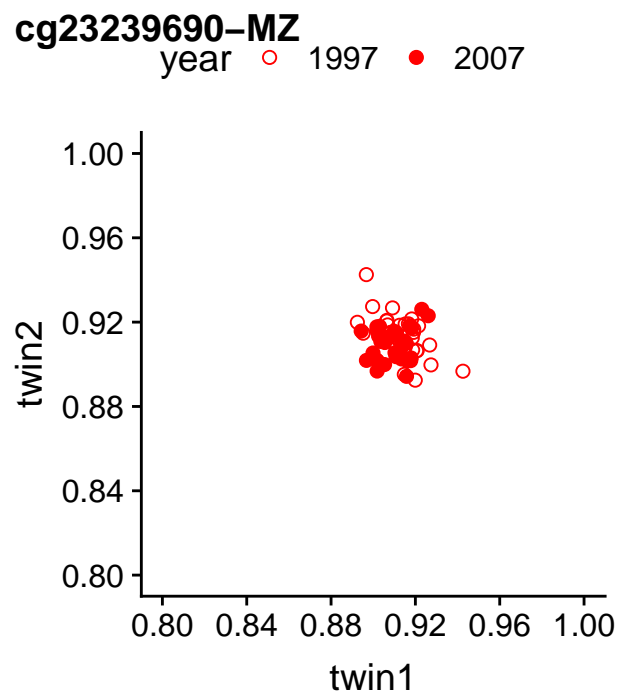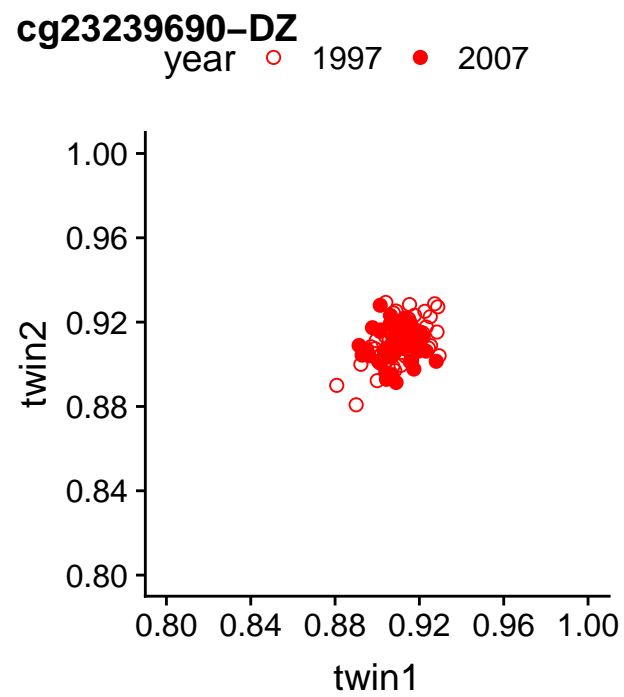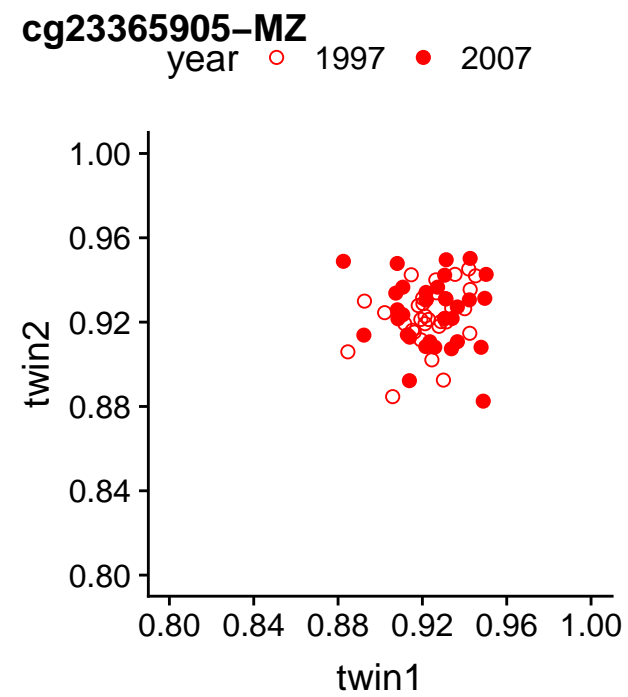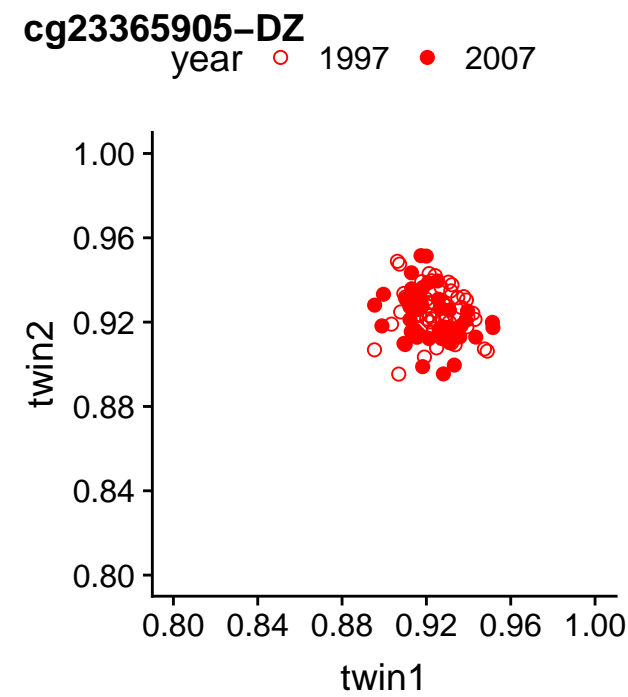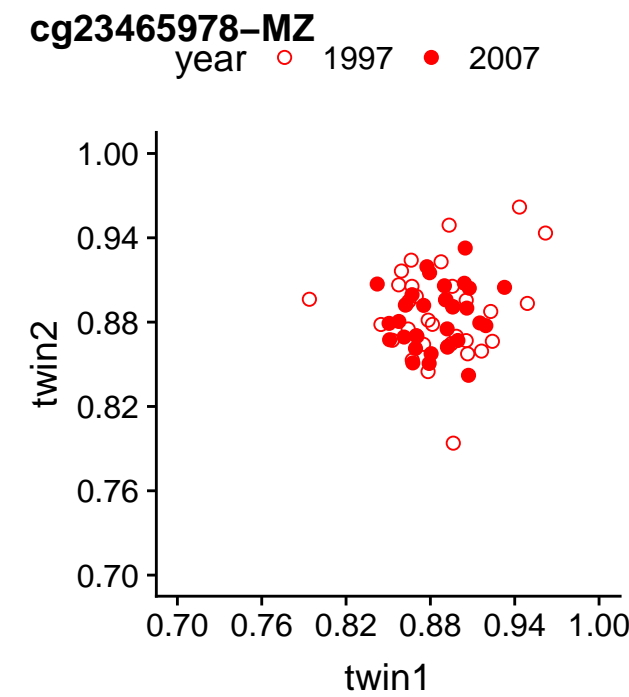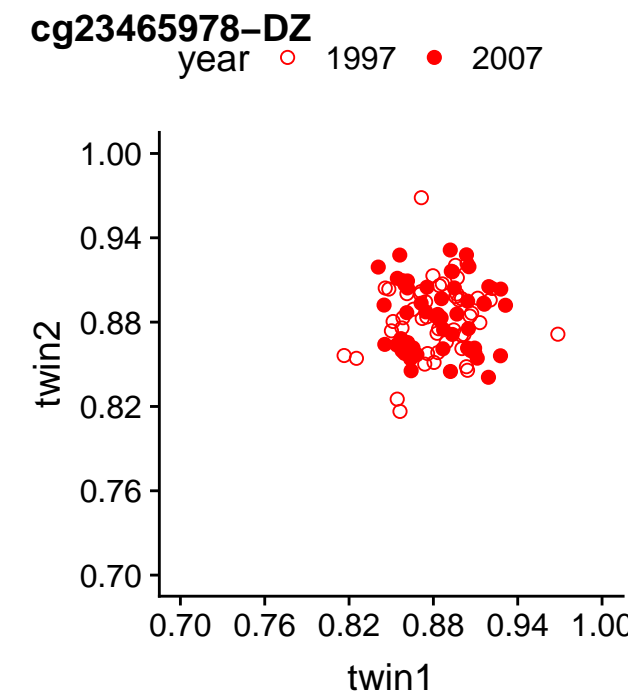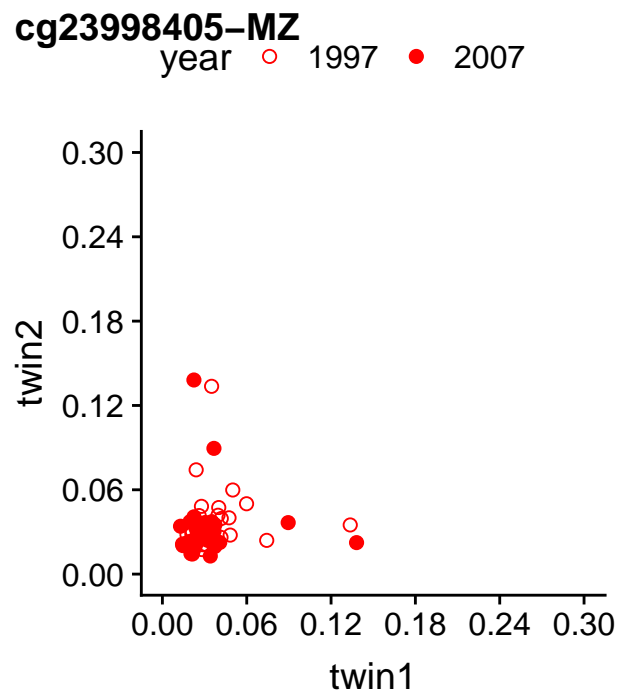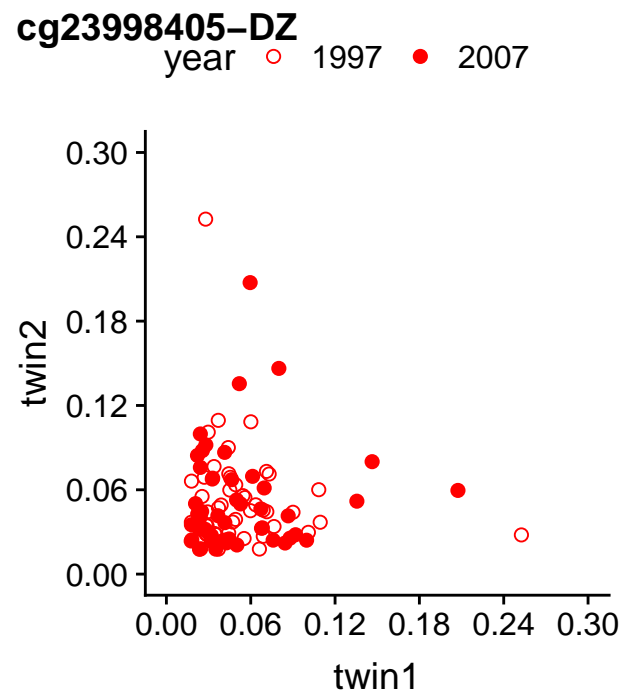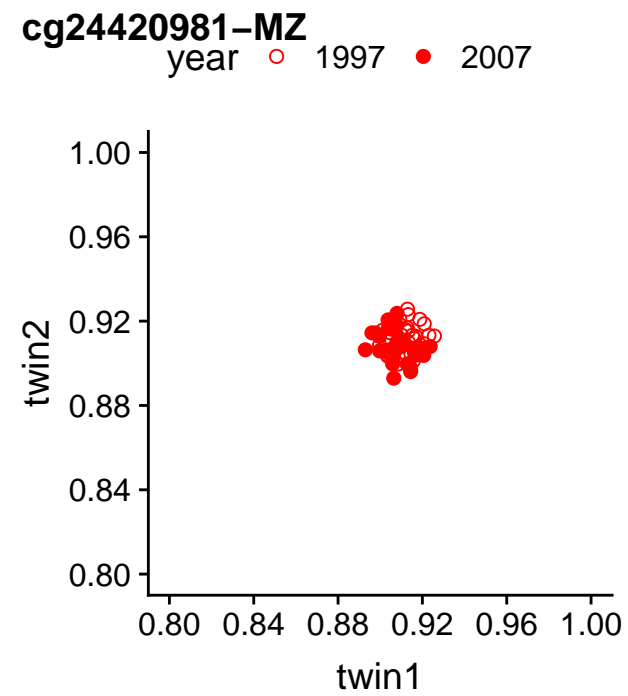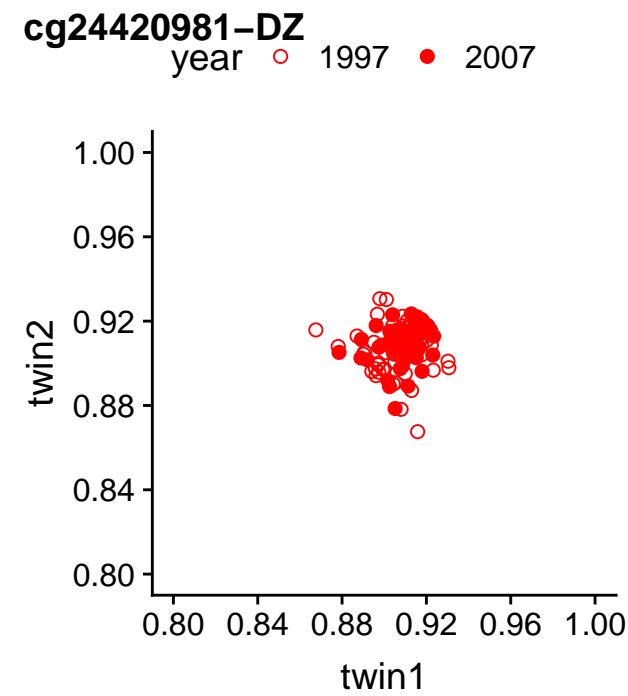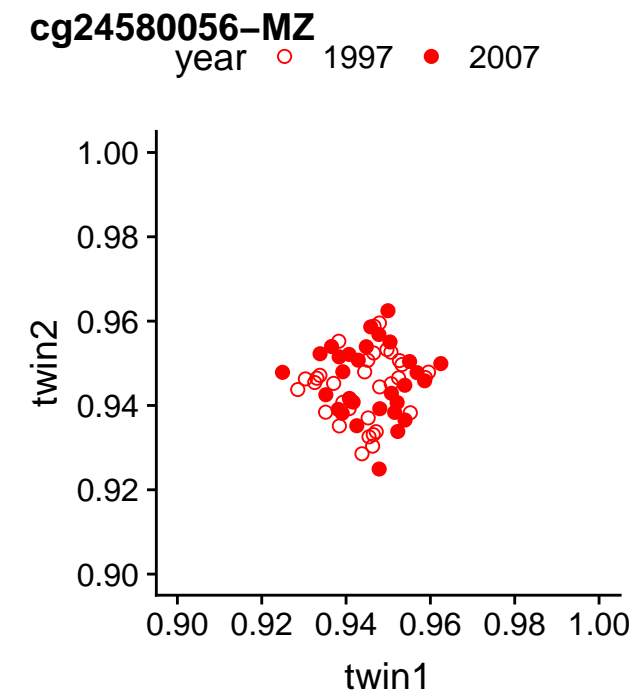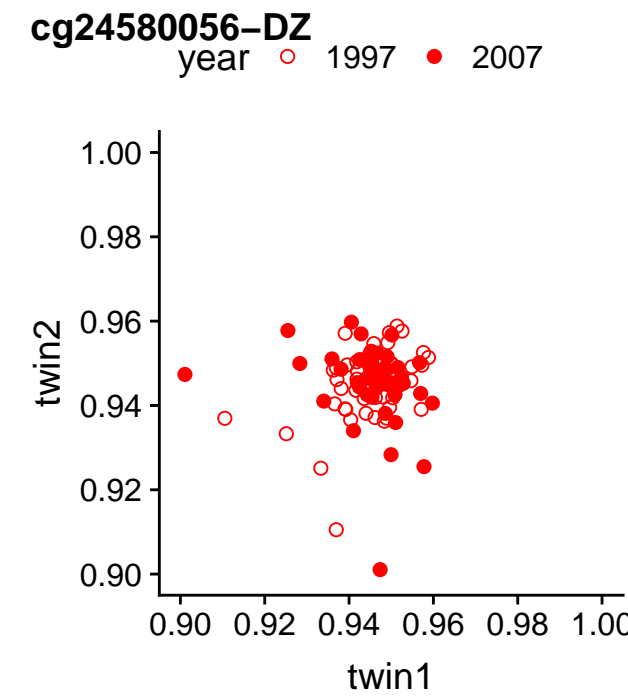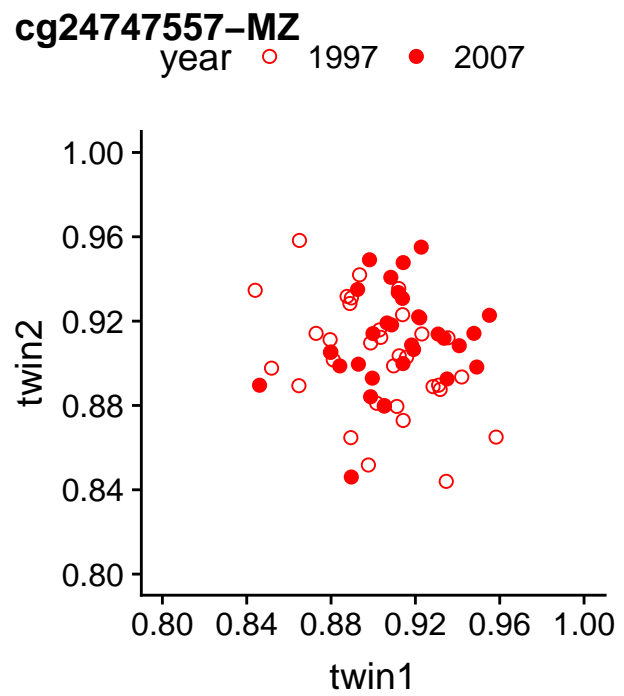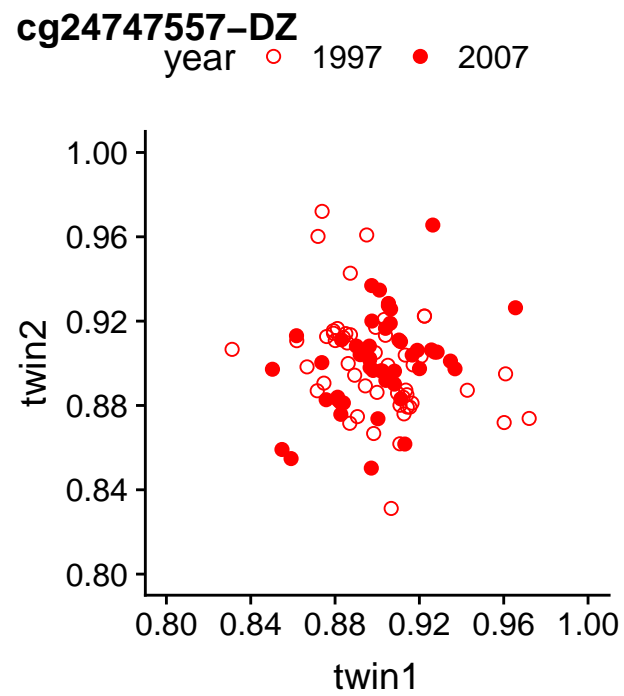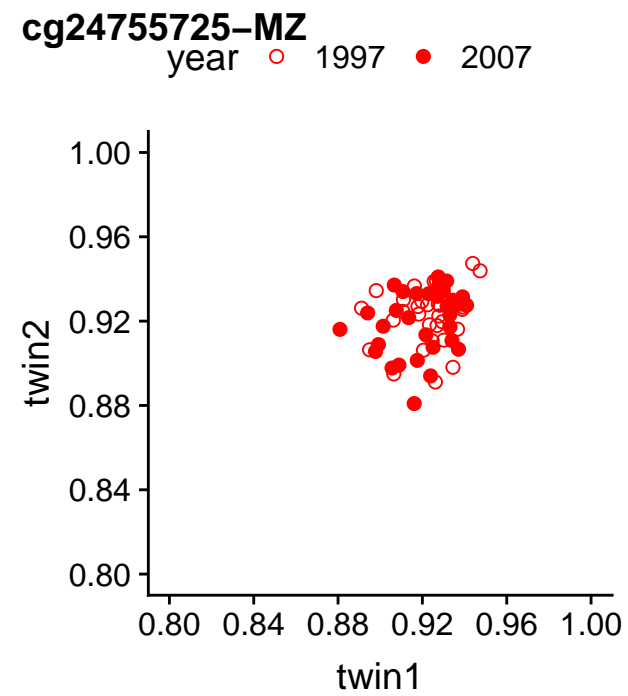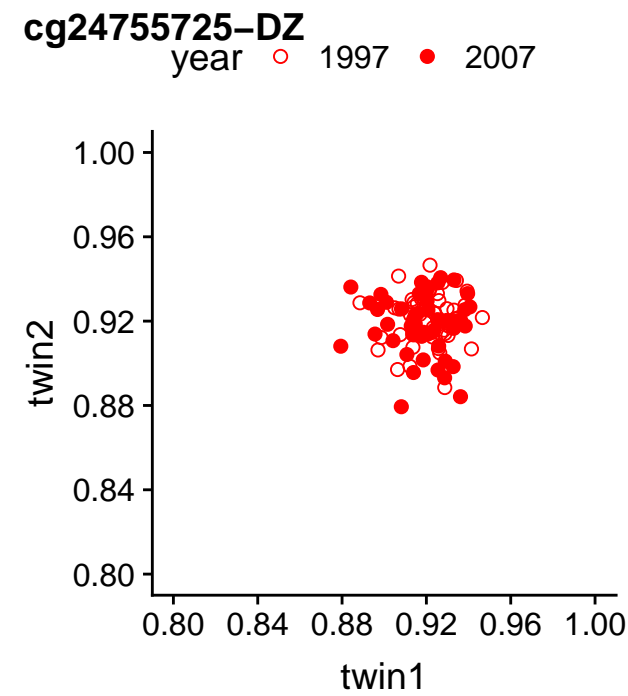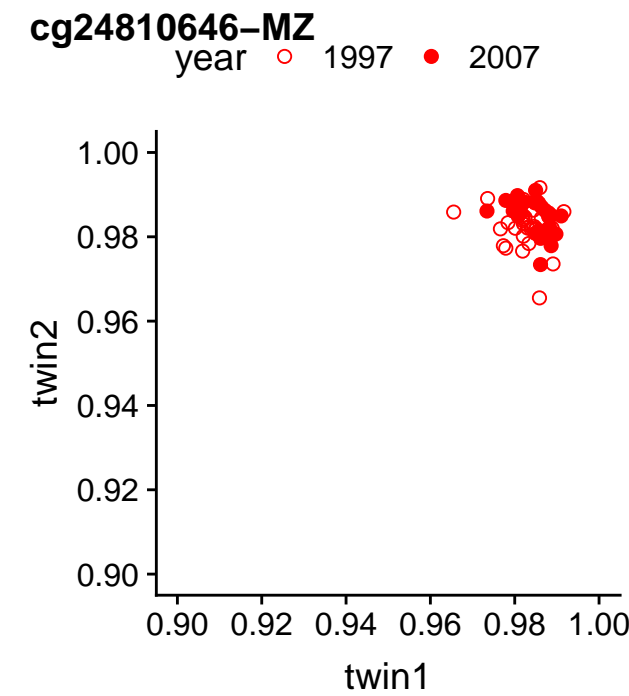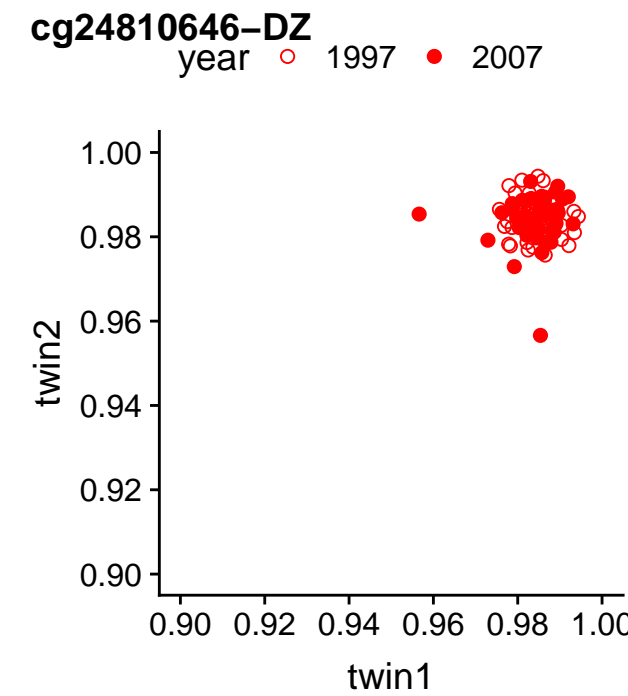

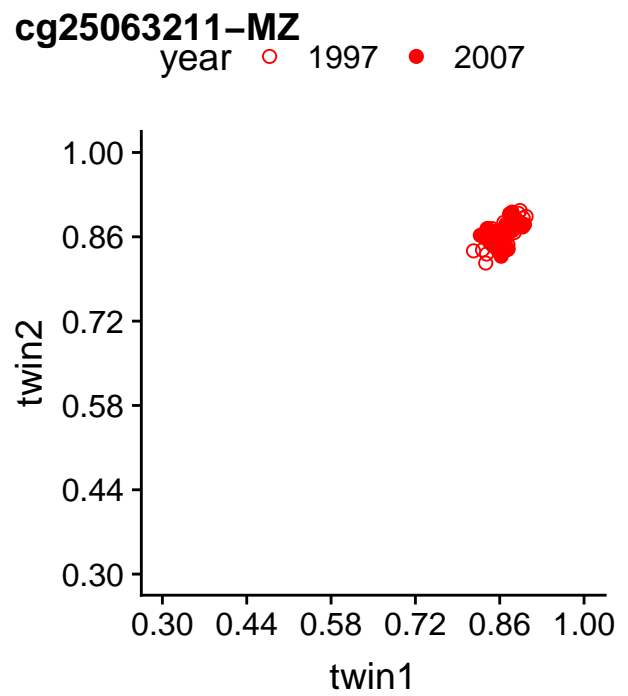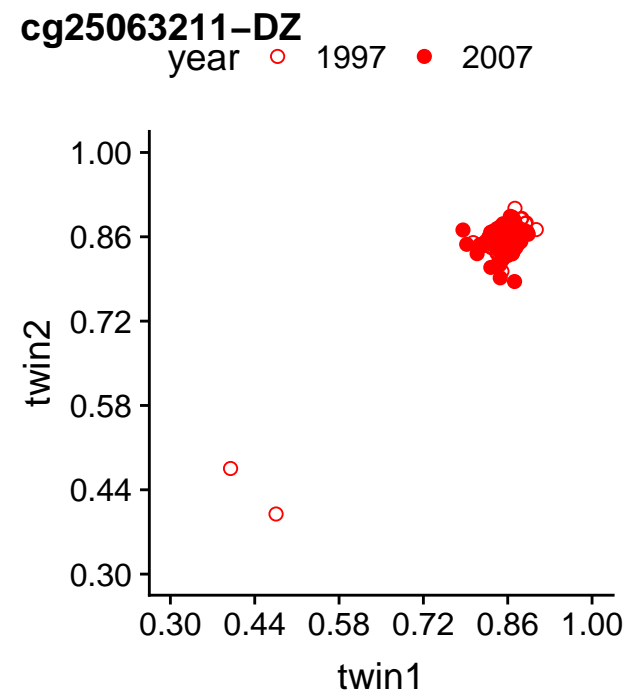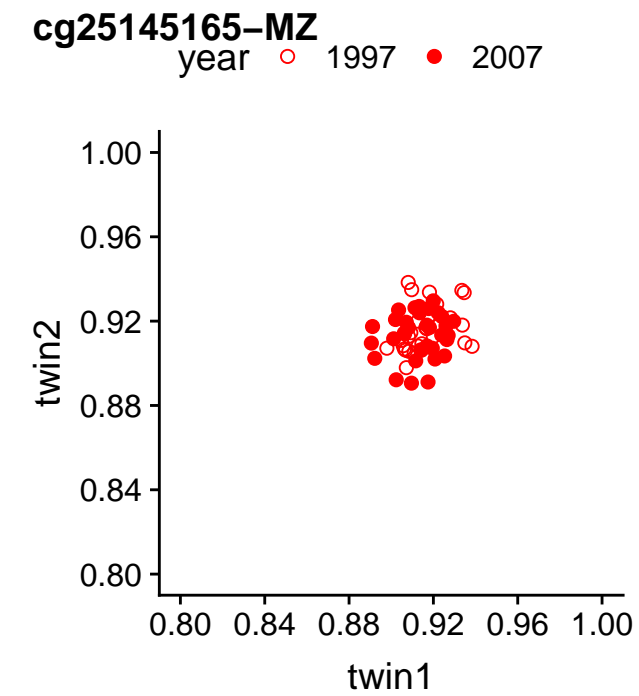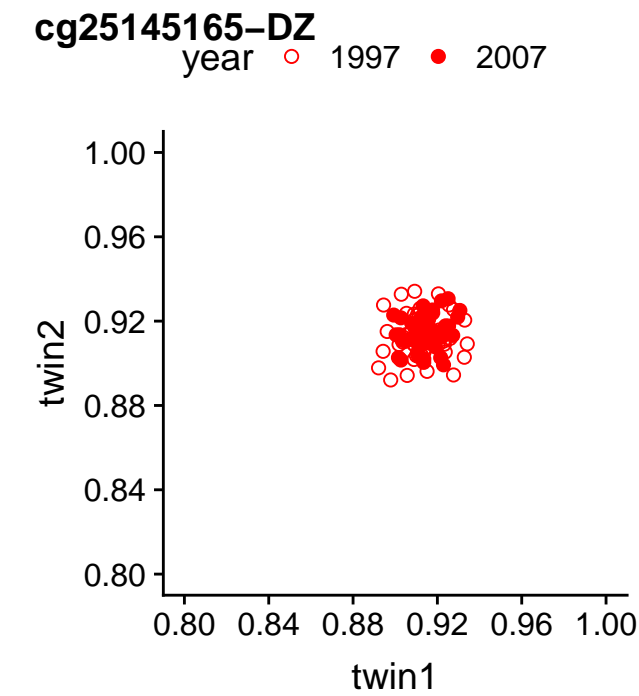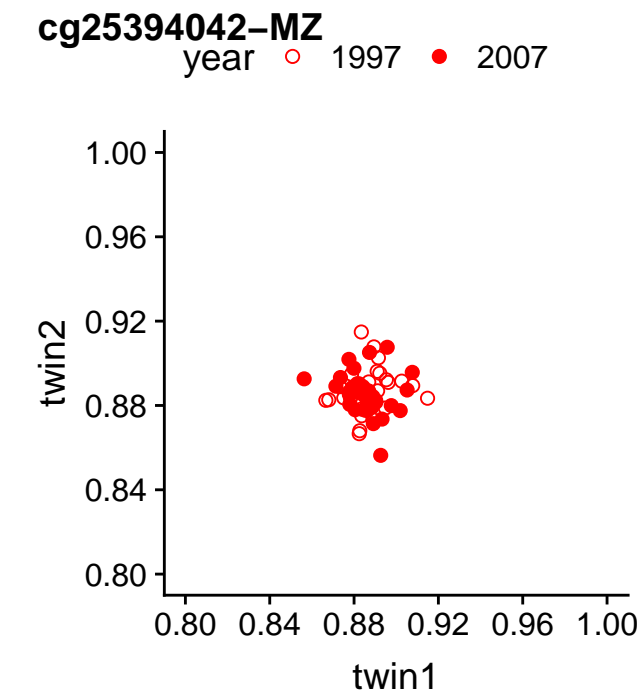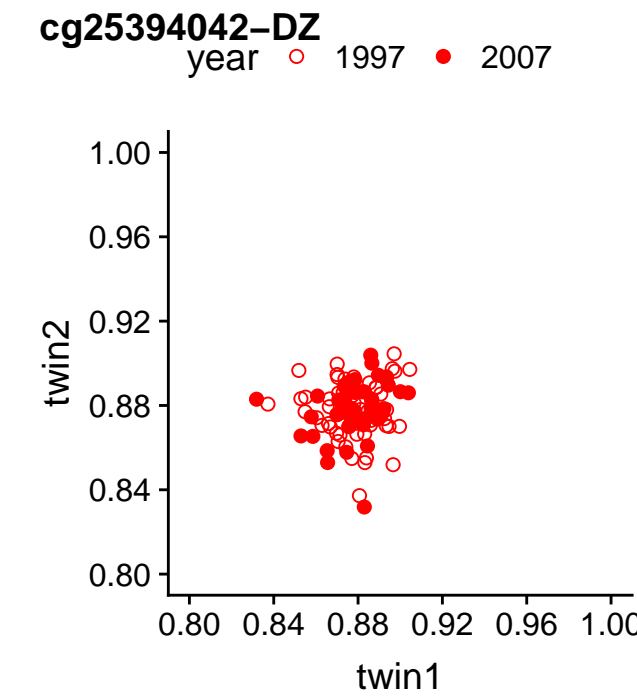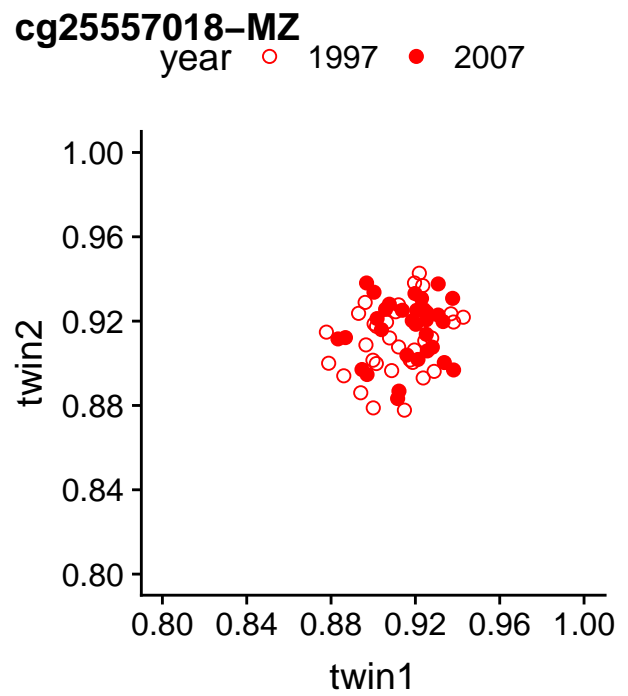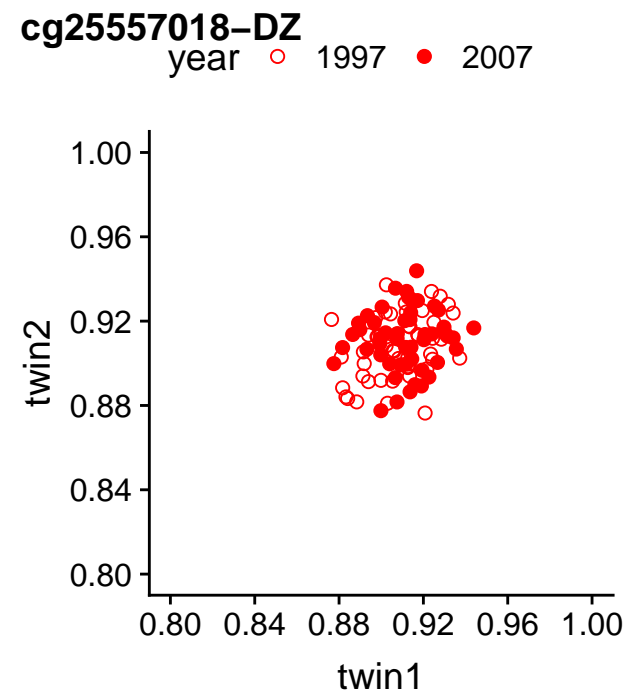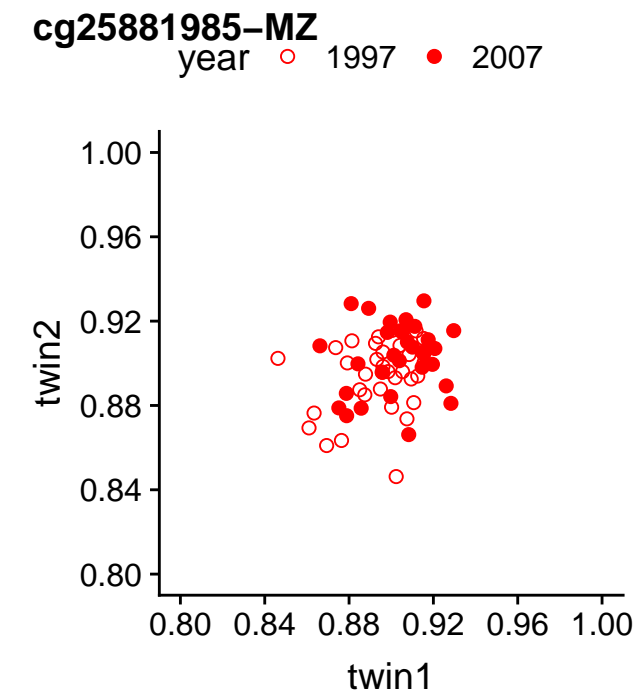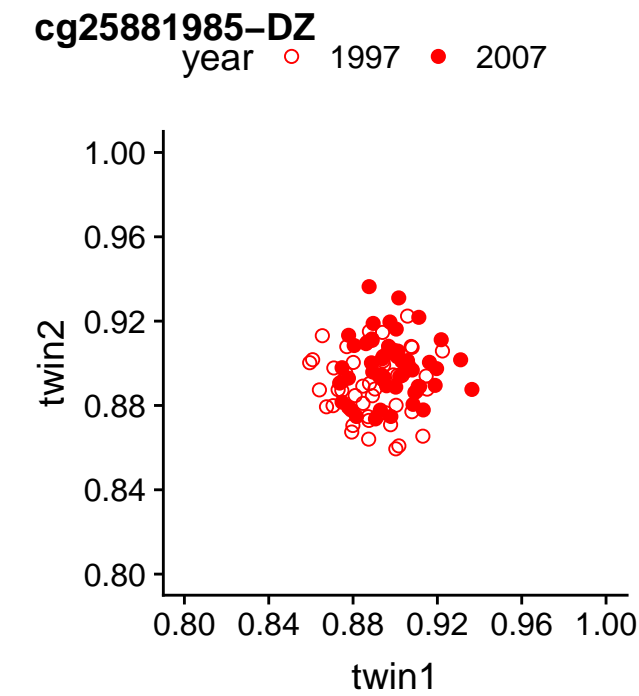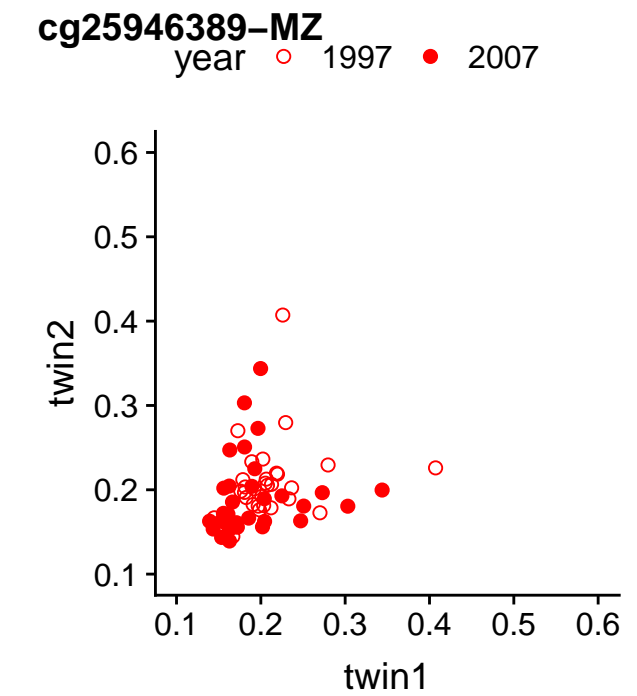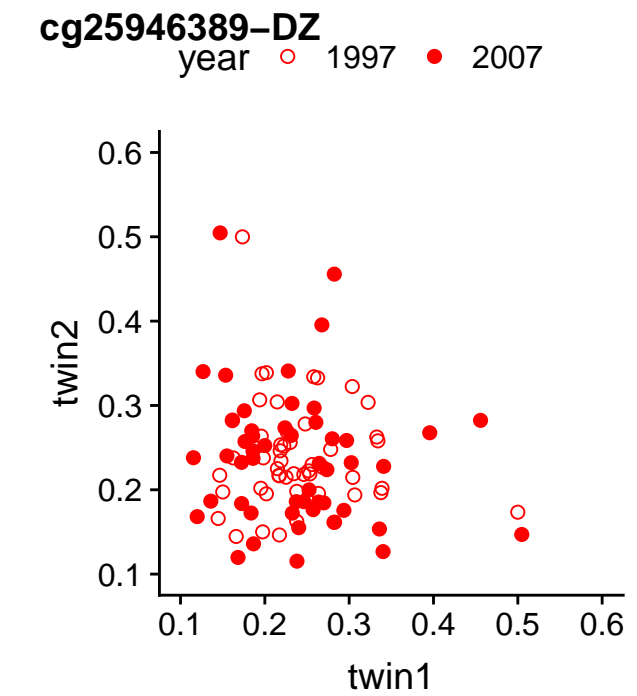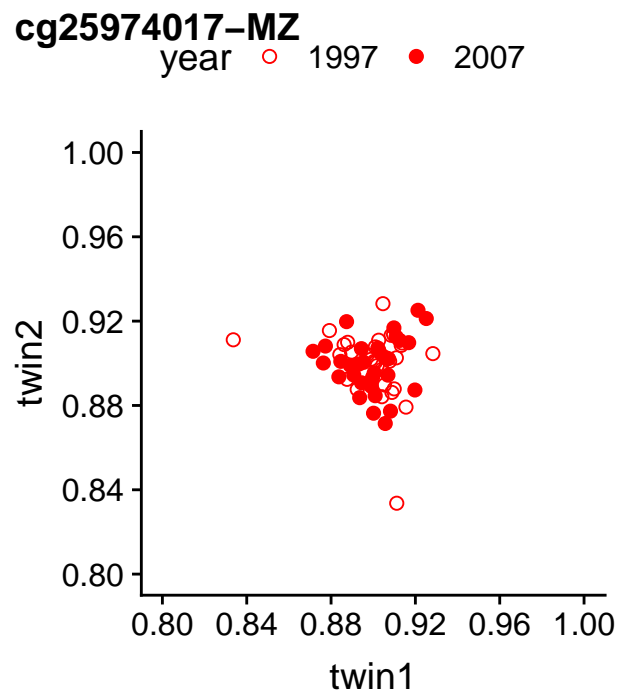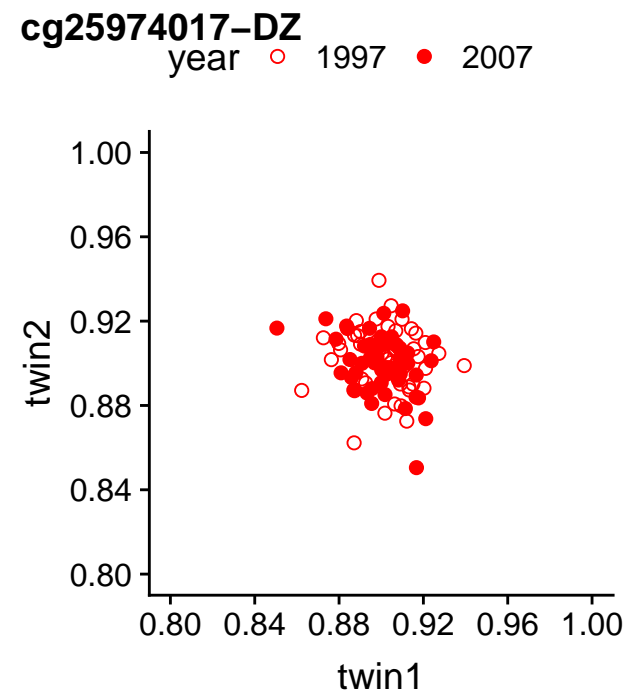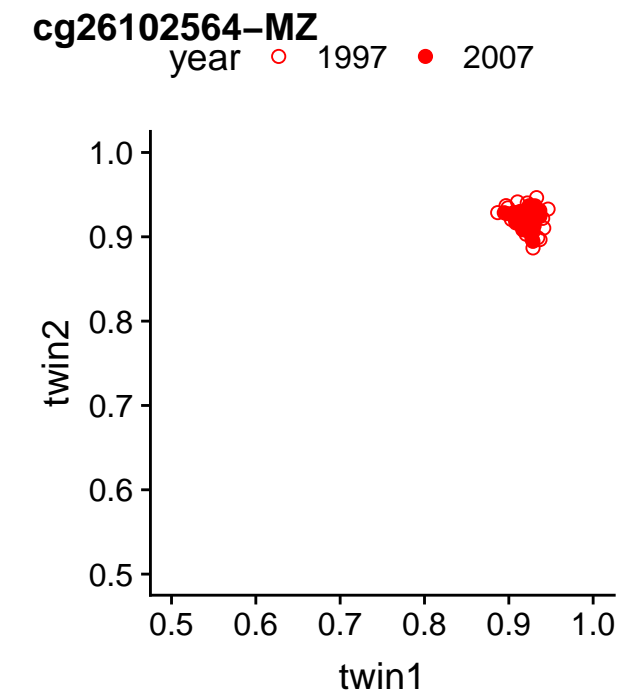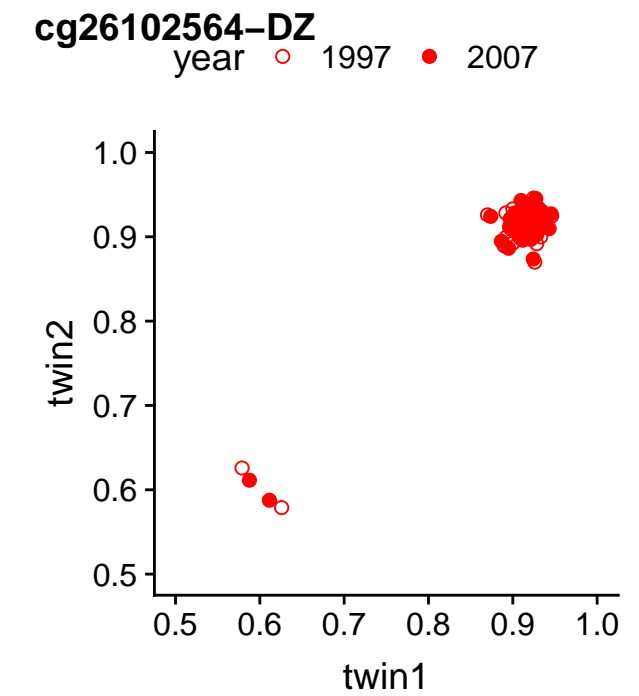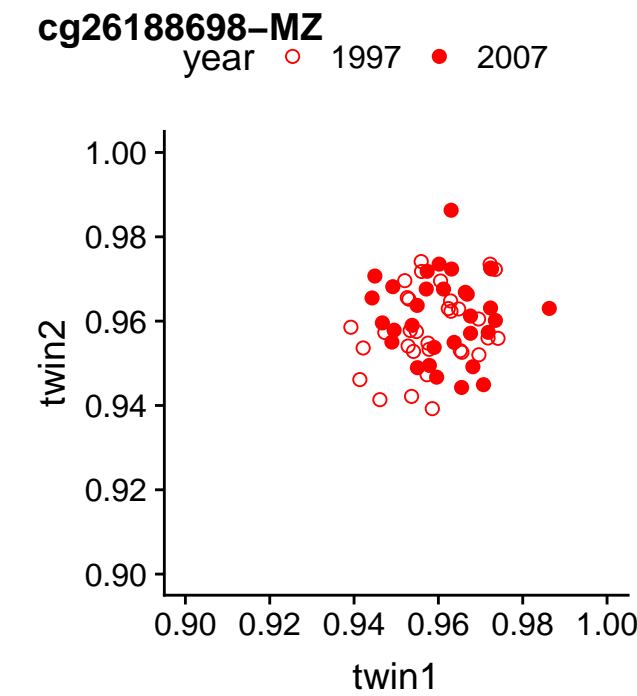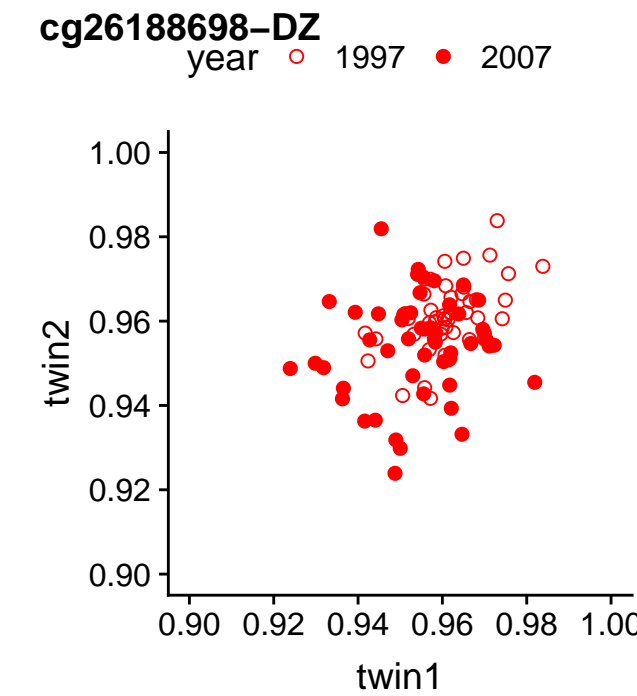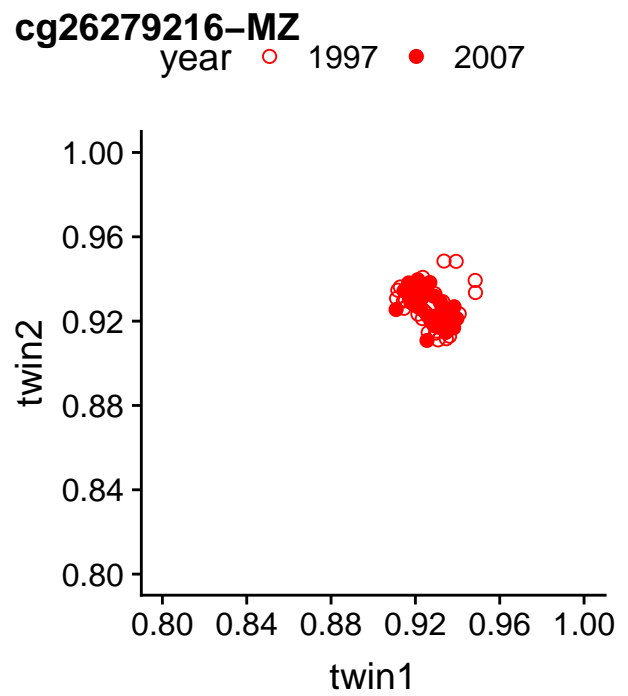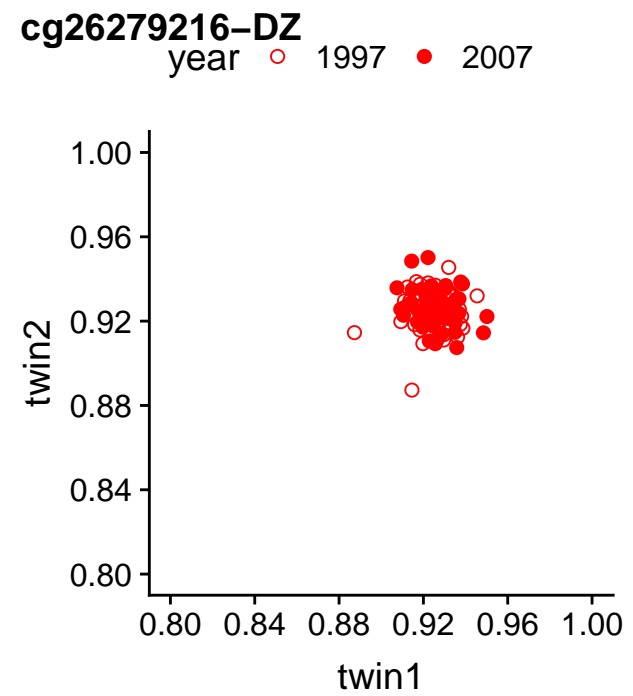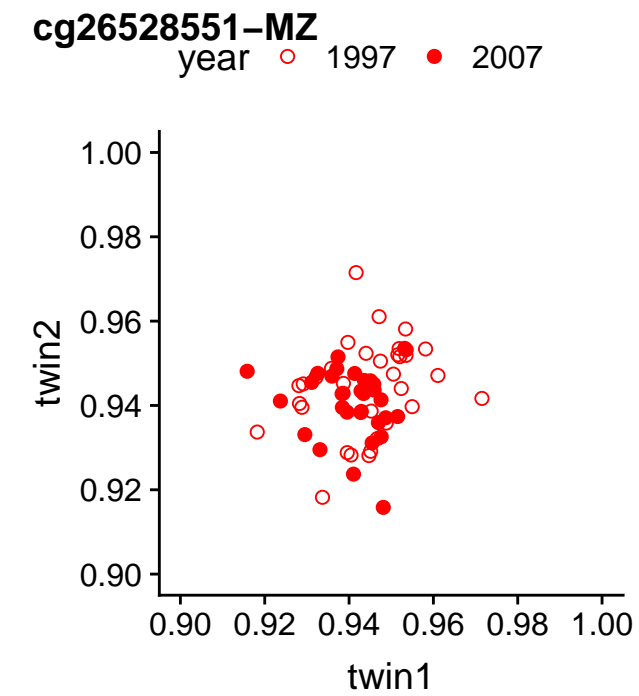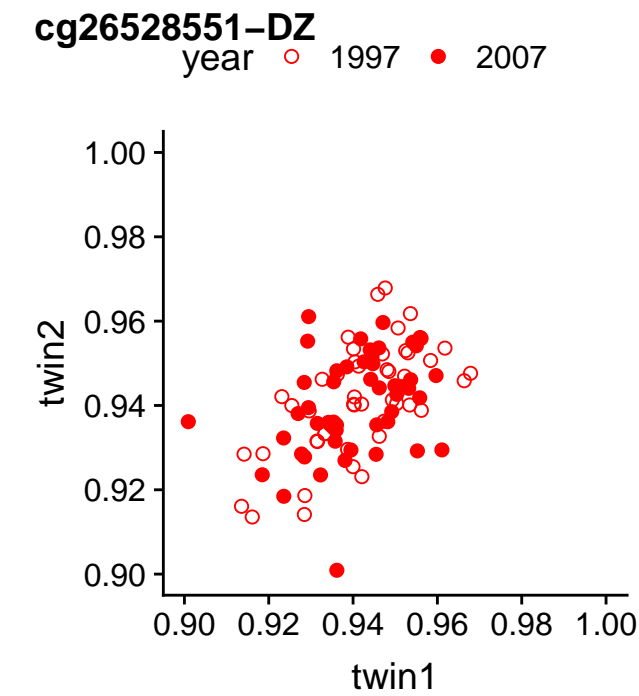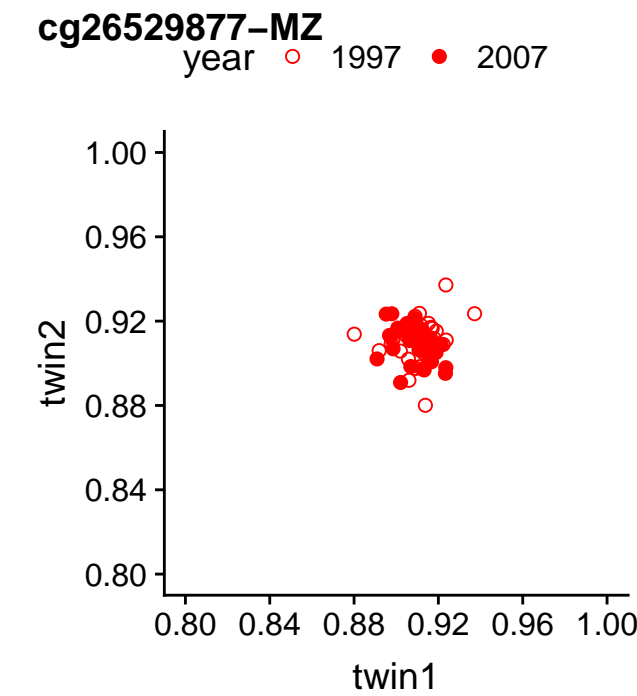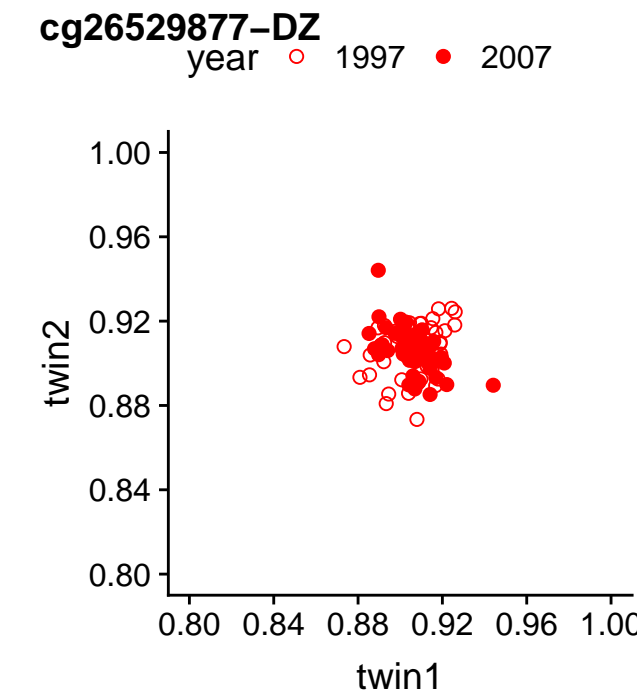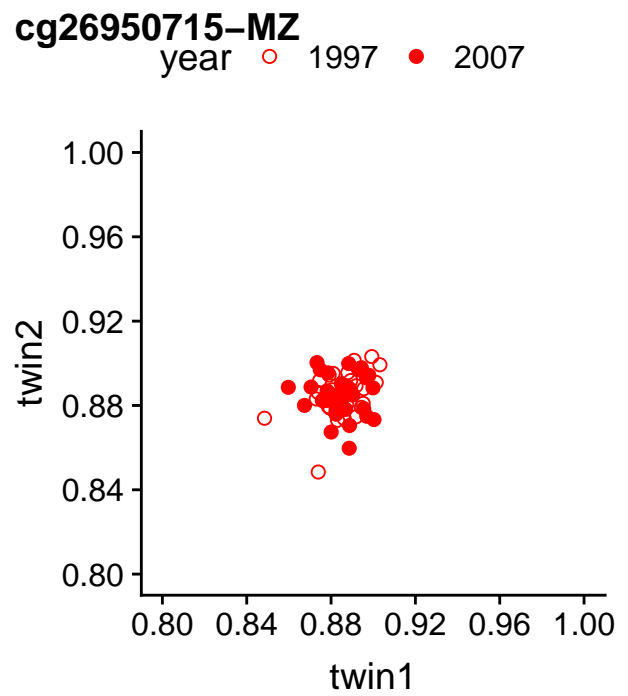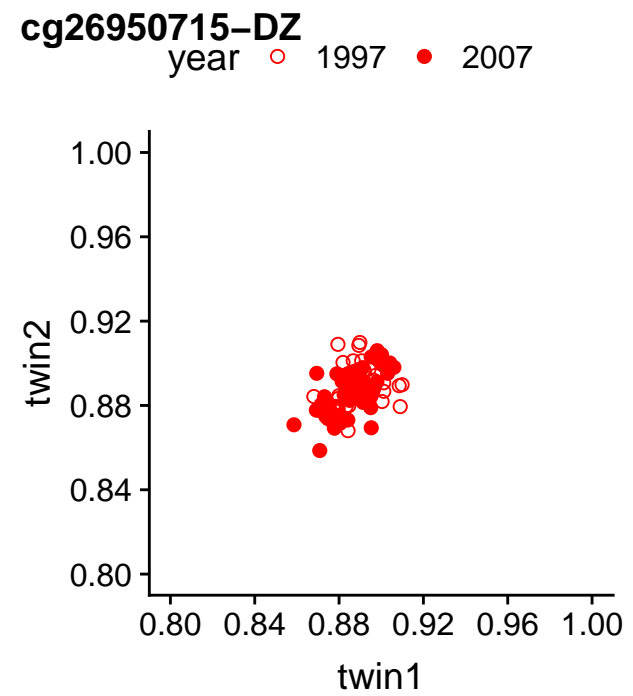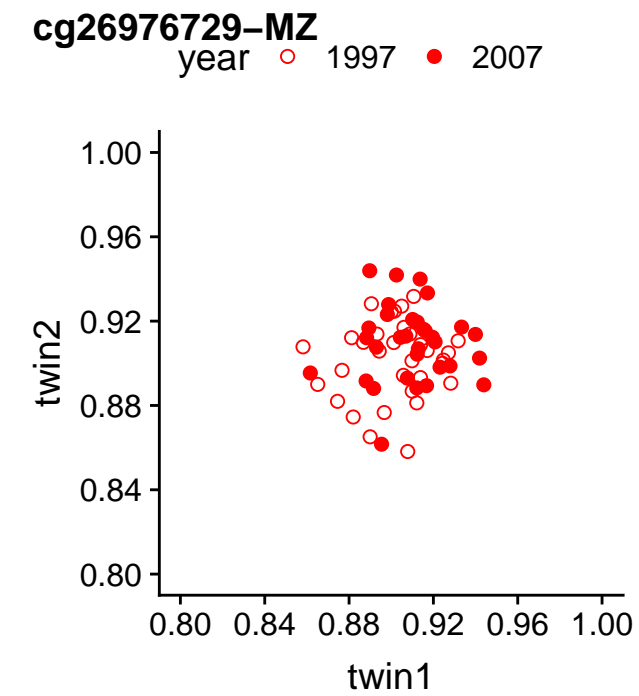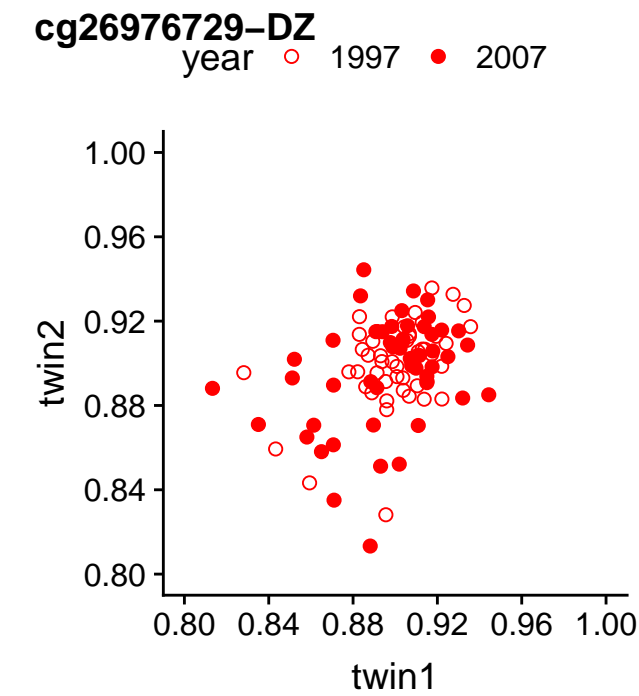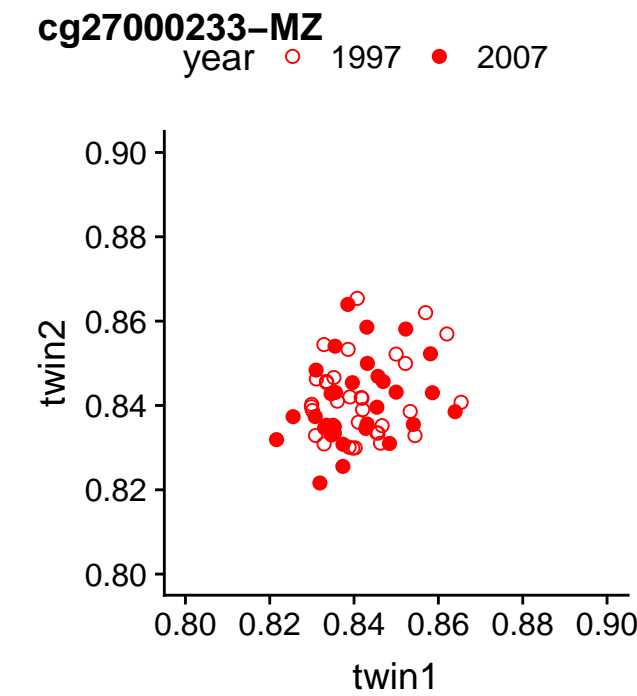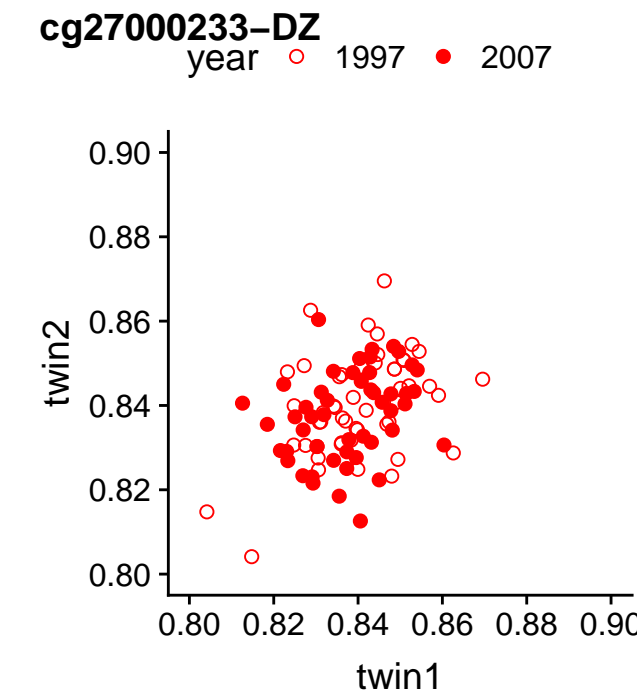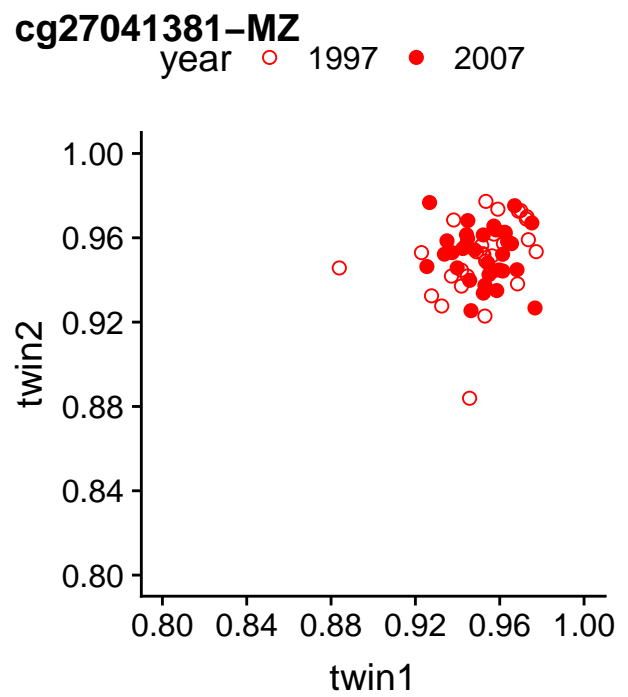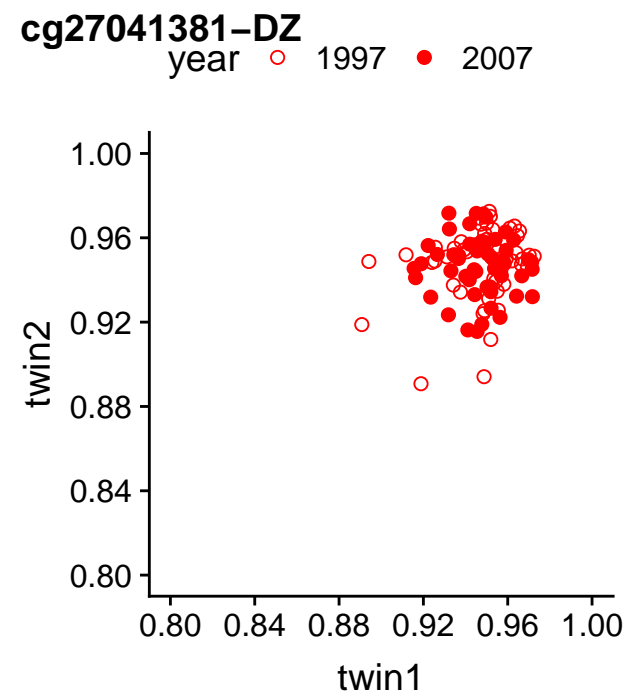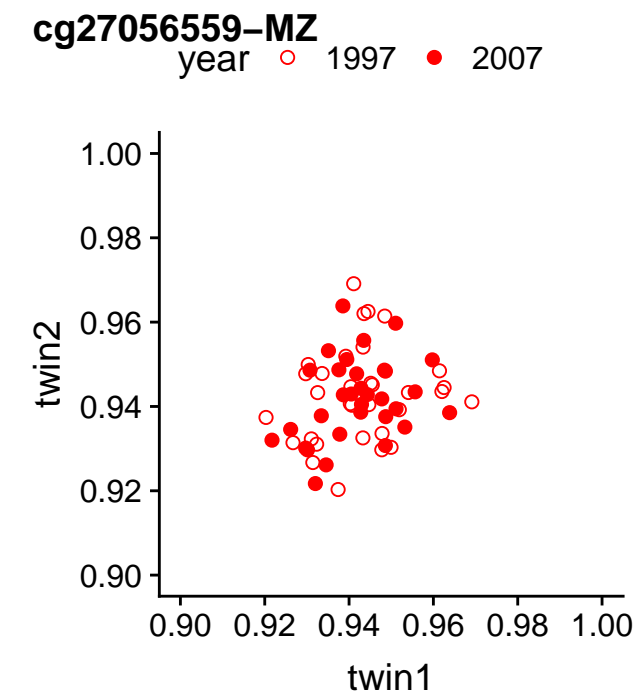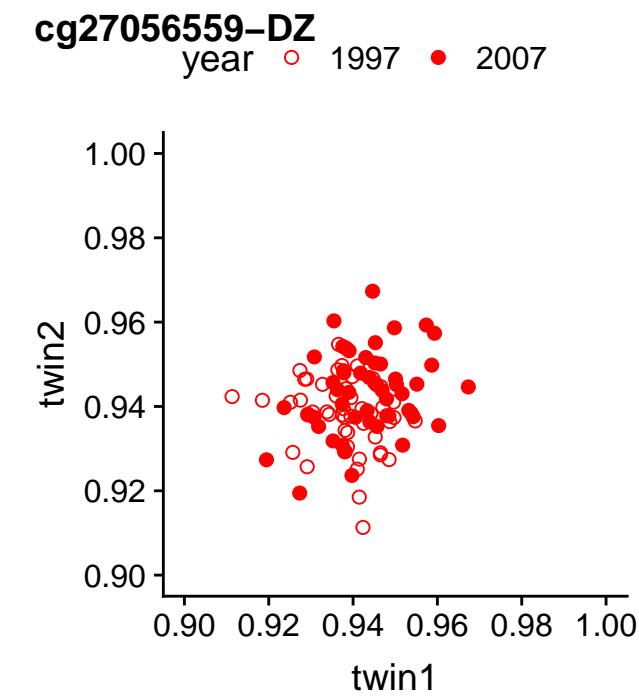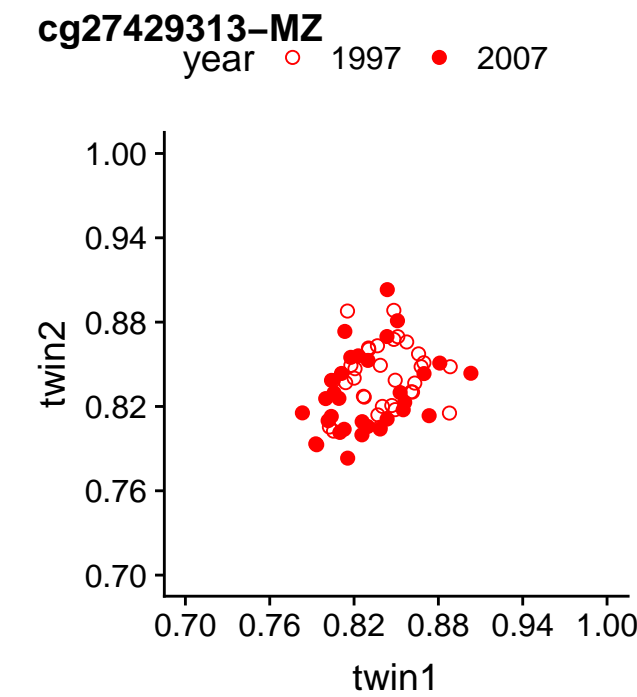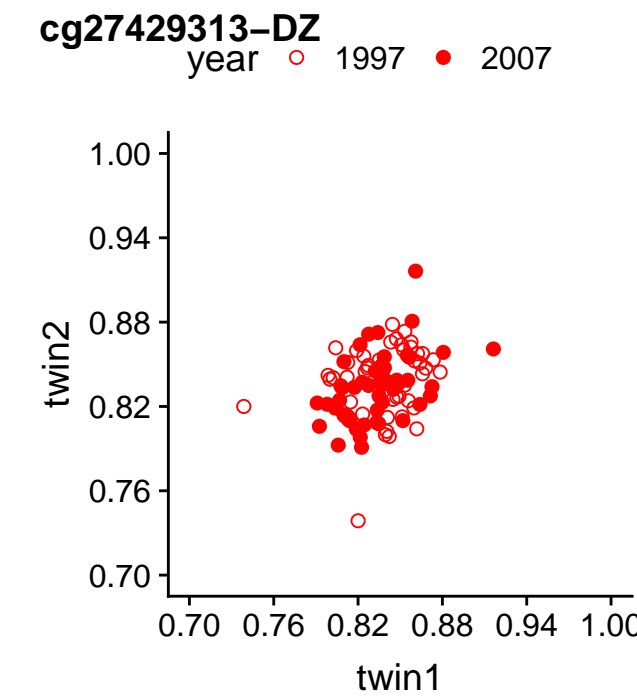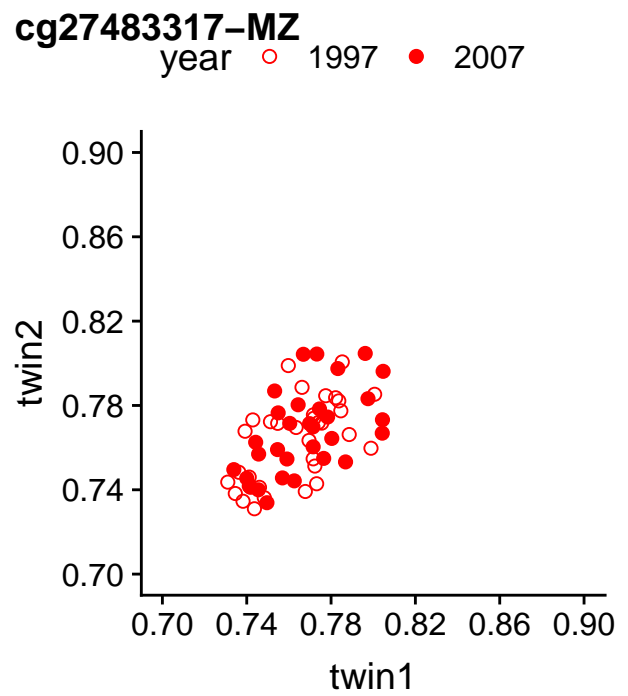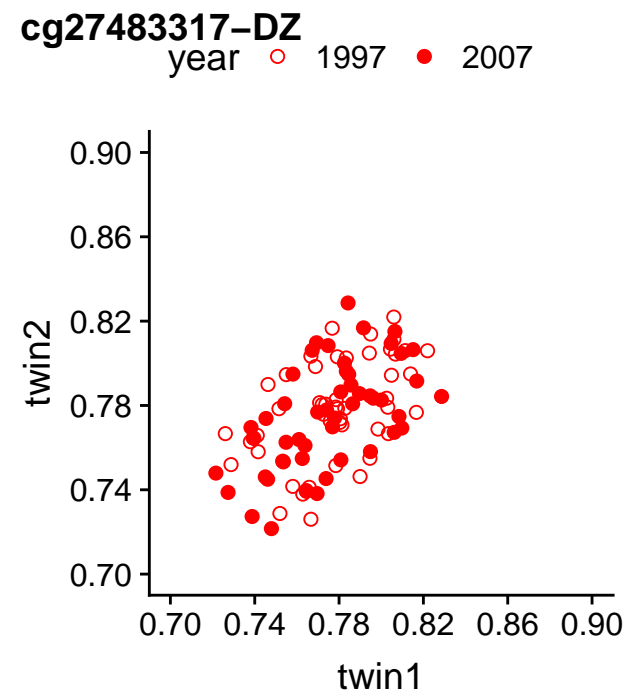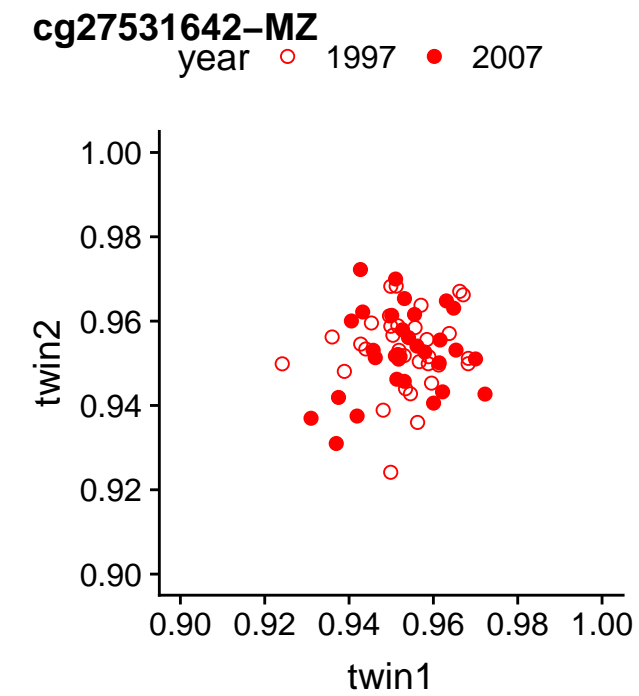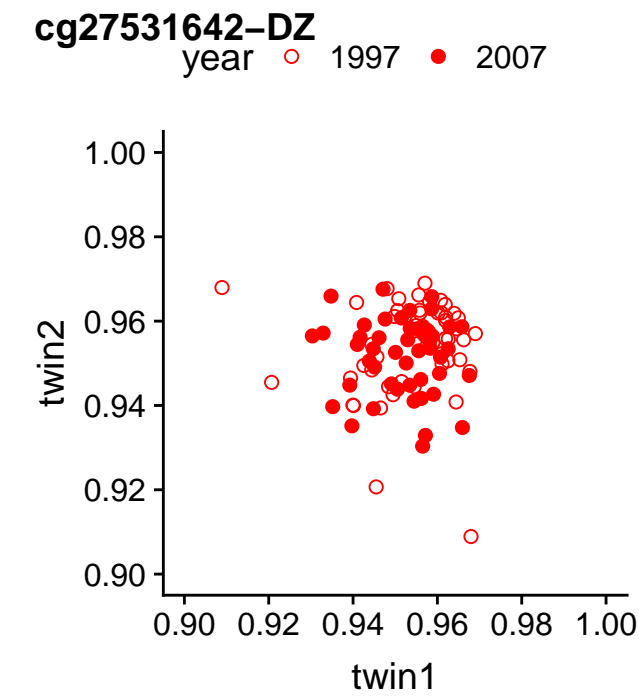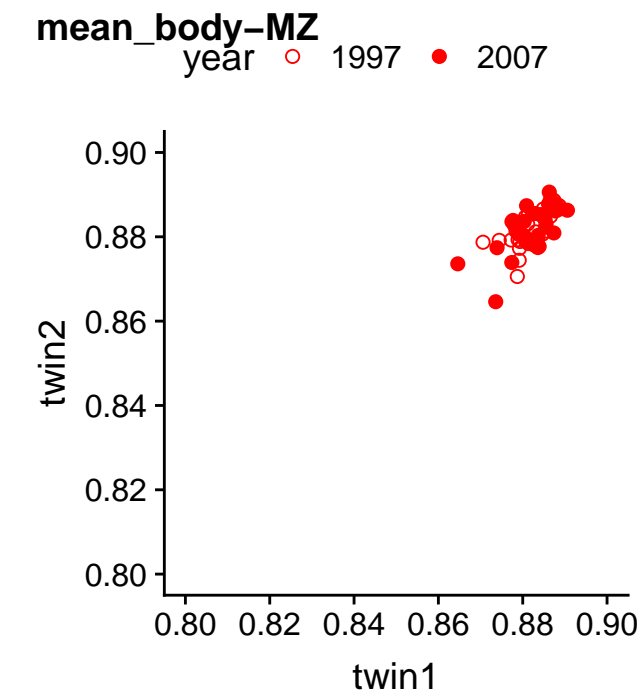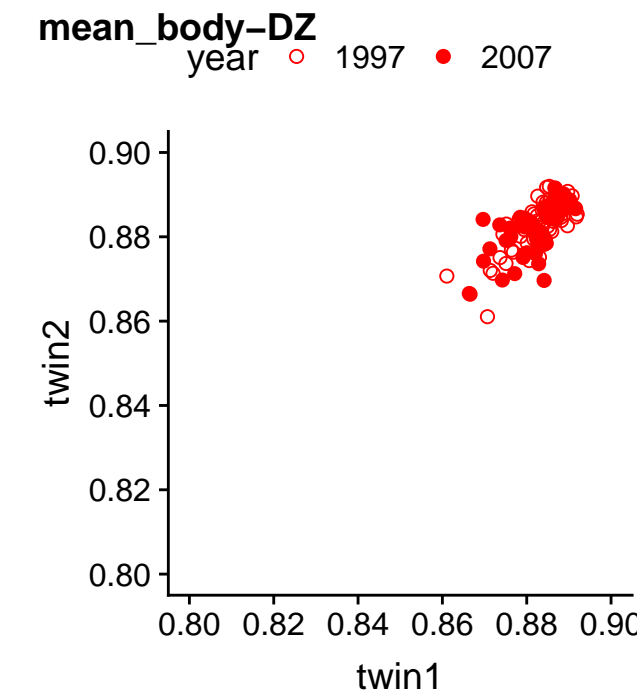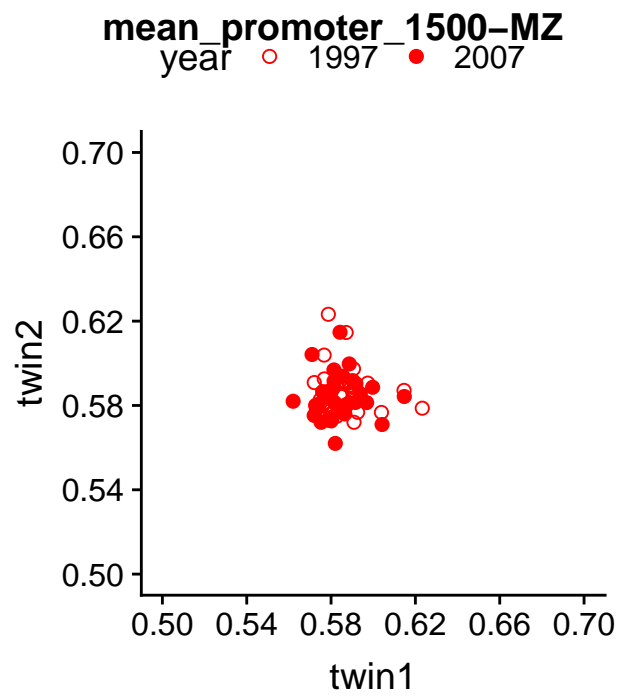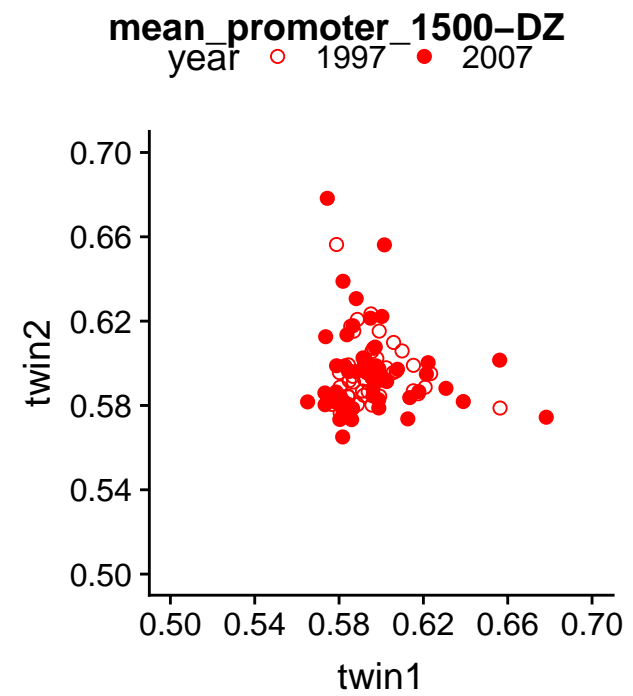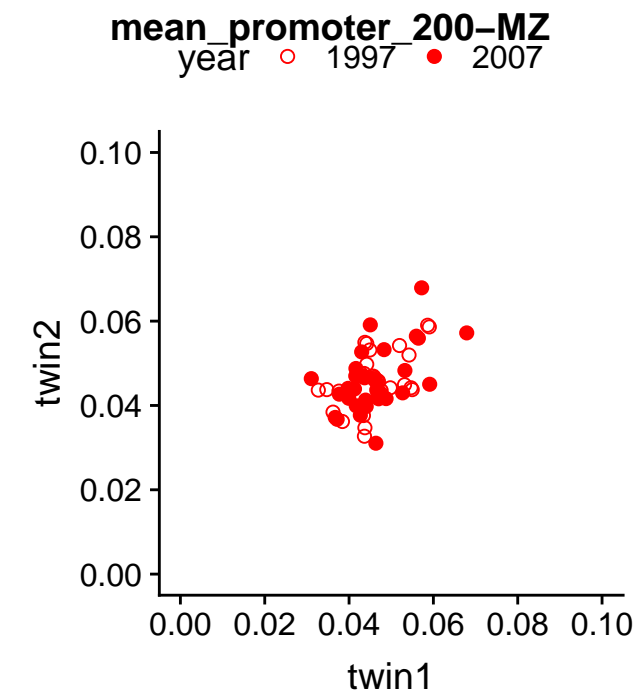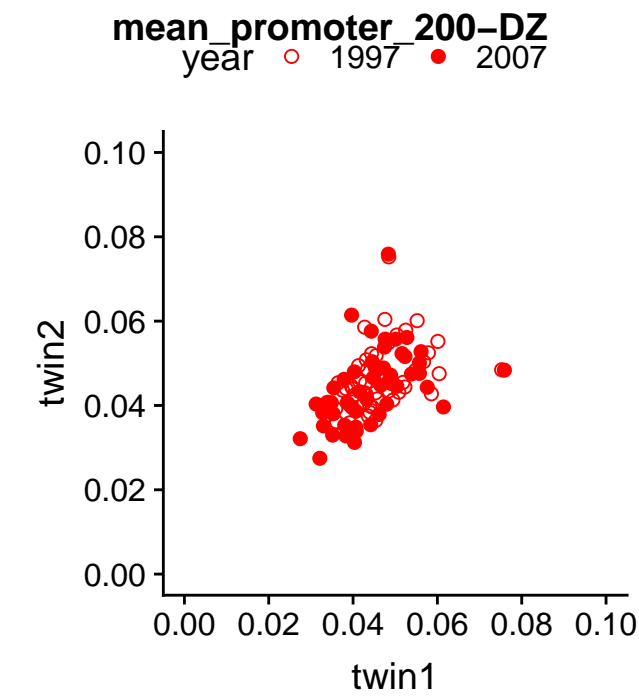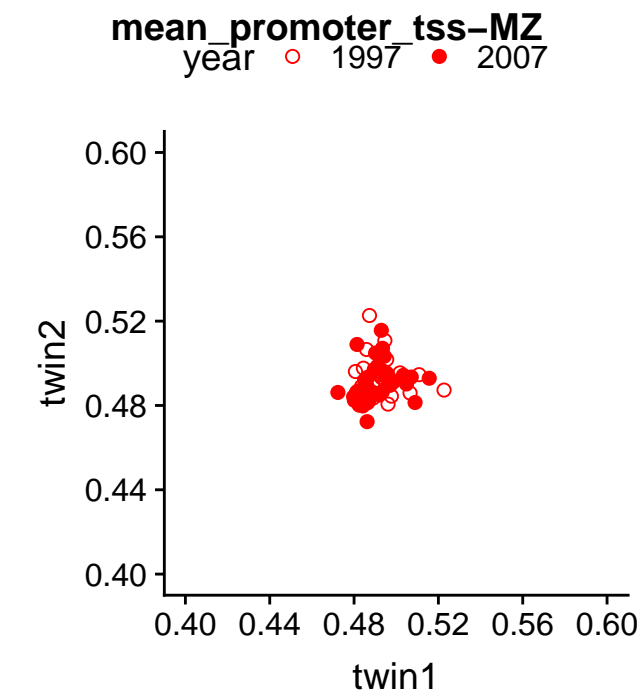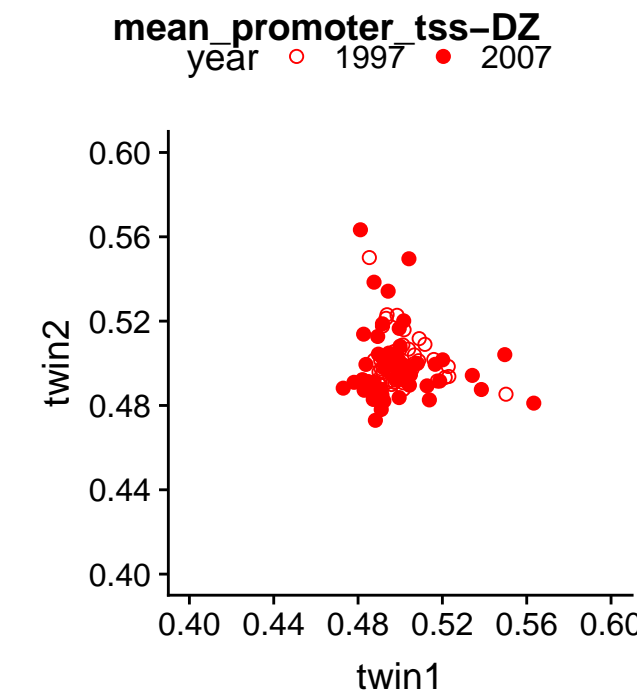

Supplement: Supplementary file 7 — Additional file 7: Figure S4. Scatter plot for MZ twins and DZ twins of the beta value for all the other non-significant CpG sites of MGMT. [file 13148_2021_1009_MOESM7_ESM.pdf]

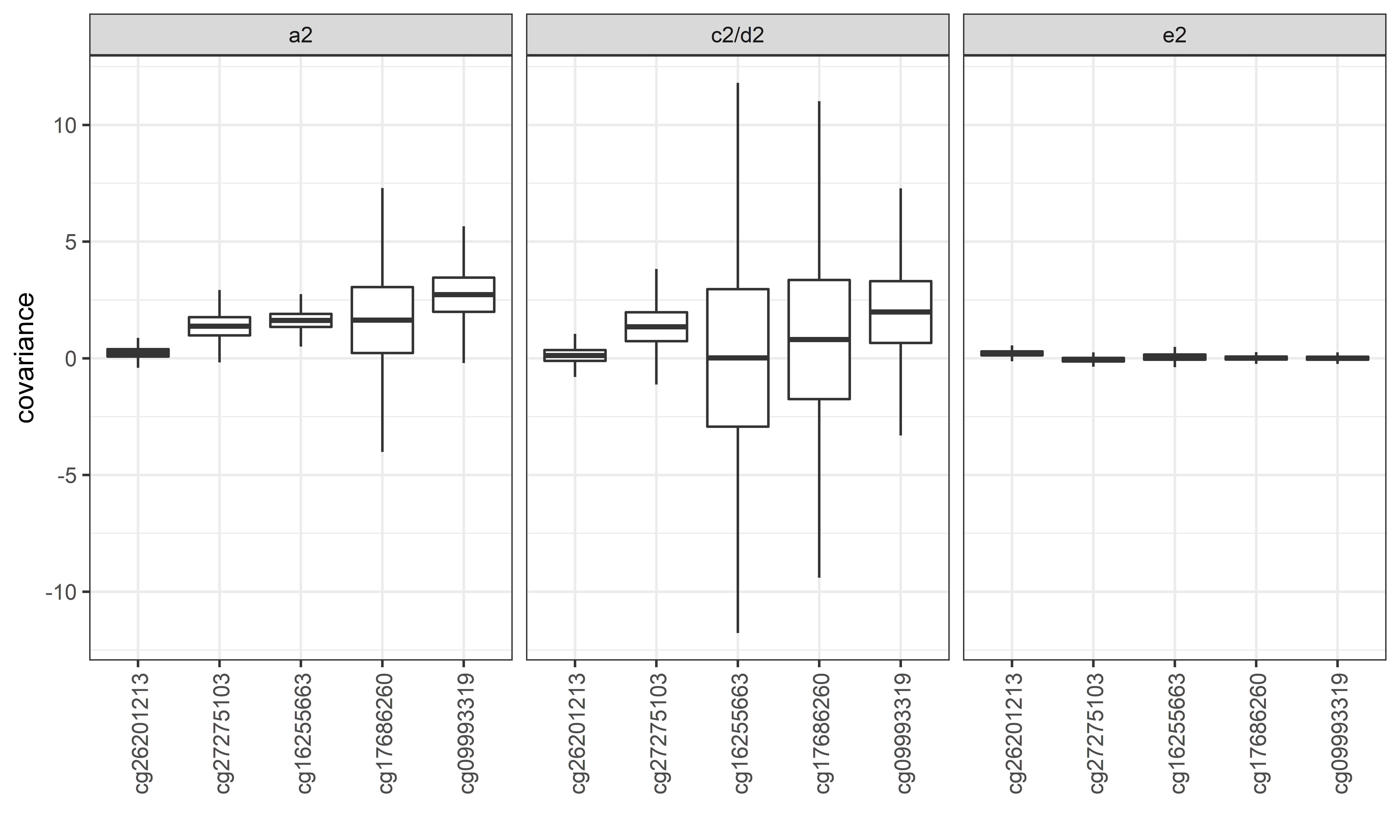

Supplement: Supplementary file 9 — Additional file 9: Figure S5. Boxplot of the 5 CpGs which are significant in bivariate twin model shows the covariance of additive genetic (a2), shared environmental (c2), dominant genetic (d2), and unique environmental proportion (e2). [file 13148_2021_1009_MOESM9_ESM.tiff]

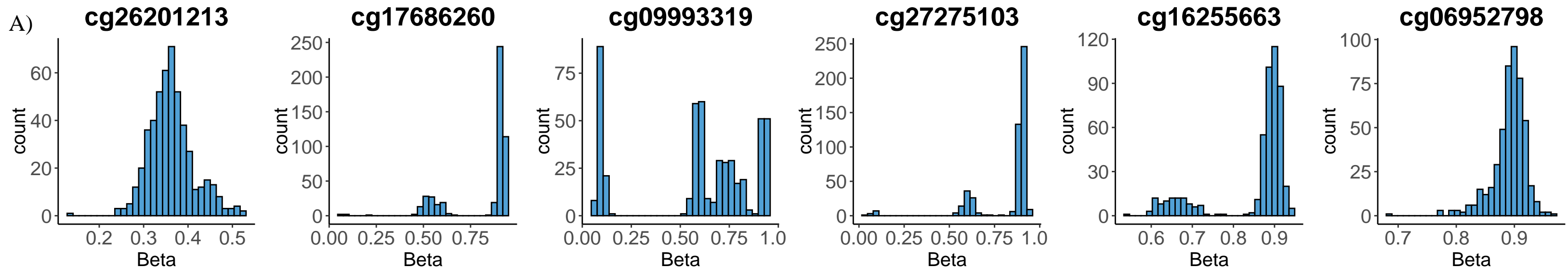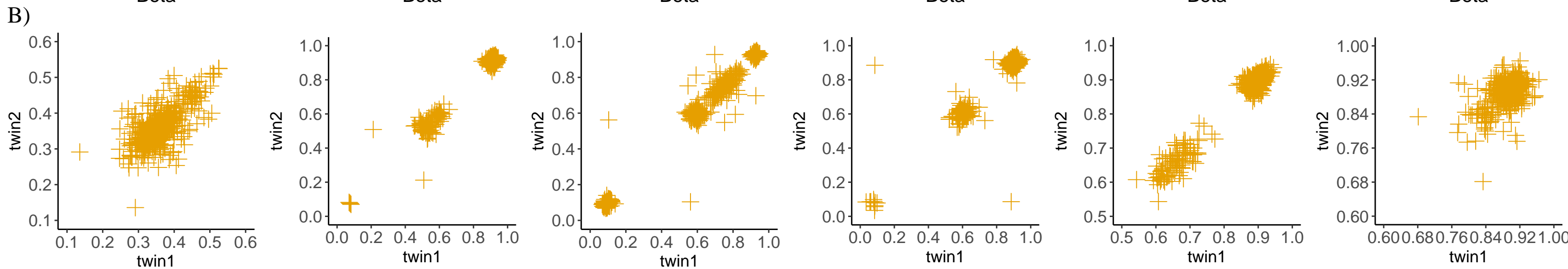

Supplement: Supplementary file 10 — Additional file 10: Figure S6. Histogram A) and scatter plot for MZ twins B) of the beta value for the 6 CpGs which are significant in both waves in MADT cohort. [file 13148_2021_1009_MOESM10_ESM.pdf]
